# Linking evolutionary mode to palaeoclimate change reveals rapid radiations of staphylinoid beetles in low-energy conditions

**DOI:** 10.1093/cz/zoz053

**Published:** 2019-10-22

**Authors:** Liang Lü, Chen-Yang Cai, Xi Zhang, Alfred F Newton, Margaret K Thayer, Hong-Zhang Zhou

**Affiliations:** 1 Key Laboratory of Zoological Systematics and Evolution, Institute of Zoology, Chinese Academy of Sciences, 1 Beichen West Rd, Chaoyang District, Beijing 100101, China; 2 College of Life Science, Hebei Normal University, No.20 Road East. 2nd Ring South, Yuhua District, Shijiazhuang, Hebei 050024, China; 3 University of Chinese Academy of Sciences, 19A Yuquan Rd, Shijingshan District, Beijing 100049, China; 4 Key Laboratory of Economic Stratigraphy and Palaeogeography, Nanjing Institute of Geology and Palaeontology, and Centre for Excellence in Life and Paleoenvironment Chinese Academy of Sciences, Nanjing 210008, China; 5 School of Earth Sciences, University of Bristol, Life Sciences Building, Tyndall Avenue, Bristol BS8 1TQ, UK; 6 Department of Parasitology, Medical College, Zhengzhou University, Zhengzhou 450052, China; 7 Integrative Research Center, Field Museum of Natural History, Chicago, IL 60605, USA; 8 Committee on Evolutionary Biology, University of Chicago, Chicago, IL 60637, USA

**Keywords:** evolutionary diversification, low-energy conditions, palaeoclimate change, rapid radiation, Staphylinoidea

## Abstract

Staphylinoidea (Insecta: Coleoptera) is one of the most species-rich groups in animals, but its huge diversity can hardly be explained by the popular hypothesis (co-radiation with angiosperms) that applies to phytophagous beetles. We estimated the evolutionary mode of staphylinoid beetles and investigated the relationship between the evolutionary mode and palaeoclimate change, and thus the factors underlying the current biodiversity pattern of staphylinoid beetles. Our results demonstrate that staphylinoid beetles originated at around the Triassic–Jurassic bound and the current higher level clades underwent rapid evolution (indicated by increased diversification rate and decreased body size disparity) in the Jurassic and in the Cenozoic, both with low-energy climate, and they evolved much slower during the Cretaceous with high-energy climate. Climate factors, especially low O_2_ and high CO_2_, promoted the diversification rate and among-clade body size disparification in the Jurassic. In the Cenozoic, however, climate factors had negative associations with diversification rate but little with body size disparification. Our present study does not support the explosion of staphylinoid beetles as a direct outcome of the Cretaceous Terrestrial Revolution (KTR). We suppose that occupying and diversifying in refuge niches associated with litter may elucidate rapid radiations of staphylinoid beetles in low-energy conditions.

## Introduction

Why there are so many beetles is a longstanding question that has drawn the persistent attention of biologists (Gould 1993; Farrell 1998; Hunt et al. 2007; McKenna et al. 2015b; Zhang et al. 2018). As one of the most species-rich superfamilies of beetles, Staphylinoidea (Agyrtidae, Hydraenidae, Leiodidae, Ptiliidae, Silphidae, and Staphylinidae) contains nearly 70,000 extant species (Supplementary Table S1) and accounts for approximately 18% of beetle diversity (Ślipiński et al. 2011). Numbers of species are dramatically uneven among (sub-)families (Supplementary Table S1), ranging from 1 (Neophoninae and Solieriinae) to over 16,000 species (Aleocharinae). Staphylinoid beetles also vary considerably in body size, from diminutive species shorter than 0.4 mm (e.g., some Ptiliidae species being the smallest known non-parasitoid insects [Grebennikov 2008]) to giants that measure up to 40 mm (e.g., some Silphidae species). Evidence from palaeontological studies (see Supplementary Table S2) and inferences made by molecular dating (McKenna et al. 2015b; Zhang et al. 2018) both reveal that staphylinoid beetles possibly appeared in the Late Triassic or the Early Jurassic. Since then, the global climate has experienced large-scale shifts (Jansen et al. 2007; Price 2009). Therefore, if climate is a pivotal element that affects the diversification of organisms as proposed by many previous theories (e.g., Erwin 2009; Bellard et al. 2012; Condamine et al. 2013), the prediction would be that the diversity dynamics of such a species-rich group should be correlated with the climatic variation during the entire history or at least in some decisive periods.

Species diversity is largely the cumulative outcome of a few radiations that produce most extant species. As a prevalent ecological explanation of speciation, adaptive radiation is characterized by a positive diversification shift (an increased speciation rate and/or a decreased extinction rate leading to species multiplication) and rapid morphological variation (Glor 2010; Losos and Mahler 2010). Climate in high-energy conditions is expected, under the species-energy hypothesis, to promote niche positions and breadth and ultimately to enhance net diversification rate (Wright 1983; Evans et al. 2005; Erwin 2009). More available and/or broader niches, in turn, are predicted to result in increased morphological variation (or disparification) (Schluter 2000; Harmon et al. 2003) as the ecologically relevant traits constrained by environmental factors respond to the opening and shrinking of new niches (Schluter 2000; Harmon et al. 2003; Glor 2010; Burbrink et al. 2012). Caraboidea (ground beetles and related) is an example in beetles, whose diversity and morphological variation expanded in the Cretaceous and contracted before and after that period (Erwin 1985). Consequently, not only diversification shifts but also morphological disparification should be taken into account when examining the impact of climate change on adaptive radiation and thus on species diversity.

In this study, we use staphylinoid beetles as a model system. We estimate the divergence time of Staphylinoidea with a time-calibrated phylogeny and detect the shifts of net diversification rate. Then, we investigate the relationship among climatic factors, diversification rate, and disparification of body size (as an ecologically relevant trait). We would expect that 1) there would be significant increase in diversification rate (radiations) during climate-changing periods and the radiations are responsible for the higher-level current diversity patterns; 2) the temporal mode of both the diversification rate and body size disparification would be correlated with climate change (at least in some critical periods); and 3) the relationship between diversification and disparification would be explainable in the light of the adaptive modes referred to in (2).

## Materials and Methods

### Estimation of divergence time

Bayesian relaxed clock methods (e.g. BEAST, MrBayes) are popular for inferring tree topology and estimating divergence times simultaneously, but computational burden prevents their use for large datasets. We therefore built a phylogeny of Staphyliniformia and Scarabaeoidea (distant outgroups) with 664 terminal taxa and estimated the substitution rate per site using RAxML v8.2.10 (Stamatakis 2014) (see details in Supplementary Appendices S1 and S3). We then used the program r8s v1.80 (Sanderson 2003) to date the maximum likelihood tree (Supplementary Figure S1) with the penalized likelihood (Sanderson 2002) method with an additive penalty function and TN Algorithm. We used 50 fossils to calibrate the phylogeny (Supplementary Table S2, Supplementary Figure S2). The recent limit of the geological age of the stratum where the fossils were found was used as minimum age and the boundary age of Upper and Lower Cretaceous (100.5 million years ago (Ma)) and that of Triassic and Permian (252 Ma) were used as maximum ages (soft bound) for the Cenozoic fossils and the Mesozoic fossils, respectively. When the time-scaled phylogeny was created, we used R 3.3.1 (R Core Team 2016) with APE package (Paradis et al. 2004) to resolve the chronogram randomly (2 polytomies were collapsed by r8s for very short branches) and pruned the tree into a genus-level one for analyses regarding body size.

### Shifts of diversification rate

We detected the diversification shifts using BAMM 2.5 and the associated R package BAMMtools (Rabosky et al. 2013; Rabosky et al. 2014; Rabosky 2014), which offer program/functions for modelling dynamics of speciation (λ) and extinction (μ) by reversible jump Markov chain Monte Carlo (MCMC) method and thus detect shifts in net diversification rates (*r*  =  λ–μ). We used the dataset that associates (sub-/super-)family richness (Supplementary Table S1) with the relevant clades on the tree (distant outgroups dropped). Omaliinae, Empelinae, Glypholomatinae, and Microsilphinae were clustered together, so their species richness was used in summation (OMA + EMP + GLY + MICS). Protopselaphinae were not sampled, thus its richness was attached to Pselaphinae (PSE + PROP). The richness of Phloeocharinae was averaged into each of the 2 parts, and the data of Osoriinae, Tachyporinae, and Leiodidae were counted according to cladal divisions. Because this dataset includes all the subgroups of the superfamily and the complete species richness data, but some clades on the tree are incompletely sampled, we set the global sampling probability to 1.0, and the clade-specific sampling probabilities are calculated by program with cladal richness and number of tips. We expected 30 shifts and left other parameters in their default settings. We performed 4 MCMC runs for 100 million generations per run, sampled every 10,000 generations, and dropped the first 20% (stably convergent thereafter and effective sample size of all parameters > 200) when inputting the results for the subsequent analyses. The best shift model (expected shifts = 33, core shifts = 11, distinct threshold = 35) was selected by Bayesian factor (1,211.97 over the null model and 65.81 over the second) and posterior probability (0.099). The net diversification rate of each lineage was averaged across all shift conﬁgurations sampled during simulation of the posterior, and diversification rate variation through time (DRTT) of both Staphylinoidea and Staphyliniformia (including near outgroups) was calculated by median of lineage rates (Rabosky et al. 2014).

### Evolution and disparification of body size

We compiled a body size dataset of 5,364 species representing 218 genera (out of 4,268 staphylinoid genera) that appeared in the genus-level tree. Body size here indicates body length, which is commonly documented in the literature on staphylinoid beetles. The dataset was built on the data extracted from 1,143 taxonomic publications (see Supplementary Appendix S4) as well as data accumulated in our laboratory by the following protocols: 1) typically, we directly used the mean or unique value provided by the author; 2) when body length was provided as a range, we calculated the mid-value (arithmetic mean of the extremes); 3) when it was provided as a sample of individual values, we calculated and used the mean; 4) in a few cases, scaled images of the specimens were offered but there was no data in the text, we thus measured the images to obtain the data required. The extracted body length data were multiplied by a factor of 10 (in case of any value < 1 mm) and then naturally log-transformed. We then calculated the mean of the ln (10 × body length), the standard deviations, and the standard errors for each genus (Supplementary Table S3).

We analyzed body size at the genus level, using the genus-level tree and the mean and standard error values for each genus. Here we considered only the ingroup taxa (genera of Staphylinoidea). We used the lambda model to detect the phylogenetic signal of body size by the function “fitContinuous” in GEIGER (Harmon et al. 2008); when λ  =  1, it recovers the Brownian motion (BM) model (strong phylogenetic signal); when λ  =  0, there is no phylogenetic signal (Münkemüller et al. 2012).

We evaluated the disparification and compared it among clades and within clades by a disparity through time (DTT) plot and quantitatively by the morphological disparity index (MDI) (Harmon et al. 2003). The disparity of a lineage is the mean of all the elements in the distance matrix of the traits of 2 subclades generated from that lineage. The relative disparity is the disparity of a lineage divided by the disparity of the entire tree. A DTT plot depicts the average relative disparity of all the lineages appearing at each node age, in which both the observed and simulated (10,000 times under BM model) disparities are calculated and drawn (Harmon et al. 2003; Slater et al. 2010). MDI is the overall difference in the relative disparity of a clade compared with that expected under the BM model (Harmon et al. 2003; Slater et al. 2010). These calculations were performed using the GEIGER package (Harmon et al. 2008). The disparity curve being beneath the simulation line (or a negative MDI value) suggests that among-clade disparification surpasses within-clade disparification, and the opposite situation suggests a predominance of within-clade disparification. Significantly larger among-clade disparification indicates an adaptive radiation, if the trait fits the open/broadened niche. Under the hypothesis of adaptive radiation, one can expect that both diversification and among-clade trait disparification would increase with the filling of the available ecological niches and then they would slow down after saturation of the niches (Harmon et al. 2003; Slater et al. 2010).

For a given tree, however, the DTT curve always goes from 1 to 0 and fluctuates in between (despite values larger than 1). To eliminate the inherent 1-to-0 direction of DTT, we calculated “disparity deviations,” an index derived from the difference between the observed disparity of each node and the simulated one. Under the null model, the timing of the disparity deviation should be a constant line at 0, and alternatively, the stronger the influence of ecological factors is, the deeper the trough of the disparity deviation curve will be. The disparity deviation, measuring disparification of body size, is suitable for evaluating the ecological effect on adaptive radiations.

### Effect of climate

Climate is a complicated set of variables, but herein we simply considered temperature, O_2_, and CO_2_ as the markers of climate change. Data concerning temperature deviations relative to today (ΔT in °C) and atmospheric CO_2_ concentration (cCO_2_ in p.p.m.) were published in Royer et al. (2004) and provided by Dr. D. L. Royer (Wesleyan University, USA). The atmospheric oxygen percentage data (pO_2_ in %) were extracted from Berner (2009). We then performed a natural log-transformation for cCO_2_ and a logit transformation for pO_2_, followed by cubic spline interpolations of all three climate variables to harmonize the timing of the climate dataset with that of disparity deviation. We correlated the net diversification rates and the disparity deviations, respectively, with the climate factors to assess the impact of these climate factors by using generalized least square (GLS) in the NLME package, treating the time (age) as a continuous time covariate. We accounted for all 7 combinations of the climate factors and selected the best-fitting model using the Akaike Information Criterion (AIC) and Akaike weight (*w*). The models with difference of AIC from the minimum AIC (dAIC) less than 2 units are also considered. We assessed the goodness-of-fit of each model by likelihood-ratio-based pseudo-R-square (*R*_pse_^2^) (MuMIn package). We also tested the statistical significance of the regression coefficients (*b*) for all the models and calculated the standardized regression coefficients (β) for the best-fitting model(s). Climate factors with high standardized regression coefficients have strong explanatory power. We investigated the correlation between the net diversification rate and the disparity deviations by using Pearson’s correlation coefficient (cor). Both the curve of diversification rate and the curve of disparity deviations are obviously nonmonotonic, so we separated the whole history of staphylinoids into 3 stages: early (Jurassic, root age to 145 Ma), middle (Cretaceous, 145–66 Ma), and late (Cenozoic, 66 Ma to present), and performed the correlation tests for all the 3 stages. All these calculations were executed in R.

## Results

### Phylogeny and divergence times of higher level taxa

**Figure 1.**
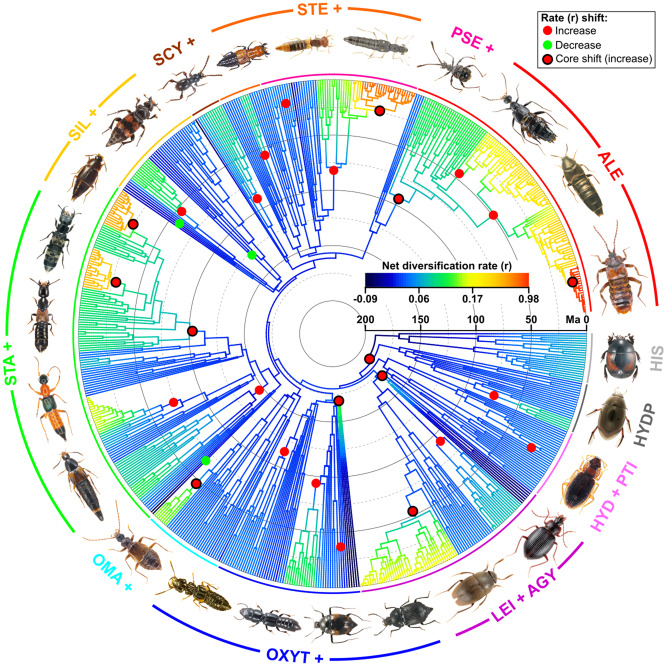
Radial chronogram of Staphylinoid phylogeny (without distant outgroups). Tip labels are omitted. Branch colors indicate model-averaged net diversification rates (increasing from cold colors to hot colors), and the spots indicate the nodes where shifts of diversification rate occur according to BAMM results (red—rate increases, green—rate decreases, red with black border—“core shifts” with increase in rate). Expanded view with tip labels is shown in Supplementary Appendix S2: Figure S3. Dashed and solid concentric circles indicate every 50 Myr before present. Arcs with different colors indicate the major ingroup clades, and gray arcs indicate the near outgroups. Photos of some exemplars are attached nearby and unscaled (Photos of *Tropisternus* sp., *Hydraena* sp. and *Alzadaesthetus* sp. © Field Museum of Natural History; *Aleochara* sp. © Tian-Hong Luo; *Creophilus* sp. © Liang He; *Ptomaphagus* sp. and *Stenus* sp. © Cheng-Bin Wang; *Apteroloma* sp. © Liang Tang; *Tachinus* sp. © Zi-Wei Yin. All used with permission).

**Figure 2.**
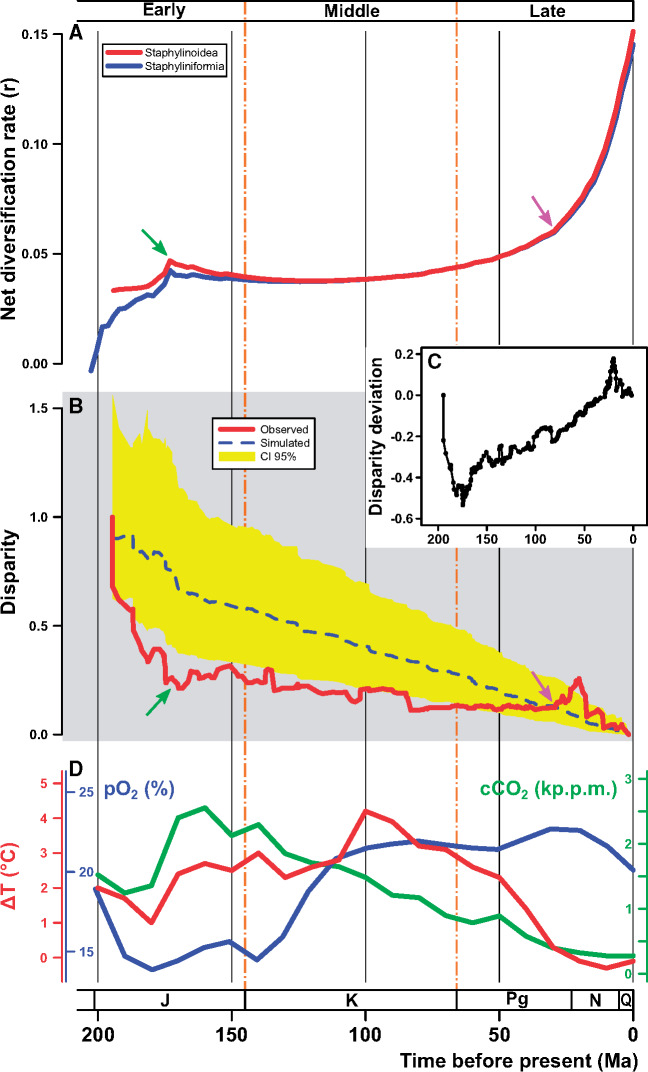
Diversification tempo, adaptive radiation, disparification, and climate changes. (**A**) Net diversification rate variation through time; blue line depicts trajectory of Staphyliniformia (including near outgroups), red line depicts that of Staphylinoidea (ingroups only). Green arrow indicates the crest of diversification rate in the middle of the Jurassic. Fuchsia arrow indicates the inflection point where the rate starts exponential increase. (**B**) DTT plot of Staphylinoidea. Red solid line depicts observed curve and blue dashed line depicts simulated curve under BM model. CI 95% is the 95% confidence interval of the simulated curve (yellow area). Green arrow indicates the trough of disparity in the middle of the Jurassic. Fuchsia arrow indicates the point where the observed curve leaps over the simulated curve. (**C**) Disparity deviation (DD). (**D**) Variations of temperature (ΔT in red), oxygen (pO_2_ in blue), and carbon dioxide (CO_2_ in green) since the last 200 Myr. Two vertical dot-dashed lines (145 Ma and 66 Ma, respectively) separate the whole history into three stages: early (Jurassic), middle (Cretaceous), and late (Cenozoic). Era abbreviations: J, Jurassic; K, Cretaceous; Pg, Palaeogene; N, Neogene; Q, Quaternary.

Our estimation depicts the timing of major diversification events in the early history of staphylinoid beetles (Supplementary Table S1, Figures 1 and 2A, and see detail in Supplementary Figure S2). The ancestor of staphylinoid beetles was estimated to have appeared 199.3 Ma, and the basal split occurred 194.6 Ma (very beginning of the Jurassic). All 6 families (Silphidae nested into Staphylinidae) appeared and started radiating in the Middle Jurassic or earlier. Most of the main subfamilies of Staphylinidae have their origins in the Jurassic, but some hyperdiverse subfamilies, such as Aleocharinae, Pselaphinae, Staphylininae (Staphylinini), and Paederinae, emerged in the Late Jurassic or the Early Cretaceous. Since many recent studies have discussed relationships among the higher level taxa and our study focuses on climatic influence on the early radiations of Staphylinoidea, we detail the results and discussion of our phylogenetic analysis in Supplementary Appendix S3. Here we tentatively divide Staphylinoidea into 10 major clades: 1) Hydraenidae + Ptiliidae, 2) Leiodidae + Agyrtidae, 3) “Oxytelinae +”, 4) “Omaliinae +”, 5) “Staphylininae +”, 6) “Silphidae +”, 7) “Scydmaeninae +”, 8) “Steninae +”, 9) “Pselaphinae +”, and 10) Aleocharinae.

### Diversification shifts and climate change

We investigated the tempo of diversification by detecting the branches and the time at which the diversification rate changes. The best-fitting model of BAMM recognizes 29 rate shifts (excluding the root process) on our tree, and 11 of them are “core” shifts (Figure 1 and Supplementary Figure S3). Our result shows that the diversification process of staphyliniform beetles was fueled by multiple radiations (where diversification rate increases). The oldest shift (core, marginal probability = 0.827) took place at the most recent common ancestor (MRCA) of Staphylinoidea and Hydrophiloidea, but within the clade Hydrophiloidea there were no core shifts. Four increased shifts (3 core shifts) occurred in the early stage; 11 (2 core, 2 decreased) occurred in the middle stage; 14 (6 core, 1 decreased) occurred in the late stage. Among the 10 major clades, the Hydraenidae + Ptiliidae, “Silphidae +”, and “Steninae +” clades did not show core rate shifts, but each of the other 7 clades possessed at least 1 core shift. A basic pattern is that the diversification rate in basal groups is prone to decrease (Figure 1 and Supplementary Figure S3, cold colored) and all the decreasing shifts occurred in basal groups of relevant clades or subclades, for example, Agyrtidae (though its rate increased for a while at first), Apateticinae, Habrocerinae, Trichophyinae, Trigonurinae, Olisthaerinae, Phloeocharinae (*Phloeocharis*), Solieriinae, and Neophoninae; whereas the rate in derivative groups is prone to increase (Figure 1 and Supplementary Figure S3, hot coloured), for example, the cave-living Leiodidae (Leptodirini minus *Platycholeus*), some Scaphisomatini (Scaphidiinae), some Omaliinae (plus Empelinae), Xantholinini + Maorothiini (Staphylininae), Philonthina (Staphylininae), Xanthopygina (Staphylininae), Clavigeritae (Pselaphinae), and the “higher” Aleocharinae (especially the clades embracing Athetini, Tachyusini, Lomechusini, Pygostenini, Oxypodini, and Liparocephalini).

The temporal mode of rate evolution (Figure 2A) shows that the net diversification rate rose rapidly in the Early Jurassic and peaked in the middle of the Jurassic, then kept stable and even fell slightly during the entire Cretaceous, but climbed exponentially since the Paleogene.

### Climate change impacts on both diversification rate and body size disparification

The evolution of body size strongly fits the lambda model. The λ value (0.9708), which is close to 1, indicates a high phylogenetic signal in the body size of staphylinoid beetles and the evolution of body size closely fits the BM model. The DTT plot (Figure 2B) shows the observed disparity steeply declined since the origin of Staphylinoidea until the middle of the Jurassic and it was clearly lower than the simulated disparity in most of staphylinoid history (MDI = −0.196). In the late Paleogene (∼30 Ma, roughly Oligocene), the observed disparity leapt over the simulated curve. Pearson’s correlation supports that the relationship, in fact, has different patterns among 3 stages: the diversification rate had a negative correlation with the disparity deviation (cor = −0.3644, *P *=* *0.009) before 145 Ma but had positive correlations in 145–66 Ma (cor = 0.5799, *P *<* *0.001) and after 66 Ma (cor = 0.5458, *P *<* *0.001).

**Figure 3.**
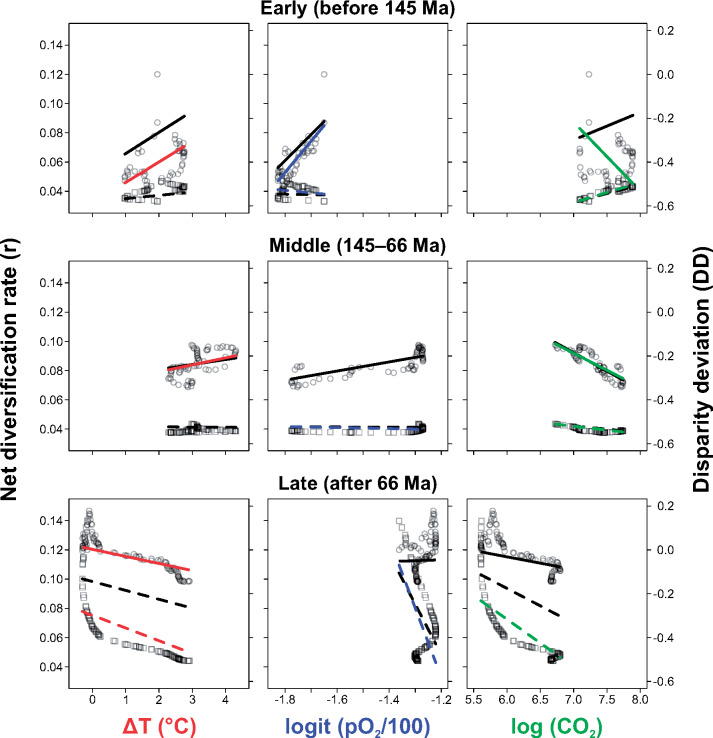
GLS regression of net diversification rate (*r*) versus climate factors (squares and dashed lines) and disparity deviation (DD) of body size versus climate factors (circles and solid lines). Black lines depict the trends with single climate predictors, and colored lines depict the (partial) trends of the individual climate predictor(s) in the best-fitting model (see Table 1).

The AIC-based model selection (Figure 3; Table 1) also shows different patterns among 3 stages. In the early stage, the best-fitting model (*R*_pse_^2^ = 0.974, *w* = 0.556) shows that the net diversification rate is significantly and negatively associated with pO_2_ (*b* = −0.0172, β = −0.2372, *P *=* *0.0037), but positively associated with cCO_2_ (*b *=* *0.0138, β  =  0.9932, *P *<* *0.001). Meanwhile, the disparity deviation is correlated to all the 3 factors (*R*_pse_^2^ = 0.877, *w* = 0.715), among which pO_2_ (*b *=* *1.4228, β  =  0.7957, *P *<* *0.001) has a significantly positive association, whereas cCO_2_ (*b* = −0.3142, β = −0.9314, *P *<* *0.001) has a negative association and the strongest power (ΔT is not significant). The second best-fitting model (without ΔT) shows the same pattern. In the middle stage, the net diversification rate has a significantly negative association with both pO_2_ (*b* = −0.0035, β = −0.4328, *P *<* *0.001) and cCO_2_ (*b* = −0.0050, β = −0.8904, *P *<* *0.001), according to the best-fitting model (*R*_pse_^2^ = 0.994, *w* = 0.727). The disparity deviation has a significantly positive association with ΔT (*b *=* *0.0318, β  =  0.3106, *P *=* *0.0036) and a negative association with cCO_2_ (*b* = −0.1551, β = −0.7591, *P *<* *0.001). In the late stage, the net diversification rate is significantly and negatively associated with all the 3 climate factors (*R*_pse_^2^ = 0.999, *w* = 0.992) but the disparity deviation is negatively associated with only ΔT (*b* = −0.0323, *P *=* *0.0409).

**Table 1.** GLS estimation of the models of net diversification rate (*r*) versus climate factors and disparity deviation (DD) of body size versus climate factors, according to the AIC-based selection (ascending order of AIC)

Response	Explanatory	*R*_pse_^2^	AIC	dAIC	*w*
**Early (before 145 Ma)**					
r	− **0.0172** pO_2_ + **0.0138** cCO_2_ − **0.0949**	0.974	−598.545	0.000	0.556
	− **0.2372** pO_2_ + **0.9932** cCO_2_ (#)				
	− 0.0014 ΔT − **0.0142** pO_2_ + **0.0163** cCO_2_ − **0.1053**	0.974	−597.472	1.073	0.325
	− 0.2290 ΔT − **0.1921** pO_2_ + **1.1733** cCO_2_ (#)				
	− **0.0029** ΔT + **0.0181** cCO_2_ + **0.0910**	0.972	−594.837	3.709	0.087
	**0.0117** cCO_2_ − 0.0490	0.970	−592.863	5.682	0.032
	**0.0036** ΔT − **0.0164** pO_2_ + 0.0020	0.964	−582.293	16.253	0.000
	**0.0023** ΔT + 0.**0327**	0.961	−580.017	18.529	0.000
	− 0.0030 pO_2_ + **0.0326**	0.957	−575.361	23.185	0.000
DD	0.0934 ΔT **+ 1.4228** pO_2_ − **0.3142** cCO_2_**+** 4.3012	0.877	−193.212	0.000	0.715
	0.6246 ΔT **+ 0.7957** pO_2_ − **0.9314** cCO_2_ (#)				
	**1.8573** pO_2_ − **0.1610** cCO_2_**+ 4.0993**	0.867	−191.254	1.958	0.268
	**1.0396** pO_2_ − **0.4773** cCO_2_ (#)				
	**1.1854** pO_2_ + **1.7410**	0.842	−184.720	8.492	0.010
	− **0.0645** ΔT **+ 2.0034** pO_2_ + **3.2744**	0.845	−183.607	9.605	0.006
	0.0967 ΔT − **0.4580**	0.820	−177.989	15.223	0.000
	**0.2805** ΔT − **0.6035** cCO_2_ + **3.6081**	0.827	−177.956	15.257	0.000
	0.1261 cCO_2_ − 1.1817	0.810	−175.254	17.958	0.000
**Middle (145–66 Ma)**					
r	− **0.0035** pO_2_ − **0.0050** cCO_2_ + **0.0722**	0.994	−1073.633	0.000	0.727
	− **0.4328** pO_2_ − **0.8904** cCO_2_ (#)				
	0.0000 ΔT − **0.0035** pO_2_ − **0.0050** cCO_2_ + **0.0722**	0.994	−1071.637	1.996	0.268
	0.0025 ΔT − **0.4334** pO_2_ − **0.8911** cCO_2_ (#)				
	− **0.0041** cCO_2_ + **0.0711**	0.993	−1062.802	10.831	0.003
	− 0.0001 ΔT − **0.0041** cCO_2_ + **0.0711**	0.993	−1060.904	12.729	0.001
	− 0.0001 ΔT + **0.0418**	0.989	−1026.695	46.939	0.000
	− 0.0006 pO_2_ + **0.0405**	0.989	−1026.445	47.188	0.000
	− 0.0001 ΔT − 0.0006 pO_2_ + **0.0410**	0.989	−1024.909	48.724	0.000
DD	**0.0318** ΔT − **0.1551** cCO_2_ + **0.7980**	0.929	−381.926	0.000	0.642
	**0.3106** ΔT − **0.7591** cCO_2_ (#)				
	**0.0315** ΔT + 0.0028 pO_2_ − **0.1537** cCO_2_ + **0.7930**	0.929	−379.929	1.998	0.236
	**0.3075** ΔT + 0.0114 pO_2_ − **0.7510** cCO_2_ (#)				
	− **0.1681** cCO_2_ + **0.9917**	0.922	−377.590	4.337	0.073
	0.0669 pO_2_ − **0.1357** cCO_2_ + **0.8511**	0.923	−376.438	5.488	0.041
	**0.2075** pO_2_ + 0.0632	0.914	−371.191	10.735	0.003
	0.0225 ΔT − **0.3065**	0.914	−370.551	11.375	0.002
	0.0217 ΔT + **0.1867** pO_2_ − 0.0342	0.916	−370.544	11.382	0.002
**Late (after 66 Ma)**					
*r*	− **0.0087** ΔT − **0.4732** pO_2_ − **0.0324** cCO_2_ − **0.3244**	0.999	−1105.852	0.000	0.992
	− **0.4361** ΔT − **0.7230** pO_2_ − **0.6180** cCO_2_ (#)				
	− **0.4586** pO_2_ − **0.0518** cCO_2_ − **0.1967**	0.999	−1096.278	9.574	0.008
	− **0.0203** ΔT − **0.4892** pO_2_ − **0.5311**	0.999	−1083.123	22.729	0.000
	− **0.3442** pO_2_ − **0.3647**	0.996	−962.963	142.889	0.000
	0.0139 ΔT − **0.0556** cCO_2_ + **0.4140**	0.992	−903.819	202.033	0.000
	− **0.0239** cCO_2_ + **0.2368**	0.992	−902.045	203.807	0.000
	− 0.0061 ΔT + **0.0984**	0.992	−896.594	209.258	0.000
DD	− **0.0323** ΔT + 0.0025	0.973	−542.586	0.000	0.305
	− 0.0575 cCO_2_ + 0.3132	0.973	−541.856	0.730	0.212
	− 0.0522 ΔT + 0.0475 cCO_2_ − 0.2665	0.974	−540.737	1.849	0.121
	− 0.8171 ΔT + 0.3146 cCO_2_ (#)				
	0.0396 pO_2_ + 0.0020	0.973	−540.708	1.878	0.119
	− 0.0326 ΔT − 0.0835 pO_2_ − 0.1059	0.973	−540.657	1.929	0.116
	− 0.4627 ΔT − 0.0225 pO_2_ (#)				
	− 0.0422 pO_2_ − 0.0587 cCO_2_ + 0.2656	0.973	−539.874	2.712	0.079
	− 0.0532 ΔT – 0.0961 pO_2_ + 0.0509 cCO_2_ − 0.4109	0.974	−538.830	3.756	0.047

The hash-signed (#) expressions show the standardized regression coefficients of the best-fitting models (multiple regressions only). *R*_pse_^2^, likelihood-ratio-based pseudo-R^2^ of each model; AIC, Akaike information criterion; dAIC, difference of AIC from the minimum AIC; w, Akaike weight. The estimates in bold type indicate the statistical significance (*P *<* *0.05) of relevant coefficients.

## Discussion

### Age of staphylinoid beetles

The result of molecular clock indicates the MRCA of staphylinoid beetles possibly lived at the very beginning of the Jurassic (194.6 Ma). This is older than, but does not contradict, the oldest fossil record *Ochtebiites minor* (Ponomarenko 1985), a Hydraenidae species that was present 191–183 Ma. A Triassic fossil was described as the oldest staphylinid species, *Leehermania prorova* (Chatzimanolis et al. 2012), but it is recently moved to Myxophaga by Fikáček et al. (2019). Currently there are no staphylinid fossils having been found in the Triassic strata. As another *a posteriori* test, we did not calibrate the clade of Paederinae; their resulting age is 147.6 Ma, older than *Mesostaphylinus* spp. (Yixian formation, 125 Ma), the oldest paederine fossils (Solodovnikov et al. 2013). As to the previous molecular results, McKenna et al. (2015b), under the frame of the whole Coleoptera, estimated the age of the MRCA of staphylinoids as 193.16 (210.26–175.26) Ma. Toussaint et al. (2017) questioned it and dated the MRCA of staphylinoids at 280.42 (294.88–265.46) Ma by using a different set of fossil calibrations. Zhang et al. (2018) used extensive sampling of genes and another set of fossils, which estimated the age as 199.4 (192.0–207.6) Ma. Different time priors, applications (BEAST or MCMCTREE), and calibrating positions (on stem or crown) may be responsible for their discrepancy. The results of McKenna et al. (2015b) and Zhang et al. (2018) are closer and close to our results. Unlike the 3 previous studies, in which the clade Staphylinoidea were calibrated and constrained, we left the MRCA node free and dated it by the expanded sampling of species and more time priors which were evenly placed across the tree, on deep and shallow subordinary nodes. Not including Jacobsoniidae in our study would not significantly change the result, because this family has a small number of species and is nested within Staphylinoidea (not a sister group) (see McKenna et al. 2015b; Zhang et al. 2018). Another older estimate by Zhang and Zhou (2013) dated the family Staphylinidae back to the Early Triassic epoch (243.35 Ma). This overestimation was caused by the poorly built phylogeny and inappropriate calibrations. From both fossil records and molecular clock studies, we can safely draw the conclusion that staphylinoid beetles were probably present at around the Triassic–Jurassic bound and started radiating in not earlier than the beginning of the Jurassic.

### Climatic impact on staphylinoid evolution: a 3-staged pattern

Our results indicate that the significant radiations of staphylinoids tend to take place in the period of changing (cooling) climate (see the first expectation in the Introduction). The three relevant climate factors of interest experienced wide but synchronous fluctuations during the period in question (Figure 2D). The result of diversification rate shifts shows that most of the rate increases (9 of 11 core increases) occurred in the Jurassic and the Cenozoic. Most of modern staphylinoid (sub-)family-level taxa had been present by the Middle Jurassic (a short period of about 30 million years (Myr) after the 200-Ma initial radiation), although Aleocharinae, Staphylininae, Paederinae and Pselaphinae were absent (Figure 1 and Supplementary Figure S3). These current species-rich groups and other two (Leiodidae and Scydmaeninae) comprise the groups with more than 4,000 species in Staphylinoidea, and their diversity was largely produced by the Cenozoic radiations (Figure 1 and Supplementary Figure S3). Zhang et al. (2018) detected the radiations using a family-level beetle tree but failed to find any radiations in the staphylinoid clade, which is partly due to the method and incomplete sampling (May and Moore 2016). Despite lack of reliable molecular clock studies on the subgroups of Staphylinoidea, our results can be tested by many palaeontological studies (see the literature cited in Supplementary Table S2). For example, Solodovnikov et al. (2013) studied the fossil fauna of Staphylininae and supposed that the hyperdiverse groups of Staphylininae originated later than the Early Cretaceous, which is also supported by this study.

Our findings also reveal the correlations between climate change and the diversification rate and between climate change and body size disparification (see the second expectation in the Introduction). Specifically, the 3 climatic factors have different patterns of impact on the evolution of staphylinoid beetles in the 3 stages of their history (Figure 3; Table 1). O_2_ and CO_2_ have opposite effects on both diversification rate and body size disparification in the early stage (before 145 Ma), that is, low O_2_ and high CO_2_ would promote net diversification rate and among-clade disparification. Considering the negative correlation between net diversification rate and disparity deviation, we suppose the among-clade disparification should be positively associated with the increase of diversification rate. Our results indicate an adaptive radiation, which is characterized by a rise of diversification rate and an increase of among-clade morphological disparification (Figure 2B) (Schluter 2000; Harmon et al. 2003; Glor 2010), under the influence of the decrease of O_2_ and the increase of CO_2_ in the early history of staphylinoid beetles. The effect of temperature on net diversification rate in the early stage is similar to O_2_ in function and explanatory power, but it is not supported by the statistical test (Table 1). In our GLS result, the addition of climate factors did not enhance the *R*_pse_^2^ of the polynomial model (Table 1), which indicates high collinearity of the historical climate factors. Nevertheless, our results demonstrated that low O_2_ and high CO_2_ played prominent roles in the increase of the diversification and among-clade disparification in the Jurassic, which explain the early radiations. The diversification of non-phytophagous beetles, including staphylinoid beetles, is unable to be explained by the rise of angiosperms and the most species-rich predacious families (Staphylinidae and Carabidae) are not originated in Cretaceous (Hunt et al. 2007; Zhang et al. 2018). Our climatic explanation provides a new insight into this issue.

The middle stage (145–66 Ma) is a “watershed” for staphylinoid beetles. Both net diversification rate and disparity deviation are negatively associated with CO_2_ (Figure 3; Table 1). The increasing disparity deviation suggests that the ecological opportunity that enhanced diversification in the early stage has been saturated and body size evolution is in a transition from among-clade to within-clade disparification in this period. In addition, the net diversification rate changes little and this stage has the comparatively lowest proportion of core shifts (2 out of all 11 core shifts) and the most decreased shifts (2 of all 3 decreased shifts) (Figure 1 and Supplementary Figure S3). Nevertheless, we find that the curve of median net diversification rate transforms from a logarithmic curve into an exponential curve (Figure 2A) and more lineage color changes can be observed in this period (Figure 1). These illustrate that the diversification rate was discriminating between species-rich and species-poor groups. Many studies have made a conjecture that the land-dwelling animals experienced a similar Cretaceous Terrestrial Revolution (KTR) (125–80 Ma), during which the replacement of ferns and gymnosperms by angiosperms and the subsequent explosion of the latter provided new evolutionary opportunities for pollinating insects (Grimaldi 1999), squamates (Evans 2003; Longrich et al. 2012), and mammals (Meredith et al. 2011; Grossnickle and Polly 2013) and drove them to diversify rapidly. Some studies, however, denied this conjecture in particular groups, for example, dinosaurs (Lloyd et al. 2008). Whether the success of beetles is the outcome of KTR remains in dispute. Hunt et al. (2007) supposed that the extreme variety of niches is the reason. Zhang et al. (2018) found that most of coleopteran families (most are herbivorous) fit the KTR model, which explains the diversification upsurge of the whole beetle tree in the Cretaceous. Although our results do not support any direct linkage of staphylinoid radiation with KTR (in agreement with Zhang et al. [2018]; most staphylinoids do not eat plants), the Cretaceous origins of the groups (e.g., Aleocharinae and Pselaphinae) that would diversify in the Cenozoic does make sense in the present diversity of staphylinoids.

In the late stage (after 66 Ma), the net diversification rate, rocketing up under an accelerating and exponential mode, is positively correlated with within-clade disparification and negatively associated with all 3 climate factors, whereas within-clade disparification is associated only with temperature (negatively) (Figure 3; Table 1). However, the Cenozoic radiations occurred locally and the uneven pattern of diversification rate had simultaneously become approximately consistent with that of current diversity (Figure 1 and Supplementary Figure S3). The most striking rate increases belong to Leptodirini, Philonthina, Xanthopygina, Clavigeritae, and some derivative (higher) Aleocharinae clades (embracing Athetini, Tachyusini, Lomechusini, Pygostenini, and Oxypodini) (Figure 1 and Supplementary Figure S3). All of them started radiating in the Palaeogene, when the within-clade disparification of body size increased over the among-clade disparification (Figure 2). Therefore, it is reasonable to interpret their proliferation as the outcome of interspecific competition (the predator species) or as the outcome of habitat/host isolation (the rest include major groups of endoparasites, myrmecophiles, termitophiles, or troglobites in rove beetles [Seevers 1957; Seevers 1965; Fresneda et al. 2011; Parker and Grimaldi 2014; Newton et al. 2016]). These suggest that the rise of the diversification rate and that of the within-clade disparification in the Palaeogene are not directly due to the cooling temperature but due to a variety of local adaptations in the specific habitats or niches opened during the Palaeogene cooling.

### Rapid radiations in low-energy conditions

Our study finds that the initial radiation of staphylinoid beetles and the steep falling of body size disparity both happened in the Jurassic, which implies that the typical low-energy conditions (low temperature, low O_2_, and high CO_2_) may promote among-clade diversification, thus prompting the Jurassic radiations of staphylinoid beetles. Additionally, the Cenozoic (particularly the recent 30 Ma), also a low-energy era (with low temperature and decreasing O_2_, though with low CO_2_) after the Cretaceous, coincided with increasing diversification rate and within-clade disparification. It is surprising that staphylinoid beetles would have a positive response of diversification to low-energy climatic conditions. On the contrary, in the high-energy Cretaceous (high temperature, high O_2_, and moderate but decreasing CO_2_), the net diversification rate of staphylinoid beetles went flat and even declined slightly, and the among-clade disparification of body size also regressed. This appears to contradict the prediction under the “ecological opportunity” hypothesis that both diversification and morphological disparification would intensify under high-energy conditions (Wright 1983; Evans et al. 2005; Erwin 2009; Grossnickle and Polly 2013), although more recent studies have found similar examples by different approaches, for example, the rapid body size evolution of birds and mammals in the cool Cenozoic (Clavel and Morlon 2017).

Our findings imply that there could be alternative connotations of “opportunity” for early staphylinoid beetles. McKenna et al. (2015a) hypothesized that forest litter would play important roles in diversiﬁcation of staphylinoid beetles. Examining the main habitats (litter and the associated habitats, e.g., dead wood, carcasses, dung, nests of social insects, etc.) of extant staphylinoid species, we agree with McKenna et al. (2015a) and suppose that these habitats provided “refuges” that would be unoccupied or newly occurred in the low-energy climate, for example, the niche vacancy caused by the Triassic–Jurassic or Cretaceous–Paleogene mass extinction and the slow recovery of biodiversity constrained by the low-energy climate in the Early and Middle Jurassic. We thus suppose that the “litter-refuge” hypothesis explains well the rapid radiations of staphylinoids in low-energy conditions (see our third expectation in Introduction).

## Data accessibility

- DNA sequences: GenBank accession numbers are available in Supplementary Appendix S1: Table S4.
- Species richness data: Numbers of species of Staphyliniformia (Supplementary Table S1) are provided by A. F. Newton in 2016. The full database is currently unpublished and will be eventually deposited at the website of the Field Museum of Natural History. An updated (November 2018) but simplified version of the database is available at the Catalogue of Life web site (http://www.catalogueoflife.org/col/, accessed 26 October 2019).
- Climate data: Temperature and CO_2_ data were provided by Dana L. Royer (Wesleyan University); O_2_ data were extracted from the Figure 3 in Berner (2009).
- Body size data: Those prepared for analyses are available in Supplementary Appendix S1: Table S4. The raw data that we collected are available upon request. The references of the body size data are given in Supplementary Appendix S4.

## Author contributions

L.L. and H-Z.Z. designed the study; L.L. and X.Z. performed the phylogenetic analyses; L.L. and C-Y.C. executed molecular dating; A.F.N. provided the data of species richness of involved taxa; A.F.N., M.K.T., C-Y.C., and L.L. contributed to the interpretation of phylogenetic results; L.L. gathered data and performed the rest analyses; L.L., C-Y.C., and X.Z. wrote the first draft of the manuscript, all authors contributed substantially to revisions.

## Supplementary Material

zoz053_Supplementary_Data
