# Supplementary material for: Linking evolutionary mode to palaeoclimate change reveals rapid radiations of staphylinoid beetles in low-energy conditions
Source: Curr Zool. 2019 Oct 22;66(4):435–44. doi: 10.1093/cz/zoz053 (PMC7319441; doi:10.1093/cz/zoz053)
Supplement: zoz053_Supplementary_Data [file zoz053_supplementary_data.zip › zoz053-Suppl_Data/Appendix_S4_ReferencesOfDataSources.pdf]

Appendix S4: References used for compiling body length dataset

Abarbanell, N. R. & J. S. Ashe (1989) Revision of the Species of *Pinophilus* Gravenhorst (Coleoptera, Staphylinidae) of America North of Mexico. *Fieldiana: Zoology (New Series)*, **54**, iv+32 pp.

Adachi, T. (1935) New staphylinid beetles from Japan (Studies on Staphylinidae of Japan I). *Kontyû*, **9**, 125-129.

Ádám, L. (1992) Two new Staphylinid species from Hungary (Coleoptera: Staphylinidae). *Folia Entomologica Hungarica*, **52**, 5-8.

Ádám, L. (1994) A new *Dinaraea* species from Hungary (Coleoptera: Staphylinidae). *Folia Entomologica Hungarica*, **55**, 21-22.

Aguilera, P., I. Ribera, C. Hernando (1998) Notes on the Palaearctic species of *Aulacochthebius*, with a description of *A. libertarius* sp. n. from the Moroccan Anti Atlas (Coleoptera, Hydraenidae). *European Journal of Entomology*, **95**, 629-637.

Ahn, K.-J. & J.S. Ashe. (1995) Revision of the intertidal aleocharine genus *Amblopusa* Casey and description of the new genus *Paramblopusa* (Coleoptera: Staphylinidae). *Journal of the New York Entomological Society*, **103**, 138-154.

Ahn, K.-J. (1996) A review of the *Diaulota* Casey (Coleoptera: Staphylinidae: Aleocharinae), with description of a new species and known larvae. *Coleopterists Bulletin*, **50**, 270-290.

Ahn, K.-J. (1997) A review of *Liparocephalus* Mäklin (Coleoptera: Staphylinidae: Aleocharinae) with description of larvae. *Pan-Pacijic Entomologist*, **73**, 79-92.

Ahn, K.-J. (2004) *Moorea zealandica*, new genus and species from New Zealand with a discussion of its phylogenetic relationships (Coleoptera: Staphylinidae: Aleocharinae). *New Zealand Journal of Zoology*, **31**, 255-261.

Ahn, K.-J., M.-J. Jeon & H.-J. Kim. (2000) New records of intertidal *Aleochara* Gravenhorst species and key to the species in Korea (Coleoptera: Staphylinidae: Aleocharinae). *Korean Journal of Entomology*, **30**, 243-247.

Anderson, R. S. & S. B. Peck (1985) *The Insects and Arachnids of Canada, part 13. The carrion beetles of Canada and Alaska (Coleoptera: Silphidae and Agyrtidae)*. Research Branch, Agriculture Canada, Ottawa, 121 pp.

Asenjo, A. & Ribeiro-Costa, C. S. (2010) *Pseudopsis monica* sp. nov. from the western Andes, Peru, with a key to the South American species of *Pseudopsis* Newman (Coleoptera, Staphylinidae, Pseudopsinae). *Revista Brasileira de Entomologia*, **54**, 229-234.

Ashe, J. S., and A. F. Newton (1993) Larvae of *Trichophya* and phylogeny of the tachyporine group of subfamilies (Coleoptera: Staphylinidae) with a review, new species and characterization of the Trichophyinae. *Systematic Entomology*, **18**, 267-286.

Assing, V. & A. Yu. Solodovnikov (1998) Three new species of *Othius* Stephens from the Caucasus (Coleoptera: Staphylinidae: Xantholininae). *Zoosystematica Rossica*, **7**, 299-305.

Assing, V. & M. Schülke (2000) A new microphthalmous species of *Lathrobium* GRAVENHORST from Greece (Insecta: Coleoptera: Staphylinidae: Paederinae). *Reichenbachia*, **33**, 321-326.

Assing, V. & M. Schülke (2007) Supplemente zur mitteleuropäischen Staphylinidenfauna (Coleoptera, Staphylinidae) III. *Entomologische Blätter für Biologie und Systematik der Käfer*, **102**, 1-78. [2007-3-27]

Assing, V. & P. Wunderle (1995) A revision of the Madeiran species of the genus *Othius* Stephens (Coleoptera: Staphylinidae). *Boletim do Museum Municipal do Funchal*, (1993)**45**, 53-65.

Assing, V. & P. Wunderle (1995) The fifth endemic *Stenus* from Madeira: *Stenus* (*Tesnus*) *ruivomontis* spec. nov. (Coleoptera, Staphylinidae). *Bocagiana*, **175**, 1-4.

Assing, V. & P. Wunderle (2001) On the Staphylinidae of Greece. II. New species and new records from central and northern Greece (Insecta: Coleoptera). *Linzer biologische Beiträge*, **33**, 103-136.

Assing, V. (1996) Revision der in den Süd- und Ostalpen endemischen Arten aus der Verwandtschaft des *Lathrobium testaceum* Kraatz, 1857 (Insecta: Coleoptera: Staphylinidae). *Annalen des Naturhistorischen Museums in Wien*, **98**, 425-434.

Assing, V. (1997) A revision of *Othius* Stephens, 1829. III. The species of the Western Palaearctic region exclusive of the Atlantic Islands (Coleoptera: Staphylinidae, Xantholininae). *Nova Supplementa Entomologica*, **10**, 3-130.

Assing, V. (1997) The species of *Othius* Stephens, 1832 of the Canary Islands (Coleoptera, Staphylinidae, Xantholininae). *Vieraea*, **251**, 103-115.

Assing, V. (1998) A new species of *Autalia* Leach in Samouelle from China (Insecta: Coleoptera: Staphylinidae: Aleocharinae). *Reichenbachia*, **32**, 209-212.

Assing, V. (1998) A revision of *Othius* Stephens - V. The species of the Himalayan region. *Beiträge zur Entomologie*, **48**, 293-342.

Assing, V. (1998) Two new Palaearctic species and two new synonyms of *Atrecus* Jacquelin du Val (Coleoptera: Staphylinidae, Xantholininae). *Beiträge zur Entomologie*, **48**, 343-351.

Assing, V. (1998) Zur Kenntnis der Staphylinidenfauna der Atlantischen Inseln: neue Arten, Synonyme und Nachweise (Col., Staphylinidae). *Entomologische Nachrichten und Berichte*, **42**, 139-146.

Assing, V. (1999) A revision of *Othius* Stephens (Coleoptera, Staphylinidae). VIII. Further records, new species, and a new synonym. *Linzer biologische Beiträge*, **31**, 661-691.

Assing, V. (1999) A revision of *Othius* Stephens, 1829. VII. The species of the Eastern Palaearctic region east of the Himalayas. *Beiträge zur Entomologie*, **49**, 3-96.

Assing, V. (1999) On Some *Autalia* Leach in Samouelle from Japan and Taiwan (Coleoptera, Staphylinidae, Aleocharinae). *The Japanese Journal of Systematic Entomology*, **5**, 163-165.

Assing, V. (2000) A new species of *Atrecus* Jacquelin du Val from China (Coleoptera: Staphylinidae, Staphylininae). *Linzer biologische Beiträge*, **32**, 75-78.

Assing, V. (2001) A revision of the microphthalmous *Lathrobium* Gravenhorst of Turkey, with descriptions of two new *Lathrobium* species from Italy and Albania (Insecta: Coleoptera: Staphylinidae). *Annalen des Naturhistorischen Museums in Wien*, **103**, 375-389.

Assing, V. (2002) A taxonomic and phylogenetic revision of *Amarochara* Thomson. I. The species of the Holarctic region (Coleoptera: Staphylinidae, Aleocharinae, Oxypodini). *Beiträge zur Entomologie*, **52**, 111-204.

Assing, V. (2002) On some micropterous species of Athetini from Nepal and China (Coleoptera: Staphylinidae, Aleocharinae). *Linzer biologische Beiträge*, **34**, 953-969.

Assing, V. (2002) Two new species of Athetini from Italy and Greece (Coleoptera: Staphylinidae, Aleocharinae). *Linzer biologische Beiträge*, **34**, 1021-1028.

Assing, V. (2003) A new species of *Atheta* Thomson from caves in southern Spain (Coleoptera: Stapbylinidae, Aleocharinae). *Linzer biologische Beiträge*, **35**, 543-546.

Assing, V. (2003) A new species of *Oligota* from Morocco, with redescription of *O. tugurtana* Fauvel and *O. pilicornis* Fauvel (Coleoptera: Staphylinidae, Aleocharinae). *Linzer biologische Beiträge*, **35**, 533-537.

Assing, V. (2003) A new species of the *Stenus glacialis* group from the northeastern Iberian Peninsula (Coleoptera: Staphylinidae, Steninae). *Linzer biologische Beiträge*, **35**, 711-716.

Assing, V. (2003) A new species of the *Stenus subcylindricus* group from northwestern Spain (Insecta: Coleoptera: Staphylinidae: Steninae). *Entomologische Abhandlungen*, **60**, 125-127.

Assing, V. (2003) A revision of Othiini. XIII. Horizontal and vertical distribution of *Othius*, new species, and additional records (Coleoptera: Staphylinidae: Staphylininae). *Entomological Problems*, **33**, 69-88.

Assing, V. (2003) A revision of *Othius* Stephens. IX. New taxa, new synonyms, additional records, and a checklist of species (Coleoptera, Staphylinidae, Staphylininae, Othiini). pp. 727-752. In: G. Cuccodoro & R. A. B. Leschen (eds.). Systematics of Coleoptera: Papers celebrating the retirement of Ivan Löbl. vol. 17, 2003, Gainsville: Memoirs on Entomology, International, v+955 pp.

Assing, V. (2003) A revision of the Western Palaearctic species of *Diochus* (Coleoptera: Staphylinidae: Staphylininae: Diochini). *Entomological Problems*, **33**, 111-117.

Assing, V. (2003) New species and records of Staphylinidae from Turkey (Coleoptera, Staphylinidae). *Entomologische Blätter für Biologie und Systematik der Käfer*, **98**, 153-177.

Assing, V. (2003) On the identity of *Trichophya huttoni* Wollaston (Coleoptera: Staphylinidae, Trichophyinae). *Linzer biologische Beiträge*, **35**, 515-518.

Assing, V. (2003) Review of Palaearctic *Autalia*. V. New species, additional records, and a key to species (Coleoptera: Staphylinidae, Aleocharinae). *Entomological Problems*, **33**, 45-50.

Assing, V. (2003) The genus *Phloeocharis* Mannerheim in the southern and western Iberian Peninsula (Coleoptera: Staphylinidae, Phloeocharinae). *Linzer biologische Beiträge*, **35**, 705-710.

Assing, V. (2003) The Turkish species of *Leptusa* Kraatz in the Schubert collection (Naturhistorisches Museum, Wien) (Coleoptera: Staphylinidae, Aleocharinae). *Koleopterologische Rundschau*, **73**, 75-82.

Assing, V. (2004) A new microphthalmous *Atheta* species from Yunnan, China (Coleoptera: Staphylinidae, Aleocharinae). *Linzer biologische Beiträge*, **36**, 589-592.

Assing, V. (2004) New species and records of Staphylinidae from Turkey III (Insecta: Coleoptera). *Linzer biologische Beiträge*, 36, 669-733.

Assing, V. (2004) On some species of *Leptusa* Kraatz, primarily from Spain (Coleoptera: Staphylinidae, Aleocharinae). *Linzer biologische Beiträge*, **36**, 61-75.

Assing, V. (2004) Two new species of *Cypha* Leach from Spain (Coleoptera: Staphylinidae, Aleocharinae). *Linzer biologische Beiträge*, **36**, 735-740.

Assing, V. (2004) Two new species, two new synonyms, and new records of *Leptusa* Kraatz (Coleoptera: Staphylinidae, Aleocharinae). *Linzer biologische Beiträge*, **36**, 643-653.

Assing, V. (2005) A revision of Othiini. XIV. New species, new synonyms, and new records (Insecta: Coleoptera: Staphylinidae). *Entomological Problems*, **35**, 51-67.

Assing, V. (2005) A revision of Othiini. XV. Three new *Othius* species from China and additional records (Insecta: Coleoptera: Staphylinidae). *Entomological Problems*, **35**, 137-146.

Assing, V. (2005) A revision of the Middle Asian species of *Drusilla* Leach (Coleoptera, Staphylinidae, Aleocharinae). *Entomologische Blätter*, **101**, 43-56.

Assing, V. (2005) A revision of Western Palaearctic *Medon*. IV. A new species and new records from Iran, and a catalogue of species (Coleoptera: Staphylinidae, Paederinae). *Linzer biologische Beiträge*, **37**, 1027-1034.

Assing, V. (2005) New species and records of Staphylinidae from Tunisia (Insecta: Coleoptera). *Linzer biologische Beiträge*, **37**, 749-770.

Assing, V. (2005) On the Taxonomy and biogeography of *Stenus* (*s. str.*) *erythrocnemus* Eppelsheim and related species (Insecta: Coleoptera: Staphylinidae). *Bonner zoologische Beiträge*, **53**, 303-310.

Assing, V. (2005) On the western Palaearctic species of *Drusilla* Leach, with special reference to the species of the eastern Mediterranean (Coleoptera: Staphylinidae, Aleocharinae). *Koleopterologische Rundschau*, **75**, 111-149.

Assing, V. (2005) Review of Palaearctic *Autalia* VI. A new species and a first record from China (Coleoptera: Staphylinidae, Aleocharinae). *Entomological Problems*, **35**, 147-150.

Assing, V. (2005) Two new species and new records of Staphylinidae from the Greek island Lesbos (Insecta: Coleoptera). *Linzer biologische Beiträge*, **37**, 1035-1046.

Assing, V. (2006) A revision of the Palaearctic species of *Orphnebius* Motschulsky (Insecta: Coleoptera: Staphylinidae: Aleocharinae). *Entomological Problems*, **36**, 1-26.

Assing, V. (2006) New species and records of Staphylinidae from Turkey IV, with six new synonymies (Coleoptera: Staphylinidae). *Koleopterologische Rundschau*, **76**, 223-276.

Assing, V. (2006) On the Italian species of *Drusilla* Leach, 1819. with a note on *D. taygetana* Assing (Coleoptera: Staphylinidae, Aleocharinae). *Beiträge zur Entomologie*, **56**, 281-296.

Assing, V. (2006) On the *Orphnebius* species of China (Insecta: Coleoptera: Staphylinidae: Aleocharinae). *Entomological Problems*, **36**, 75-84.

Assing, V. (2006) Six new species and additional records of *Leptusa* from northern Yunnan, China (Coleoptera: Staphylinidae, Aleocharinae). *Linzer biologische Beiträge*, **38**, 1157-1174.

Assing, V. (2006) Six new species of micropterous *Atheta* and *Apimela* from northern Yunnan, China (Coleoptera: Staphylinidae, Aleocharinae). *Linzer biologische Beiträge*, **36**, 589-592.

Assing, V. (2006) Three new species of Staphylinidae from Spain, with a new synonymy (Insecta: Coleoptera). *Linzer biologische Beiträge*, **38**, 1129-1137.

Assing, V. (2007) A new species of *Nudobius* from China (Coleoptera, Staphylinidae: Staphylininae, Xantholinini). *Zootaxa*, **1413**, 65-68.

Assing, V. (2007) A revision of *Amarochara* of the Holarctic region. III. A new species, a new synonym, and additional records (Coleoptera, Staphylinidae: Aleocharinae). *Zootaxa*, **1411**, 25-32.

Assing, V. (2007) A revision of the species of *Pronomaea* ERICHSON of the Western Palaearctic region, including Middle Asia - Coleoptera Staphylinidae Aleocharinae Pronomaeini. *Beiträge zur Entomologie*, **57**, 367-396.

Assing, V. (2007) A revision of Western Palaearctic *Medon*. V. A new species from Iran and additional records (Coleoptera: Staphylinidae, Paederinae). *Linzer biologische Beiträge*, **39**, 23-32.

Assing, V. (2007) A revision of Western Palaearctic *Medon*. VI. A new species from Turkey and additional records (Coleoptera: Staphylinidae: Paederinae). *Linzer biologische Beiträge*, **39**, 791-797.

Assing, V. (2007) New species and additional records of Paederinae and Aleocharinae from Iran (Coleoptera, Staphylinidae). *Deutsche Entomologische Zeitschrift*, **54**, 179-193.

Assing, V. (2007) New species and additional records of Staphylinidae from Turkey V (Coleoptera). *Stuttgarter Beiträge zur Naturkunde Serie A (Biologie)*, **700**, 1-64.

Assing, V. (2007) On the Aleocharini of Turkey, with notes on some species from adjacent regions (Coleoptera: Staphylinidae, Aleocharinae). *Beiträge zur Entomologie*, **57**, 177-209.

Assing, V. (2007) On the *Oxypoda* species of Turkey and adjacent regions. II. Three new species, additional records, and a checklist (Coleoptera: Staphylinidae, Aleocharinae). *Zootaxa*, **1411**, 1-24.

Assing, V. (2007) On the Xantholinini of Turkey and adjacent regions (Coleoptera: Staphylinidae: Staphylininae). *Zootaxa*, **1474**, 1-54.

Assing, V. (2007) *Proteinus crenulatus* - a complex of five species (Coleoptera: Staphylinidae: Proteininae). *Beiträge zur Entomologie*, **57**, 355-366.

Assing, V. (2007) Three new species, three new synonyms, and additional records of *Leptusa* from Turkey (Insecta: Coleoptera: Staphylinidae: Aleocharinae). *Entomological Problems*, **37**, 7-19.

Assing, V. (2007) Two new species and additional records of "small"-eyed *Quedius* from the Eastern Mediterranean (Coleoptera: Staphylinidae: Staphylininae). *Beiträge zur Entomologie*, **57**, 335-345.

Assing, V. (2008) A new species of *Cypha* Leach and the first record of *Bolitochara sogdiana* Gusarov from Kyrgyzstan (Coleoptera: Staphylinidae: Aleocharinae). *Linzer biologische Beiträge*, **40**, 231-234.

Assing, V. (2008) A review of the western Palaearctic and Middle Asian species of *Drusilla* Leach. IV. A new species from Iran and additional records (Coleoptera, Staphylinidae, Aleocharinae, Lomechusini). *Entomologische Blätter*, **103/104**, 51-58.

Assing, V. (2008) A revision of Othiini XVI. Four new species of *Othius* Stephens from the Himalaya and China, and additional records (Coleoptera: Staphylinidae, Staphylininae). *Koleopterologische Rundschau*, **78**, 245-263.

Assing, V. (2008) A revision of the Habrocerinae of the world. IV. A new species of *Habrocerus* from China and additional records (Coleoptera: Staphylinidae). *Beiträge zur Entomologie*, **58**, 135-144.

Assing, V. (2008) Nine new species and additional records of Staphylinidae from southern Spain, with new synonymies (Insecta: Coleoptera). *Linzer biologische Beiträge*, **40**, 1237-1294.

Assing, V. (2008) On the taxonomy and zoogeography of some Palaearctic Paederinae and Xantholinini (Coleoptera: Staphylinidae). *Linzer biologische Beiträge*, **40**, 1301-1325.

Assing, V. (2008) Review of Palaearctic *Autalia* VII. A new species from Yunnan, China (Coleoptera: Staphylinidae: Aleocharinae). *Linzer biologische Beiträge*, **40**, 247-250.

Assing, V. (2008) Seven new species and additional records of Palaearctic *Leptusa*, primarily from Yunnan, China. *Linzer biologische Beiträge*, **40**, 251-273.

Assing, V. (2009) A new species of *Metolinus* from Yunnan, China (Coleoptera: Staphylinidae: Staphylininae: Xantholinini). *Linzer biologische Beiträge*, **41**, 481-484.

Assing, V. (2009) A revision of Western Palaearctic *Medon*. VII. A new species from southern Turkey and additional records (Coleoptera: Staphylinidae: Paederinae). *Linzer biologische Beiträge*, **41**, 1253-1268.

Assing, V. (2009) New species and additional records of Homalotini, Athetini, Pygostenini, and Lomechusini from Yunnan, China (Coleoptera: Staphylinidae: Aleocharinae). *Linzer biologische Beiträge*, **41**, 485-507.

Assing, V. (2009) New species and additional records of Lomechusini from the Palaearctic region (Coleoptera: Staphylinidae: Aleocharinae). *Stuttgarter Beiträge zur Naturkunde A, Neue Serie*, **2**, 201-226.

Assing, V. (2009) On the *Leptusa* species of Turkey. VII. Notes on distribution, a new species, a new synonymy, and additional records (Coleoptera: Staphylinidae: Aleocharinae). *Linzer biologische Beiträge*, **41**, 427-436.

Assing, V. (2009) On the Staphylinidae of Turkey VI. Thirteen new species and additional records (Coleoptera: Staphylinidae). *Koleopterologische Rundschau*, **79**, 117-172.

Assing, V. (2009) Two new species and subspecies of *Leptusa* from Nepal and Montenegro (Coleoptera: Staphylinidae, Aleocharinae). *Linzer biologische Beiträge*, **41**, 453-458.

Assing, V. (2010) A new micropterous species of *Deropoda*, subgenus of *Oxypoda*, from Italy (Coleoptera: Staphylinidae: Aleocharinae: Oxypodini). *Linzer biologische Beiträge*, **42**, 1177-1181.

Assing, V. (2010) A revision of *Amarochara* of the Holarctic region. IV. Three new species from China, a new synonymy, additional records, and an updated key to species (Coleoptera: Staphylinidae: Aleocharinae: Oxypodini). *Linzer biologische Beiträge*, **42**, 1139-1154.

Assing, V. (2010) A revision of Othiini. XVII. A new species from China and additional records (Coleoptera: Staphylinidae: Staphylininae). *Linzer biologische Beiträge*, **42**, 1077-1091.

Assing, V. (2010) A revision of Palaearctic *Medon*. VIII. A new species from Nepal and additional records (Coleoptera: Staphylinidae: Paederinae). *Linzer biologische Beiträge*, **42**, 489-498.

Assing, V. (2010) Four new species and additional records of Staphylinidae from Spain, primarily from the south (Insecta: Coleoptera). *Linzer biologische Beiträge*, **42**, 1105-1124.

Assing, V. (2010) On the *Cypha* fauna of China, with remarks on some species described from other regions (Coleoptera: Staphylinidae: Aleocharinae: Hypocyphtini). *Linzer biologische Beiträge*, **42**, 1169-1176.

Assing, V. (2010) Two new species and additional records of Lomechusini from the Palaearctic region (Coleoptera: Staphylinidae: Aleocharinae). *Linzer biologische Beiträge*, **42**, 1093-1104.

Assing, V. (2011) On the Staphylinidae (Coleoptera) of Iran. II. New species and additional records, with special reference to the Paederinae, Xantholinini, and Aleocharinae. *Stuttgarter Beiträge zur Naturkunde A, Neue Serie*, **4**, 137-183.

Assing, V. (2011) Six new species and additional records of Aleocharinae from China (Coleoptera: Staphylinidae: Aleocharinae). *Linzer biologische Beiträge*, **43**, 291-310.

Assing, V. (2014) A revision of *Pronomaea* ERICHSON. II. A new species from China and additional records (Coleoptera: Staphylinidae: Aleocharinae). *Linzer biologische Beiträge*, **46**, 1211-1216.

Assing, V. (2014) A revision of *Tetartopeus* IV. A new species from Turkey, new synonymies, and additional records (Coleoptera: Staphylinidae: Paederinae). *Linzer biologische Beiträge*, 46, 1119-1131.

Assing, V. (2014) On the genus *Crataraea* (Coleoptera: Staphylinidae: Aleocharinae: Oxypodini). *Linzer biologische Beiträge*, **46**, 1111-1118.

Assing, V. (2014) On the *Oedichirus* fauna of China (Coleoptera: Staphylinidae: Paederinae). *Linzer biologische Beiträge*, **46**, 1229-1240.

Assing, V. (2014) On the Staphylinidae of Turkey X. Two new species and additional records (Insecta: Coleoptera). *Linzer biologische Beiträge*, **46**, 1133-1146.

Assing, V. (2014) Three new species, redescriptions, a new synonymy, and additional records of *Cypha* from the West Palaearctic region (Coleoptera: Staphylinidae: Aleocharinae). *Linzer biologische Beiträge*, **46**, 1167-1178.

Assing, V., and P. Wunderle (1995) A revision of the species of the subfamily Habrocerinae (Coleoptera: Staphylinidae) of the world. *Revue suisse de Zoologie*, **102**, 307-359.

Assing, V., and P. Wunderle (1996) A revision of the species of the subfamily Habrocerinae of the world. Supplement I. (Coleoptera: Staphylinidae). *Beiträge zur Entomologie*, **46**, 373-378.

Assing, V., J. Frisch, M. Kahlen, I. Löbl, G. H. Lohse, V. Puthz, M. Schülke, H. Terlutter, M. Uhlig, J. Vogel, P. Wunderle, & L. Zerche (1998) 23. Familie: Staphylinidae. pp. 119-197. In W. Lucht and B. Klausnitzer, Die Käfer Mitteleuropas. Band 15. 4 Supplementband. 398 pp. Krefeld: Goecke & Evers.

Aubé, C. (1844) Révision de la famille des Psélaphiens. *Annales de la Société Entomologique de France*, (2)2: 73-160.

Baranowski, R. (1980) Taxonomic notes on *Atheta kaiseriana* Brundin (Coleoptera: Staphylinidae), with lectotype designation. *Insect Systematics & Evolution*, **11**, 277-280.

Bekchiev, R., and P. Hlaváč (2008) A new species of the genus *Dicentrius* Reitter, 1882 (Coleoptera: Staphylinidae, Pselaphinae) from western Serbia. In D. Pavićević and M. Perreau (editors), Advances in the studies of the fauna of the Balkan Peninsula - Papers dedicated to the memory of Guido Nonveiller. 241-243. Belgrade: Institute for Nature Conservation of Serbia, 564 pp.

Benick, G. (1980) Weitere neue Atheten aus Sowjet-Russland (Col. Staphyl.). *Nouvelle Revue d'Entomologie*, 10, 335-340.

Benick, G. (1981) *Atheta* (Parameotica) difficilis Bris. (rigua Williams) ist eine gute Art. Neubeschreibung von zwei nächstverwandten Arten. *Revue suisse de Zoologie*, **88**, 561-565.

Benick, G. (1989) Neue Arten der Gattungen *Atheta* Thoms. und *Gnypeta* Thoms. aus der Mongolischen Volksrepublik (Coleoptera, Staphylinidae). *Deutsche Entomologische Zeitschrift, (N. F.)*, **36**, 299-307.

Benick, G. von (1967) Die palaearktischen Arten der Gattung *Amischa* C. G. Thomson (Col. Staph.). *Entomologische Blätter, Krefeld*, **63**, 16-29.

Benick, L. (1925) Bemerkungen zum Catalogus Coleopterorum regionis palaearcticae, Unterfamilie Steninae. *Koleopterologische Rundschau*, **11**, 71-76.

Bernhauer, M. (1898) Neue Staphyliniden aus Europa. *Verhandlungen der Zoologisch-Botanischen Gesellschaft in Österreich*, **48**, 338-341

Bernhauer, M. (1904) Neue exotische Staphyliniden. *Stettiner Entomologische Zeitung*, **65**, 217-242.

Bernhauer, M. (1906) Neue Staphyliniden aus Afrika. *Deutsche Entomologische Zeitschrift*, **1906**, 185-192.

Bernhauer, M. (1907) Zur Staphylinidenfauna von Japan. *Verhandlungen der k. k. zoologisch-botanischen Gesellschaft in Wien*, **57**, 371-414.

Bernhauer, M. (1908) Beitrag zur Staphylinidenfauna von Südamerika. *Archiv für Naturgeschichte*, **74**, 283-372.

Bernhauer, M. (1908) Neue Staphylinidae aus dem subtropischen und tropischen Afrika. In: Schultze, L. (1908). Zoologische und anthropologische Ergebnisse einer Forschungsreise im westlichen und zentralen Südafrika ausgeführt in den Jahren 1903-1905. Erster Band: Systematik und Tiergeographie. *Denkschriften der Medizinisch-Naturwissenschaftlichen Gesellschaft zu Jena*, 13: pp. 101-118

Bernhauer, M. (1908) Neue Staphylinidae aus dem subtropischen und tropischen Afrika. In L. Schultze, Zoologische und Anthropologische Ergebnisse einer Forschungsreise im westlichen und zentralen Südafrica . . . . 1. Systematik und Tiergeographie. *Denkschriften der medicinische-naturwissenschaftlichen Gesellschaft zu Jena*, **13**, 101-118.

Bernhauer, M. (1910) Beitrag zur Kenntnis der Staphyliniden-Fauna von Zentralamerika. *Verhandlungen der k. k. zoologisch-botanischen Gesellschaft in Wien*, **60**, 350-393.

Bernhauer, M. (1911) Zur Staphylinidenfauna von Süd-Amerika (Col.). *Deutsche Entomologische Zeitschrift*, **1911**, 403-422.

Bernhauer, M. (1912) Beitrag zur Staphylinidenfauna von Afrika. *Entomologische Mitteilungen*, **1**, 177-183, 203-209.

Bernhauer, M. (1912) Zur Staphylinidenfauna von Südamerika. *Entomologische Blätter*, **8**, 167-179.

Bernhauer, M. (1915) Neue Staphyliniden des tropischen Afrika. *Verhandlungen der k. k. zoologisch-botanischen Gesellschaft in Wien*, **65**, 287-321.

Bernhauer, M. (1915) Staphyliniden. In Ergebnisse der Zweiten Deutschen Zentral-Afrika-Expedition 1910-1911 unter Führung Adolf Friedrichs, Herzog zu Mecklenburg. I. Zoologie, 361-368. Leipzig: Klinkhardt & Biermann.

Bernhauer, M. (1915) Zur Staphylinidenfauna der Philippinen: VI. Beitrag zur Kenntnis der indo-malayischen Fauna. *The Philippine Journal of Science*, **10**, 117-129.

Bernhauer, M. (1915) Zur Staphyliniden-fauna des tropischen Afrika. Annales Musei Nationalis Hungarici 13, 95-189.

Bernhauer, M. (1917) Ein neuer Phucobius aus China. *Wiener Entomologische Zeitung*, **36**, 125.

Bernhauer, M. (1917) Neue südamerikanische Staphyliniden. *Wiener Entomologische Zeitung*, **36**, 102-116.

Bernhauer, M. (1922) No. II. Coleoptera, Staphylinidae. In Reports of the Percy Sladen Trust Expedition to the Indian Ocean in 1905, under the leadership of Mr. J. Stanley Gardiner, M. A., 7. *The Transactions of the Linnean Society of Londo, Zoology*, (2)**18**, 165-186.

Bernhauer, M. (1922) Sauter's Formosa-Ausbeute: Staphylinidae. *Archiv für Naturgeschichte*, (A)**88**, 220-237.

Bernhauer, M. (1926) Neue Staphyliniden aus Ostindien. *Wiener Entomologische Zeitung*, **43**, 19-25.

Bernhauer, M. (1928) Dr. E. Mjöbergs Zoological Collections from Sumatra. 8. Staphylinidae. *Arkiv för Zoologi*, (1927) (A)**19**, 1-28.

Bernhauer, M. (1931) Zur Staphylinidenfauna des chinesischen Reiches. *Wiener Entomologische Zeitung*, **48**, 125-132.

Bernhauer, M. (1932) Neuheiten der paläarktischen Staphylinidenfauna. *Koleopterologische Rundschau*, **17**, 232-245.

Bernhauer, M. (1933) Neues aus der Staphylinidenfauna China's. Entomologisches Nachrichtenblatt. *Troppau*, **7**, 39-54.

Bernhauer, M. (1934) Neuheiten der javanischen Staphylinidenfauna. *Atti del Museo Civico di Storia Naturale, Trieste*, **12**, 171-175.

Bernhauer, M. (1936) Neue Staphyliniden vom belgischen Kongo. *Revue de Zoologie et de Botanique Africaines*, **29**, 21-28.

Bernhauer, M. (1943) Neue Staphyliniden der paläarktischen Fauna. *Koleopterologische Rundschau*, **29**, 71-76.

Bernhauer, M. 1932b. Neue Kurzflügler aus dem belgischen Kongostaate. *Revue de Zoologie et de Botanique Africaines*, **22**, 140-174.

Besuchet, C. & E. Sundt (1971) Familie: Ptiliidae. In: K. W. Harde & G. A. Lohse (eds.): Die Käfer Mitteleuropas. Vol. 3. Krefeld: Goecke & Evers. pp. 311-342.

Besuchet, C. & S. Kurbatov (2007) Les *Bryaxis* du Caucase et du secteur oriental des chaÎnes Pontiques (Coleoptera: Staphylinidae: Pselaphinae). *Russian Entomological Journal*, **16**, 155-206.

Besuchet, C. (1964) Psélaphides paléarctiques Espèces nouvelles et notes synonymiques. II (Coleoptera). *Revue suisse de Zoologie*, **74**, 411-443.

Besuchet, C. (1980) *Neuraphes* et Scydmoraphes de la Suisse, de l'ain et de la Haute-Savoie (Coleoptera Scydmaenidae). *Mitteilungen der Entomologischen Gesellschaft Basel*, **30**, 189-196.

Besuchet, C. (1980) Revision des *Leptinus* paléarctiques (Coleoptera: Leptinidae). *Revue suisse de Zoologie*, **87**, 131-142.

Besuchet, C. (1983) *Bryaxis* nouveaux ou méconnus du Nord de l'Ita1ie (Coleoptera: Pselaphidae). *Revue suisse de Zoologie*, **90**, 769-780.

Besuchet, C. (1999) Le genre *Dicentrius* Reitter (Coleoptera Staphylinidae Pselaphinae). Mitteilungen der *Schweizerischen Entomologischen Gesellschaft*, **72**, 221-233.

Besuchet, C. (2002) *Bryaxis* nouveaux pour la France et l'Italie (Coleoptera, Staphylinidae, Pselaphinae). Mitteilungen der *Schweizerischen Entomologischen Gesellschaft*, **74**, 211-216.

Bierig, A. (1933) Especies nuevas o poco conocidas de *Neobisnius* (Col. Staphylinidae) de la region neotrópica. *Revista de Entomologia*, **3**, 48-57.

Bierig, A. (1934) Neues aus der Staphyliniden-Gattung *Cafius* (Col.), nebst Beschreibung neuer Arten aus Kuba und Nordamerika. *Revista de Entomologia*, **4**, 65-70.

Bilton, D. T. (2014) New species and new records of *Pterosthetops*: eumadicolous water beetles of the South African Cape (Coleoptera, Hydraenidae). *Zootaxa*, **3811**, 438-462.

Blackburn, T. (1888) Notes on Australian Coleoptera, with descriptions of new species. *Transactions and Proceedings and Report of the Royal Society of South Australia*, **10**, 12-30.

Blas, M. & P. A. V. Borges (1999) A new species of *Catops* Paykull, 1798 (Coleoptera: Cholevidae) from the Azores with remarks on the Macaronesian fauna. *Elytron*, **13**, 173-184.

Blas, M. (1980) El genéro *Choleva* Latreille (1796) en la Penisula Iberica (Col. Catopidae). *Mémoires de Biospéologie*, **7**, 127-139.

Boháč J. (1993) *Algon viridis* sp. n. from Vietnam (Coleoptera, Staphylinidae). *Acta Universitatis Carolinae Biologica*, **36**, 445-448.

Boheman, C. H. (1848) Insecta Caffrariae annis 1838-1845 a J. A. Wahlberg collecta. Coleoptera. (Carabici, Hydrocanthari, Gyrinii et Staphylinii), 1(1): viii + 297. Holmiae: Norstedtiana.

Boheman, C. H. (1858) Coleoptera. Species novas descripsit. pp. 1-112, 1 pl. In C. Virgin, Kongliga Svenska fregatten Eugenies Resa Omkring Jorden. Zoologi 1. Insecta. Stockholm: P. A. Norstedt & Söner.

Bondroit, J. (1913) Descriptions de Staphylinides nouveaux. *Annales de la Société Entomologique de Belgique*, **57**, 90-93.

Bordoni, A. & G. Castellini (1973) Sulle specie Paleartique del genere Mastigus Latreille, con osservazioni su due specie dell'Africa Australe (Coleoptera Scydmaenidae). *Redia*, **54**, 295-323.

Bordoni, A. (1971) Note sugli *Xantholinus* della Turchia e descrizione di una nuova specie (Col. Staphylinidae). *Redia*, **52**, 679-689.

Bordoni, A. (1974) Contributo alla conoscenza sistematica e faunistica degli *Staphylinidae* Italiani - III - specie Italiane del genere *Lithocharis* Boisd. & Lac. (Col. Staphylinidae). *Estratto da Redia*, **55**, 321-329.

Bordoni, A. (1982) Coleoptera. Staphylinidae. Generalità - Xantholininae. In Fauna d'Italia 19: i-xii + 1-434. Bologna: Calderini.

Bordoni, A. (1983) Studi sulla sistematica degli *Xantholinus* Berthold 1827, XIII. Una nuova specie dell'Iran (Insecta: Coleoptera: Staphylinidae). *Senckenbergiana Biologica*, **63**, 61-63.

Bordoni, A. (1983) Una nuova *Choleva* della Calabria (Coleoptera, Catopidae). *Fragmenta entomologica, Roma*, **17**, 101-104.

Bordoni, A. (1984) Note su alcuni stafilinidi del Libano (Coleoptera). *Fragmenta Entomologica*, **17**, 331-345.

Bordoni, A. (1994) Nuove specie di *Acylophorus* Nordmann della Sierra Leone. *Problemi Attuali di Scienza e di Cultura*, **267**, 307-314.

Bordoni, A. (1997) A revision of the tribe Xantholinini from Japan, I (Coleoptera, Staphylinidae). *Japanese Journal of Systematic Entomology*, **3**, 167-179.

Bordoni, A. (2000) Contribution to the knowledge of the Xantholinini from China. I (Coleoptera, Staphylinidae). Mitteilungun aus dem Museum für Naturkunde in Berlin, *Zoologische Reihe*, **76**, 121-133.

Bordoni, A. (2002) Xantholinini della Regione Orientale (Coleoptera: Staphylinidae). Classificazione, filogenesi e revisione tassonomica. *Monografie di Museo Regionale di Scienze Naturali*, *Torino*, **33**, 1-998.

Bordoni, A. (2003) Contributo alla conoscenza degli Xantholinini della Cina. IV. Un nuovo genere e nuove specie raccolti da Michael Schülke nello Shaanxi e nel Sichuan (Coleoptera, Staphylinidae). *Beiträge zur Entomologie*, **53**, 253-275.

Bordoni, A. (2003) Nuovi dati per la conoscenza degli Xantholinini della regione orientale. III. Nuove specie del Naturhistorisches Museum di Vienna. *Quaderno di Studi e Notizie di Storia Naturale della Romagna*, **17**(suppl.), 43-54.

Bordoni, A. (2003) Nuovi dati per la conoscenza degli Xantholinini della regione orientale. IV. Nuove especie della collezione Tateo Ito di Kyoto (Coleoptera: Staphylinidae) - 141° contributo alla conoscenza degli Staphylinidae. Animma, **10**, 1-14.

Bordoni, A. (2003) Una nuova specie di *Gyrohypnus* dell'Asia Minore (Coleoptera Staphylinidae). *Bollettino della Societa Entomologica Italiana*, **134**, 229-232.

Bordoni, A. (2004) New data on the Xantholinini of the Oriental Region. VIII. Species from Mount Kinabalu (Sabah) collected by Ales Smetana (Coleoptera Staphylinidae) - 156° contribution to knowledge of the Staphylinidae. *Redia*, **87**, 27-38.

Bordoni, A. (2005) Contributo alla conoscenza degli Xantholinini della Cina. VII. Nuove specie di Gangsu, Sichuan e Shaanxi (Coleoptera, Staphylinidae). Atti del Museo di Storia naturale della Maremma, **21**, 25-31.

Bordoni, A. (2005) Contributo alla conoscenza degli Xantholinini della Regione Orientale. XI. *Thyreocephalus* punctifasciatus sp. n. della Tailandia (Coleoptera, Staphylinidae). *Onychium*, **3**, 11-13.

Bordoni, A. (2005) Descrizione del Maschio di *Choleva* (*s. str.*) *leucophthalma* della Maiella (Coleoptera, Cholevidae). *Fragmenta entomologica, Roma*, **37**, 47-56.

Bordoni, A. (2005) Revision of the Xantholinini of Australia (Coleoptera: Staphylinidae) - 152° contribution to the knowledge of the Staphylinidae. In: Daccordi, M. & Giachino, P. M. (eds.) Results of the Zoological Missions to Australia of the Regional Museum of Natural Sciences of Turin, Italy - II. *Monografie di Museo Regionale di Scienze Naturali, Torino*, **42**, 435-614.

Bordoni, A. (2005) *Stenus* (*Hemistenus*) *nocturnus* sp. n. del Monte Baldo, Italia (Coleoptera Staphylinidae) - 145° contributo alla conoscenza degli Staphylinidae. *Monografie di Museo Regionale di Scienze Naturali, Torino*, **22**, 319-328.

Bordoni, A. (2006) Nuovi dati per la conoscenza degli Xantholinini della Regione Orientale. XII. Specie della collezione Jiri Janak (Coleoptera Staphylinidae). *Bollettino della Società Entomologica Italiana*, **138**, 197-206.

Bordoni, A. (2006) Nuovi dati per la conoscenza degli Xantholinini della Regione Orientale. XIII. Specie de]1'India meridionale raccolte da H. Franz e conservate nel Naturhistorisches Museum di Vienna (Coleoptera, Staphylinidae) - 168° contributo alla conoscenza degli Staphylinidae. *Entomologica-Bari*, **39**, 89-97.

Bordoni, A. (2007) Palaearctic Xantholinini of the Museum of Zoology in Lund, Sweden, with description of two new species (Coleoptera, Staphylinidae). 182° contribution to the knowledge of the Staphylinidae. *Zootaxa*, **1624**, 59-68.

Bordoni, A. (2009) Contribution to the knowledge of the Xantholinini from China. XIV. *Nudobius linanensis* n. sp. from Zhejiang, notes and new records on some interesting species (Coleoptera, Staphylinidae). *Quaderno di Studi e Notizie di Storia Naturale della Romagna*, **28**, 107-111.

Bordoni, A. (2009) New data on the Xantholinini of the Oriental Region. 18. Specimens from Nepal of the Naturkundemuseum Erfurt (Insecta: Coleoptera: Staphylinidae). In: Hartmann, M. & Weipert, J. (eds.). Biodiversität und Naturausstattung im Himalaya III. - Verein der Freunde und Förderer des Naturkundemuseums Erfurt e.V., Erfurt, pp. 243-247.

Bordoni, A. (2009) Notes on the Palaearctic Xantholinini. V. *Megalinus christophi* (Lokay) (Coleptera Staphylinidae) - 206° contribution to the knowledge of the Xantholinini. *Redia*, **92**, 23-24.

Bordoni, A. (2009) Xantholinini della Cina, della regione orientale e dell'Australia. nuove specie e nuovi dati geonemici (Coleoptera, Staphylinidae). *Fragmenta entomologica, Roma*, **41**, 87-111.

Bordoni, A. (2010) *Allolinus* Coiffait, 1966 nuovo sinonimo di *Leptacinus* Erichson, 1839 e descrizione di *Leptacinus yemeniticus* n. sp. dello Yemen (Coleoptera Staphylinidae). *Bollettino della Societa Entamologica Italiana*, **142**, 77-79.

Bordoni, A. (2010) Male genitalia of palaearctic species of the genus *Nudobius* (Coleoptera Staphylinidae). *Bollettino della Societa Entamologica Italiana*, **142**, 19-26.

Bordoni, A. (2010) New data on the Xantholinini from the Oriental Region. XXII. New species from Laos (Coleoptera, Staphylinidae) - 203° contribution to the knowledge of the Staphylinidae. *Linzer biologische Beiträge*, **42**, 523-528.

Bordoni, A. (2010) Revisione degli Xantholinini della Nuova Guinea e delle isole austromalesi (Coleoptera: Staphylinidae) - 165° contributo alla conoscenza degli Staphylinidae. *Bollettino del Museo Regionale di Scienze Naturali*, **27**, 253-635.

Bordoni, A. (2010) Xantholinini from the Australian and Oriental Regions. New genus, new species and new records (Coleoptera, Staphylinidae) - 208° contribution to the knowledge of the Staphylinidae. *Zootaxa*, **2538**, 38-46.

Bordoni, A. (2014) Contribution on the Xantholinini from China. XVI. *Megalinus neolizipingensis* sp. n. from Yunnan and Sichuan and new combinations (Coleoptera Staphylinidae) - 204th contribution to the knowledge of the Staphylinidae. *Redia*, **97**, 49-50.

Bordoni, A. (2014) Contribution to the Knowledge of the Xantholinini from Japan, III - *Megalinus amamiko* sp. n., a very interesting taxon from Amami-Oshima Islands (Ryukyu, Japan) and New Records (Coleoptera: Staphylinidae) - 249° Contribution to the Knowledge of the Staphylinidae. *Japanese Journal of Systematic Entomology*, **20**, 269-272.

Bordoni, A. (2015) Revision of the Oriental *Platyprosopus* (Coleoptera Staphylinidae Platyprosopini) - 255th Contribution to the knowledge of the Staphylinidae. *Redia*, **98**, 57-75.

Breit, J. (1904) Zwei neue Käferarten aus dem mitteleuropäischen Faunengebiete. *Münchener Koleopterologische Zeitschrift*, **2**, 28-29.

Broun, T. (1880-1881) Manual of the New Zealand Coleoptera, I-II. Wellington, xx+744+iii pp.

Broun, T. (1886) Manual of the New Zealand Coleoptera. Parts III. and IV. *Colon*ial Museum and Geological Survey Department, Wellington, xvii + pp. 745-973.

Broun, T. (1921) Descriptions of new genera and species of Coleoptera. *Bulletin of the New Zealand Institute*, **1**, 475-590.

Brunke, A. & A. Y. Solodovnikov. (2014) Male secondary sexual characters resolve taxonomic uncertainty: five new species and a review of the formerly monotypic rove beetle genus *Mimosticus* Sharp (Coleoptera: Staphylinidae: Staphylininae). *Zootaxa*, **3893**, 56-76.

Cai, Y.-P., Z.-Y. Zhao & H.-Z. Zhou. (2015) Three new species of the genus *Quedius* (subgenus *Microsaurus*) from China (Coleoptera: Staphylinidae: Staphylinini: Quediina). *Zootaxa*, **3973**, 567-578.

Cameron, M. (1918) New species of Staphylinidae from Singapore. Part I. *Transactions of the Entomological Society of London*, **1918**, 58-90.

Cameron, M. (1925) New oriental Staphylinidae (Piestini). *Entomologist's Monthly Magazine*, **61**, 230-243.

Cameron, M. (1926) New species of Staphylinidae from India. Part III. *Transactions of the Entomological Society of London*, **1926**, 171-191.

Cameron, M. (1930) New Staphylinidae from the Malay Peninsula. *Journal of the Federated Malay States Museums*, **16**, 154-159.

Cameron, M. (1930) The fauna of British India including Ceylon and Burma. Coleoptera. Staphylinidae. Vol. I, Taylor and Francis, London.

Cameron, M. (1932) The fauna of British India including Ceylon and Burma. Coleoptera. Staphylinidae. Vol. 3: xiii + 1-443. London: Taylor and Francis.

Cameron, M. (1933) New species of Staphylinidae (col.) from the Belgian Congo. Bulletin et *Annales de la Société Entomologique de Belgique*, **73**, 35-53.

Cameron, M. (1934) The staphylinid Coleoptera of the New Hebrides. *Stylops*, **3**, 20-24.

Cameron, M. (1939) Fauna of British India, including Ceylon and Birma. Coleoptera. Staphylinidae-Vol. IV. Part II. London: Taylor Francis, 691pp.

Cameron, M. (1939) The fauna of British India including Ceylon and Burma. Coleoptera. Staphylinidae. Vol. IV, Part I. London: Taylor and Francis, xviii+410 pp.

Cameron, M. (1941) Descriptions of new Staphylinidae (Coleopt.). *The Proceedings of the Royal Entomological Society of London (B)*, **10**, 56-60, 142-147.

Cameron, M. (1944) Descriptions of new Staphylinidae (Coleoptera). *The Proceedings of the Royal Entomological Society of London (B)*, **13**, 11-15, 49-52.

Cameron, M. (1945) Some observations on the Staphylinidae of the Broun Collection of Coleoptera in the British Museum, with descriptions of new genera and species. *The Annals and Magazine of Natural History*, (11)**11**, 779-793.

Cameron, M. (1950) New species of Staphylinidae (Col.) from the Malay Peninsula. *The Annals and Magazine of Natural History*, (12)**3**, 1-40, 89-131.

Cameron, M. (1951) New species of African Staphylinidae. Part II. *Journal of the East Africa Natural History Society*, **19**, 398-407.

Cameron, M. (1952) Results of the Archibold Expeditions. New species of Staphylinidae (Col.) from New Guinea and Misool. *Treubia*, **21**, 241-256.

Campbell, J. M. (1974) A new species of *Micropeplus* (Coleoptera: Micropeplidae) from Oregon. *The Canadian Entomologist*, **106**, 465-466.

Campbell, J. M. (1982) A revision of the genus *Lordithon* Thomson of North and Central America (Coleoptera: Staphylinidae). *Memoirs of the Entomological Society of Canada*, **119**, 1-116.

Campbell, J. M. (1983) A revision of the North American Omaliinae (Coleoptera: Staphylinidae). The genus *Olophrum* Erichson. *The Canadian Entomologist*, **115**, 577-622.

Campbell, J. M. (1989) *Micropeplus nelsoni*, a new species from the Cascade Range of Washington (Coleoptera: Micropeplidae). *Coleopterists Bulletin*, **43**, 305-310.

Campbell, J. M. (1990) A new species of *Oxyporus* (Coleoptera: Staphylinidae) and rediscovery of *O. flohri* from Guatemala. *Coleopterists Bulletin*, **44**, 211-215.

Caron, E., C. S. Ribeiro-Costa, and A. F. Newton (2008) New position of an abdominal defensive gland complex in Staphylinidae (Coleoptera) with redescription of *Piestus* heterocephalus Fauvel, 1902 (Piestinae). *Zootaxa*, **1895**, 1-9.

Caron, E., C. S. Ribeiro-Costa, and A. F. Newton (2012) Cladistic analysis and revision of *Piestus* Gravenhorst with remarks on related genera (Coleoptera: Staphylinidae: Piestinae). *Invertebrate Systematics*, **25**, 490-585.

Caron, E., J. C. De Castro, M. R. D. Silva & C. S. Ribeiro-Costa. (2016) Phylogeny and revision of a colorful Neotropical genus of rove beetles: *Xenopygus* Bernhauer (Coleoptera: Staphylinidae). *Zootaxa*, **4138**, 59-82.

Casey, T. L. (1886) Revision of the Californian species of *Lithocharis* and allied genera. *Bulletin of the California Academy of Sciences*, **5**, 1-40.

Casey, T. L. (1893) Coleopterological notices. V. *Annals of the New York Academy of Sciences*, **7**, 281-606+Plate I.

Casey, T. L. (1906) Observations on the staphylinid groups Aleocharinae and Xantholinini chiefly of America. *Transactions of the Academy of Science of St. Louis*, **16**, 125-434.

Casey, T. L. (1910) Memoirs on the Coleoptera. vol. 1. Lancaster, Pennsylvania, USA: New Era Printing Company, 205 pp.

Casey, T. L. (1911) Memoirs on the Coleoptera. vol. 2. Lancaster, Pennsylvania, USA: New Era Printing Company, 259 pp.

Casey, T. L. (1911) New American species of Aleocharinae and Myllaeninae. In: Memoirs on the Coleoptera. II. The New Era Printing Co., Lancaster, Pennsylvannia, 245 pp.

Castellini, G. (1986) A proposito di *Bryaxis paganettii* (Blattn'y, 1914) (Coleoptera, Pselaphidae). *Atti del Museo Civico di Storia Naturale (Grosseto)*, **9**, 103-106.

Castellini, G. (1990) Quattro nuovi *Euconnus* di sierra leone (Coleoptera, Scydmaenidae). *Problemi Attuali di Scienza e di Cultura*, **(265)**, 185-189.

Castro, A., A.M. García & M. Ferreras (2000) *Hydraena* (*Haenydra*) *madronensis* sp.nov. from the Iberian Peninsula (Coleoptera: Hydraenidae). *Entomological Problems,* **31**, 61-64.

Champion, G. C. (1918) Notes on various South American Coleoptera collected by Charles Darwin during the voyage of the "Beagle", with descriptions of new genera and species. *Entomologist's Monthly Magazine,* **54**, 43-55.

Chatzimanolis, S. (2004) A revision of the Neotropical beetle genus *Nordus* Blackwelder (Insecta: Coleoptera: Staphylinidae: Xanthopygina). *Entomologische Abhandlungen*, **62**, 3-64.

Chatzimanolis, S. (2016) A revision of the myrmecophilous genus *Smilax* Laporte (Coleoptera: Staphylinidae: Staphylininae). *Zootaxa*, **4162**, 283-303.

Cho, Y. B. (1996) Studies on the tribe Philonthini (Coleoptera: Staphylinidae) from Korea I. Genera *Neobisnius* and *Erichsonius*. *The Korean Journal of Zoology*, **39**, 159-163.

Coiffait, H. & F. Saiz (1968) Les Staphylinidae (*sensu lato*) du Chile. In C. Deboutteville and E. Rapoport (eds.), Biologie de Amérique Australe. Études sur la faune du sol. 4: 339-468. Paris: Centre National de la Recherche Scientifique.

Coiffait, H. (1956) Les "*Staphylinus*" et genres voisins de France et des régions voisines. *Mémoires du Muséum National d'Histoire Naturelle. Zoologie (A)*, **8**, 177-224.

Coiffait, H. (1956) Les Xantholinitae de France et des régions voisines, (Col. Staphylinidae). *Revue Française d'Entomologie*, **23**, 31-75.

Coiffait, H. (1957) Les *Phloeocharis anophtalmes* (subg. *Scotodytes* Saulcy) (Coleoptera Staphylinidae). *Revue Française d'Entomologie*, **24**, 232-243.

Coiffait, H. (1959) Les *Eusphalerum* (*Anthobium* auct.) de France et des régions voisines. *Bulletin de la Société d'Histoire Naturelle de Toulouse*, **94**, 213-252.

Coiffait, H. (1961) Les *Hypomedon* d'Europe et de la région Méditerranéenne [Coleoptera Staphylinidae]. *Revue Française d'Entomologie,* **28**, 16-40.

Coiffait, H. (1962) Les Leptotyphlitae (Col. Staphylinidae) de Californie. *Revue Française d'Entomologie*, **29**, 154-166.

Coiffait, H. (1965) Sur quelques Xantholininae de l'Asie Mineure (Coleoptera, Staphylinidae). *Reichenbachia*, **5**, 119-123.

Coiffait, H. (1966) Nouveaux Xantholinini du Caucase de du Turkestan (Col. Staphylinidae). *Bulletin de la Société Entomologique de France*, **71**, 123-126.

Coiffait, H. (1966) Novye Xantholinini iz sovetskogo soiuza (Coleoptera, Staphylinidae). *Zoologicheskii Zhurnal*, **45**, 195-202.

Coiffait, H. (1967) *Quedius* nouveaux ou mal connus. *Bulletin de la Société d'Histoire Naturelle de Toulouse*, **103**, 391-424.

Coiffait, H. (1970) Formes nouvelles ou mal connues des genres *Medon* et *Hypomedon*. *Annales de Spéléologie*, **24**, 702-727.

Coiffait, H. (1970) Un nouveau *Medon* cavernicole du Péloponnèse. *Annales de Spéléologie*, **25**, 231-233.

Coiffait, H. (1972) Coléoptères Staphylinidae de la région Paléarctique occidentale. I. Généralités. Sous-familles: Xantholininae et Leptotyphlinae. *Nouvelle Revue d'Entomologie*. Supplément, 2, i-ix + 1-651.

Coiffait, H. (1974) Coléoptères staphylinides de la région paléarctique occidentale II. Sous famille Staphilininae, Tribus Philonthini et Staphylinini. *Nouvelle Revue d'Entomologie*. Supplément. 4, 1-593. 1974a. Staphylinides récoltés en Ussuri (Asie Orientale) par S. M. Khnzorian-Iablokoff. *Nouvelle Revue d'Entomologie*, **4**, 197-204.

Coiffait, H. (1975) Staphylinides nouveaux d'U.R.S.S. récoltés par S. M. Khnzorian-Iablokoff. *Nouvelle Revue d'Entomologie*, **5**, 31-37.

Coiffait, H. (1976) Clef de détermination des *Medon* de la région Paléarctique occidentale avec description d'une espèce cavernicole nouvelle. *Annales de Spéléologie*, **31**, 229-243.

Coiffait, H. (1978) Coléoptères staphylinides de la région paléarctique occidentale III. Sous famille Staphylininae, Tribu Quediini. Sous famille Paederinae, Tribu Pinophilini. *Nouvelle Revue d'Entomologie, (Suppl.)*, **8**, 1-364.

Coiffait, H. (1978) Staphylinides nouveaux ou mal connus de la France et de la région Méditerranéenne. *Nouvelle Revue d'Entomologie*, **8**, 267-279.

Coiffait, H. (1978) Staphylinides récoltés par T. Deuve en Anatolie septentrionale (Col. Staph.). *Nouvelle Revue d'Entomologie*, **8**, 163-175.

Coiffait, H. (1982) Coléoptères Staphylinidae de la région Paléarctique Occidentale, IV. Sous famille Paederinae, Tribu Paederini 1 (Paederi, Lathrobii). *Nouvelle Revue d'Entomologie, (Suppl.)*, **12**, 7-440.

Coiffait, H. (1982) Contribution à la connaissance des Staphylinides de l'Himalaya (Népal, Ladakh, Cachemire) (Insecta: Coleoptera: Staphylinidae). *Senckenbergiana Biologica*, **62**, 21–179.

Coiffait, H. (1982) Staphylinides (Col.) de la région himalayenne et de l'Inde (I. Xantholininae, Staphylininae et Paederinae). *Entomologica Basiliensia*, **7**, 231-302.

Coiffait, H. (1984) Staphylinides (Col.) de la région himalayenne et de l'Inde. II. Tachyporinae, Oxytelinae et Aleocharinae. *Entomologica Basiliensia*, **9**, 116-157.

Cooter, J. & A. Kilian (2002) New species of *Leiodes* Latreille, 1796 (Col., Leiodidae) from China. *Entomologist's Monthly Magazine*, **138**, 157-164.

Cuccodoro, G. & I. Löbl (1995) Revision of the Afrotropical rove-beetles of the gepnus *Megarthrus* (Coleoptera, Staphylinidae, Proteininae). *Revue suisse de Zoologie*, **102**, 655-761.

Cuccodoro, G. & I. Löbl (1996) Revision of the rove-beetles of the genus *Megarthrus* of America north of Mexico (Coleoptera, Staphylinidae, Proteininae). *Mitteilungen Muenchener Entomologischen Gesellschaft*, **86**, 145-188.

Cuccodoro, G. & I. Löbl (1997) Revision of the Palaearctic rove beetles of the genus *Megarthrus* Curtis (Coleoptera: Staphylinidae: Proteininae). *Journal of Natural History*, **31**, 1347-1415.

Cuccodoro, G. (2011) Revision of the Neotropical types of *Megarthrus* Curtis, 1829 and description of two new species from Costa Rica and Peru Coleoptera, Staphylinidae, Proteininae. *Revue suisse de Zoologie*, **118**, 107-147.

Cuccodoro, G., and G. Makranczy (2013) Review of the Afrotropical species of *Deleaster* Erichson, 1839 (Coleoptera, Staphylinidae, Oxytelinae). *Revue suisse de Zoologie*, **120**, 537-547.

Cuppen, J. G. M. & O. Vorst (2002) *Ptinella denticollis* nieuw voor België (Coleoptera: Ptiliidae). *Phegea*, **30**, 187-191.

Daffner, H. (1984) *Bryaxis troglodytes lausbergeri*, eine neue Subspecies aus Norditalien (Coleoptera, Pselaphidae). *Entomologische Blätter*, **80**, 133-135.

Daffner, H. (1986) Die Arten der Gattung *Leiodes* Latreille (Coleoptera, Leiodidae) aus Indien und Nepal. *Revue suisse de Zoologie*, **93**, 71-87.

Daffner, H. (1986) Eine neue Art der Gattung *Bryaxis* Kugelann, 1794 aus Norditalien ‒Prealpi Bresciane (Coleoptera, Pselaphidae, Bythinini). *Acta Coleopterologica*, **1**, 37-40.

Daffner, H. (1986) *Euconnus* (*Tetramelus*) *lombardus* n. sp. aus Nord-Italien (Col. Scydmaenidae Stenichnini). *Acta Coleopterologica*, **2**, 21-24.

Daffner, H. (1986) Zwei neue Arten der Gattung *Bryaxis* Kugelann, 1794 aus Italien ‒Friaul und Slovenien (Coleoptera, Pselaphidae, Bythinini). *Acta Coleopterologica*, **8**, 51-59.

Dauphin, P. (1995) Description de *Proteinus meuseli*, nouvelle espèce d'Europe centrale (Coleoptera, Staphylinidae). *Bulletin de la Société Linnéenne de Bordeaux*, **23**, 193-197.

Dauphin, P. (1995) Sur les *Proteinus* d'europe occidentale (Coleoptera, Staphylinidae). *Bulletin de la Société Linnéenne de Bordeaux*, **23**, 101-118.

Dauphin, P. (1999) Description de deux nouveaux *Proteinus* méditerranéens (Coleoptera Staphylinidae). *Bulletin de la Société Linnéenne de Bordeaux*, **27**, 135-141.

Dauphin, P. (2004) Notes sur les *Cypha* (= Hypocyphtus) d'Europe occidentale (Coleoptera Staphylinidae Aleocharinae). *Bulletin de la Société Linnéenne de Bordeaux*, **32**, 85-102.

Dauphin, P. (2006) Sur quelques *Cypha* d'Afrique du Nord et d'Italie décrits par Fauvel (Coleoptera Staphylinidae Aleocharinae). *L'Entomologiste*, **62**, 147-150.

Deane, C. (1933) Australian Hydrophilidae--Notes and new species. No. 2. *Proceedings of The Royal Society of Victoria*, **46**, 20-27.

Dvořák, M. (1966) *Gyrophaena rousi* sp. n., eine neue Staphyliniden-Art aus der Slowakei (Col., Staphylinidae). *Annotationes Zoologicae et Botanicae*, **30**, 1-4.

Easton, A. M. (1971) A new British *Atheta* (Col., Staphylinidae) in a newe subgenus. *Entomologist's Monthly Magazine*, **107**, 24-26.

Enderlein, G. (1901) *Meropathus Chuni* nov. gen., nov. spec. Eine neue Helephorinengattung von der Kerguelen-Insel. *Zoologischer Anzeiger*, **24**, 121-124.

Eppelsheim, E. (1878) Staphylinidae. In O. Schneider and H. Leder, Beiträge zur Kenntniss der Kaukasischen Käferfauna. *Verhandlungen des naturforschenden Vereines in Brünn*, **16**, 90-131, pls. 1, 2.

Eppelsheim, E. (1880) [New species]. In E. Reitter, Coleopterologische Ergebnisse einer Reise nach Croatien, Dalmatien und der Herzegowina im Jahre 1879. *Verhandlungen der k. k. zoologisch-botanischen Gesellschaft in Wien*, **30**, 201-228.

Eppelsheim, E. (1884) Diagnosen neue Coleopteren aus Lenkoran. *Verhandlungen des naturforschenden Vereines in Brünn*, **22**, 11-16.

Eppelsheim, E. (1885) Beitrag zur Staphylinidenfauna West-Afrika's. *Deutsche Entomologische Zeitschrift*, **29**, 97-147.

Eppelsheim, E. (1889) Neue Staphylinen aus den Kaukasusländern, besonders aus Circassien. *Wiener Entomologische Zeitung*, **8**, 11-22.

Eppelsheim, E. (1890) Neue Staphylinen aus den Kaukasusländern. *Wiener Entomologische Zeitung*, **9**, 161-172.

Eppelsheim, E. (1892) Zur Staphylinenfauna Turkestan's. *Deutsche Entomologische Zeitschrift*, **1892**, 321-346.

Erichson, W. F. (1837) Die Käfer der Mark Brandenburg. 1(1): viii + 1-384. Berlin: F. H. Morin.

Erichson, W. F. (1839) Die Käfer der Mark Brandenburg. 1(2): 385-740, Berlin: F. H. Morin.

Erichson, W.F. (1839–1840) Genera et Species Staphylinorum Insectorum Coleopterorum Familiae. F. H. Morin, Berlin.

Ericson, J. B. (1909) [New species]. In: L. Gangibauer (Vorsitzender), Berichte der Sektion für Koleopterologie. Versammlung am 3. Dezember (1908) Verhandlungen der kaiserlich-königlichen zoologisch - botanischen Gesellschaft in Wien. Bd. **59**, 288-290.

Escolà, O. & J. Fresneda (2000) Las especies ibéricas del grupo de *Speonomus speluncarum* (Delarouzée, 1857). Descripción de *Speonomus ere* sp. n. del Pirineo de Huesca, España (Coleoptera, Cholevidae, Leptodirinae). *Miscellània Zoològica*, **23**, 35-43.

Escolà, O., X. Bellés & J. Comas (1986) *Speonomus tincatincensis* n. sp. y *S. saforensis* n. sp., nuevos Bathysciinae (Col., Catopidae) del Pallars, Lleida. *Miscellània Zoològica*, **9**, 223-227.

Fagel, G. (1960) Contribution à la connaissance des Staphylinidae. LXVII. Le complexe des Anisopsis. *Bulletin Institut royal des Sciences naturelles de Belgique*, **36**, 1-51.

Fagel, G. (1960) Contribution à la connaissance des Staphylinidae. LXVIII. Notes sur quelques espèces méditerranéennes. *Bulletin et Annales de la Société Royale d'Entomologie, de Belgique*, **96**, 222-233.

Fagel, G. (1965) Contribution à la l'étude des Staphylinidae, XCI. Quelques Aleocharinae nouveaux du Liban. *Bulletin et Annales de la Société Royale d' Entomologie, de Belgique*, **101**, 251-266.

Faille, A., I. Ribera & J. Fresneda. (2016) On the genus *Aphaobius* Abeille de Perrin, 1878, with description of a new species from the mesovoid shallow substratum (MSS) of Austria (Coleoptera: Leiodidae: Cholevinae: Leptodirini). *Zootaxa*, **4169**, 44-56.

Fairmaire, L. (1858) Miscellanea Entomologica. *Annales de la Société Entomologique de France*, (3)**5**, 725-745.

Fairmaire, L. (1889) Coléoptères de l'intérieur de la Chine. *Annales de la Société Entomologique de France*, (6)**9**, 7-84.

Fairmaire, L. (1893). Description d'une nouvelle espèce de Staphylinide. *Bulletin de la Société Entomologique de France*, **62**, 212.

Faldermann, F. (1835) Additamenta entomologica ad faunam rossicam in itineribus jussu imperatoris augustissimi annis 1827-1831 a Cl. Ménétriés et Szovitz susceptis collecta, in lucem edita. *Nouvelle Mémoires de la Société Impériale des Naturalistes de Moscou*, **4**, 1-310.

Fauvel, A. (1865) Énumération des insectes recueillis en Savoie et en Dauphine (1861-1863) et descriptions d'espèces nouvelles. *Bulletin de la Société Linnéenne de Normandie*, **9**, 253-321.

Fauvel, A. (1868) Faune du Chili. Insectes Coléoptères. Staphylinides. (Suite et fin). *Bulletin de la Société Linnéenne de Normandie*, (2)**1**, 6-67.

Fauvel, A. (1878) Les staphylinides des Moluques et de la Nouvelle Guinée. *Annali del Museo Civico di Storia Naturale di Genova*, **12**, 171-315.

Fauvel, A. (1879) Les staphylinides des Moluques et de la Nouvelle Guinée. (2e. Mémoire). *Annali del Museo Civico di Storia Naturale di Genova*, **15**, 63-121.

Fauvel, A. (1886) Staphylinides des Iles Philippines. *Revue d'Entomologie*, **5**, 143-150.

Fauvel, A. (1889) Les Coléoptères de la Nouvelle-Calédonie et dépendances avec descriptions, notes et synonymies nouvelles. *Revue d'Entomologie*, **8**, 242-271.

Fauvel, A. (1895) Staphylinides nouveaux de l'Inde et de la Malaisie. *Revue d'Entomologie*, **14**, 180-286.

Fauvel, A. (1899) Staphylinides nouveaux de Barbarie. *Revue d'Entomologie*, **18**, 97-99.

Fauvel, A. (1900) Staphylinides paléarctiques nouveaux. *Revue d'Entomologie*, **19**, 218-253.

Fauvel, A. (1900) Sur les Oxyteliens de Nouvelle-Zélande. *Revue d'Entomologie*, **19**, 181–189.

Fauvel, A. (1907) Voyage de M. Ch. Alluaud dans l'Afrique Orientale. Staphylinidae. *Revue d'Entomologie,* **26**, 10-70.

Fauvel, A. (1908) Staphylinides nouveaux d'Afrique tropicale. *Revue d'Entomologie*, **24**, 194-198.

Fauvel, A. (1908) Staphylinides nouveaux de Madagascar. 2e Partie. *Revue d'Entomologie*, **24**, 149-184.

Feldmann B, Peng Z, Li L-Z (2014) On the *Domene* species of China, with descriptions of four new species (Coleoptera, Staphylinidae). *Zookeys*, **456**, 109-138. doi: 10.3897/zookeys.456.8413

Feldmann, B. (2004) A new species of *Phloeocharis* Mannerheim from Mallorca (Spain) (Insecta, Coleoptera, Staphylinidae: Phloeocharinae). *Linzer biologische Beiträge*, **36**, 797-800.

Feldmann, B. (2007) On the identity of *Atheta mucronata* (Kraatz 1859) (Coleoptera: Staphylinidae, Aleocharinae). *Linzer biologische Beiträge*, **39**, 57-63.

Ferro, M. L. & C. E. Carlton (2014) Two new species of *Batrisodes* Reitter (Coleoptera: Staphylinidae: Pselaphinae) from eastern North America. *Insecta Mundi*, **0380**, 1-21.

Frank, J. H. (1975) A revision of the New World species of the genus *Erichsonius* Fauvel (Coleoptera: Staphylinidae). *Coleopterists' Bulletin*, **29**, 177-203.

Frank, J. H. (1979) A new species of *Proteinus* Latreille (Coleoptera: Staphylinidae) from Florida. *The Florida Entomologist*, **62**, 329-340.

Frank, J. H. (1981) A new *Erichsonius* species from Arizona with discussion on phylogeny within the genus (Coleoptera: Staphylinidae). *Coleopterists' Bulletin*, **35**, 97–106.

Frank, J. H. (1981) A revision of the New World species of the genus *Neobisnius* Ganglbauer (Coleoptera: Staphylinidae: Staphylininae). *Occasional Papers of the Florida State Collection of Arthropods*, 1: vii + 1-60.

Franz, H. (1960) Revision der *Stenichnus*Arten des Westlichen Mediterrangebietes sowie Mittel- und Nordwesteuropas (Col. Scydmaenidae). *Eos*, **36**, 277-371.

Franz, H. (1961) Nachträge zur Revision der *Stenichnus*-Arten des westlichen Mediterrangebietes sowie Mittel- und Nordeuropas. *Zoologische Anzeiger*, **167**, 15-28.

Franz, H. (1971) Revision der *Scydmaenus*-Arten der Sammlung Schaufuss aus Neu-Guinea. *Koleopterologische Rundschau*, **49**, 29-41.

Franz, H. (1975) Eine neue *Neuraphes*-Art aus Schweden (Col., Scydmaenidae). *Entomologisk Tidskrift*, **96**, 1-2.

Franz, H. (1979) Zur kenntnis der Hypqmedon-arten von den Kanarischen Inseln (Staphylinidae, Col.). *Koleopterologische Rundschau*, **54**, 65-71.

Franz, H. (1986) Drei neue *Euconnus*-Arten aus dem Kaukasus (Coleoptera, Scydmaenidae). *Zeitschrift der Arbeitsgemeinschaft Österreichischer Entomologen*, **38**, 41-45.

Franz, H. (1988) Zwei neue *Scydmaenus*-Arten aus Süd-China (Coleoptera, Scydmaenidae). *Elytron*, **2**, 23-25.

Franz, H. (1989) Eine neue *Scydmaenus*-Art (Coleoptera, Scydmaenidae) aus Neu-Guinea. *Revue suisse de Zoologie*, **96**, 279-280.

Franz, H. (1994) Vier neue *Euconnus*-Arten (Coleoptera, Scydmaenidae) aus Australien. *Zeitschrift der Arbeitsgemeinschaft Österreichischer Entomologen*, **46**, 99-102.

Fresneda, J. & A. Lagar (1990) *Hydraena* (*Phothydraena*) *hernandoi* n. sp., nueva especie de España (Coleoptera, Hydraenidae). *Annales de Limnologie*, **26**, 177-181.

Fresneda, J. & C. Hernando (1988) *Speonomus aldomai* sp. n. (Coleoptera, Catopidae) nuevo de l'Alta Ribagorça (LLeida). *Miscellània Zoològica*, **12**, 163-169.

Fresneda, J. & C. Hernando (1990) *Speonomus torresi* n. sp., nuevo Bathysciinae (Coleoptera, Catopidae) del Pallars Sobirà, Lleida. *Ilerda "Ciències"*, **48**, 85-90.

Fresneda, J. & C. Hernando (1991) *Speonomus kryophilos* n. sp. (Coleoptera, Cholevidae) nuevo Bathysciinae del Pirineo Catalán. *Ilerda "Ciències"*, **49**, 259-264.

Fresneda, J. & C. Hernando (1994) *Speonomus escollae* n. sp. (Coleoptera, Cholevidae) del M.S.S. del Prepirineo Oscense (España). *Mémoires de Biospéologie*, **21**, 63-66.

Fresneda, J., C. Hernando, & A. Lagar (1998) Revisión de los *Speonomus* Jeannel, 1908 de los grupos *bolivari* y *brieti* (Coleoptera: Cholevidae, Leptodirinae). *Zapateri: Revista Aragonesa de Entomologia*, **8**, 121-156.

Fresneda, J., J. L. Lencina & J. M. Salgado (2006) Descripción del primer *Catops* Paykull, 1798 endémico de la Península Ibérica: *Catops punctatulus* n. sp. (Coleoptera, Leiodidae, Cholevinae). *Nouvelle Revue d'Entomologie, (N.S.)*, **23**, 45-54.

Fresneda, J., P. Aguilera & C. Hernando (1994) *Hydraena* (*Haenydra*) *catalonica* n. sp. (Coleoptera, Hydraenidae) de la Península Ibérica. *Zapateri: Revista Aragonesa de Entomologia*, **4**, 81-86.

Freude, H. (1957) Die in Bayern nachgewiesenen und zu erwartenden *Anthobium*-Arten. *Entomologische Blätter für Biologie und Systematik der Käfer*, **53**, 100-113.

Ganglbauer, L. (1895) Die Käfer von Mitteleuropa. Die Käfer der österreichisch-ungarischen Monarchie, Deutschlands, der Schweiz, sowie des französischen und italienischen Alpengebietes. 2. Familienreihe Staphylinoidea. Theil I. Staphylinidae, Pselaphidae. 881 pp. Wien: Carl Gerold's Sohn.

Garrido, J., L. F. Valladares & J. A. Régil (1991) *Ochthebius* (*Asiobates*) *figueroi* n. sp. in the north of Spain (Col., Hydraenidae). *Entomologica Basiliensia*, **14**, 93-99.

Garrido-Gonzalez, J., L. F. Valladares-Diez & J. A. Regil-Cueto (1991) *Hydraena* (*Haenydra*) *polita* Kiesenwetter, 1849, nueva para la fauna de la Península Ibérica (Coleoptera Hydraenidae). *Bollettino della Societa Entomologica Italiana*, **122**, 205-210.

Gers, C. (1989) *Speonomus orgibetensis*, n. sp., Coléoptère Bathysciinae Troglobie du milieu souterrain superficiel. *Annales de la Société Entomologique de France*, (N.S.), **25**, 105-116.

Gerstaecker, C. E. A. (1867) Beitrag zur Insekten-Fauna von Zanzibar, nach dem während der Expedition des Baron v. d. Decken gesammelten Material zusammengestellt. *Archiv für Naturgeschichte*, **33**, 1-49.

Gestro, R. (1879) Descrizioni di nuove specie di Coleotteri raccolte nella regione Austro-Malese dal signor L. M. D'Albertis. *Annali del Museo Civico di Storia Naturale di Genova*, **14**, 552-565.

Giachino, P. M. (1985) *Choleva* (*s. str.*) *gabriellae* e *Sciodrepoides casalei* nuove specie della Kabylia e note corologiche sui Catopidi d'Algeria (Coleoptera, Catopidae). *Museo Regionale di Scienze Naturali Bollettino (Torino)*, **3**, 337-348.

Gildenkov, M. Yu. (2002) Classification of the genus *Carpelimus* (Coleoptera, Staphylinidae) in the Palaearctic Region: 1. Introduction and description of new taxa. *Zoologicheskii Zhurnal [Зоологический журнал]*, **81**, 1461-1475.

Gildenkov, M. Yu. (2003) Classification of the genus *Carpelimus* (Coleoptera, Staphylinidae) in the Palaearctic Region: 2. Keys to species of the subgenera *Carpelimus*, Paratrogophloeus, Myopinus, and Bucephalinus. *Zoologicheskii Zhurnal [Зоологический журнал]*, **82**, 22-34.

Gildenkov, M. Yu. (2003) Classification of the genus *Carpelimus* (Coleoptera, Staphylinidae) in the Palaearctic Region: 3. Keys to species of the subgenus *Trogophloeus* and *Troginus*. *Zoologicheskii Zhurnal [Зоологический журнал]*, **82**, 366-381.

Gildenkov, M. Yu. (2004) New and little known Palaearctic species of the staphylinid genus *Carpelimus* Leach (Coleoptera, Staphylinidae). *Entomologicheskoe Obozrenie [Энтомологическое обозрение]*, **83**, 538-552.

Gildenkov, M. Yu. (2007) A review of the fauna of the subgenus *Troginus*, genus *Carpelimus* (Coleoptera, Staphylinidae), from Tropical Africa. *Zoologicheskii Zhurnal [Зоологический журнал]*, **86**, 1315-1326.

Gildenkov, M. Yu. (2007) A review of the subgenus *Carpelimus s. str.* (Coleoptera, Staphylinidae) from tropical Africa. *Zoologicheskii Zhurnal [Зоологический журнал]*, **86**, 1073-1085.

Gravenhorst, J.L.C. (1802) Coleoptera Microptera Brunsvicensia nec non exoticorum quotquot exstant in collectionibus entomologorum Brunsvicensium in genera familias et species distribuit, Carolus Reichard, Brunsuigae.

Gravenhorst, J.L.C. (1806) Monographia Coleopterorum Micropterorum, Henricus Dieterich, Gottingae.

Grebennikov, K. A. (2002) Western Palaearctic species of the genus *Deleaster* Erichson, 1839 (Coleoptera: Staphylinidae: Oxytelinae). *Zoosystematica Rossica*, **10**, 373-378.

Gridelli, E. (1943) Quattordicesimo contributo alla conoscenza degli Staphylinini. I. Le specie europee del genere *Neobisnius* Ganglb. II. Note su alcune specie di *Philonthus*. *Atti del Museo Civico di Storia Naturale di Trieste*, **15**, 111-130.

Gusarov, V. (1993) New and little-known Palaearctic Staphylinidae (coleoptera). 5th communication. *Zoosystematica Rossica*, **1**, 65-74.

Gusarov, V. I. (1995) Two new species of *Medon* Stephens, 1832, from Turkmenistan Staphylinidae, Ceoleoptera[sic]. *Entomologische Blätter*, **91**, 47-52.

Gusarov, V. I. (2004) A revision of the Nearctic species of the genus *Halobrecta* Thomson, 1858 (Coleoptera: Staphylinidae: Aleocharinae) with notes on some Palaearctic species of the genus. *Zootaxa*, **746**, 1-25.

Hammond, P. (1975) The phylogeny of a remarkable new genus and species of gymnusine staphylinid (Coleoptera) from the Auckland Islands. *Journal of Entomology Series B, Taxonomy*, **44**, 153-173.

Hammond, P. M. (1971) Notes on British Staphylinidae 2. — On the British species of *Platystethus* Mannerheim, with one species new to Britain. *Entomologist's Monthly Magazine*, **107**, 93-111.

Hampe, C. (1863) Ein kleiner Beitrag zur gross-österreichischen Käferfauna. *Wiener Entomologische Monatschrift*, **7**, 285-290.

Hatch, M. H. (1957) The beetles of the Pacific Northwest. Part II, Staphyliniformia. University of Washington Publications in Biology, vol. 16. University of Washington Press, Seattle, 384 pp.

Hatch, M. H. (1957) The beetles of the Pacific Northwest. Part II: Staphyliniformia. ix + 1-384 pp. Seattle: University of Washington Press.

Háva, J., J. Schneider & J. Růžička, (1999) Four new species of carrion beetles from China (Coleoptera: Silphidae). *Entomological Problems*, **30**, 67-83.

Hayashi, Y. (1984) Notes on Staphylinidae from Taiwan (Col.), III. *Entomological Review of Japan*, **39**, 91-93.

Hayashi, Y. (1986) Revisional notes on *Catops nipponensis* Jeannel from Kyushu, Japan (Coleoptera: Catopidae). *Transactions of the Shikoku Entomological Society,* **17**, 185-187.

Hayashi, Y. (1987) The two new *Catops*-species from Tanba, Japan (Coleoptera: Catopidae). *Entomological Review of Japan*, **42**(suppl.), 81-85.

Hayashi, Y. (1987) The two new species of the genus *Tachinus* Gravenhorst (Coleoptera: Staphylinidae) from Japan and Taiwan. *Entomological Review of Japan,* **42**(suppl.), 13-19.

Hayashi, Y. (1988) Studies on Staphylinidae from Japan. II. *Entomological Review of Japan*, **43**, 17-23.

Hayashi, Y. (1994) A new *Catops*-species from Japan (Coleoptera: Catopidae). *Entomological Review of Japan*, **49**, 1-4.

Hayashi, Y. (1995) Studies on Staphylinidae (Coleoptera) from Japan, V. Revision on the type specimens of Amichrotus *excellens* Bernhauer and *Staphylinus* (*Xanthoocyptus*) [*sic*] *ganglbauerianus* Bernhauer. *Entomological Review of Japan*, **50**, 45-57.

Hayashi, Y. (1997) Studies on the Asian Staphylininae (Coleoptera, Staphylinidae). III. The characteristics of the Xanthopygini. *Elytra*, **25**, 475-492.

Hayashi, Y. (2000) A new species of *Proteinus* from Shikoku, Japan (Coleoptera: Staphylinidae: Proteininae). *Entomological Review of Japan*, **55**, 91-94.

Hayashi, Y. (2006) A new species of the *Omalium* from Japan (Coleoptera: Staphylinidae). *Entomological Review of Japan*, **61**, 75-79.

Hayashi, Y. (2007) A new species of the genus *Eushalerum* from Japan (Coleoptera: Staphylinidae: Omalinae[*sic*]). *Entomological Review of Japan*, **62**, 103-106.

Hayashi, Y. (2008) Three new species of *Proteinus* (Coleoptera: Staphylinidae) from Shikoku, Japan. *Special Publication of the Japan Coleopterological Society, Osaka*, **(2)**, 165-175.

Hayashi, Y. (2011) A new species of *Algon* (Coleoptera, Staphylinidae) from China, with some notes on the generic characteristics. *Elytra (new series)*, **1**, 67-72.

Hayashi, Y. (2012) Description of a new speices of *Dinothenarus* from China with some notes on the genus (Coleoptera, Staphylinidae). *The Japanese Journal of Systematic Entomology*, **18**, 437-442.

He, W.-J., L. Tang & L.-Z. Li (2008) A review of the genus *Scaphidium* Oliver (Coleoptera, Staphylinidae, Scaphidiinae) from Tianmushan, East China. *Zootaxa*, **1898**, 55-62.

He, W.-J., L. Tang & L.-Z. Li (2008) Notes on the genus *Scaphidium* Olivier of China with description of a new species (Coleoptera: Staphylinidae: Scaphidiinae). *Entomological Review of Japan*, **62**, 117-182.

He, W.-J., L. Tang & L.-Z. Li (2008) Three New Species of the Genus *Scaphidium* Olivier (Coleoptera; Staphylinidae: Scaphidiinae) from China. *Entomological Review of Japan*, **63**, 103-108.

He, W.-J., L. Tang & L.-Z. Li (2009) A new species and a new record species of the genus *Scaphidium* Olivier (Coleoptera, Staphylinidae, Scaphidiinae) from China. *Acta Zootaxonomica Sinica*, **34**, 481-484.

Heer, O. (1839) Fauna Coleopterorum Helvetica. 1(2): 145-360, Turici: Orelii, Fuesslini et Sociorum.

Henrot, H. (1962) Un *Choleva* nouveau du Maroc [Col. Catopidae]. *Revue française d'entomologie*, **29**, 44-47.

Herman, L. H. (1972) A revision of the rove-beetle genus *Charhyphus* (Coleoptera, Staphylinidae, Phloeocharinae). *American Museum Novitates*, **2496**, 1-16.

Herman, L. H. (1972) Revision of *Bledius* and related genera. Part 1, The *aequatorialis*, *mandibularis*, and *semiferrugineus* groups and two new genera (Coleoptera, Staphylinidae, Oxytelinae). *Bulletin of the American Museum of Natural History*, **149**, 113-253.

Herman, L. H. (1975) Revision and phylogeny of the monogeneric subfamily Pseudopsinae for the World (Staphylinidae, Coleoptera). *Bulletin of the American Museum of Natural History*, **155**, 241-318.

Herman, L. H. (1976) Revision of *Bledius* and related genera. Part 2. The *arrnatus*, *basalis*, and *melanocephalus* groups (Coleoptera, Staphylinidae, Oxytelinae). *Bulletin of the American Museum of Natural History*, **157**, 71-172.

Herman, L. H. (1983) *Pseudopsis*: Two New Species from India (Coleoptera, Staphylinidae, Pseudopsinae). *American Museum Novitates*, **2755**, 1-6.

Herman, L. H. (1983) Revision of *Bledius*. Part 3. The *annularis* and *emarginatus* groups (Coleoptera, Staphylinidae, Oxytelinae). *Bulletin of the American Museum of Natural History*, **175**, 1-146.

Herman, L. H. (2013) Revision of the new world species of *Oedichirus* (Coleoptera: Staphylinidae: Paederinae: Pinophilini: Procirrina). *Bulletin of the American Museum of Natural History*, **375**, 1-137.

Hernando, C. & M. Baena (2006) Un nuevo estafilínido cavernícola de Andalucía: *Domene* (*s. str.*) *gevia* sp. nov. (Coleoptera: Staphylinidae: Paederinae). *Heteropterus Revista de Entomología*, **6**, 29-33.

Hernando, C. (2002) *Phloeocharis* (*Scotodytes*) *montnegrensis* sp. nov., un nuevo estafilínido endógeo del noreste de la Península Ibérica (Coleoptera: Staphylinidae: Phloeocharinae). *Heteropterus Revista de Entomología*, **2**, 1-5.

Hernando, C., P. Aguilera, and I. Ribera (1999) *Limnebius alibeii* sp. n. (Coleoptera: Hydraenidae) from Morocco. *Aquatic Insects*, **21**, 141-145.

Hernando, C., P. Aguilera, and I. Ribera (2008) *Limnebius zaerensis*, a new species from the Pays Zaër-Zaïane, central Morocco. *Koleopterologische Rundschau*, **78**, 195-198.

Hlaváč, P. (2008) A new cavernicolous species of the genus *Bryaxis* (Coleoptera: Staphylinidae: Pselaphinae) from the island of Mljet. *Natura Croatica*, **17**, 1-8.

Hlaváč, P. (2009) Taxonomic notes on the *Bryaxis splendidus* species group (Coleoptera: Staphylinidae: Pselaphinae), with the description of a new species from the Ukraine. *Acta Entomologica Musei Nationalis Pragae*, **49**, 651-659.

Hlisnikovský, J. (1964) Zur Kenntnis der Gattung *Agyrtes* Lap. (Coleoptera, Silphidae, Agyrtini). *Reichenbachia*, **62**, 275-278.

Hochhuth, J. H. (1849) Die Staphylinen-Fauna des Kaukasus und Transkaukasiens. *Bulletin de la Société Impériale des Naturalistes de Moscou*, **22**, 18-214.

Hoebeke, E. R. & J. S. Ashe (1994) New species of *Autalia* Leach 1819 from the Neotropics (Coleoptera Staphylinidae Aleocharinae), with new distributional data for *A. phricotrichosa* Hoebeke 1988 and key to the Neotropical species of *Autalia*. *Tropical Zoology*, **7**, 191-208.

Hoebeke, E. R. (1995) Three Palearctic species of *Rugilus* Leach in North America (Coleoptera: Staphylinidae: Paederinae): redescriptions, new synonymy, and new records. *Insecta Mundi*, **9**, 69-80.

Hoebeke, E. R. (2010) *Rugilus ceylanensis* (Kraatz) (Coleoptera: Staphylinidae: Paederinae): a South Asian rove beetle new to North America. *Proceedings of the Entomological Society of Washington*, **112**, 508-516.

Hopp, K. & M. Caterino (2009) Seven new species of *Cephennium* Müller & Kunze (Coleoptera, Staphylinidae, Scydmaeninae, Cephenniini) from California with a key to native North American species. *Zookeys*, **24**, 31-54.

Horn, G. H. (1880) Synopsis of the Silphidae of the United States with reference to the genera of other countries. *Transactions of the American Entomological Society*, **8**, 219-322.

Hoshina, H. & H. Sugaya (2003) A taxonomic study of the genus *Ptomaphaginus* (Coleoptera: Leiodidae: Cholevinae) from the Ryukyu Islands, Japan. *Entomological Review of Japan*, **58**, 121-131.

Hoshina, H. (2006) A redescription of *Scydmaenus takaranus* (Coleoptera, Scydmaenidae) in Japan. *Elytra*, **34**, 103-106.

Hoshina, H. (2007) A new species of the genus *Euconnus* (Coleoptera: Scydmaenidae) from Fukui Pref., Honshu, Japan. *Bulletin of the Regional Environmental Research and Education Center, University of Fukui, "Nature and Environment Sea Area"*, **(14)**, 17-22.

Hoshina, H. (2009) A taxonomic revision of the subfamily *Colon*inae (Coleoptera: Leiodidae) from Japan and Taiwan. *Tijdschrift voor entomologie*, **152**, 237-286.

Hoshina, H. (2012) Review of the tribes Sogdini and Leiodini from Japan and North Chishima Islands. Part II. Genera *Hydnobius* and *Leiodes* (Coleoptera: Leiodidae). *Acta Entomologica Musei Nationalis Pragae*, **52**(suppl. 1), 1-168.

Hoshina, H., S. Arai & H. Ushijima (2003) Descriptions of two new species of the genus *Euconnus* (Coleoptera: Scydmaenidae) from Japan. *Entomological Review of Japan*, **58**, 27-33.

Hu, J.-Y., L.-Z. Li & M.-J. Zhao (2012) Three new species of *Anchocerus* Fauvel (Coleoptera: Staphylinidae: Staphylininae), with an updated key to Chinese species. *Zootaxa*, **3318**, 57-62.

Hu, J.-Y., L.-Z. Li & Y.-L. ZHAO (2010) A new species of the genus *Anchocerus* Fauvel, 1905 from China (Coleoptera: Staphylinidae: Staphylininae). *Zootaxa*, **2523**, 65-68.

Hu, J.-Y., T.-T. Liu, and L.-Z. Li (2011) New and Little-Known Species of the Genus *Bolitogyrus* Chevrolat from China (Coleoptera: Staphylinidae: Staphylininae). *Journal of the Kansas Entomological Society*, **84**, 58-63.

Huang, J.-J., M.-J. Zhao, L.-Z. Li & Y. Hayashi (2006) Four new species of the genus *Oxyporus* from China (Coleoptera: Staphylinidae: Oxyporinae). *Entomological Review of Japan*, **61**, 205-213.

Hubenthal, W. (1911) Die Gattung *Pseudopsis* Newmann. *Entomologische Blätter*, **1911**, 97-103.

Hwang, W.-S. & K.-J. Ahn (2000) Taxonomy of the Korean Oxyporinae (lnsecta, Coleoptera, Staphylinidae). *The Korean Journal of Systematic Zoology*, **16**, 191-202.

Irmler, U. (2006) New species and synonymies of the genus *Nacaeus* Blackwelder, 1942 (Coleoptera: Staphylinidae: Osoriinae) from the Neotropical Region. *Arthropod Systematics & Phylogeny*, **64**, 205-216.

Irmler, U. (2010) A new genus of Osoriinae in the Neotropical region with a cladistic analysis of the tribe Thoracophorini (Insecta: Coleoptera: Staphylinidae). *Arthropod Systematics & Phylogeny*, **68**, 229-237.

Irmler, U. (2015) Osoriinae of Cuba with description of new species and an identification key (Coleoptera: Staphylinidae). *Acta Entomologica Musei Nationalis Pragae*, **55**, 145-172.

Irmler, U. (2015) The neotropical genus *Glyptoma* Erichson, 1839 with descriptions of new species and a key to the species (Coleoptera: Staphylinidae: Osoriinae). *Beiträge zur Entomologie*, **65**, 297-325.

Irmler, U. (2015) Three new species of the genus *Heterocylindropsis* Fagel, 1955 from Tanzania (Coleoptera, Staphylinidae: Osoriinae). *Folia Entomologica Hungarica*, *Rovartani Közlemények*, **76**, 5-14.

Israelson, G. (1969) Some additions to the Coleopterous fauna of the Canary Islands. *EOS*, **44**, 149-157.

Israelson, G. (1972) The *Stenichnus* species of the Canary Islands (Col., Scydmaenidae). *Entomologische Blätter*, **68**, 103-114.

Ito, T. (1982) A new species of the genus *Platydracus* from Japan (Coleoptera, Staphylinidae). *Entomological Review of Japan*, **37**, 61-63.

Ito, T. (1991) A new species of *Anotylus gibbulus* group from Japan (Coleoptera, Staphylinidae). *Entomological Review of Japan*, **46**, 87-90.

Ito, T. (1994) Notes on the species of Staphylinidae from Japan, VI (Coleoptera). *Entomological Review of Japan*, **49**, 81-86.

Jäch, M. A., and J. A. Delgado (2012) *Limnebius dioscoridus* sp. nov. from Socotra Island (Coleoptera: Hydraenidae). *Acta Entomologica Musei Natioalis Pragae*, **52**(suppl. 2), 131-134.

Jacquelin Du Val, P. N. C. (1852) Description de deux genres nouveaux et de plusieurs espèces nouvelles de coléoptères propres a la faune francaise. *Annales de la Société Entomologique de France*, **2**, 695-718.

Jałoszyński, P. & K. Arai (2009) First record of *Stenichnus* Thomson (Coleoptera, Scydmaenidae) from Honshu, Japan, with description of *S. sakurayamanus* sp. nov. *Elytra*, **37**, 65-69.

Jałoszyński, P. (2004) A new species of *Stenichnus* Thomson (Coleoptera, Scydmaenidae) from Nakanoshima Is., Tokara Archipelago, Japan. *Bulletin of the National Science Museum (A)*, **32**, 53-56.

Jałoszyński, P. (2004) Redescriptions and new records of Japanese species of *Neuraphes* Stephens and *Scydmoraphes* Reitter (Insecta, Coleoptera, Scydmaenidae). *Bulletin of the National Science Museum (A)*, **30**, 137-145.

Jałoszyński, P. (2004) Revision of *Stenichnus* Thomson (Insecta, Coleoptera, Scydmaenidae) of Japan and Taiwan. *Bulletin of the National Science Museum (A)*, **30**, 155-171.

Jałoszyński, P. (2008) Revision of *Neuraphes* Thomson of the Himalaya Mts. (Coleoptera: Scydmaenidae). *Genus*, **19**, 619-637.

Jałoszyński, P. (2009) Two new species of *Stenichnus* Thomson from China (Coleoptera, Scydmaenidae). *Genus*, **20**, 27-34.

Jałoszyński, P. (2010) *Neuraphes hengduanus* n. sp. from Yunnan, China (Coleoptera: Staphylinidae: Scydmaeninae). *Genus*, **21**, 495-499.

Jałoszyński, P. (2010) Two new species of Himalayan and sub-Himalayan *Neuraphes* Thomson (Coleoptera: Staphylinidae: Scydmaeninae). *Stuttgarter Beiträge zur Naturkunde A (Neue Serie)*, **3**, 127-131.

Jałoszyński, P. (2013) *Neuraphes pseudojumlanus* n. sp. from Yunnan, China (Coleoptera: Staphylinidae: Scydmaeninae). *Genus*, **24**, 149-154.

Jałoszyński, P. (2013) Phylogeny of a new supertribe Cephenniitae with generic review of Eutheiini and description of a new tribe Marcepaniini (Coleoptera: Staphylinidae: Scydmaeninae). *Systematic Entomology*, **39**, 159-189.

Janák, J. & Gy. Makranczy. (2016) Description of a blind and flightless species of *Thinobius* from South Africa (Coleoptera: Staphylinidae: Oxytelinae). *Acta Entomologica Musei Nationalis Pragae*, **56**, 203–210.

Janák, J. (1998) Eine neue Art und neue Funde der Gattung *Stenus* aus Uganda (Coleoptera: Staphylinidae). *Entomological Problems*, **29**, 91-93.

Janák, J. (2010) On the genus *Thyreocephalus* from Africa south of Sahara with description of four new species (Coleoptera: Staphylinidae: Xantholinini). *Studies and Reports Taxonomical Series*, **6**, 129-147.

Jarrige, J. (1941) Staphylinides nouveaux ou mal connus de France. *Bulletin de la Société Entomologique de France*, **46**, 46-50.

Jarrige, J. (1963) Nouveaux Brachyélytres pyrénéens. *Bulletin de la Société Entomologique de France*, **67**, 164-168.

Jarrige, J. (1965) Les *Acylophorus* malgaches (Col. Staphylinidae). *Bulletin de la Société Entomologique de France,* **70**, 139-143.

Jarrige, J. (1965) Un *Tachinus* nouveau d'Italie. *L'Entomologiste*, **21**, 99-101.

Jarrige, J. (1966) *Stenus* nouveaux des Comores (Col. Stenidae). *Bulletin de la Société Entomologique de France*, **71**, 121-123.

Jarrige, J. (1970) Contribution a l'étude des Coleoptera Brachelytra du massif du Tsaratanana (Madagascar Nord). In: Nouvelles données scientifiques sur le massif du Tsaratanana (Madagascar). *Mémoires O.R.S.T.O.M.*, **37**, 31-63.

Jarrige, J. (1971) Contribution a la faune de L'Iran. 21. Coléoptères Brachelytra. *Annales de la Société Entomologique de France*, **7**, 483-502.

Jarrige, J. (1972) Nouveaux Coléoptères pyrénéens. *L'Entomologiste*, **28**, 155-159.

Jarrige, J. (1972) Nouveaux Staphylinides malgaches. *Bulletin de la Société Entomologique de France*, **77**, 135-137.

Jeannel, R. (1911) Biospeologica XIX. Révision des Bathysciinae (Coléoptères Silphides). Morphologie, distribution géographique, systématique. *Archives de Zoologie Expérimentale et Générale*, (5)**7**, 1-641, pls. 1-24.

Jeannel, R. (1934) Les *Ptomaphagus* Paléarctiques (Col.). *Revue française d'entomologie*, **1**, 161-170.

Jeannel, R. (1936) Monographie des Catopidae [Insectes Coleopteres]. *Memoires du Museum National Histoire Naturelle (n.s.)*, **1**, 1-438.

Jeannel, R. (1953) Sur la faune entomologique de l'ile Marion. *Revue française d'entomologie*, **20**, 161-167.

Jeon, M.-J. & K.-J. Ahn (2002) The Korean species of the intertidal genus *Cafius* (Coleoptera, Staphylinidae, Staphylininae). *The Korean Journal of Systematic Zoology*, **18**, 65-74.

Johnson, C. (1975) Arthropoda of the subantarctic islands of New Zealand - *8. Coleoptera: Ptiliidae. *New Zealand Journal of Zoology*, **2**, 9-14.

Johnson, C. (1975) Ptiliidae (Coleoptera) from the Kermadec Islands. *The New Zealand Entomologist*, **6**, 56-58.

Johnson, C. (1988) Revision of Sri Lankan Acrotrichines (Coleoptera: Ptiliidae). *Revue suisse de Zoologie*, **95**, 257-275.

Joy, N. H. (1921) *Phyllodrepa linearis* Zett.: a new British Staphylinid beetle. *Entomologist's Monthly Magazine*, **57**, 142.

Kangas, E. (1957) Eine neue *Gnypeta*-Art (Col., Staphylinidae) aue Sibirien. *Sonderabdruck aus Suomen Hyönteistieteellinen Aikakauskirja-Annales Entomologici Fennici*, **23**, 85-86.

Kapp, A. (2010) Eine neue Art der Gattung *Bolitochara* Mannerheim 1830 aus Sizilien (Italien) (Staphylinidae, Aleocharinae). *Linzer biologische Beiträge*, **42**, 1515-1520.

Karsch, F. (1881) Die Käfer der Rohlfs'schen Afrikanischen Expedition 1878-79. *Berliner Entomologische Zeitschrift*, **25**, 41-50, pl. II.

Kasapoğlu, A., O. Erman & Ö. K. Erman (2004) First record of *Ochthebius* (*s. str*) *minabensis* Ferro, 1983 (Coleoptera, Hydraenidae) from Turkey. *Turkish Journal of Zoology*, **28**, 217-218.

Kim, H.-J. & K.-J. Ahn (2000) Korean species of the genus *Sepedophilus* Gistel (Coleoptera, Staphylinidae, Tachyporinae). *Insecta Koreana*, **17**, 251-264.

Kim, T.-K. & K.-J. Ahn (2011) Taxonomy of Korean *Lesteva* with a description of a new species (Coleoptera: Staphylinidae: Omaliinae). *Florida Entomologist*, **94**, 28-34.

Kim, Y.-H. & Ahn, K.-J. (2009) Four *Gyrophaena* species new to Korea (Coleoptera: Staphylinidae: Aleocharinae). *The Korean Journal of Systematic Zoology*, **25**, 159-165.

Kim, Y.-H. & Ahn, K.-J. (2010) New synonyms and redescriptions of three species of the mycophagous genus *Gyrophaena* (Coleoptera: Staphylinidae: Aleocharinae) in East Asia. *Florida Entomologist*, **93**, 333-338.

Kim, Y.-H. & K.-J. Ahn (2014) Taxonomy of the genus *Homalota* Mannerheim in Korea (Coleoptera, Staphylinidae, Aleocharinae). *Zookeys*, **447**, 109-123.

Kim, Y.-H. & K.-J. Ahn. (2016) Taxonomy of the genus *Phymatura* J. Sahlberg (Coleoptera: Staphylinidae: Aleocharinae) in the Korean Peninsula. *Zootaxa*, **4061**, 189-196.

Kimura, F. (2008) Two New Species of the Genus *Scaphidium* (Coleoptera: Staphylinidae) from Japan. *Special Publication of the Japan Coleopterological Society*, **(2)**, 157-164.

Kistner, D. H. (1962) A revision of the Nearctic and Ethiopian species of the genus *Edaphus* (Coleoptera: Staphylinidae). *Annals of the Entomological Society of America*, **55**, 619-632.

Klimaszewski, J. (1979) A revision of the Gymnusini and Deinopsini of the world (Coleoptera: Staphylinidae: Aleocharinae). *Research Branch Agriculture Canada Monograph*, **25**, 1-169.

Klimaszewski, J. (1984) A revision of the genus *Aleochara* Gravenhorst of America north of Mexico (Coleoptera: Staphylinidae, Aleocharinae). *Memoirs of the Entomological Society of Canada*, **129**, 1-211.

Klimaszewski, J. (1992) Review of the Afrotropical species of the genus *Autalia* Leach with descriptions of four new species (Coleoptera: Staphylinidae: Aleocharinae). *Journal of African Zoology*, **106**, 513-525.

Klimaszewski, J., and C. G. Majka (2012) Two new *Atheta* species (Coleoptera: Staphylinidae: Aleocharinae) from eastern Canada: taxonomy, bionomics, and distribution. *The Canadian Entomologist*, **139**, 45-53.

Klimaszewski, J., G. Pelletier & C. Majka (2004) A revision of Canadian *Leptusa* Kraatz (Col., Staphylinidae, Aleocharinae): new species, new distribution records, key and taxonomic considerations. *Belgian Journal of Entomology*, **6**, 3-42.

Klimaszewski, J., K. Savard, G. Pelletier, and R. Webster (2008) Species review of the genus *Gnypeta* Thomson from Canada, Alaska and Greenland (Coleoptera, Staphylinidae, Aleocharinae): systematics, bionomics and distribution. *Zookeys*, **2**, 11-84.

Kocian, M. (1997) A revision of Western Palearctic species of the genus *Ischnosoma* Stephens (Coleoptera, Staphylinidae: Tachyporinae). *Acta Universitatis Carolinae. Biologica*, **40**, 241-299.

Kocian, M. (2003) Monograph of the world species of the genus *Ischnosoma* (Coleoptera: Staphylinidae). *Acta Universitatis Carolinae. Biologica*, **47**, 241-299.

Kolenati, F. A. (1846) Meletemata entomologica. Fasc. III. Brachelytra caucasi cum distributione geographica adnexis pselaphinis, scydmaenis, notoxidibus et xylophagis. 44 pp., 3 pls. Petropolis: Imperialis Academiae Scientiarum.

Korge, H. (1971) Beiträge zur Kenntnis der Koleopterenfauna Kleinasiens. Annotationes Zoologicae et Botanicae, **67**, 1-68.

Kraatz, G. (1857) Naturgeschichte der Insecten Deutschlands. Abt. 1. Coleoptera. Zweiter Band. Lief. 3-4. pp. 377-768. Lief. 5-6. pp. 769-1080. Berlin: Nicolai.

Kraatz, G. (1858) Beitrag zur Käferfauna Griechenlands. Zweites Stück: Palpicornia, *Silpha*les, Scydmaenidae, Pselaphidae, Staphylinidae. *Berliner Entomologische Zeitschrift*, **2**, 37-67.

Kraatz, G. (1859) Die Staphylinen-Fauna von Ostindien, insbesondere der Insel Ceylan. *Archiv für Naturgeschichte*, **25**, 1-196.

Kraatz, G. (1859) Über die Gattungen *Micropeplus, Thorictus, Antidipnis, Cnemeplatia* und *Foucartia*. *Berliner Entomologische Zeitschrift*, **3**, 65-78.

Kraatz, G. (1899) *Leistotrophus* (?) *giganteus* nov. spec. (Staphylinidae). *Deutsche Entomologische Zeitschrift*, **1899**, 112.

Kurbatov, S. A. & I. Löbl (1995) Contribution to the Knowledge of the East Asian *Bryaxis* (Coleoptera, Staphylinidae, Pselaphinae). *Archives des Sciences (Genèva)*, **48**, 161- 172.

Kurbatov, S. A. & I. Löbl (1998) Nouvelles espèces asiatiques du genre *Bryaxis* et quelques données sur des espèces connues (Coleoptera: Staphylinidae: Pselaphinae). *Revue suisse de Zoologie*, **105**, 823-833.

Kurbatov, S. A. (1988) Species of the genus *Euplectus* (Coleoptera, Pselaphidae) deom the Far East of the USSR. *Zoologicheskii Zhurnal [Зоологический Журнал]*, **67**, 1085-1091.

Kurbatov, S. A. (1993) Beetles of the genus *Scydmaenus* (Coleoptera, Scydmaenidae) from the Russian Far East. *Zoologicheskii Zhurnal [Зоологический Журнал]*, **72**, 152-155.

Kurbatov, S. A. (1993) Scydmaenid beetles of the genera *Stenichnus* Thoms. and *Euconnus* Thoms. (Coleoptera, Scydmaenidae) of Russian Far East. *Entomological Review [Энтомологическое Обозрение]*, **72**, 591-596.

Last, H. R. (1948) *Neobisnius cerrutii* Gridelli and var. *rubripennis* Gridelli (Col., Staphylinidae), additions to the British list of Coleoptera. *Entomologist's Monthly Magazine*, **84**, 148-150.

Last, H. R. (1954) A new species of African *Rugilus* (Col. Staphylinidae). *Bulletin de l'Institut royal des Sciences naturelles de Belgique*, **30**, 1-3.

Last, H. R. (1981) Records of the genera *Philonthus*, *Gabrius*, *Neobisnius* and *Hesperus* from New Guinea with descriptions of new species (Coleoptera: Staphylinidae). *Folia Entomologica Hungarica*, **34**, 117-134.

LeConte, J. L. (1863) New species of North American Coleoptera. Part I. *Smithsonian Miscellaneous Collections*, **6**, 1-92.

LeConte, J. L. (1863) New species of North American Coleoptera. Prepared for the Smithsonian Institution. *Smithsonian Miscellaneous Collections*, **6**, 1-177.

Leschen, R. A. B., M. S. Bullians, B. Michaux, and K. Ahn (2002) Systematics of Baeostethus chiltoni, a subantarctic liparocephaline (Coleoptera: Staphylinidae: Aleocharinae): A Pangean relic or a more recent immigrant? *Journal of the Royal Society of New Zealand*, **32**, 189-201.

Levasseur, L. (1969) *Neobisnius* et *Erichsonius* (Philonthini) nouveaux d'Afrique. Col. Staphylinidae. *Bulletin de l'Institut Fondamental d'Afrique Noire (A)*, **31**, 881-898.

Li, L. & H.-Z. Zhou (2010) Revision of the Chinese species of the genus *Belonuchus* Nordmann (Coleoptera: Staphylinidae: Philonthina). *Journal of Natural History*, **44**, 2149-2177.

Li, L.-Z. & M. Ohbayashi (1996) The genus *Tachinus* (Coleoptera, Staphylinidae) from the Himalayas with descriptions of three new species. *Japanese Journal of Entomology*, **64**, 151-162.

Li, L.-Z. & M. Ohbayashi (1997) *Lordithon aitai*, a new tachyporine species, (Coleoptera, Staphylinidae) from Tsushima Island, Japan. *The Japanese Journal of Systematic Entomology*, **3**, 95-98.

Li, L.-Z. & M. Sakai. (1996) Two new species of the genus *Lordithon* (Coleoptera, Staphylinidae) form [*sic*] Japan. *The Japanese Journal of Systematic Entomology*, **2**, 251-256.

Li, L.-Z. & M.-J. Zhao (2001) *Micropeplus shanghaiensis*, a new species (Coleoptera, Staphylinidae) from East China. *The Japanese Journal of Systematic Entomology*, **7**, 91-94.

Li, L.-Z. & M.-J. Zhao (2002) Description of a new species of the genus *Tachinus* (Coleoptera, Staphylinidae) from East China. *The Japanese Journal of Systematic Entomology*, **8**, 13-15.

Li, L.-Z. & M.-J. Zhao (2003) A new *Tachinus* (Coleoptera, Staphylinidae, Tachyporinae) from Hubei Province, Central China. *The Japanese Journal of Systematic Entomology*, **9**, 177-179.

Li, L.-Z. & M.-J. Zhao (2003) *Tachinus satoi* (Coleoptera, Staphylinidae), a new tachyporine species from China. *Special Bulletin of the Japanese Society of Coleopterology*, **(6)**, 157-160.

Li, L.-Z. & M.-J. Zhao (2005) Two new species of the genus *Tachinus* (Coleoptera, Staphylinidae) from Tibet, China. *The Japanese Journal of Systematic Entomology*, **11**, 67-71.

Li, L.-Z. (1995) A revision of the genus *Tachinus* Gravenhorst (Coleoptera, Staphylinidae) of Japan, I. *The Japanese Journal of Systematic Entomology*, **1**, 51-72.

Li, L.-Z. (1995) A revision of the genus *Tachinus* Gravenhorst (Coleoptera, Staphylinidae) of Japan, II. *The Japanese Journal of Systematic Entomology*, **1**, 201-216.

Li, W.-R., N.-N. Xie & L.-Z. Li. (2015) Redescription of *Oedichirus flammeus* Koch, and description of two new *Oedichirus* species from China (Coleoptera, Staphylinidae, Paederinae, Pinophilini). *Zootaxa*, **3911**, 81-90.

Li, X. & H. Zhou (2009) Phylogenetic analysis and taxonomy of the subgenus Gnathopaederus Chapin and related groups of *Paederus* s.l. (Coleoptera: Staphylinidae: Paederinae). *Invertebrate Systematics*, **23**, 422-444.

Li, X.-J., L.-Z. Li & M.-J. Zhao (2005) A new species of the genus *Lesteva* (Coleoptera: Staphylinidae: Omaliinae) from China. *Entomotaxonomia*, **27**, 111-113.

Li, X.-Y. & H.-Z. Zhou (2007) Study on the subgenus *Harpopaederus* of the genus *Paederus* Fabricius (Coleoptera, Staphylinidae, Paederinae) from China. *Deutsche Entomologische Zeitschrift*, **54**, 219-233.

Li, X.-Y. & H.-Z. Zhou (2008) Taxonomy and biogeography of the subgenus *Eopaederus* of the genus *Paederus* Fabricius (Coleoptera: Staphylinidae: Paederinae) from China. *Annales de la Société Entomologique de France, (N.S.)*, **44**, 327-344.

Li, X.-Y. & H.-Z. Zhou (2009) A review of Chinese species of the subgenus *Paederus s. str.* (Coleoptera: Staphylinidae: Paederinae) with description of a new species. *Zootaxa*, **2083**, 46-64.

Li, X.-Y., H.-Z. Zhou, and A. Solodovnikov (2013) Five new species of the genus *Paederus* from mainland China, with a review of the Chinese fauna of the subtribe Paederina (Coleoptera: Staphylinidae: Paederinae). *Annals of the Entomological Society of America*, **106**, 562-574.

Löbl, I. & F. G. Calame (1996) Taxonomy and phylogeny of the Dasycerinae (Coleoptera: Staphylinidae). *Journal of Natural History*, **30**, 247-291.

Löbl, I. & J. Mattila (2010) *Euplectus lapponicus* (Coleoptera: Staphylinidae: Pselaphinae), a new species from boreal Finland. *Entomologica Fennica*, **21**, 181-186.

Löbl, I. & S. A. Kurbatov (1996) The *Bryaxis* of Taiwan (Coleoptera: Staphylinidae: Pselaphinae). *Bulletin of the national Museum of Natural Science*, **(8)**, 1-21.

Löbl, I. (1976) The Australian species of *Scaphidium* Olivier (Coleoptera: Scaphidiidae). *Journal of the Australian Entomological Society*, **15**, 285-295.

Löbl, I. (1978) Contribution to the knowledge of the new Guinean species of *Scaphidium* (Coleoptera: Scaphidiinae). *Pacific Insects*, **19**, 115-119.

Löbl, I. (1988) Trois espèces nouvelles du genre *Dasycerus* Brongniart (Coleoptera, Dasyceridae). *Archives des Sciences, Genève*, **41**, 259-265.

Löbl, I. (1992) The Scaphidiidae (Coleoptera) of the Nepal Himalaya. *Revue suisse de Zoologie*, **99**, 471-627.

Löbl, I. (1999) A review of the Scaphidiinae (Coleoptera: Staphylinidae) of the People's Republic of China, I. *Revue suisse de Zoologie*, **106**, 691-744.

Löbl, I. (2002) On *Baeocera* (Coleoptera: Staphylinidae: Scaphidiinae) of new Guinea. Mitteilungen der schweizerischen *Entomologischen Gesellschaft*, **75**, 1-20.

Löbl, I. (2003) A supplement to the knowledge of the Scaphidiines of China. Mitteilungen der Münchner *Entomologischen Gesellschaft*, **93**, 61-76.

Löbl, I., and R. A. B. Leschen (2003) Scaphidiinae (Insecta: Coleoptera: Staphylinidae). vol. 48. Lincoln, Canterbury (New Zealand): Manaaki Whenua Press, 94 pp.

Löbl, I., S. A. Kurbatov & S. Nomura (1998) On the Japanese species of *Bryaxis* (Coleoptera: Staphylinidae: Pselaphinae), with notes on allied genera and on Endoskeletal Polymorphy. *Species Diversity*, 1998, 219-269.

Löbl, L. & D. Burckhardt. (1988) *Cerapeplus* gen.n. and the classification of micropeplids (Coleoptera: Micropeplidae). *Systematic Entomology*, 13, 57-66.

Lohse, G. A. (1964) Staphylinidae I. (Micropeplinae bis Tachyporinae). In H. Freude, K. Harde and G. Lohse, Die Käfer Mitteleuropas. 4, 1-264. Krefeld: Goecke & Evers.

Lohse, G. A. (1989) *Gnypeta groenlandica* sp. n., eine neue Staphylinide aus Grönland. *Entomologische Blätter*, **85**, 58-60.

Lohse, G. A., and W. H. Lucht (1989) Die Käfer Mitteleuropas. Supplementband 1 mit Katalogteil. 346 pp. Krefeld: Goecke and Evers.

Lohse, G.A. (1967) Zwei neue westeuropäische Arten der Gattung *Lesteva* Latr. *Entomologische Blätter*, **63**: 10-12.

Lü, L. & H.-Z. Zhou (2012) Taxonomy of the genus *Oxytelus* Gravenhorst (Coleoptera: Staphylinidae: Oxytelinae) from China. *Zootaxa*, **3576**, 1-63.

Lü, L. & H.-Z. Zhou (2015) Review of the genus *Platystethus* Mannerheim (Coleoptera: Staphylinidae: Oxytelinae) in China. *Zootaxa*, **3915**, 151-205.

Lü, L. & H.-Z. Zhou (2015) Revision of the genus *Oxytelus* Gravenhorst (Staphylinidae: Oxytelinae) in Southeast Asia. *Zootaxa*, **3992**, 1-94.

Lundkvist, H. (2009) *Euaesthetus superlatus* Peyerimhoff, 1937 (Staphylinidae) och *Corticarina alemannica* Schiller, 1984 (Latridiidae) tva för Norden nya skalbaggsarter. *Entomologisk Tidskrift*, **130**, 141-144.

Luo, T.-H. & Zhou, H.-Z. (2012) Taxonomic study of the subgenus *Aleochara* (*s. str.*) Gravenhorst (Coleoptera: Staphylinidae: Aleocharinae) in China, with descriptions of four new species. *Annals of the Entomological Society of America*, **105**, 179–200.

Luo, T.-H. (2011) Taxonomy of Staphyliniform (Coleoptera) of China and Studies on Diversity of the Forensic Beetles. Institute of Zoology of Chinese Academay of Sciences, Beijing.

Luze, G. (1903) Revision der paläarktischen Arten der Staphyliniden-gattung *Lesteva* Latr. *Verhandlungen der k. k. zoologisch-botanischen Gesellschaft in Wien*, **53**, 179-197.

Luze, G. (1906) Revision der paläarktischen Arten der Staphyliniden-Genera: *Xylodromus*, *Omalium*, *Phyllodrepa*, *Hypopycna*, *Dialycera*, *Pycnoglypta* und *Phloeonomus*. *Verhandlungen der k. k. zoologisch-botanischen Gesellschaft in Wien*, **56**, 485-602.

Luze, G. (1910) Eine neue Art der Staphylinidengattung *Phyllodrepa* Thoms. *Verhandlungen der k. k. zoologischbotanischen Gesellschaft in Wien*, **6**, 394-395.

Lynch Arribálzaga, F. (1884) Los estafilinos de Buenos Aires. *Boletin de la Academia Nacional de Ciencias, Córdoba*, **7**, 5-392.

Makhan, D. & S. Ezzatpanah (2011) *Scydmaenus hawkeswoodi* sp. nov., an additional new ant-like stone beetle from central Java, Indonesia (Coleoptera, Scydmaenidae). *Calodema*, **154**, 1-3.

Makhan, D. & S. Ezzatpanah (2011) *Scydmaenus jacquelineae* sp. nov., an additional new ant-like stone beetle from central Java, Indonesia (Coleoptera, Scydmaenidae). *Calodema*, **153**, 1-3.

Makhan, D. & S. Ezzatpanah (2011) *Scydmaenus ramjiawani* sp. nov., an additional new ant-like stone beetle from central Java, Indonesia (Coleoptera, Scydmaenidae). *Calodema*, **165**, 1-3.

Makhan, D. & S. Ezzatpanah (2011) *Scydmaenus seayi* sp. nov., an new ant-like stone beetle from central Java, Indonesia (Coleoptera, Scydmaenidae). *Calodema*, **152**, 1-3.

Makhan, D. (1998) A new *Limnebius* and a new *Hydraena* for Turkey (Coleoptera: Hydraenidae). *Phegea*, **26**, 151-154.

Makhan, D. (2004) *Ochthebius amrishi* sp. nov. from central Java (Coleoptera: Hydraenidae). *Acta Biologica, Universitatis Daugavpiliensis*, **4**, 111-112.

Makhan, D. (2006) *Euconnus roepi* sp. nov., a new Scydmaenidae species from Langkawi Island, Malaysia (Coleoptera). *Calodema*, **7**, 6-7.

Makhan, D. (2008) *Hydraena nelsonmandelai* sp. nov., a new water beetle from Suriname (Coleoptera: Hydraenidae). *Calodema (Suppl.)*, **78**, 1-3.

Makranczy, Gy. (2006) Systematics and phylogenetic relationships of the genera in the *Carpelimus* group (Coleoptera: Staphylinidae: Oxytelinae). *Annales Historico-Naturales Musei Nationalis Hungarici*, **98**, 29-119.

Makranczy, Gy. (2011) Four new Neotropical species of *Anotylus* with an interesting sexual dimorphism (Coleoptera, Staphylinidae: Oxytelinae). *Annales Historico-Naturales Musei Nationalis Hungarici*, **103**, 43-64.

Makranczy, Gy. (2009) The genus *Thinodromus* Kraatz, 1857 in West-Central Africa (Coleoptera, Staphylinidae: Oxytelinae). *Annales Historico-Naturales Musei Nationalis Hungarici*, **101**, 33-61.

Makranczy, Gy. (2014) Review of the *Thinodromus circulus* species group (Coleoptera: Staphylinidae: Oxytelinae). *Acta Entomologica Musei Nationalis Pragae*, **54**, 539–554.

Makranczy, Gy. (2014) Revision of the genus *Ochthephilus* Mulsant & Rey, 1856 (Coleoptera: Staphylinidae, Oxytelinae). *Revue suisse de Zoologie*, **121**, 457-694.

Makranczy, Gy. (2014) Synonymies in the Holarctic *Thinobius major* and *linearis* species groups (Coleoptera: Staphylinidae: Oxytelinae). *Acta Zoologica Academiae Scientiarum Hungaricae*, **60**, 13-38.

Makranczy, Gy. (2014) The Far Eastern species of *Thinobius* Kiesenwetter, 1844 (Coleoptera: Staphylinidae, Oxytelinae) lacking female modified genital appendage. *Revue suisse de Zoologie*, **121**, 319-347.

Mann, W.M. (1926) New neotropical myrmecophiles. *Journal of the Washington Academy of Sciences*, **16**, 448-455.

Mannerheim, C. G. von (1846) Nachtrag zur Kaefer-fauna der aleutischen Inseln und der Insel Sitkha. *Bulletin de la Société Impériale des Naturalistes de Moscou*, **19**, 501-516.

Mannerheim, C. G. von. (1852) Zweiter Nachtrag zur Kaefer-Fauna der Nord-Amerikanischen Laender des Russischen Rieches. *Bulletin de la Société Impériale des Naturalistes de Moscou*, **25**, 283-387.

Märkel, F. (1845) Beiträge zur Kenntniss der unter Ameisen lebenden Insekten. In E. Germar's, Zeitschrift für die Entomologie, **5**, 193-271.

Márquez, J. & J. Asiain. (2016) Taxonomy of the Mexican species of *Thyreocephalus* Guérin-Méneville (Coleoptera: Staphylinidae, Xantholinini). *Zootaxa*, **4169**, 251-285.

Marsham, T. (1802) Coleoptera Britannica, sistens Insecta Coleoptera Britanniae indigena, secundum methodum Linnaeanam disposita. In two volumes. xxxi + 547 pp. + 1 unnumbered page. 30 plates. Londini: Publisher or printer not stated, sold by J. White.

Maruyama, M & M. Hayashi (2009) Description of the intertidal aleocharine *Halorhadinus sawadai* sp.n. from Japan, with notes on the genus *Halorhadinus* Sawada, 1971 (Coleoptera: Staphylinidae). *Koleopterologische Rundschau*, **79**, 71-82.

Maruyama, M. & T. Kishimoto (2002) Myrmecophilous species of *Drusilla* (Coleoptera, Staphylinidae, Aleocharinae) associated with *Lasius* (*Dendrolasius*) spp. (Hymenoptera, Formicidae, Formicinae) from China Part 2. *Elytra*, **30**, 111-118.

Maruyama, M. (2006) Revision of the Palearctic species of the myrmecophilous genus *Pella* (Coleoptera, Staphylinidae, Aleocharinae). *National Science Monograph*, **32**, 1-207.

Maruyama, M., and K.-J. Ahn (2000) Redescription of *Liparocephalus litoralis*, and key to the species of *Liparocephalus* (Coleoptera: Staphylinidae: Aleocharinae). *The Canadian Entomologist*, **132**, 567-571.

Maruyama, M., S. H. Yek, R. Hashim & F. Ito (2003) A new myrmecophilous species of *Drusilla* (Coleoptera, Staphylinidae, Aleocharinae) from Peninsular Malaysia, a possible Batesian mimic associated with *Crematogaster inflata* (Hymenoptera, Formicidae, Myrmicinae). *The Japanese Journal of Systematic Entomology*, **9**, 267-275.

Maruyama, M., S. Yamamoto & K. T. Eldredge. (2014) Synopsis of the Japanese species of Aleocharinae (Coleoptera: Staphylinidae), with review of the type specimens I. Tribes Himalusini and Leucocraspedini. *Zootaxa*, **3887**, 393-400.

Masch, R. & M. Uhlig. (1990) Revision der äthiopischen *Erichsonius robustus*-Gruppe (Col. Staphylinidae). *Deutsche Entomologische Zeitschrift, (Neue Folge)*, **37**, 231–278.

Mathis, K. A. & K. T. Eldredge. (2014) Descriptions of two new species of *Myrmedonota* Cameron (Staphylinidae: Aleocharinae) from Mexico with comments on the genus taxonomy and behavior. *Zootaxa*, **3768**, 95-100.

Meybohm, H. & S. Vít (2005) On *Euconnus* (*Napochus*) campestris Schaufuss and its synonymy (Coleoptera: Scydmaenidae). *Zootaxa*, **1086**, 37-46.

Meybohm, H. (2009) *Neuraphes* (*Pararaphes*) *planiceps* Reitter und verwandte Arten vom Südrand der Alpen (Coleoptera, Scydmaenidae). *Entomologische Blätter*, **105**, 61-72.

Motschulsky, V. (1862) Entomologie spéciale. Insectes du Japon. *Etudes Entomologiques*, **9**, 4-39.

Mroczkowski, M. (1959) *Nicrophorus* (*Nicrophorus*) *kieticus* sp. n. from the Solomon Islands (Coleoptera, Silphidae). *Annales Zoologici Warszawa*, **18**, 65-69.

Mulsant, E & C. Rey (1875) Tribu de brévipennes. Famille des aléochariens. Suite. Sixième branche - Aléocharaires (suite). *Annales de la Société Linnéenne de Lyon (N.S.)*, **21**, 1-403.

Mulsant, E. & C. Rey (1861) Description de quelques coléoptères nouveaux ou peu connus. *Opuscules Entomologiques*, **12**, 139-188.

Mulsant, E. and C. Rey (1876) Tribu des brévipennes. [Staphyliniens]. *Annales de la Société d'Agriculture Histoire Naturelle et Arts utiles de Lyon*, (4)**8**, 145-856.

Muona, J. (1990) The Fennoscandian and Danish species of the genus *Amischa* Thomson (Coleoptera, Staphylindiae). *Entomologisk Tidskrift*, **111**, 17-24.

Muona, J. (1993) A new *Acrotona* species from Europe (Coleoptera, Staphylinidae). *Entomologische Blätter*, Krefeld, **89**, 9-14.

Naomi, S.-I. (1983) Revision of the subtribe Xanthopygina (Coleoptera, Staphylinidae) of Japan. II. *Kontyû*, **51**, 47-55.

Naomi, S.-I. (1986) Taxonomic study on the genus *Velleius* Mannerheim of Japan, with description of a new species (Coleoptera: Staphylinidae). *Transactions of the Shikoku Entomological Society*, **17**, 239-246.

Naomi, S.-I. (1994) Revision of the *debile* group of the genus *Siagonium* Kirby et Spence from Japan (Coleoptera, Staphylinidae, Piestinae). *The Entomological Review of Japsan*, **49**, 145-154.

Naomi, S.-I. (1995) Description of a new species of the genus *Trichophya* (Coleoptera, Staphylinidae) from Japan. In Y. Watanabe, M. Satô, and M. Owada (eds.), Beetles and Nature. *Special Bulletin of the Japanese Society of Coleopterology*, Tokyo, **4**, 347-350.

Naomi, S.-I. (1995) Revision of the subfamily Piestinae (Coleoptera: Staphylinidae) from Japan, I. *Natural History Research*, **3**, 141-151.

Naomi, S.-I. (1996) Revision of the genera *Lispinus* Erichson and *Neolosus* Blackwelder (Coleoptera: Staphylinidae) from Japan. *Japanese Journal of Entomology*, **64**, 762-771.

Naomi, S.-I. (1996) Two new species of the family Staphylinidae (Coleoptera) from Japan. *New Entomologist*, **45**, 69-73.

Naomi, S.-I. (1997) A revision of the genus *Nacaeus* Blackwelder (Coleoptera: Staphylinidae) from Japan. *Japanese Journal of Entomology*, **65**, 127-142.

Naomi, S.-I. (2006) Description of a new species of the genus *Siagonium* Kirby et Spence from Japan (Coleoptera, Staphylinidae, Piestinae). *Natural History Research*, **9**, 41-44.

Navarrete-Heredia, J. L. & J. Márquez-Luna (1998) A new Mexican species of *Gastrisus* (Coleoptera: Staphylinidae). *Entomological News*, **109**, 225-232.

Neuhäuser-Happe, L. (1999) Eine neue *Bryaxis*-Art aus der nordwestlichen Türkei: *Bryaxis schusteri* n. sp. (Coleoptera, Staphylinidae, Pselaphinae). *Entomologische Blätter*, **95**, 171-174.

Newton, A. F. (1982) A new genus and species of Oxytelinae from Australia, with a description of its larva, systematic position, and phylogenetic relationships (Coleoptera, Staphylinidae). *American Museum Novitates*, **2744**, 1-24.

Nikolaev, G. V. & V. O. Kozminykh (2002) The carrion beetles (Coleoptera: Agyrtidae, Silphidae) of Kazakhstan, Russia and adjacent countries. Almaty: Kazak universiteti, 160 pp (in Russian, with English summary).

Nishikawa, M (1995) New cave-dwelling *Catops* (Coleoptera, Cholevidae) from the Abukuma Hills, central Japan. *Elytra*, **23**, 71-75.

Nishikawa, M (1997) A new *Catops* (Coleoptera, Cholevidae) of the hilleri group from Japan. *Elytra*, **25**, 117-121.

Nishikawa, M. & G. Sh. Lafer (2000) Notes on *Catops lydiae* Iablokoff-Khnzorian (Coleoptera, Cholevidae) from the Russian Far East and north Japan. *Elytra*, **28**, 79-85.

Nishikawa, M. & Y. B. Cho (2000) Three new *Catops* (Coleoptera, Leiodidae) from South Korea, with a preliminary check-list of the subfamily Cholevinae known from Korea. *Elytra*, **28**, 87-99.

Nonveiller, G., D. Pavićević & C. Besuchet (2003) *Bryaxis tuberculiceps* sp. n., Psélaphide cavernicole du Monténégro (Coleoptera Staphylinidae Pselaphinae). *Mitteilungen der Schweizerischen Entomologischen Gesellschaft*, **76**, 287-291.

O'Keefe, S. T. (1997) *Euconnus longiceps* Fall, an odd Ant-like Stone Beetle from the Pacific northwest (Coleoptera: Scydmaenidae). *Coleopterists Bulletin*, **51**, 277-283.

Ordish, R. G. (1984) Hydraenidae (Insecta: Coleoptera). Fauna of New Zealand, 6. Wellington: Science Information Publishing Centre, DSIR, 56.

Orousset, J. (1989) Coléoptères hypogés de Corse. XXIV. Les genres *Phloeocharis*, Typhlocyptus et Platyola (Col. Staphylinidae). *Bulletin de la Société entomologique de France*, **94**, 35-46.

Orth, R. E., and I. Moore (1980) A revision of the species of *Cafius* Curtis from the west coast of North America with notes of [*sic*] the east coast species (Coleoptera: Staphylinidae). *Transactions of the San Diego Society of Natural History*, **19**, 181-211.

Outerelo, R. (1984) *Phloeocharis* (*Scotodytes*) *vivesi* n.sp. de Los *Pirineos* Españoles. (Col. Staphylinidae: Phloeocharinae). *Pirineos*, **122**, 5-9.

Pace, R. (1978) *Bryaxis kahleni*, nuova specie del Trentino (Coleoptera, Pselaphidae). *Studi Trentini di Scienze Naturali (Acta Biologica)*, **55**, 3-236.

Pace, R. (1980) Le *Leptusa* del gruppo *schaschli* Gangl. e note su *L. major* Bernh. (XIV Contributo alla conoscenza delle Aleocharinae) (Coleoptera Staphylinidae). *Atti della Societa italiana di Scienze naturali e del Museo civico di Storia naturale di Milano*, **121**, 47-60.

Pace, R. (1983) Risultati delle studio delle specie del genere *Leptusa* Kraatz della collezione Scheerpeltz al Naturhistorisches Museum di Vienna. (Coleoptera, Staphylinidae) (XXII Contributo alla conoscenza delle Aleocharinae). *Annalen des Naturhistorischen Museums in Wien*, **85**, 53-102.

Pace, R. (1984) Aleocharinae delle Mascarene, parte I: tribù Myllaenini, Pronomaeini, Oligotini e Bolitocharini (Coleoptera Staphylinidae) (XLV Contributo alla conoscenza delle Aleocharinae). *Revue suisse de Zoologie*, **91**, 3-36.

Pace, R. (1987) Aleocharinae del Cile (Coleoptera Staphylinidae) (XCVII Contributo alla conoscenza delle Aleocharinae). *Redia*, **70**, 459-522.

Pace, R. (1987) Staphylinidae dell'Himalaya Nepalese Aleocharinae raccolte dal Prof. Dr. J. Martens (Insecta: Coleoptera). *Courier Forschungsinstitut Senckenberg*, **93**, 383-441.

Pace, R. (1994) Aleocharinae della Sottoregione Africana Orientale al Museo di Ginevra (Coleoptera, Staphylinidae). Parte I. (117° Contributo alla conoscenza delle Aleocharinae.) *Revue suisse de Zoologie*, **100**, 117-193.

Pace, R. (1996) Aleocharinae del Burundi al Museo di Erfurt (Coleoptera, Staphylinidae) (128° Contributo alla conoscenza delle Aleocharinae). *Bollettino della Società Entomologica Italiana*, **127**, 231-242.

Pace, R. (1996) Coleoptera. Staphylinidae. Leptotyphlinae. In Fauna d'Italia 34: i-viii + 1-328. Bologna: Calderini.

Pace, R. (1998) Aleocharinae della Cina: Parte I (Coleoptera, Staphylinidae). *Revue suisse de Zoologie*, **105**, 139-220.

Pace, R. (1998) Aleocharinae della Cina: Parte II (Coleoptera, Staphylinidae). *Revue suisse de Zoologie*, **105**, 395-463.

Pace, R. (1998) Aleocharinae della Cina: Parte III (Coleoptera, Staphylinidae). *Revue suisse de Zoologie*, **105**, 665-732.

Pace, R. (1998) Aleocharinae della Cina: Parte IV (Coleoptera, Staphylinidae). *Revue suisse de Zoologie*, **105**, 911-982.

Pace, R. (1999) Aleocharinae della Cina: Parte V (conclusione) (Coleoptera, Staphylinidae). *Revue suisse de Zoologie*, **106**, 107-164.

Pace, R. (1999) Nuove specie del genere *Leptusa* Kraatz raccolte da Manfred Kahlen. *Veröffentlichungen des Tiroler Landesmuseums Ferdinandeum*, **79**, 207-214.

Pace, R. (2002) Specie dei generi *Eusteniamorpha* Cameron e *Autalia* Leach del Borneo (Coleoptera, Staphylindae). *Revue suisse de Zoologie*, **109**, 295-323.

Pace, R. (2004) Aleocharinae della Cina all'Institut royal des Sciences naturelles de Belgique (Coleoptera, Staphylinidae). *Belgian Journal of Entomology*, **6**, 353-361.

Pace, R. (2007) Biodiversità delle *Leptusa* della Cina, Monografia del genere *Leptusa* Kraatz. Supplemento XVI (Coleoptera, Staphylinidae, Aleocharinae). *Beiträge zur Entomologie*, **60**, 295-299.

Pace, R. (2007) Description of *Amischa paolettii* sp. nov. and Thamiaraea tsitsilasi sp. nov. from the Australian region (Coleoptera, Staphylinidae). *Memoirs of Museum Victoria*, **64**, 7-11.

Pace, R. (2007) Le specie dei generi *Gyrophaena* Mann. e *Brachida* Muls. & Rey di Taiwan (Coleoptera, Staphylinidae). *Bollettino del Museo Civico di Storia Naturale di Verona*, **31**, 103-129.

Pace, R. (2007) Le specie di Oligotini, Leucocraspedini, Hygronomini, Placusini, Bolitocharini e Diestotini nel Borneo (Coleoptera, Staphylinidae). *Revue suisse de Zoologie*, **114**, 771-815.

Pace, R. (2007) Nuovi dati e nuove specie del genere *Leptusa* Kraatz di Taiwan. Monografia del genere *Leptusa* Kr. Supplemento XIV (Coleoptera, Staphylinidae). *Bollettino del Museo Civico di Storia Naturale di Verona*, **31**, 141-145.

Pace, R. (2008) New records of Aleocharinae from Ecuador and Peru, with the description of new species, new subgenera and new genera (Coleoptera, Staphylinidae). Pp. 225-398 in: Giachino, P.M. (ed) Biodiversity of South America. I. Memoirs on biodiversity. 1. World Biodiversity Association onlus, Verona, Italy.

Pace, R. (2009) Aleocharinae dell'Irian Jaya (Nuova Guinea) al Naturkundemuseum di Erfurt (Insecta: Coleoptera: Staphylinidae). (230° Contributo alla conoscenza delle Aleocharinae). *Veröffentlichungen des Naturkundemuseums Erfurt-Vernate*, **28**, 259-329.

Pace, R. (2012) New data and new species of Aleocharinae from Tropical Africa in the Natural History Museum of the Humboldt University, Berlin (Coleoptera, Staphylinidae). *Linzer biologische Beiträge*, **44**, 1331-1362.

Pace, R., (2010) Biodiversita delle Aleocharinae della Cina: Hypocyphtini, Leucocraspedini e Pronomaeini (Coleoptera, Staphylinidae). *Beiträge zur Entomologie*, **60**, 81-105.

Palm, T. (1948) Svensk Insekfauna. 9. Skalbaggar. Coleoptera. Kortvingar: Fam. Staphylinidae. Unterfam. Micropeplinae, Phloeocharinae, Olisthaerinae, Proteininae, Omaliinae. 133 pp. Uppsala: Almquist and Wiksells.

Palm, T. (1966) De svenska *Gnypeta*-arterna (Col. Staphylinidea, Aleocharinae). *Entomologisk Tidskrift*, **87**, 136-141.

Pandellé, L. (1867) Synopsis des *Oxytelus* Français du groupe du depressus. In A. Grenier, Matériaux pour servir a la faune des Coléoptères France, receuillis et publiés. pp. 170-173. Paris: A. Grenier.

Pandellé, L. (1869) Études monographique sur les staphylins européens de la tribu des Tachyporini Erichson. *Annalesde la Société Entomologique de France*, (4)**9**, 261-366.

Park, J.-S. & K.-J. Ahn (2009) Synonymy of *Aleochara* (*Aleochara*) claviger and A. niponensis (Coleoptera: Staphylinidae: Aleocharinae), and first record of A. claviger in Korea. *Florida Entomologist*, **92**, 521-523.

Park, J.-S. & K.-J. Ahn (2010) Korean species of *Aleochara* Gravenhorst subgenus *Xenochara* Mulsant & Rey (Coleoptera, Staphylinidae, Aleocharinae). *Zookeys*, **60**, 21-36.

Park, J.-S. & K.-J. Ahn (2010) Redescription of *Aleochara* (*Aleochara*) *kochi* Bernhauer with a lectotype designation and key to species of the subgenus *Aleochara* (Coleoptera: Staphylinidae: Aleocharinae) from the Far East. *Journal of the Kansas Entomological Society*, **83**, 313-317.

Park, J.-S., and K.-J. Ahn (2005) *Autalia* villosa, a new species from Korea, and an annotated catalog of the genus *Autalia* Leach (Coleoptera: Staphylinidae: Aleocharinae). *Zootaxa*, **1043**, 1-16.

Park, S.-J. & K.-J. Ahn (2007) The Korean species of the genus *Leiodes* Latreille with descriptions of five new species (Coleoptera: Leiodidae: Leiodinae). *Journal of the Kansas Entomoligical Society*, **80**, 27-42.

Park, S.-J., H. Hoshina, K.-J. Ahn (2005) Descriptions of two new species of the genus *Colon* Herbst (Coleoptera: Leiodidae: *Colon*inae) from Korea and Japan. *Coleopterists Bulletin*, **59**, 407-413.

Paśnik, G. (2001) The North Korean Aleocharinae (Coleoptera, Staphylinidae): diversity and biogeography. *Acta zoologica cracoviensia*, **44**, 185-234.

Paśnik, G. (2002) Four new species of the genus *Gnypeta* Thomson, 1858 from the Oriental Region (Coleoptera, Staphylinidae, Aleocharinae). *Revue suisse de Zoologie*, **109**, 705-713.

Paśnik, G. (2005) A new species of the genus *Gnypeta* Thomson from the Eastern Palearctic Region (Coleoptera, Staphylinidae, Aleocharinae). *Linzer biologische Beiträge*, **37**, 729-731.

Paśnik, G. (2005) Five new species of the genus *Gnypeta* Thomson from the Australian and Oriental Regions (Coleoptera, Staphylinidae, Aleocharinae). *Linzer biologische Beiträge*, **37**, 707-715.

Paśnik, G. (2007) A taxonomic review of *Stenectinobregma* SCHEERPELTZ, a South African genus of Aleocharinae (Coleoptera, Staphylinidae), with descriptions of two new species. *Linzer biologische Beiträge*, **39**, 1111-1120.

Peck, S. B. & J. Cook (2007) Systematics, distributions, and bionomics of the *Neoeocatops* gen. nov. and *Nemadus* of North America (Coleoptera: Leiodidae: Cholevinae: Anemadini). *The Canadian Entomologist*, **139**, 87-117.

Peck, S. B. & J. Cook (2014) A review of the small carrion beetles and the round fungus beetles of the West Indies (Coleoptera: Leiodidae), with descriptions of two new genera and 61 new species. *Insecta Mundi*, **0397**, 1-76.

Peck, S. B. & P. Gnaspini (1997) *Ptomaphagus inyoensis* n.sp., a new microphthalmic montane beetle from California (Coleoptera; Leiodidae; Cholevinae; Ptomaphagini). *The Canadian Entomologist*, **129**, 769-776.

Peck, S. B. & P. Gnaspini (1997) Review of the myrmecophilous *Ptomaphagus*, subgenus Echinocoleus (new status), of north America (Coleoptera: Leiodidae: Cholevinae: Ptomaphagini). *The Canadian Entomologist*, **129**, 93-104.

Peck, S. B. & R. S. Anderson (1985) Taxonomy, phylogeny and biogeography of the carrion beetles of Latin America (Coleoptera: Silphidae). *Quaestiones Entomologicae*, **21**, 247-317.

Peck, S. B. (1973) A systematic revision and the evolutionary biology of the *Ptomaphagus* (*Adelops*) beetles of North America (Coleoptera; Leiodidae; Catopinae), with emphasis on cave-inhabiting species. *Bulletin of the Museum of Comparative Zoology*, **145**, 29-162.

Perkins, P. D. & J. Balfour-Browne (1994) A contribution to the taxonomy of aquatic and humicolous beetles of the family Hydraenidae in Southern Africa. *Fieldiana: Zoology (N. S.)*, **77**, 1-159.

Perkins, P. D. (2007) A review of the coastal marsh water beetle *Ochthebius queenslandicus* Hansen (Coleoptera: Hydraenidae). *Zootaxa*, **1625**, 35-42.

Perkins, P. D. (2014) A revision of the water beetle genus *Hydraena* Kugelann for southern Africa (Coleoptera: Hydraenidae). *Zootaxa*, **3758**, 1-92.

Peyerimhoff, P. de (1913) Nouveaux Coléoptères du Nord-Africain (seizième note, faune du Djurdjura). *Bulletin de la Société Entomologique de France*, **1913**, 253-255.

Peyerimhoff, P. de (1938) Notes sur les *Amischa* Thomson. *Revue Francaise d'Entomologie*, **5**, 64-73.

Peyron, E. (1858) Catalogue des Coléoptères des environs de Tarsous (Caramanie), avec la description des espèces nouvelles. *Annales de la Société Entomologique de France*, (3)**6**, 353-434.

Pic, M. (1926) Nouveaux Coléoptères du Tonkin [2e article] (2). *Bulletin de la Société Zoologique de France*, **51**, 45-48.

Pic, M. (1954) Coléoptères nouveaux de Chine. *Bulletin de la Société Entomologique de Mulhouse*, **1954**, 53-59.

Polilov, A. A. (2008) An introduction to the Ptiliidae (Coleoptera) of Primorskiy region with descriptions of three new genera, new and little known species. *Russian Entomological Journal*, **17**, 149-176.

Pushkin, S. V. (2006) Review of the *Thanatophilus* Leach, 1815 (Coleoptera: Silphidae) of Southern of Russia. *Caucasian Entomological Bulletin [Кавказский энтомологический бюллетень]*, **2**, 41-46.

Puthz, V. (1968) Die *Stenus*- und *Megalopinus*-Arten Motschulskys und Bemerkungen über das Subgenus Tesnus Rey, mit einer Tabelle der paläarktischen Vertreter (Coleoptera, Staphylinidae). *Notulae Entomologicae*, **48**, 197-219.

Puthz, V. (1968) Über einige europäische *Stenus*-Endemiten (Coleoptera, Staphylinidae). *Entomologische Blätter für Biologie und Systematik der Käfer*, **64**, 86-92.

Puthz, V. (1970) Noch ein neuer *Stenus* von den Kanarischen Inseln (Coleoptera, Staphylinidae). *Koleopterologische Rundschau*, **48**, 75-77.

Puthz, V. (1970) *Stenus* (*s. str.*) *anatolicus* nov. spec. aus der Türkei. *Mitteilungen der Deutschen Entomologischen Gesellschaft*, **29**, 22-24.

Puthz, V. (1971) Revision der afrikanischen Steninenfauna und Allgemeines über die Gattung *Stenus* Latreille (Coleoptera, Staphylinidae). *Annales Musee Royal de l'Afrique Centrale, Tervuren (8) Sciences Zoologiques*, **187**, 1-376.

Puthz, V. (1974) Bemerkungen über die Gattung *Edaphus* Motschulsky und über Kistnersche *Edaphus*-Arten nebst neuen und alten afrikanischen Euaesthetinen (Coleoptera, Staphylinidae). *Revue de Zoologie et de Botanique Africaines*, **88**, 749-770.

Puthz, V. (1975) Revision der paläarktischen *Edaphus*-species (Coleoptera: Staphylinidae). *Entomologica Germanica*, **1**, 170-184.

Puthz, V. (1977) Neue Bemerkungen über afrikanische *Megalopinus*-Arten (Coleoptera: Staphylinidae). *Folia Entomologica Hungarica*, **30**, 129-132.

Puthz, V. (1980) Beiträge zur Kenntnis der Steninen. CLXXVI. Neue *Dianous*-Arten (Staphylinidae, Coleoptera). *Philippia*, **4**, 234-240.

Puthz, V. (1980) Die gelblich gemakelten *Dianous*-Arten der Welt: Bestimmungstabelle und Neubeschreibungen. *Reichenbachia*, **18**, 1-11.

Puthz, V. (1981) Neue westpaläarktische *Stenus*, vorwiegend aus dem Genfer Museum (Coleoptera, Staphylinidae). *Revue suisse de Zoologie*, **88**, 693-706.

Puthz, V. (1981) Was ist *Dianous* Leach, 1819, was ist *Stenus* Latreille, 1796? oder Aie Aporie des Stenologen und ihre taxonomischen Konsequenzen (Coleoptera, Staphylinidae). *Entomologische Abhandlungen*, **44**, 87-132.

Puthz, V. (1983) Beiträge zur Kenntnis der Euaesthetinen. XXXVIII. Über einige *Edaphus*-Arten aus China (Staphylinidae, Coleoptera). *Philippia*, **5**, 163-165.

Puthz, V. (1984) Euaesthetinae aus Ghana II. (Coleoptera: Staphylinidae). *Folia Entomologica Hungarica*, **45**, 195-203.

Puthz, V. (1990) Beiträge zur Kenntnis der Megalopinien XVII. Drei neue orientalische *Megalopinus*-Arten (Staphylinidae, Coleoptera). *Philippia*, **6**, 265-269.

Puthz, V. (1990) *Edaphus*-Arten von der Elfenbeinküste (Coleoptera, Staphylinidae) 60. Beitrag zur Kenntnis der Euaesthetinen. *Revue suisse de Zoologie*, **97**, 195-222.

Puthz, V. (1990) Fünf neue orientalische *Dianous*-Arten (Insecta, Coleoptera, Staphylinidae: Steninae). *Reichenbachia*, **27**, 115-125.

Puthz, V. (1992) Zwei neue *Megalopinus*-Arten aus Madagaskar (Coleoptera, Staphylinidae). *Nouvelle Revue d'Entomologie*, (n. ser.), **8**, 417-421.

Puthz, V. (1994) Beiträge zur Kenntnis der Megalopsidiinen. XX. Über neue und alte neotropische *Megalopinus*Arten 2 (Staphylinidae, Coleoptera). *Philippia*, **6**, 421-467.

Puthz, V. (1997) Beiträge zur Kenntnis der Steninae. CCLII. Neue Arten der Gattung *Dianous* Leach. *Philippia*, **8**, 93-116.

Puthz, V. (1997) *Megalopinus heissi* sp. n. von Madagaskar (Coleoptera, Staphylinidae). *Entomologische Nachrichtenblatt*, **4**, 7-8.

Puthz, V. (1999) *Megalopinus wagneri* sp. n. aus Uganda (Coleoptera, Staphylinidae). *Zeitschrift der Arbeitsgemeinschaft Österreichischer Entomologen*, **51**, 21-24.

Puthz, V. (2001) Neotropical *Euaesthetus* Gravenhorst (Coleoptera: Staphylinidae) (82th Contribution to the knowledge of Euaesthetinae). *Dugesiana*, **8**, 29-36.

Puthz, V. (2005) Über zwei *Euaesthetus*-Arten aus China (Coleoptera, Staphylinidae) 89. Beitrag zur Kenntnis der Euaesthetinen. *Entomologische Blätter*, **101**, 167-170.

Puthz, V. (2007) New Neotropical *Edaphus*-species (Coleoptera: Staphylinidae). *Amazoniana*, **19**, 97-130.

Puthz, V. (2008) Neue *Edaphus* aus Sri Lanka (Coleoptera: Staphylinidae) - 97. Beitrag zur Kenntnis der Euaesthetinen. *Revue suisse de Zoologie*, **115**, 325-330.

Puthz, V. (2008) The first Euaesthetines from the Solomon Islands (Coleoptera: Staphylinidae). *Mitteilungen des Internationalen Entomologischen Vereins E.V. Frankfurt A.M.*, **33**, 1-5.

Puthz, V. (2010) A new species of the genus *Euaesthetus* Gravenhorst (Coleoptera: Staphylinidae) from Japan (102nd Contribution to the Knowledge of Euaesthetinae). *Entomological Review of Japan*, **65**, 11-14.

Puthz, V. (2010) Der erste *Megalopinus* Eichelbaum von Taiwan (Coleoptera, Staphylinidae). *Mitteilungen des Internationalen Entomologischen Vereins*, **35**, 155-158.

Qubaiová, J., J. Růžička & H. Šípková (2015) Taxonomic revision of genus *Ablattaria* Reitter (Coleoptera, Silphidae) using geometric morphometrics. *Zookeys*, **477**, 79-142.

Quedenfeldt, G. (1882) Diagnosen neuer Staphylinen aus dem Mittelmeer-Faunengebiet. *Berliner Entomologische Zeitschrift*, **26**, 181-183.

Rambousek, F. J. (1908) *Euplectus* Jurečeki m., nový Pselaphid z okolí pražského. *Časopis České Společnosti Entomologické*, **2**, 105-107.

Ratcliffe, B. C. & M. L. Jameson (1992) New nebraska occurrences of the endangered American burying beetle (Coleoptera: Silphidae). *Coleopterists Bulletin*, **46**, 421-425.

Reitter, E. (1877) Coleopterologische Ergebnisse einer Reise nach Südungarn und in die Transsylvanische Alpen. *Verhandlungen des Naturforschendes Vereins in Brünn*, **15**, 3-30.

Reitter, E. (1880) Beitrag zur Kenntniss europäischer Pselaphidae und Scydmaenidae. *Verhandlungen der k. k. zoologisch-botanischen Gesellschaft in Wien*, **29**, 533-542.

Reitter, E. (1882) Bestimmungs-Tabellen der europäischen Coleopteren. V. Enthaltend die Familien: Paussidae, Clavigeridae, Pselaphidae und Scydmaenidae. *Verhandlungen der Kaiserlich-Königlichen Zoologisch-Botanischen Gesellschaft, Wien*, **31**, 443-593.

Reitter, E. (1882) Clavigeridae, Pselaphidae, Scydmaenidae [Erste Lieferung, pp. 1-198]. In: Naturgeschichte der Insecten Deutschlands. Erste Abtheilung. Coleoptera. Dritter Band. Zweite Abtheilung. Berlin: Nicolaische Verlags-Buchhandlung, iv+362 pp.

Reitter, E. (1902) Neue ung seltene Coleopteren, gesammelt im Jahre 1901, in der Herzegowina, in Dalmatien und Bosnien. *Wiener Entomologische Zeitung*, **21**, 1-9.

Reitter, E. (1909) Fauna Germanica. Die Käfer des Deutschen Reiche. Nach der analytischen Methode bearbeitet. 2: 1-392. Stuttgart: K. G. Lutz.

Reitter, E. (1909) Fauna Germanica. Die Käfer des Deutschen Reiches. Nach der analytischen Methode bearbeitet. II Band. Schriften des Deutschen Lehrervereins für Naturkunde. 24. Stuttgart: K. G. Lutz, 392 +xl pp.

Ribera, I. & A. Millán (1998) *Limnebius aguilerai* sp. nov. from south Morocco (Coleoptera: Hydraenidae). *Entomological Problems*, **29**, 109-110.

Ribera, I. & C. Hernando (1998) Description of *Limnebius millani* sp.n. (Insecta: Coleoptera: Hydraenidae) from the Sierra de Alcaraz (Southeast Spain). *Annalen des Naturhistorischen Museums in Wien*, **100**, 199-202.

Ribera, I., A. Castro, and C. Hernando (2010) *Ochthebius* (*Enicocerus*) *aguilerai* sp. n. from central Spain, with a molecular phylogeny of the Western Palaearctic species of *Enicocerus* (Coleoptera, Hydraenidae). *Zootaxa*, **2351**, 1-13.

Rosskothen, P. (1934) Ptilium Horioni und Ptiliolum Wüsthoffi zwei neue deutsche Ptiliiden. *Entomologische Blätter*, **30**, 200-202.

Rottenberg, A. (1870) Beiträge zur Coleopteren-Fauna von Sicilien. *Berliner Entomologische Zeitschrift*, **14**, 11-40.

Rottenberg, A. von. (1871) Mastigus Heydenii nov. spec. *Berliner Entomologische Zeitschrift*, **14**, 233-234.

Rougemont, G. M. de (2001) Description of a new species of *Apteroloma* from China (Coleoptera, Agyrtidae). *Revue suisse de Zoologie*, **108**, 351-353.

Rougemont, G.-M. de (1983) *Dianous martensi* n. sp. from Nepal. Insecta: Coleoptera: Staphylinidae). *Senckenbergiana biologica*, **63**, 363-366.

Rougemont, G.-M. de (1985) In the footsteps of H. G. Champion: New *Dianous* species from the Himalaya (Coleoptera, Staphylinidae). *Entomologica Basiliensia*, **10**, 123-144.

Růžička, J. & J. Schneider (1995) A new species of *Apteroloma* from the Far East, and new faunistic records on Palaearctic Agyrtidae (Coleoptera). *Entomological Problems*, **26**, 111-115.

Růžička, J. & J. Vávra (2003) A revision of the *Choleva agilis* species group (Coleoptera, Leiodidae, Cholevinae). In: G. Cuccodoro & R. A. B. Leschen (eds.), Systematics of Coleoptera: papers celebrating the retirement of Ivan Löbl -- Memoirs on Entomology International, **17**, 141-255.

Růžička, J. & M. Perreau (2011) A revision of the Chinese *Catops* Paykull 1798 of the *Catops fuscus* species group (Coleoptera: Leiodidae: Cholevinae). *Annales de la Société Entomologique de France, (N.S.)*, **47**, 280-292. DOI: 10.1080/00379271.2011.10697721

Růžička, J. (1993) A new species of *Catops* and notes on Cholevinae (Coleoptera, Leiodidae) from Bulgaria. *Klapalekiana*, **31**, 121-129.

Růžička, J. (1993) *Catops* nikodymi sp. n. and notes on Cholevinae from China (Coleoptera, Leiodidae). *Acta Societatis Zoologicae Bohemoslovacae*, **56**, 279-284.

Růžička, J. (1993) Three new species of *Choleva* (Coleoptera: Leiodidae: Cholevinae) from the Caucasus and Turkey, with a key to species of the *cisteloides* group. *European Journal of Entomology*, **90**, 337-348.

Růžička, J., and A. Pütz (2009) New species and new records of Agyrtidae (Coleoptera) from China, India, Myanmar, Thailand and Vietnam. *Acta Entomologica Musei Natioalis Pragae*, **49**, 631-650.

Růžička, J., and J. Schneider (2011) Revision of Palaearctic and Oriental *Necrophila* Kirby & Spence, part 1: subgenus *Deutosilpha* Portevin (Coleoptera: Silphidae). *Zootaxa*, **2987**, 1-12.

Růžička, J., J. Háva & J. Schneider (2000) Taxonomical and distributional notes on Oriental Silphidae, with description of *Nicrophorus sausai* sp. n. (Insecta: Coleoptera). *Reichenbachia*, **33**, 377-384.

Růžička, J., J. Háva & J. Schneider (2004) Revision of Palaearctic and Oriental *Oiceoptoma* (Coleoptera: Silphidae). *Acta Societatis Zoologicae Bohemicae*, **68**, 30-51.

Růžička, J., J. Schneider & J. Háva (2004) A revision of *Apteroloma* (Coleoptera: Agyrtidae) from China. *Acta Societatis Zoologicae Bohemicae*, **68**, 113-126.

Růžička, J., J. Schneider, J. Qubaiová, and M. Nishikawa (2012) Revision of Palaearctic and Oriental *Necrophila* Kirby & Spence, part 2: subgenus *Chrysosilpha* Portevin (Coleoptera: Silphidae). *Zootaxa*, **3261**, 33-58.

Růžička, J., N. Jansson & M. Coskun (2006) Complementary description of *Catops hanusi* (Coleoptera: Leiodidae: Cholevinae), with notes on its bionomy and occurrence in Turkey. *Entomological Problems*, **36**, 43-46.

Ryabukhin, A. S. (1992) A new species of the genus *Syntomium* (Coleoptera, Staphylinidae, Oxytelinae) from the North-east of Asia [Новый вид рода *Syntomium* (Coleoptera, Staphylinidae, Oxytelinae) с северо-востока азии]. *Zoologicheskii Zhurnal [Зоологический журнал]*, **71**, 147-149.

Ryabukhin, A. S. (2007) First record of the genus *Rugilus* Leach, 1819 (Coleoptera: Staphylinidae: Paederinae) in the North-east Asia with a description of a new species. *Far Easten Entomologist*, **172**, 1-4.

Sabella, G. (1989) Ricerche sugli Pselaphidae di Sicilia III. Su due nuovi interessanti pselafidi di Sicilia e considerazioni su alcune specie del genere *Bryaxis* (Coleoptera Pselaphidae). *Revue suisse de Zoologie*, **96**, 433-444.

Sáinz-Cantero, C. E. & N. Bennas (2006) *Limnebius kamali* sp. n. from Northern Morocco (Coleoptera, Hydraenidae). *Revue suisse de Zoologie*, **113**, 559-563.

Saiz, F. (1970) Nuevo *Megalopinus* Eich. (*Megalopdidia* Leng.) para Chile (Coleoptera, Staphylinidae). *Noticiario Mensual del Museo Nacional de Historia Natural*, **25**, 3-9.

Saiz, F. (1972) Nuevos Euaesthetinae de Chile (Col. Staphylinidae). *Anales del Museo de Historia Natural de Valparaiso*, **5**, 173-187.

Saiz, F. (1974) Revision de la subfamilia Leptotyphlinae (Col. Staph) en Chile, con notas sobre su ecologia y su biogeographia. *Revista Chilena de Entomología*, **8**, 47-66.

Saiz, F. (1975) Nuevos Leptotyphlinae de Chile (Col. Staphylinidae). Importancia biogeográfica. *Revista Chilena de Entomología*, **9**, 7-10.

Saiz, F. (1975) Une nouvelle espèce de Leptotyphlinae de Californie (U.S.A.) (Coleopt. Staphylinidae). *Nouvelle Revue d'Entomologie*, **5**, 43-45.

Salgado, J. M., M. Blas & J. Fresneda (2004) Nuevos datos sobre el género *Choleva* Latreille, 1796 en la Península Ibérica con la descripción de una nueva especie (Coleoptera: Cholevidae). *Elytron*, **17-18**, 47-71.

Sanderson, M. W. (1946) A New Genus of Nearctic Staphylinidae (Coleoptera). *Journal of the Kansas Entomological Society*, **19**, 130-133.

Santiago-Jiménez, Q. J. (2014) Two new species of *Myrmedonota* Cameron (Staphylinidae, Aleocharinae) from Mexico. *Zookeys*, **464**, 49-62.

Saulcy, F. de (1864) Descriptions des espèces nouvelles de Coléoptères recueillies en Syrie, en Égypte et en Palestine, pendant les mois d'octobre 1863 à janvier 1864, par M. de Saulcy, Senateur, Membre de l'Institut. pt. 2. *Annales de la Société de Entomologique de France*, (4)**4**, 629-660.

Sawada, K. (1965) A new species of the genus *Ptenidium* from Japan (Coleoptera: Ptiliidae). *Fragmenta Coleopterologica*, **13**, 51.

Sawada, K. (1977) Studies on the genus *Athzeta* Thomson and its allies (Coleoptera, Staphylinidae) III. Japanese species described by the previous authors. *Contributions from the Biological Laboratory, Kyoto University*, **25**, 171-222.

Sawada, K. (1980) *Atheta* and its allies of Southeast Asia (Coleoptera: Staphylinidae) I. Reexamination of some species from Borneo and Singapore. *Pacific Insects*, **21**, 335-354.

Sawada, K. (1982) *Atheta* and its allies of Southeast Asia (Coleoptera: Staphylinidae) III. Oriental species described by V. von Motschulsky and G. Kraatz. *Contributions from the Biological Laboratory, Kyoto University*, **26**, 141-187.

Sawada, K. (1984) Studies on the genus *Atheta* Thomson and its allies (Coleoptera, Staphylinidae) IV. Systematic studies on *Liogluta* series with notes of taxa established in C. G. Thomson, 1858 and G. Kraatz, 1859. *Contributions from the Biological Laboratory, Kyoto University*, **26**, 429-452.

Sawada, K. (1985) *Atheta* and its allies of Southeast Asia (Coleoptera: Staphylinidae) IV. Ten oriental species described by M. Bernhauer and M. Cameron. *Contributions from the Biological Laboratory, Kyoto University*, **27**, 91-110.

Sawada, K. (1987) *Atheta* and its allies of Southeast Asia (Coleoptera: Staphylinidae) V. Singaporean species described in Cameron, (1920) *Contributions from the Biological Laboratory, Kyoto University*, **27**, 137-150.

Sawada, K. (1989) New species of Aleocharinae from Japan, I (Coleoptera, Staphylinidae). *Contributions from the Biological Laboratory, Kyoto University*, **27**, 273-307.

Sawada, Y. & T. Hirowatari. (2002) A revision of the genus *Acrotrichis* Motschulsky (Coleoptera: Ptiliidae) in Japan. *Entomological science*, **5**, 77-101.

Schawaller, W. (1979) Eine neue *Agyrtes*-Art aus dem Kashmir-Himalaya mit ergänzenden Bemerkungen zur Gattung (Insecta: Coleoptera: Silphidae). *Senckenbergiana biologica*, **59**, 399-405.

Schawaller, W. (1982) Die Aaskäfer des Himalaya (Insecta: Coleoptera: Silphidae s. str.). *Senckenbergiana biologica*, **62**, 237-260.

Schawaller, W. (1996) Eine neue *Silpha*-Art aus China (Coleoptera: Silphidae). *Entomologische Zeitschrift, mit Insekten-Börse*, **106**, 139-143.

Schawaller, W. (1999) A new species of *Agyrtes* from China, and new faunistic data on Palaearctic Agyrtidae (Coleoptera). *Linzer biologische Beiträge*, **31**, 713-718.

Scheerpeltz, O. (1926) Ein neues mikrophthalmes *Lathrobium* (Col. Staphyl.) vom Obir in Kärnten. (8. Beitrag zur Kenntnis der paläarktischen Staphylinidenfauna.). *Koleopterologische Rundschau*, **12**, 193-203.

Scheerpeltz, O. (1926) Zwei neue Arten aus dem Subg. *Gyrohypnus* Mannerh. der Gattung *Xantholinus* Serv. (Col. Staphyl.). Mit einer Übersicht der mir bekannten paläarktischen Arten. *Coleopterologisches Centralblatt*, **1**, 81-93.V

Scheerpeltz, O. (1937) Über einen zoogeographisch bemerkenswerten Fund der Art *Acrolocha pliginskii* Bernh. aus Finnland. Mit einer Be[s]timmungstabelle der bisher bekannt gewordenen Arten der Gattung *Acrolocha* Thoms. (Col. Staphylinidae). *Notulae Entomologicae*, **17**, 119-123.

Scheerpeltz, O. (1944) Eine für Europa neue Art der Gattung *Lithocharis* Boisd. Lac. (Col. Staphylindae) (36. Beitrag zur Kenntnis der paläarktischen Staphyliniden). *Entomologische Blätter*, **40**, 33-38.

Scheerpeltz, O. (1947) A British and continental species of *Autalia* Mannerh. (Col. Staphylinidae) new to science, with a key to palaearctic species of the genus. *Entomologist's Monthly Magazine*, **83**, 104-107.

Scheerpeltz, O. (1947) Neue Staphyliniden (Coleoptera) aus Österreich. I. *Sitzungsberichte der Österreichische Akademie der Wissenschaften Mathematisch-naturwissenschaftliche. Klasse, Abt. 1*, **156**, 251-356.

Scheerpeltz, O. (1957) Neue Arten der Gattungen *Leptusa* Kr. und *Sipalia* Muls. Rey aus dem Museo Civico di Storia Naturale di Trieste (70. Beitrag zur Kenntnis der paläarktischen Staphyliniden) (Col. Staphylinidae). *Memorie della Societa entomologica italiana*, **36**, 53-60.

Scheerpeltz, O. (1962) Ein neuner *Staphylinus* aus Syrien (111. Beitrag zur Kenntnis der paläarktischen Staphyliniden). *Zeitschrift der Arbeitsgemeinschaft Österreichischer Entomologen*, **14**, 19-22.

Scheerpeltz, O. (1963) Coleoptera, Staphylinidae. *Lunds Universitets Årsskrift (N.F.) Avd. 2*, **58**, 1-38.

Scheerpeltz, O. (1965) Wissenschaftliche Ergebnisse der Schwedischen Expedition 1934 nach Indien und Burma. Coleoptera Staphylinidae (except Megalopsidiinae et Steninae). *Arkiv för Zoologi*, (2)**17**, 93-371.

Scheerpeltz, O. (1967) Eine neue alpine Art der Gattung *Amischa* C. G. Thomson (Col. Staphylinidae) (138. Beitrag zur Kenntnis der paläarktischen Staphyliniden). *Entomologische Blätter*, **63**, 13-15.

Scheerpeltz, O. (1971) Studien an den Arten der Gattung *Hesperus* Fauvel (Col. Staphylinidae). *Entomologische Arbeiten aus dem Museum G. Frey*, **22**, 150-197.

Scheerpeltz, O. (1972) Wissenschaftliche Ergebnisse der Studienreise von Gy. Topál nach Südwest-Argentinien (Coleoptera: Staphylinidae). *Folia Entomologica Hungarica, (New Series)*, **25**(Suppl.): 1-269.

Scheerpeltz, O. (1974) Coleoptera: Staphylinidae (exclus. Subfam. Paederinae, except. pars min.). In: B. Hanstrom, P. Brinck and G. Rudebeck (eds.), South African Animal Life, 15, 43-394.

Scheerpeltz, O. (1976) Wissenschaftliche Ergebnisse entomologischer Aufsammlungen in Nepal (Col. Staphylinidae). In: W. Hellmich and H. Janetschek, "Khumbu Himal" Ergebnisse des Forschungsunternehmens Nepal Himalaya. 5, 77-173. Innsbruck: Wagner.

Schillhammer, H. (1991) Four new Philonthini from Asia and synonymical notes on the genus *Philonthus* Curtis (Coleoptera: Staphylinidae). *Koleopterologische Rundschau*, **61**, 51-56.

Schillhammer, H. (1992) Six new Palaearctic species of the genus *Gabrius* Stephens and synonymical notes on some Philonthini. *Koleopterologische Rundschau*, **62**, 61-67.

Schillhammer, H. (1996) A new species of the genus *Naddia* Fauvel from Viet Nam and Laos (Insecta: Coleoptera: Staphylinidae). *Annalen des Naturhistorischen Museums in Wien*, **98**, 421-424.

Schillhammer, H. (1998) Revision of the east Palaearctic and Oriental species of *Philonthus* Stephens—Part 1. The *cyanipennis* group (Coleoptera: Staphylinidae, Staphylininae). *Koleopterologische Rundschau*, **68**, 101-118.

Schillhammer, H. (1999) Revision of the East Palaearctic and Oriental species of *Philonthus* Stephens, part 2. The *spinipes* and *cinctulus* groups (Coleoptera: Staphylinidae, Staphylininae). *Koleopterologische Rundschau*, **69**, 55-65.

Schillhammer, H. (1999) Synopsis of the genus *Hesperopalpus* Shibata, 1973 (Insecta: Coleoptera: Staphylinidae) with description of a new species from Vietnam. *Annalen des Naturhistorischen Museums in Wien*, **101**, 303-307.

Schillhammer, H. (2000) Revision of the East Palaearctic and Oriental species of *Philonthus* Stephens Part 3. The politus complex (Coleoptera: Staphylinidae, Staphylininae). *Koleopterologische Rundschau*, **70**, 113-176.

Schillhammer, H. (2002) Three new Oriental species of *Hesperus* Fauvel (Coleoptera: Staphylinidae). *Koleopterologische Rundschau*, **72**, 127-135.

Schillhammer, H. (2003) On some Central Asian species of the *Gabrius astutus* group (Insecta: Coleoptera: Staphylinidae). *Annalen des Naturhistorischen Museums in Wien*, **104**, 353-361.

Schillhammer, H. (2003) Revision of the East Palaearctic and Oriental species of *Philonthus* Stephens - Part 5. The *rotzmdicollis* and *sanguinolentus* species groups (Coleoptera: Staphylinidae, Staphylininae). *Koleopterologische Rundschau*, **73**, 85-136.

Schillhammer, H. (2006) Revision of the genus *Algon* Sharp (Coleoptera: Staphylinidae: Staphylininae). *Koleopterologische Rundschau*, **76**, 135-218.

Schillhammer, H. (2008) New species and records of *Algon* Sharp (Coleoptera: Staphylinidae: Staphylininae). *Koleopterologische Rundschau*, **78**, 233-240.

Schillhammer, H. (2009) Notes on some West Palearctic Staphylinini, with description of a new species from Spain (Coleoptera: Staphylinidae: Staphylininae). *Koleopterologische Rundschau*, **79**, 97-116.

Schillhammer, H. (2016) *Hesperus rougemonti* sp. n. (Coleoptera, Staphylinidae) from Borneo. *Zootaxa*, **4061**, 197-200.

Schillhammer, H., S. Snäll, M. Coskun & H. Jansson (2007) The West Palearctic species of *Hesperus* Fauvel, 1874, with descriptions of three new species from Turkey (Coleoptera: Staphylinidae: Staphylininae). *Koleopterologische Rundschau*, **77**, 132-132.

Schmidt-Göbel, H.M. (1836) Dissertatio Inauguralis Zoologica de Pselaphis Faunae Pragensis cum Anatomia Clavigeri.

Schubert, K. (1911) Neue exotische Staphyliniden. (Col.). *Deutsche Entomologische Zeitschrift*, **1911**, 1-39.

Schülke, M. & A. Kleeberg (1997) Eine neue Art der Gattung *Tachinus* Gravenhorst aus Nepal (Coleoptera: Staphylinidae). *Beiträge zur Entomologie*, **47**, 353-358.

Schülke, M. (1990) *Sepedophilus*-Studien II. *Sepedophilus* apfelbecki (Luze, 1902) und *Sepedophilus* michailovi spec. nov. aus Tadshikistan (Staphylinidae, Tachyporinae). *Novius*, **11**, 243-250.

Schülke, M. (1991) Studien zur Systematik und Faunistik der Gattung *Tachyporus* Gravenhorst (Coleoptera: Staphylinidae: Tachyporinae). Teil 2. Revision von G. Luze beschriebener mittelasiatischer Arten und Beschreibung einer neuen Art aus Afghanistan. *Annalen des Naturhistorischen Museums in Wien*, **92**, 211-227.

Schülke, M. (1995) Studien zur Systematik und Faunistik der Gattung *Tachyporus* Gravenhorst (Col., Staphylinidae, Tachyporinae). Teil 3. *Entomologische Nachrichten und Berichte*, **39**, 81-89.

Schülke, M. (1995) *Tachyporus lohsei* spec. nov., die erste Art der Gattung vom südchinesischen Festland (Coleoptera, Staphylinidae). *Entomologische Blätter für Biologie und Systematik der Käfer*, **91**, 142-148.

Schülke, M. (1996) Studien zur Systematik und Faunistik der Gattung *Tachyporus* Gravenhorst (Coleoptera: Staphylinidae) Teil 4: *Tachyporus* vafer spec. nov., eine neue Art aus Mitteleuropa. *Linzer biologische Beiträge*, **28**, 483-495.

Schülke, M. (1997) Beitrag zur Systematik der Gattung *Tachinus* Gravenhorst, 1802 (Insecta: Coleoptera: Staphylinidae: Tachyporinae). *Reichenbachia*, **32**, 41-48.

Schülke, M. (1997) Studien zur Systematik und Faunistik der Gattung *Tachyporus* Gravenhorst. Teil 5: Zur Kenntnis der wespaläarktischen Arten der *Tachyporus atriceps* Stephens, 1832 - Gruppe (Coleoptera: Staphylinidae). *Koleopterologische Rundschau*, **67**, 131-158.

Schülke, M. (1998) Eine neue Art der Gattung *Ischnosoma* Stephens aus Griechenland (Insecta: Coleoptera: Staphylinidae: Tachyporinae). *Reichenbachia*, **32**, 231-234.

Schülke, M. (1998) Studien zur Systematic und Faunistik der Gattung *Tachyporus* Gravenhorst. Teil 6: Zum Vorkommen von *Tachyporus* im südlichen Afrika (Insecta: Coleoptera: Staphylinidae: Tachyporinae). *Reichenbachia*, **32**, 225-229.

Schülke, M. (1998) Studien zur Systematik und Faunistik der Gattung *Tachyporus* Gravenhorst — Teil 7. Alte und neue *Tachyporus* aus dem Fernen Osten, Sachalin und Japan. *Beiträge zur Entomologie*, **48**, 367-406.

Schülke, M. (1998) Über *Sepedophilus cavicola* (Scriba, 1870) und S. crypticola (Rey, 1881). (Coleoptera: Staphylinidae, Tachyporinae). *Beiträge zur Entomologie*, **48**, 407-410.

Schülke, M. (1998) Zwei bisher verkannte westpaläarktische *Sepedophilus*-Arten (Coleoptera, Staphylinidae). *Entomologische Blätter für Biologie und Systematik der Käfer*, **94**, 57-63.

Schülke, M. (1999) A new species of *Derops* Sharp from China (Coleoptera, Staphylinidae, Tachyporinae). *Linzer biologische Beiträge*, **31**, 345-350.

Schülke, M. (1999) Über *Sepedophilus nigripennis* (Stephens, 1832) (Coleoptera, Staphylinidae). *Entomologische Blätter für Biologie und Systematik der Käfer*, **95**, 55-60.

Schülke, M. (1999) Über *Sepedophilus transcaspicus* (Bernhauer, 1917) (Coleoptera, Staphylinidae). *Entomologische Blätter für Biologie und Systematik der Käfer*, **95**, 53-54.

Schülke, M. (2000) Eine bemerkenswerte Art der Gattung *Lordithon* Thomson aus Australien (Coleoptera, Staphylinidae, Tachyporinae). *Linzer biologische Beiträge*, **32**, 883-889.

Schülke, M. (2000) Eine weiterre neue Art der Gattung *Derops* Sharp aus China (Coleoptera, Staphylinidae, Tachyporinae). *Linzer biologische Beiträge*, **32**, 913-916.

Schülke, M. (2000) Untersuchungen zur Systematik und Taxonomie der Gattung *Lordithon* Thomson, 1859. *Koleopterologische Rundschau*, **70**, 87-111.

Schülke, M. (2001) Eine neue Art und neue Funde von Arten der Gattung *Ischnosoma* Stephens, 1829 (Insecta: Coleoptera: Staphylinidae: Tachyporinae). *Reichenbachia*, **34**, 127-135.

Schülke, M. (2001) Eine neue Art und neue Funde von Arten der *Ischnosoma spelaeum*-Gruppe aus Anatolien (Coleoptera, Staphylinidae, Tachyporinae). *Linzer biologische Beiträge*, **35**, 453-460.

Schülke, M. (2002) A new microphthalmous *Lathrobium* (Coleoptera, Staphyliniade, Paederinae) from Sichuan. *Special Bulletin of the Japanese Society of Coleopterology*, **(5)**, 251-254.

Schülke, M. (2003) Beitrag zur Kenntnis der *Tachinus*-Arten Taiwans und der Ryukyu-Inseln (Coleoptera: Staphylinidae, Tachyporinae). *Linzer biologische Beiträge*, **35**, 763-784.

Schülke, M. (2003) Übersicht über die *Derops*-Arten Chinas und der angrenzenden Gebiete (Coleoptera: Staphylinidae, Tachyporinae). *Linzer biologische Beiträge*, **35**, 461-486.

Schülke, M. (2004) Über einige von T.V. Wollaston von Inseln des Madeira-Archipels beschriebene Staphyliniden-Arten (Coleoptera: Staphylinidae: Omaliinae, Oxytelinae et Staphylininae). *Linzer biologische Beiträge*, **36**, 393-415.

Schülke, M. (2004) Zur Taxonomie der Tachyporinae (Coleoptera: Staphylinidae) Typenrevision, Typendesignation, Neukombinationen, Untergattungszuordnungen, Nomina nova und neue Synonymien. *Linzer biologische Beiträge*, **36**, 919-1000.

Schülke, M. (2005) Zur Kenntnis einiger von Motschulsky (1858) beschriebenen Arten der Gattung *Coproporus* Kraatz (Coleoptera, Staphylinidae, Tachyporinae). *Linzer biologische Beiträge*, **37**, 1625-1632.

Schülke, M. (2006) Drei neue Adventivarten der europäischen Staphyliniden-Fauna, mit Bemerkungen zu *Coproporus colchicus* Kraatz (Coleoptera, Staphylinidae, Tachyporinae). *Entomologische Blätter für Biologie und Systematik der Käfer*, **102**, 173-201.

Schülke, M. (2006) *Tachinus* (*Tachinoderus*) *joachimschmidti* n. sp., eine neue brachyptere Art der Gattung *Tachinus* Gravenhorst aus Nepal (Coleoptera, Staphylinidae, Taehyporinae). *Veröffentlichungen Naturkundemuseum Erfurt*, **25**, 217-221.

Schülke, M. (2006) Zur Kenntnis einiger Arten der Gattung *Tachinus* Gravenhorst aus dem Himalaya-Gebiet und Südwest-China (Coleoptera, Staphylinidae, Tachyporinae). - in: Hartmann, M. & J. Weipert (Hrsg.): Biodiversität und Naturausstattung im Himalaya II. - Verein der Freunde und Förderer des Naturkundemuseums Erfurt, **5**, 327-342.

Schülke, M. (2006) Zur Kenntnis von *Tachyporus evanescens* Boheman 1858 und *Paracilea insulicola* Watanabe & Shibata 1972 (Coleoptera, Staphylinidae, Tachyporinae). *Linzer biologische Beiträge*, **38**, 845-852.

Schülke, M. (2007) On the Turkish species of the genus *Ischnosoma* (Insecta: Coleoptera: Staphylinidae: Tachyporinae). *Entomological Problems*, **37**, 21-30.

Schülke, M. (2010) Zur Taxonomie und Systematik einiger Arten der Untergattung *Bledius* Leach 1819 (Coleoptera, Staphylinidae, Oxytelinae). *Linzer biologische Beiträge*, **42**, 1495-1509.

Schülke, M. (2011) *Anchocerus smetanai* nov.sp., eine neue Art aus Yunnan (Staphylinidae, Staphylininae, Staphylinini, Quediina). *Entomofauna*, **32**, 461-468.

Schülke, M. (2011) Zur Kenntnis der Verwandtschaft von *Bledius* (*Hesperophilus*) *atricapillus* (GERMAR) (Coleoptera, Staphylinidae: Oxytelinae). *Linzer biologische Beiträge*, **43**, 1595-1608.

Schülke, M. (2012) Vier neue paläarktische Oxytelini (Coleoptera, Staphylinidae, Oxytelinae). *Linzer biologische Beiträge*, **44**, 1641-1666.

Schülke, M. (2012) Zur Verbreitung von *Anotylus speculifrons* (Kraatz, 1857), *A. clypeonitens* (Pandellé, 1867) und *A. schatzmayri* (Koch, 1937) (Coleoptera, Staphylinidae, Oxytelinae). *Entomologische Blätter und Coleoptera*, **108**, 121-130.

Schülke, M. (2013) Zwei neue Arten der *Tachinus subterraneus* Gruppe aus der Türkei und dem Irak (Coleoptera, Staphylinidae, Tachyporinae). *Entomofauna*, **34**, 609-624.

Schweiger, H. (1964) *Choleva vornatscheri* Schweig., neu für Kärnten (Col., Catopidae). *Mitteilungen des Naturwissenschaftlichen Vereins für Kärnten*, **73**, 192-193.

Seago, A. E. (2009) Revision of *Agyrtodes* Portevin (Coleoptera: Leiodidae). *Coleopterists Bulletin*, **63**(suppl. 7), 1-73.

Secq, B. & M. Secq (1991) Description d'un nouveau *Bryaxis* des Pyrénées (Col. Pselaphidae). *L'Entomologiste*, **47**, 253-257.

Secq, B. (2013) Une nouvelle espèce du genre *Bathysciola* Jeannel de la région pyrénéenne (Coleoptera, Leiodidae). *Bulletin de la Société entomologique de France*, **118**, 421-425.

Seevers, C. H. (1951) A revision of the north American and European Staphylinid Beetles of the subtribe *Gyrophaena*e (Aleocharinae, Bolitocharini). *Fieldiana: Zoology*, **32**, 655-762.

Seevers, C. H., and H. S. Dybas (1943) A synopsis of the Limulodidae (Coleoptera): a new family proposed for myrmecophiles of the subfamilies Limulodinae (Ptiliidae) and Cephaloplectinae (Staphylinidae). *Annals of the Entomological Society of America*, **36**, 546-586.

Semenov, V.B. (2003) Zur Kenntnis der sibirischen *Atheta*-Arten der Untergattung *Acrotona* Thomson, 1859 (Coleoptera: Staphylinidae: Aleocharinae). *Russian Entomological Journal*, **12**, 299-304.

Sharp, D. (1886) On New Zealand Coleoptera, with descriptions of new genera and species. *Scientific Transactions of the Royal Dublin Society*, (2)**3**, 351-454.

Sharp, D. (1900) Some undescribed species of *Trogophloeus*, with a new genus. *Entomologist's Monthly Magazine*, **36**, 230-234.

Sharp, D. S. (1869) XIV. A Revision of the British Species of *Homalota*. *Transactions of the Royal Entomological Society of London*, **17**, 91-272.

Sharp, D. S. (1871) Notes on some British species of *Oxypoda*, with description of new species. *Transactions of the Entomological Society of London*, **(2)**, 187-192.

Sharp, D. S. (1874) The Staphylinidae of Japan. *Transactions of the Entomological Society of London*, **1874**, 1-103.

Sharp, D. S. (1876) Contribution to an insect fauna of the Amazon Valley. Coleoptera-Staphylinidae. *Transactions of the Entomological Society of London*, **1876**, 27-424.

Sharp, D. S. (1876) Description of some new genera and species of New Zealand Coleoptera. *Entomologist's Monthly Magazine*, **13**, 20-28, 70-77, 97-102.

Sharp, D. S. (1882–1887) Insecta. Coleoptera. 1(2) In: Godman, F.D. & Salvin, O. (eds.) (1879-1915), Biologia Centrali-Americana: zoology, botany and archaeology. pp. xvi+824+19plt.

Sharp, D. S. (1884) Staphylinidae. pp. 313-392. In Biologia Centrali-Americana. Insecta. Coleoptera. 1(2). London: Taylor & Francis.

Sharp, D. S. (1889) The Staphylinidae of Japan. *The Annals and Magazine, of Natural History*, (6)**3**, 28-44; 108-121; 249-267; 319-334; 406-419; 463-476.

Sharp, D. S. (1910) Diagnoses of some new species of *Gabrius*. *Entomologist's Monthly Magazine*, (2)**21**, 129-131.

Shavrin, A. (2010) Three new species of the genus *Lesteva* Latreille, 1797 (Coleoptera: Staphylinidae: Omaliinae: Anthophagini) from Uzbekistan, Tadzhikistan and Afghanistan. *Baltic Journal of Coleopterology*, **10**, 147-152.

Sheerpeltz, O. (1955) Eine neue Art der Gattung *Platystethus* Mannh., mit einer Bestimmungstabelle der westpaläarktischen Arten und Formen dieser Gattung (Col. Staphylinidae) (61. Beitrag zur Kenntnis der paläarktischen Staphyliniden). *Koleopterologische Rundschau*, **33**, 78-88.

Shen, S.-J., M.-J. Zhao & L.-Z. Li (2008) Two new records of genus *Megarthrus* from China (Coleoptera: Staphylinidae: Proteininae). *Journal of Shanghai Normal University (Natural Sciences)*, **37**, 178-181.

Shi, K. & H.-Z. Zhou. (2009) A new *Dianous* species (Coleoptera, Staphylinidae, Steninae) from China, with a key to Chinese species of the *coerulescens* complex. *Deutsche Entomologische Zeitschrift*, **56**, 289-294.

Shi, K. & H.-Z. Zhou. (2011) Taxonomy of the genus *Dianous* (Coleoptera: Staphylinidae: Steninae) in China and zoogeographic patterns of its distribution. *Insect Science*, **18**, 363-378.

Shibata Y. (1973) On the genus *Hesperus* Fauvel and one allied new genus from Taiwan, with descriptions of two new species (Col., Staphylinidae). *Entomological Review of Japan*, **25**, 21-27.

Shibata Y. (1975) Notes on the genus *Tympanophorus* Nordmann (Coleoptera, Staphylinidae) from Japan and Taiwan, with descriptions of a new species and a new subspecies. *Kontyû*, **43**, 20-28.

Shibata Y. (1979) New or little-known Staphylinidae (Coleoptera) from Taiwan. *Entomological Review of Japan*, **33**, 19-29.

Shibata Y. (1979) Notes on the genus *Tachinus* Gravenhorst from Taiwan, with descriptions of two new species (Coleoptera: Staphylinidae). *Transactions of the Shikoku Entomological Society*, **14**, 141-149.

Shibata Y. (1990) A new species of the genus *Hesperus* (Coleoptera, Staphylinidae) from Taiwan. *Elytra*, **18**, 209-214.

Shibata Y. (1991) Three new *Gabrius* (Coleoptera, Staphylinidae) from Japan. *Elytra*, **19**, 85-92.

Shibata Y. (1993) A new species of the genus *Coprophilus* (Coleoptera, Staphylinidae) from Taiwan. *Elytra*, **21**, 313-317.

Shibata Y. (1994) Two new species of the genus *Naddia* from Taiwan (Coleoptera: Staphylinidae). *Transactions of the Shikoku Entomological Society*, **20**, 315-320.

Shibata Y. (2002) A new species of the genus *Hesperus* (Coleoptera, Staphylinidae) from Yunnan Province, Southwest China. *Special Bulletin of the Japanese Society of Coleopterology*, **(5)**, 255-260.

Shibata Y. (2007) A new species of the genus *Gabrius* (Coleoptera, Staphylinidae) from Taiwan. *Elytra*, **35**, 63-67.

Shibata, T. (1969) Some reports on the burying beetles from Japan, I (Col., Silphidae). *Entomological Review of Japan*, **21**, 47-54.

Shibata, Y. (2001) A new species of the genus *Trichophya* (Coleoptera, Staphylinidae) from Taiwan. *Elytra*, **29**, 352-357.

Sikes, D. & T. Mousseau (2013) Description of *Nicrophorus* efferens, new species, from Bougainville Island (Coleoptera, Silphidae, Nicrophorinae). *Zookeys*, **311**, 83-93.

Sikes, D. S. & S. B. Peck (2000) Description of *Nicrophorus hispaniola*, new species, from Hispaniola (Coleoptera: Silphidae) and a key to the speices of *Nicrophorus* of the New World. *Annals of the Entomological Society of America*, **93**, 392-397.

Sikes, D. S., R. B. Madge, and S. T. Trumbo (2006) Revision of *Nicrophorus* in part: new species and inferred phylogeny of the nepalensis-group based on evidence from morphology and mitochondrial DNA (Coleoptera: Silphidae: Nicrophorinae). *Invertebrate Systematics*, **20**, 305-365.

Simon, H. (1883) *Bryaxis* Retowskii nov. spec. *Wiener Entomologische Zeitung*, **2**, 8.

Skale, A. & M. A. Jäch (2006) Description of *Hydraena* (*s. str.*) *alticola* sp. n., a high altitude water beetle from Nepal (Insecta: Coleoptera: Hydraenidae). In: Hartmann, M. & Weipert, J. (eds.). Biodiversität und Naturausstattung im Himalaya II. - Verein der Freunde und Förderer des Naturkundemuseums Erfurt e.V., Erfurt, pp. 325-326.

Smetana, A. & F.-K. Zheng. (2000) Contributions to the knowledge of the Quediina (Coleoptera, Staphylinidae, Staphylinini) of China. Part 17. Genus *Bolitogyrus* Chevrolat, 1842. Section 1. *Elytra*, **28**, 55-64.

Smetana, A. & J. M. Campbell (1980) A new genus and two new Phloeocharinae species from the Pacific Coast of North America (Coleoptera: Staphylinidae). *The Canadian Entomologist*, **112**, 1061-1069.

Smetana, A. & V. Grebennikov. (2008) *Ocypus* (*Matidus*) *primoriensis* Smetana, a new species from the Russian Far East (Coleoptera, Staphylinidae, Staphylinini). *Zootaxa*, **1782**, 65-68.

Smetana, A. (1951) Eine neue Untergattung und Art der Gattung *Silpha* L. aus der Hohen Tatra. *Acta Entomologica Musei Nationalis Pragae*, **27**, 65-68.

Smetana, A. (1952) Revise Československých druhů rodu *Gabrius* Steph. ze skupiny *Gabrius* nigritulus Grav. *Časopis Československé Společnosti Entomologické*, **49**, 109-132.

Smetana, A. (1953) Výsledyky zoologické expedice Národního Musea v Praze do Turecka. 12. Coleoptera III. Staphylinidae (genera *Philonthus* Curt., *Gabrius* Steph.). *Acta Entomologica Musei Nationalis Pragae*, **28**, 117-124.

Smetana, A. (1954) Středoevropské druhy rodu *Gabrius* Steph. ze skupiny *Gabrius splendidulus* Grav. (Coleoptera, Staphylinidae), 4. Příspěvek k poznání rodu *Gabrius* Steph. paleaktické oblasti. *Acta Entomologica Musei Nationalis Pragae*, **29**, 113-119.

Smetana, A. (1954) Výsledky zoologické expedice Národního musea v Praze do Turecka. 17. Coleoptera VI. Staphylinidae (genera *Philonthus* Curt., *Gabrius* Steph.). *Acta Entomologica Musei Nationalis Pragae*, **29**, 177-180.

Smetana, A. (1955) Beiträge zur Kenntnis der Gattung *Philonthus* Curt. (Col., Staphyl.), 1. Beitrag zur Kenntnis der Gattung *Philonthus* der paläarkt. Region. *Entomologische Blätter für Biologie und Systematik der Käfer*, **50**, 223-230.

Smetana, A. (1955) Beiträge zur Kenntnis der Gattung *Philonthus* Curt. II. (Coleoptera Staphylinidae). *Annales Historico-Naturales Musei Nationalis Hungarici* (Series Nova), **6**, 205-211.

Smetana, A. (1955) Středoevropské druhy rodu *Ontholestes* Gglb. (Coleoptera, Staphylinidae). *Acta Entomologica Musei Nationalis Pragae*, **30**, 283-289.

Smetana, A. (1955) Systematické a faunistické poznámky ke zvířeně drabčíků Československa. *Acta Societatis Entomologicae Čechosloveniae*, **51**, 135-148.

Smetana, A. (1957) Bestimmungstabelle der europäischen Arten der Gattung *Gabrius* Steph., 11. Beitrag zur Kenntnis der Gattung *Gabrius* Steph. (Col., Staphylinidae) der paläarkt. Region. *Entomologische Blätter für Biologie und Systematik der Käfer*, **53**, 56-79.

Smetana, A. (1957) Bestimmungstabelle der europäischen Arten der Gattung *Gabrius* Steph. *Entomologische Blätter für Biologie und Systematik der Käfer*, **53**, 56-79.

Smetana, A. (1957) Systematické a faunistické poznámky ke zvířeně drabčíků Československa III. (Col., Staphylinidae). *Acta Societatis Entomologicae Čechosloveniae*, **54**, 246-262.

Smetana, A. (1958) Eine neue Art der Gattung *Ontholestes* Gglb. aus Deutschland (Col. Staphylinidae). *Deutsche Entomologische Zeitschrift, (N.F)*, **5**, 363-366.

Smetana, A. (1958) Fauna ČSR. Svazek 12. Drabčíkovití-Staphylinidae. I. Staphylininae. (Řád: Brouci-Coleoptera). 435 pp. Praha: Českosolvenské Akademie Věd.

Smetana, A. (1959) Bestimmungstabelle der mitteleuropäischen Arten der Gattung *Philonthus* Curt. *sensu lato*, 4. Beitrag zur Kenntnis der Gattung *Philonthus* Curt. (Col., Staphylinidae) der paläarktischen Region. *Entomologische Blätter für Biologie und Systematik der Käfer*, **54**, 140-175.

Smetana, A. (1959) Palaearctic, Oriental and Nearctic species of the genus *Ontholestes* Gglb. (Col., Staphylinidae). *Acta Entomologica Musei Nationalis Pragae*, **33**, 393-412.

Smetana, A. (1959) Zur Kenntnis der Staphyliniden-Fauna Albaniens. *Acta Entomologica Musei Nationalis Pragae*, **33**, 195-218.

Smetana, A. (1960) Monographische Bearbeitung der paläarktischen Arten der Gattung *Gabrius* Curt. aus der *nigritulus*-Gruppe. *Deutsche Entomologische Zeitschrift*, (N.F.), **7**, 295-356.

Smetana, A. (1961) Eine neue Art der Gattung *Gabrius* Curt. aus dem Kaukasus (Col., Staphylinidae). *Acta Entomologica Musei Nationalis Pragae*, **34**, 33-39.

Smetana, A. (1961) Systematické a faunistické poznámky ke zvířeně drabčíků Československa V. (Col., Staphylinidae). *Acta Musei Silesiae (Series A)*, **10**, 113-122.

Smetana, A. (1962) Bestimmungstabelle der mitteleuropäischen Arten der Gattung *Quedius* Steph. (Col., Staphylinidae). *Entomologische Blätter für Biologie und Systematik der Käfer*, **58**, 133-155.

Smetana, A. (1962) Zur Kenntnis der auf Madeira und auf den Kanarischen Inseln lebenden *Gabrius*-Arten aus der *nigritulus*-Gruppe (Col., Staphylinidae). *Entomologisk Tidskrift*, **83**, 95-102.

Smetana, A. (1963) *Oxypoda nigricornis* Motsch. nový boreoalpinní druh pro zvířenu Československa (Col., Staphylinidae). *Biológia, Bratislava*, **18**, 156-160.

Smetana, A. (1964) Paederinae and Staphylinidae from Kodiak Island, Alaska (Col. Staphylinidae). *Opuscula Entomologica, Lund*, **29**, 34-40.

Smetana, A. (1964) *Stenus* humiloides, n. sp. eine neue Art aus der Verwandtschaft von *Stenus* (*Nestus*) *humilis* Er. (Col., Staphylinidae). *Časopis Československé Společnosti Entomologické*, **61**, 47-52.

Smetana, A. (1965) Staphylinini und Quediini (Col., Staphylinidae) von Newfoundland, Südost-Labrador und Nova Scotia. *Acta Entomologica Fennica*, **20**, 1-60.

Smetana, A. (1965) Zur Kenntnis der *Staphylinus*- und *Ocypus*-Arten Nordanatoliens (Coleoptera, *Staphylinus*). *Reichenbachia*, **5**, 25-46.

Smetana, A. (1966) Systematische und Faunistische Beiträge zur Kenntnis der Staphylinidenfauna der Tschechoslowakei VII. (Col., Staphylinidae). *Acta Entomologica Bohemoslovaca*, **63**, 322-336.

Smetana, A. (1967) Ergebnisse der Zoologischen Forschungen von Dr. Z. Kaszab in der Mongolei. 86. Staphylinidae II. Unterfamilien Paederinae, Xantholininae und Staphylininae (Coleoptera). *Acta Entomologica Bohemoslovaca*, **64**, 195-218.

Smetana, A. (1967) Wissenschaftliches Ergebnis der zoologischen Expedition des Nationalmuseum in Prag nach der Türkei. Coleoptera-Staphylinidae, Subfam. Oxytelinae. *Acta Entomologica Musei Nationalis Pragae*, **37**, 297-324.

Smetana, A. (1967) Wissenschaftliches Ergebnis der zoologischen Expedition des Nationalmuseum in Prag nach der Türkei. Coleoptera-Staphylinidae, Subfam. Staphylininae. *Acta Entomologica Musei Nationalis Pragae*, **37**, 551-564.

Smetana, A. (1967) Zur Kenntnis der *Gabrius*-Arten Spaniens (Col., Staphylinidae). *Acta Faunistica Entomologica Musei Nationalis Pragae*, **12**, 153-160.

Smetana, A. (1968) Zur Kenntnis der *Staphylinus*- und *Ocypus*-Arten Anatoliens (Col., Staphylinidae). Sammelausbeuten von W. Heinz und H. Korge in Kleinasien. *Acta Faunistica Entomologica Musei Nationalis Pragae*, **13**, 155-161.

Smetana, A. (1971) Revision of the tribe Quediini of America north of Mexico (Coleoptera: Staphylinidae). *Memoirs of the Entomological Society of Canada*, **79**, vi + 1-303.

Smetana, A. (1973) Revision of the tribe Quediini of America North of Mexico (Coleoptera: Staphylinidae). Supplementum 2. *The Canadian Entomologist*, **105**, 1421-1434.

Smetana, A. (1973) Über einige von Dr. M. Bernhauer beschriebene *Gabrius*-Arten (Coleoptera, Staphylinidae). *Nouvelle Revue d'Entomologie*, **3**, 125-136.

Smetana, A. (1975) A collection of Quediini from Nepal (Coleoptera: Staphylinidae). *Oriental Insects*, **9**, 323-342.

Smetana, A. (1975) Ergebnisse der zoologischen Forschungen von Dr. Z. Kaszab in der Mongolie. 340. Staphylinidae IV. Unterfamilien Omaliinae bis Staphylininae (Coleoptera). *Acta Zoologica Academiae Scientiarum Hungaricae*, **21**, 153-179.

Smetana, A. (1975) New and little known high altitude *Quedius* from Mexico (Coleoptera: Staphylinidae). *The Canadian Entomologist*, **107**, 311-323.

Smetana, A. (1976) New species and remarks on Siberian *Quedius* (Coleoptera: Staphylinidae). *Notulae Entomologicae*, **56**, 21-28.

Smetana, A. (1976) Review of the Central American species of the subgenus *Quedionuchus* of the genus *Quedius* (Col. Staphylinidae). *Studies on Neotropical Fauna and Environment*, **11**, 223-247.

Smetana, A. (1976) Review of the Mexican species of the subgenus *Microsaurus* of the genus *Quedius* (Coleoptera: Staphylinidae). *The Canadian Entomologist*, **108**, 113-118.

Smetana, A. (1976) Revision of the tribe Quediini of America north of Mexico (Coleoptera: Staphylinidae). Supplementum 3. *The Canadian Entomologist*, **108**, 169-184.

Smetana, A. (1977) Ergebnisse der Bhutan-Expedition 1972 des Naturhistorischen Museums in Basel. Coleoptera: Fam. Staphylinidae Tribus Quediini. *Entomologica Basiliensia*, **2**, 243-250.

Smetana, A. (1977) New and interesting *Gabrius*, Rabigus and *Philonthus* from Turkey (Coleoptera, Staphylinidae). *Revue suisse de Zoologie*, **84**, 791-797.

Smetana, A. (1978) Revision of the tribe Quediini of America north of Mexico (Coleoptera: Staphylinidae). Supplementum 4. *The Canadian Entomologist*, **110**, 815-840.

Smetana, A. (1980) Review of the Mexican species of the genus *Nudobius* C. G. Thomson (Col., Staphylinidae). *Coleopterists Bulletin*, **34**, 159-165.

Smetana, A. (1980) Three species of the genus *Leptacinus* Er. described by Kraatz in 1859 (Coleoptera: Staphylinidae). *Entomologica scandinavica*, **11**, 49-55.

Smetana, A. (1981) Revision of the tribe Quediini of America North of Mexico (Coleoptera: Staphylinidae). Supplementum 5. *The Canadian Entomologist*, **113**, 631-644.

Smetana, A. (1982) Revision of the subfamily Xantholininae of America North of Mexico (Coleoptera: Staphylinidae). *Memoirs of the Entomological Society of Canada*, **12**, 1-389.

Smetana, A. (1984) A new species of the genus *Gabrius* Stephens from Japan (Coleoptera, Staphylinidae). *Revue suisse de Zoologie*, **91**, 647-650.

Smetana, A. (1984) Review of the Japanese species of the genus *Gabrius* Stephens (Coleoptera, Staphylinidae). *The Pan-Pacific Entomologist*, **60**, 122-150.

Smetana, A. (1988) Revision of the tribes Quediini and Atanygnathini. Part II. The Himalayan region (Coleoptera: Staphylinidae). *Quaestiones Entomologicae*, **24**, 163-464.

Smetana, A. (1989) Review of the Bornean species of the genus *Oxyporus* Fabricius 1775 (Coleoptera, Staphylinidae). *Nouvelle Revue d'Entomologie*, **6**, 147-152.

Smetana, A. (1990) *Gabrius subnigritulus* (Reitter), a Palearctic species recently introduced into North America (Coleoptera: Staphylinidae). *Le Naturaliste Canadien*, **116**, 175-178.

Smetana, A. (1990) Old and new east-palaearctic species of *Quedius* Steph. from the subgenus *Raphirus* Steph. (Coleoptera, Staphylinidae). *Nouvelle Revue d'Entomologie*, **7**, 199-204.

Smetana, A. (1991) *Belonuchus minax* Erichson, 1840 redescription and lectotype designation (Coleoptera: Staphylinidae). *Koleopterologische Rundschau*, **61**, 49-50.

Smetana, A. (1991) *Philonthus furvus* Nordmann, 1837 and its allies in Mexico and Central America (Coleoptera: Staphylinidae). *Insecta Mundi*, **5**, 227-246.

Smetana, A. (1992) *Philonthus orphanus* Erichson, 1840. Taxonomy and lectotype designation (Coleoptera: Staphylinidae). *Elytron*, **5**, 135-137.

Smetana, A. (1992) Revision of the tribes Quediini and Atanygnathini. Part II. The Himalayan Region. Supplement 2. (Coleoptera: Staphylinidae: Staphylinidae). *Stuttgarter Beiträge Naturkunde. Biologie. (A)*, **487**, 1-11.

Smetana, A. (1992) The Himalayan and east Asiatic species of *Dinothenarus* Thomson (Coleoptera: Staphylinidae: Staphylininae). *Bulletin of the National Museum of Natural Science*, **3**, 187-198.

Smetana, A. (1994) Lectotype designations and redescriptions of three New World species of *Philonthus* Stephens, 1829, described by Erichson in 1840 (Coleoptera Staphylinidae). *Nouvelle Revue d'Entomologie, (n. ser.)*, **10**, 341-348.

Smetana, A. (1995) Contributions to the knowledge of the Quediina (Coleoptera, Staphylinidae, Staphylinini) of China. Genus *Quedius* Stephens, 1829. Part 2. Subgenus *Microsaurus* Dejean, 1833. Section 2. *Bulletin of the National Science Museum*, (A)**21**, 231-250.

Smetana, A. (1995) *Lordithon daviesi* (Coleoptera, Staphylinidae, Tachyporinae), a remarkable new species from Taiwan. *Elytra*, **23**, 229-233.

Smetana, A. (1995) Revision of the tribes Quediini and Tanygnathinini. Part III. Taiwan. (Coleoptera: Staphylinidae). National Museum of Natural Science. *Special Publication Number*, **6**, 1-145.

Smetana, A. (1995) Rove beetles of the subtribe Philonthina of America north of Mexico (Coleoptera: Staphylinidae). Classification, phylogeny and taxonomic revision. *Memoirs on Entomology, International*, **3**, 1-946.

Smetana, A. (1995) Taxonomic and faunistic contributions to the knowledge of Palaearctic Quediina (Coleoptera, Staphylinidae, Staphylinini). *Elytra*, **23**, 77-88.

Smetana, A. (1996) Contributions to the knowledge of the Quediina (Coleoptera, Staphylinidae, Staphylinini) of China. Part 3. Genus *Quedius* Stephens, 1829. Subgenus *Microsaurus* Dejean, 1833. Section 3. *Bulletin of the National Science Museum*, (A)**22**, 1-20.

Smetana, A. (1996) Contributions to the knowledge of the Quediina (Coleoptera, Staphylinidae, Staphylinini) of China. Part 4. Genus *Quedius* Stephens, 1829. Subgenus *Raphirus* Stephens, 1829. Section 1. *Elytra*, **24**, 49-59.

Smetana, A. (1996) Contributions to the knowledge of the Quediina (Coleoptera, Staphylinidae, Staphylinini) of China. Part 7. Genus *Quedius* Stephens, 1829. Subgenus *Raphirus* Stephens, 1829. Section 2. *Elytra*, **24**, 225-237.

Smetana, A. (1996) Contributions to the knowledge of the Quediina (Coleoptera, Staphylinidae, Staphylinini) of China. Part. 5. Genus *Quedius* Stephens, 1829. Subgenus *Microsaurus* Dejean. Section 4. *Bulletin of the National Science Museum*, (A)**22**, 113-132.

Smetana, A. (1996) Revision of the tribes Quediini and Tanygnathinini, part III, Taiwan, Supplement I. *Bulletin of the National Museum of Natural Science*, **8**, 23-28.

Smetana, A. (1997) Contributions to the knowledge of the Quediina (Coleoptera, Staphylinidae, Stahylinini) of China. Part 9. Genus *Quedius* Stephens, 1829. Subgenus *Microsaurus* Dejean, 1833. Section 7. *Elytra*, **25**, 451-473.

Smetana, A. (1997) Contributions to the knowledge of the Quediina (Coleoptera, Staphylinidae, Staphylinini) of China. Part 6. Genus *Quedius* Stephens, 1829. Subgenus *Microsaurus* Dejean, 1833. Section 5. *Bulletin of the National Museum of Science*, (A)**23**, 51-68.

Smetana, A. (1997) Two new species of the genus *Quedius* Stephens, 1829 (Coleoptera, Staphylinidae, Staphylinini, Quediina) from northern Vietnam. *Elytra*, **25**, 123-128.

Smetana, A. (1998) A new species of the genus *Coprophilus* Latreille, 1829 from the high mountain elevations in Taiwan, with comments on *Zonyptilus* Motschulsky, 1845 (Coleoptera: Staphylinidae: Oxytelinae). *Zoological Studies*, **37**, 154-158.

Smetana, A. (1998) Contributions to the knowldege of the Quediina (Coleoptera, Staphylinidae, Staphylinini) of China. Part 11. Genus *Quedius* Stephens, 1829. Subgenus *Distichalius* Casey, (1915) Section 1. *Elytra*, **26**, 315-332.

Smetana, A. (1998) Contributions to the knowledge of the Quediina (Coleoptera, Staphylinidae, Staphylinini) of China. Part 10. Genus *Quedius* Stephens, 1829. Subgenus *Raphirus* Stephens, 1829. Section 3. *Elytra*, **26**, 99-113.

Smetana, A. (1998) Taxonomic and faunistic contributions to the knowledge of Palearctic Quediina (Coleoptera, Staphylinidae, Staphylinini). Part 2. *Elytra*, **26**, 115-128.

Smetana, A. (1999) Contributions to the knowledge of the Quediina (Coleoptera, Staphylinidae, Staphylinini) of China. Part 13. Genus *Quedius* Stephens, 1829. Subgenus *Microsaurus* Dejean, 1833. Section 8. *Elytra*, **27**, 213-240.

Smetana, A. (1999) Contributions to the knowledge of the Quediina (Coleoptera, Staphylinidae, Staphylinini) of China. Part 15. Genus Strouhalium Scheerpeltz, (1962) Section 3. Genus *Quedius* Stephens, 1829. Subgenus *Microsaurus* Dejean, 1833. Section 9. *Elytra*, **27**, 519-534.

Smetana, A. (1999) Contributions to the knowledge of the Quediina (Coleoptera, Staphylinidae, Staphylinini) of China. Part 16. Genus *Quedius* Stephens, 1829. Subgenus *Microsaurus* Dejean, 1833. Section 10. *Elytra*, **27**, 535-551.

Smetana, A. (2000) Contributions to the knowledge of the Quediina (Coleoptera, Staphylinidae, Staphylinini) of China. Part 18. Genus *Bolitogyrus* Chevrolat, 1848. Section 2. *Elytra*, **28**, 327-330.

Smetana, A. (2001) Contributions to the knowledge of the Quediina (Coleoptera, Staphylinidae, Staphylinini) of China. Part 19. Genus *Quedius* Stephens, 1829. Subgenus *Microsaurus* Dejean, 1833. Section 11. *Elytra*, **29**, 181-191.

Smetana, A. (2001) Revision of the subtribe Quediina and the tribe Tanygnathinini. Part III. Taiwan. (Coleoptera: Staphylinidae). Supplement II. *Special Publication of the Japan Coleopterological Society*, **(1)**, 55-63.

Smetana, A. (2002) Contributions to the knowledge of the genera of the "*Staphylinus*-complex" (Coleoptera: Staphylinidae) of China. Part 2. The genus *Dinothenarus*, section 1. *Folia Heyrovskyana*, **10**, 205-224.

Smetana, A. (2005) Contributions to the knowledge of the genera of the "*Staphylinus*-complex" (Coleoptera: Staphylinidae) of China. Part 6. On the species collected recently in the Meishan Area, Sichuan. *Elytra*, **33**, 303-311.

Smetana, A. (2005) Contributions to the knowledge of the Quediina (Coleoptera, Staphylinidae, Staphylinini) of China. Part 25. Genus *Anchocerus* Fauvel, (1908) *Elytra*, **33**, 561-565.

Smetana, A. (2005) Contributions to the knowledge of the Quediina (Coleoptera, Staphylinidae, Staphylinini) of China. Part 26. Genus *Acylophorus* Nordmann, 1837. Section 1. *Elytra*, **33**, 567-570.

Smetana, A. (2007) Contributions to the knowledge of the "*Staphylinus*-complex" (Coleoptera: Staphylinidae) of China. Part 16. The genus *Ocypus* Leach, 1819, subgenus *Ocypus s. str.* and Matidus Motschulsky, 1860. *Elytra*, **35**, 441-459.

Smetana, A. (2007) Contributions to the knowledge of the "*Staphylinus*-complex" (Coleoptera: Staphylinidae: Staphylinini) of China. Part XX. The genus *Ocypus* Leach, 1819, subgenus *Pseudocypus* Mulsant & Rey, 1876. Section 1. *Zootaxa*, **1421**, 1-72.

Smetana, A. (2008) Contributions to the knowledge of the "*Staphylinus*-complex" (Coleoptera: Staphylinidae) of China. Part 17. The genus *Ocypus* Leach, 1819, subgenus *Pseudocypus* Mulsant & Rey, 1876. Section 2. *Elytra*, **36**, 167-180.

Smetana, A. (2009) A remarkable new species of *Tasgius* (Rayacheila) from Kyrgyzstan (Coleoptera, Staphylinidae, Staphylininae, Staphylinini). *Studies and reports of District Museum Prague-East Taxonomical*, **5**, 295-298.

Smetana, A. (2009) Contributions to the knowledge of the '*Staphylinus*-complex' (Coleoptera: Staphylinidae: Staphylinini) of China. Part 19. The genus *Ocypus* Leach, 1819, subgenus *Pseudocypus* Mulsant & Rey, 1876. Section 3. *Acta Entomologica Musei Nationalis Pragae*, **49**, 683-694.

Smetana, A. (2009) Contributions to the knowledge of the "*Staphylinus*-complex" (Coleoptera: Staphylinidae: Staphylinini) of China. Part 21. The genus *Ocypus* Leach, 1819, subgenus *Pseudocypus* Mulsant & Rey, 1876. Section 4. *Zootaxa*, **2286**, 1-30.

Smetana, A. (2010) Contributions to the knowledge of the '*Staphylinus*-complex' of China (Coleoptera: Staphylinidae: Staphylinini). Part 22. The genus *Tasgius* Stephens, 1829, Section 1. *Acta Entomologica Musei Nationalis Pragae*, **50**, 145-155.

Sokolowski, K. (1957) Zwei neue japanische Catopiden (Col. Catopidae) (Catopiden-Studien 6). *Deutsche Entomologische Zeitschrift*, (N.F.), **4**, 140-142.

Solodovnikov, A. Iu. (2000) New and little-known species of the genus *Ocypus* Leach in the fauna of the Caucasus (Coleoptera: Staphylinidae). *Zoosystematica Rossica*, **8**, 313-323.

Solodovnikov, A. Y. & A. F. Newton (2005) Phylogenetic placement of Arrowinini trib.n. within the subfamily Staphylininae (Coleoptera: Staphylinidae), with revision of the relict South African genus *Arrowinus* and description of its larva. *Systematic Entomology*, **30**, 398-441.

Solodovnikov, A. Y. (2005) A new species of *Stenus* (*s. str.*) (Coleoptra, Staphylinidae, Steninae) from the western Caucasus. *Euroasian Entomological Journal [Евразиатский энтомологический журнал]*, **4**, 221-222.

Solodovnikov, A. Y. (2008) Review of the Oriental genus *Anchocerus* with the description of new species and new combinations (Coleoptera: Staphylinidae: Staphylininae). *Insect Systematics & Evolution*, **39**, 287-301.

Solodovnikov, A., A. F. Newton (2010) Revision of the rove beetle genus *Antimerus* (Coleoptera, Staphylinidae, Staphylininae), a puzzling endemic Australian lineage of the tribe Staphylinini. *Zookeys*, **67**, 21-63.

Solsky, S. M. (1872) Enumération et description des coléoptères de la famille des Staphylinides recueillis par Mrs. C. Jelsky et le Baron de Nolken pendant leurs voyages dans l'Amérique du Sud en 1870 et 1871. *Horae Societatis Entomologicae Rossicae*, **8**, 289-314.

Song, C.-Z., J.-Y. Hu, X.-Y. Jin, M.-J. Zhao & L.-Z. Li (2003) On the species of the genus *Tachinus* (Coleoptera: Tachyporinae) from Mt West Tianmu, Zhejiang Province. *Journal of Shanghai Normal University (Natural Sciences)*, **32**, 83-90.

Song, X.-B., Li, L.-Z. (2013) Description of *Pella maoershanensis* sp. n. (Coleoptera, Staphylinidae, Aleocharinae) associated with *Lasius* spathepus from Guangxi, South China. *Zookeys*, **275**, 17-21.

Sörensson, M. (1988) Studies of Danish Ptiliidae (Coleoptera). *Entomologiske Meddelelser*, **56**, 35-48.

Sörensson, M. (2001) First find of *Acrotrichis henrici* (Matthews, 1872) in Germany (Coleoptera, Ptiliidae). *Entomologische Blätter*, **97**, 75-78.

Sparacio, I. (1995) Coleotteri di Sicilia. 238 pp. L'Epos Società Editrice.

Steel, W. O. (1948) A new species of *Xantholinus* (Col., Staphylinidae) from North Africa. *Entomologist's Monthly Magazine*, **84**, 186-187.

Steel, W. O. (1949) A new genus and species of Pacific (Chatham Is.) Staphylinidae. *Entomologist's Monthly Magazine*, **85**, 309-310.

Steel, W. O. (1949) The British species of *Staphylinus* subgenus *Ocypus* Steph. (Col., Staphylinidae). *Entomologist's Monthly Magazine*, **84**, 271-275.

Steel, W. O. (1957) Notes on the Omaliinae (Col., Staphylinidae). 8. The genus *Acrolocha* Thomson. *Entomologist's Monthly Magazine*, **93**, 157-164.

Stephens, J. F. (1832) Illustrations of British entomology; or, a synopsis of indigenous insects; containing their generic and specific distinctions; with an account of their metamorphoses, times of appearance, localities, food, and economy, as far as practicable. Mandibulata, 5, 1-240. London: Baldwin and Cradock.

Stephens, J. F. (1833) Illustrations of British entomology; or, a synopsis of indigenous insects; containing their generic and specific distinctions; with an account of their metamorphoses, times of appearance, localities, food, and economy, as far as practicable. Mandibulata, 5, 241-304. London: Baldwin and Cradock.

Stevanović, M. (2011) Study of the genus *Cephennium* Müller & Kunze, 1822 from the Balkan Peninsula. Part I.(Coleoptera: Staphylinidae: Scydmaeninae). *Genus*, **22**, 551-564.

Štourač, P. (2002) *Heterothops besucheti* sp. n. und *H. orientalis* sp. n. aus der Türkei (Coleoptera: Staphylinidae). *Revue suisse de Zoologie*, **109**, 735-739.

Sundt, E. (1968) *Acrotrichis insularis* (Mäklin, 1852) (Col., Ptiliidae) designation of lectotype and redescription. *Norwegian journal of entomology*, **15**, 75-77.

Sundt, E. (1969) Description of a new subgenus, *Flachiana*, and four new species of the genus *Acrotrichis* Motschulsky, 1848 (Col., Ptiliidae). *Norwegian journal of entomology*, **16**, 49-53.

Švec, Z. (1993) *Leiodes graeca* sp. n. from Greece (Coleoptera: Leiodidae). *European Journal of Entomology*, **90**, 77-78.

Švec, Z. (1996) *Leiodes ostocki* sp. n. with new faunistic records of Leiodini from the Caucasus and Central Asia (Coleoptera: Leiodidae). *Acta Entomologica Slovenica*, **4**, 73-77.

Švec, Z. (1999) New Asian *Leiodes* species with notes on Leiodinae from the collection of the Naturkundemuseum Erfurt (Coleoptera: Leiodidae). *Veröffentlichungen Naturkundemuseum Erfurt*, **18**, 163-168.

Švec, Z. (1999) New Nepalese species of the genera *Leiodes* Latreille and *Pseudcolenis* Reitter (Insecta: Coleoptera: Leiodidae: Leiodinae). In: M. Hartmann & H. Baumbach (eds.), Biodiversity and natural heritage of the Himalaya. Verein der Freunde und Foerderer des Naturkundemuseums Erfurt, e.V., Erfurt. Pp. 197-199.

Švec, Z. (2008) A new Sub-Saharan *Leiodes* Latreille species and new faunistic data on African *Zeadolopus* Broun (Coleoptera, Leiodidae, Leiodinae). *Studies and Reports of District Museum Prague-East, Taxonomical Series*, **4**, 259-262.

Švec, Z. (2008) New Chinese and Nepalese *Leiodes* Latreille (Coleoptera: Leiodidae: Leiodinae). *Studies and Reports of District Museum Prague-East, Taxonomical Series*, **4**, 241-258.

Švec, Z. (2009) Two new species of the genus *Leiodes*, with new records of the subfamily Leiodinae (Coleoptera: Leiodidae) from Turkey. *Folia Heyrovskyana, series A*, **17**, 81-88.

Szymczakowski, W. (1964) Révision des *Colon*idae (Coleoptera) des régions orientale et australienne. *Acta Zoologica Cracoviensia*, **9**, 469-527.

Tang, L., L.-Z. Li, J. Růžička (2011) Notes on the genus *Apteroloma* of China with description of a new species (Coleoptera, Agyrtidae). *Zookeys*, **124**, 41-49.

Telenov, D., C. Fägerström & M. Kalniņš (2006) *Ochthebius* (*Asiobates*) *remotus* Reitter, 1885 (Coleoptera: Hydraenidae) in Latvia, with selected general information on this species. *Latvijas Entomologs*, **43**, 33-38.

Thayer, M. K. & A. F. Newton (1979) Revision of the south temperate genus *Glypholoma* Jeannel, with four new species (Coleoptera: Staphylinidae: Omaliinae). *Psyche*, **85**, 25-63.

Thayer, M. K. (1985) Revision, phylogeny and biogeography of the austral genus *Metacorneolabium* Steel (Coleoptera: Staphylinidae: Omaliinae). Pp. 113-179. In: Ball, G. E. (ed.) Taxonomy, phylogeny and zoogeography of beetles and ants. Series entomologica (Dordrecht), 33.

Thayer, M. K. (1987) Biology and phylogenetic relationships of *Neophonus bruchi*, an anomalous south Andean staphylind (Coleoptera). *Systematic Entomology*, **12**, 389-404.

Thayer, M. K. (2003) Omaliinae of México: new species, combinations, and records (Coleoptera: Staphylinidae). *Memoirs on Entomology, International*, **17**, 311-358.

Tishechkin, A. K. (2007) A new species of *Ptomaphagus* (*Appadelopsis*) (Coleoptera: Leiodidae) from Great Smoky Mountains National Park, U.S.A. *Zootaxa*, **1478**, 61-64.

Topkara, E. T., M. Jäch, and A. Kasapoğlu (2011) *Ochthebius ustaoglui* sp. nov. (Coleoptera: Hydraenidae), a new species of the O. metallescens group from Turkey. *Zootaxa*, **2913**, 59-62.

Tóth, L. (1976) *Acrolocha caucasica* sp. n. mit einem Bestimmungsschlüssel der paläarktischen *Acrolocha*-Arten (Coleoptera, Staphylinidae). *Annales Historico-Naturales Musei Nationalis Hungarici*, **68**, 85-88.

Tóth, L. (1993) Holyvák VIII.—Staphylinidae VIII. VII kötet (Coleoptera II) 12. füzete. 29 ábrával. Magyarország Állatvilága [Fauna Hungariae]. 171, 1-71. Budapest: Akadémiai Kiadó. [In Hungarian]

Trizzino, M., L. Carnevali, S. de Felici, and P. Audisio (2013) A revision of *Hydraena* species of the "*Haenydra*" lineage (Coleoptera, Hydraenidae). *Zootaxa*, **3607**, 1-173.

Tronquet, M. (1998) *Oxypoda* (*s. tr.*) *pseudolongiceps* [*sic*], n. sp. (Coleoptera, Staphylinidae) commensal de la Marmotte des Alpes (*Marmotta marmotta* L.) dans les Pyrénées. *L'Entomologiste*, **54**, 135-140.

Tronquet, M. (1999) *Atheta* (*s. str.*) *burlei* n. sp. du Sud de la France (Coleoptera, Staphylinidae). *Nouvelle Revue d'Entomologie, (N.S.)*, **16**, 83-88.

Tronquet, M. (1999) Le groupe d'*Atheta hybrida* Sharp, *Atheta castellanensis* nov. sp. et caractères sexuels secondaires ♂ & ♀ chez *hybrida* Sharp et *burlei* Tronquet (Coleoptera, Staphylinidae). *Nouvelle Revue d'Entomologie, (N.S.)*, **16**, 327-333.

Tronquet, M. (1999) Sur quelques *Oxypoda* des collections A. Fauvel et G. Fagel; Derocala lucida n. sp. (Coleoptera, Staphylindiae). *Bulletin de la Société entomologique de France*, **104**, 167-181.

Tronquet, M. (2000) *Atheta gulosa* nov. sp., espèce nouvelle appartenant au groupe d'*Atheta* laticollis (Stephens) (Coleoptera, Staphylinidae) (16ème contribution à la connaissance des Staphylins). *Nouvelle Revue d'Entomologie, (N.S.)*, **17**, 365-370.

Tronquet, M. (2004) *Oxypoda subnitida* Mulsant & Rey, 1874 (Coleoptera, Staphylinidae, Aleocharinae) redescription. *Nouvelle Revue d'Entomologie, (N.S.)*, **20**, 361-365.

Uhlig, M. & J. Janák. (2009) *Erichsonius* (*Sectophilonthus*) *jelineki* sp. nov., the first representative of the genus from the *Seychelles* (Coleoptera: Staphylinidae: Staphylininae). *Acta Entomologica Musei Nationalis Pragae*, **49**, 695–710.

Uhlig, M. (1997) A new *Erichsonius* species from Cape Province, South Africa, with redescription of *Erichsonius* capensis (CAMERON) (Coleoptera: Staphylinidae). *Mitteilungen aus dem Zoologischen Museum in Berlin*, **73**, 265-278.

Ullrich, W. G. & J. M. Campbell (1974) A revision of the apterus-group of the genus *Tachinus* Gravenhorst (Coleoptera: Staphylinidae). *The Canadian Entomologist*, **106**, 627-644.

Vávra, J. (1998) Distribution of the genus *Choleva* (Coleoptera: Leiodidae: Cholevinae) in Turkey with description of three new species from Turkey and Syria. *Klapalekiana*, **34**, 243-262.

Veselova, E. M. (1990) Novye vidy *Tachinus* (Coleoptera, Staphylinidae) fauny SSSR. *Vestnik Zoologii*, **1990**, 13-17.

Vít, S. & P. Hlaváč (1997) *Euconnus* (subg. *Tetramelus*) from Caucasus and Turkey (Coleoptera: Scydmaenidae). *Entomological Problems*, **28**, 95-103.

Vít, S. & P. Hlaváč (1998) Review of *Euconnus* (*Tetramelus*) of the *reitteri* group (Coleoptera: Scydmaenidae). *Entomological Problems*, **29**, 139-147.

Vít, S. & P. Hlaváč (2005) New cavernicolous ant-like beetle of the genus *Euconnus* (subg. *Tetramelus*) from Croatia (Coleoptera: Scydmaenidae). *Natura Croatica*, **14**, 29-38.

Vít, S. (1989) Une espèce nouvelle d'*Euconnus* (*Tetramelus*) de l'Espagne boréo-occidentale (Coleoptera, Scydmaenidae). *Revue suisse de Zoologie*, **96**, 863-870.

Vít, S. (2006) Description of a highly specialised *Euconnus* from Thailand and its cranial particularities (Coleoptera: Staphylinoidea: Scydmaenidae). *Entomological Problems*, **36**, 67-74.

Vít, S. (2006) On *Euconnus cerastiventris* nom. nov. for *Euconnus chinensis* (Coleoptera: Staphylinoidea: Scydmaenidae). *Entomological Problems*, **36**, 75-78.

Vít, S. (2008) On *Scydmaenus schwendingeri* sp. n. and its tegumental secretory structures (Coleoptera: Staphylinoidea: Scydmaenidae). *Studies and reports of District Museum Prague-East, Taxonomical Series*, **4**, 267-276.

Vogel, J. (2003) Eine neue Art der Gattung *Atheta* Thomson aus Europa (Coleoptera, Staphylinidae, Aleocharinae). Enotomologische Blätter, *Entomologische Blätter für Biologie und Systematik der Käfer*, **99**, 95-98.

Vogel, J. (2006) Zur Kenntnis von *Atheta pfaundleri* G. Benick, 1940, einer neuen Art für die Fauna von Sachsen (Col., Staphylinidae). *Entomologische Nachrichten und Berichte*, **50**, 91-93.

Wang, C., Nishikawa, M., Perreau, M., Růžička, J. & Hayashi, Y. (2016) Revision of the genus *Ptomaphagus* Hellwig (Coleoptera, Leiodidae, Cholevinae) from Taiwan Island. *Zookeys*, **609**, 43-62.

Wang, C.-B. & H.-Z. Zhou. (2015) Taxonomy of the genus *Ptomaphaginus* Portevin (Coleoptera: Leiodidae: Cholevinae: Ptomaphagini) from China, with description of eleven new species. *Zootaxa*, **3941**, 301-338.

Wang, D., Z.-W. Yin & C.-X. Wang (2016) On a collection of *Batraxis* Reitter (Coleoptera: Staphylinidae: Pselaphinae) from Hainan Island, southern China. *Zootaxa*, **4109**, 1-15.

Wankowicz, J. (1869) Notices entomologiques. *Annales de la Société Entomologique de France*, (4)**9**, 411-422.

Wasmann, E. (1925) Die Ameisenmimikry: Ein exakter Beitrag zum Mimikryproblem und zur Theorie der Anpassung. *Abhandlungen zur theoretischen Biologie*, **19**, 1-164.

Watanabe, Y. & N.-N. Xiao (1996) A new species of the group of *Micropeplus sculptus* (Coleoptera, Staphylinidae) from Mt. Jizu Shan in Yunnan Province, Southwest China. *Edaphologia*, **57**, 1-6.

Watanabe, Y. & Y. Shibata (1961) A Revision of the genus *Elonium* Leach in Japan (Col. Staph.). *Journal of Agricultural Science*, *Tokyo*, **7**, 43-45.

Watanabe, Y. & Y. Shibata (2013) Redescription of *Syntomium japonicum* (Coleoptera, Staphylinidae), with some new collecting records. *The Japanese Journal of Systematic Entomology*, **19**, 221-226.

Watanabe, Y. & Y. Shibata. (1965) The staphylinid-beetles from Rishiri and Rebun Isls., Hokkaido, Japan, with descriptions of three new species. *Kontyû*, **33**, 317-323.

Watanabe, Y. & Z.-Y. Luo. (1992) New species of the genus *Lathrobium* (Coleoptera, Staphylinidae) from the Wu-yan-ling Nature Protective Area in Zhejiang Province, East China. *Elytra*, **20**, 47-56.

Watanabe, Y. (1975) A revision of the Japanese species of the genus *Micropeplus* Latreille (Coleoptera, Staphylinidae). *Kontyû*, **43**, 304-326.

Watanabe, Y. (1985) A revision of the Japanese species of *Derops* (Coleoptera, Staphylinidae). *Kontyû*, **53**, 436-451.

Watanabe, Y. (1987) A new *Ocypus* (Coleoptera, Staphylinidae) collected on the Northern Japanese Alps, Central Honshu, Japan. *Bulletin of the Gifu Prefectural Museum*, **8**, 43-47.

Watanabe, Y. (1989) A new species of the genus *Lathrimaeum* (Coleoptera, Staphylinidae) from Japan. *Elytra*, **17**, 53-55.

Watanabe, Y. (1990) A new *Micropeplus* (Coleoptera, Staphylinidae) from the Islands of Oki, West Japan. *Proceedings of the Japanese Society of Systematic Zoology*, **42**, 37-41.

Watanabe, Y. (1990) A revision of the Japanese species of the genus *Velleius* (Coleoptera, Staphylinidae). *Elytra*, **18**, 59-72.

Watanabe, Y. (1990) A taxonomic study on the subfamily Omaliinae from Japan (Coleoptera, Staphylinidae). *Memoirs of the Tokyo University of Agriculture*, **31**, 55-391.

Watanabe, Y. (1993) A new species of the genus *Derops* (Coleoptera, Staphylinidae) from the Russian Far East. *Japanese Journal of Entomology*, **61**, 557-561.

Watanabe, Y. (1996) A new species of the genus *Derops* (Coleoptera, Staphylinidae) from northern Vietnam. *Japanese Journal of Entomology*, **64**, 145-149.

Watanabe, Y. (1998) Two new apterous *Lathrobium* (Coleoptera, Staphylinidae) from the Ta-hsüeh Shan Mountains in Taiwan. *Elytra*, **26**, 303-311.

Watanabe, Y. (1999) Four new Anthophilus species of the Omaliinae (Coleoptera, Staphylinidae) from Mt. Miao'er Shan in Guangxi province, China. *Elytra*, **27**, 259-270.

Watanabe, Y. (1999) Two new species of the group of *Lathrobium pollens*/*brachypterum* (Coleoptera, Staphylinidae) from Zhejiang Province, East China. *Elytra*, **27**, 573-580.

Watanabe, Y. (1999) Two new subterranean staphylinids (Coleoptera) from East China. *Elytra*, **27**, 249-257.

Watanabe, Y. (2004) A remarkable new species of the genus *Micropeplus* (Coleoptera, Staphylinidae) from Hokkaido, Northeast Japan. *Elytra*, **32**, 79-84.

Watanabe, Y. (2004) *Lathrobium japonicum* and its new relatives (Coleoptera, Staphylinidae) from the Kuril island. *Biodiversity and Biogeography of the Kuril Island and Sakhalin*, (2004)**1**, 37-44.

Watanabe, Y. (2004) Two new species of the genus *Lesteva* (Coleoptera, Staphylinidae) from the island of Dôgo of the Oki island, West Japan. *Elytra*, **32**, 71-77.

Watanabe, Y. (2005) A new species of the genus *Lesteva* (Coleoptera, Staphylinidae) from Taiwan. *Elytra*, **33**, 30-33.

Watanabe, Y. (2005) Apterous *Lathrobium* (Coleoptera, Staphylinidae) from the Kii peninsula in Japan, 2. Group of *Lathrobium pollens*. *Elytra*, **33**, 589-601.

Watanabe, Y. (2005) Two new apterous *Lathrobium* (Coleoptera, Staphylinidae) from Taiwan. *Japanese Journal of Systematic Entomology*, **11**, 203-208.

Watanabe, Y. (2006) Apterous *Lathrobium* (Coleoptera, Staphylinidae) from the Kii peninsula in Japan, 3. Group of *Lathrobium brachypterum*. *Elytra*, **34**, 47-62.

Watanabe, Y. (2007) Two new species of the genus *Acrolocha* (Coleoptera, Staphy1inidae) from Japan. *Elytra*, **35**, 433-440.

Watanabe, Y. (2008) Two new species of the *Lathrobium* (Coleoptera: Staphylinidae) from Mt. Maya-san Hyôgo prefecture in western Honshu, Japan. *Special Publication of the Japan Coleopterological Society*, **(2)**, 183-190.

Watanabe, Y. (2010) *Lathrobium brachypterum* and its new relative (Coleoptera, Staphylinidae) from central Honshu, Japan. *Elytra*, **38**, 257-265.

Watanabe, Y. (2010) *Lathrobium densum* and its two new relatives (Coleoptera, Staphylinidae) from Western Honshu, Japan. *Elytra*, **38**, 73-82.

Watanabe, Y. (2010) More New species of the group of *Lathrobium nomurai* (Coleoptera, Staphylinidae) from eastern Shikoku, Japan. *The Japanese Journal of Systematic Entomology*, **16**, 63-69.

Weise, J. (1875) Drei neue europäische Staphylinen-Arten. *Deutsche Entomologische Zeitschrift*, **19**, 367-368.

Welch, R. C. (1969) *Aleochara verna* Say (Col., Staphylinidae) new to Britain. *The Entomologist*, **102**, 207-231.

Welch, R. C. (1990) *Aleochara* binotata Kr., not *A. verna* Say (Col.: Staphylinidae), a British insect. *The Entomologist's Record and Journal of Variation*, **102**, 225-226.

Welch, R. C. (1995) *Cypha tarsalis* Luze (Col.: Staphylinidae) new to Britain. Entomologist's Record, **107**, 185-187.

Wheeler, Q. D., and J. V. McHugh (1994) A new southern Appalachian species, *Dasycerus bicolor* (Coleoptera: Staphylinidae: Dasycerinae), from declining endemic fir forests. *Coleopterists Bulletin*, **48**, 265-271.

Willers, J. (2001) Neubeschreibungen und Synonyme chinesischer Arten der Gattung *Paederus* s.l. (Coleoptera: Staphylinidae). *Stuttgarter Beiträge zur Naturkunde Serie A (Biologie)*, **625**, 1-22.

Willers, J. (2001) Neue asiatische Arten der Gattung *Paederus* Fabricius s.l. aus der Sammlung des Naturhistorischen Museums Basel (Coleoptera, Staphylinidae). *Entomologica Basiliensia*, **23**, 287-309.

Willers, J. (2001) Zwei neue *Paederus* Fabricius, 1775, aus Ostnepal und Sikkim (Coleoptera: Staphylinidae). *Entomologische Zeitschrift*, **111**, 218-222.

Willers, J. (2002) Three new species of *Paederus* and related genera from Thailand, Vietnam and South China (Coleoptera: Staphylinidae). *Zoosystematica Rossica*, **11**, 155-162.

Wollaston, T. V. (1864) Catalogue of the coleopterous insects of the Canaries in the collection of the British Museum. xiii + 648 pp. London: British Museum.

Wu, J. & H.-Z. Zhou. (2010) Taxonomic study of subgenus *Plastus s. str.* (Coleoptera, Staphylinidae, Osoriinae) in China, with descriptions of five new species. *Zookeys*, **51**, 17-32.

Wu, J. (2006) Systematics of the Subfamily Osoriinae (Coleoptera: Staphylinidae) of China and Biodiversity Study of Saproxylic Beetles.

Wu, J., & H.-Z. Zhou. (2005) Taxonomy of the subgenus *Stigmatochirus* of *Priochirus* (Coleoptera, Staphylinidae, Osoriinae) from China. *Acta Zootaitonomica Sinica*, **30**, 304-303.

Wu, J., & H.-Z. Zhou. (2007) Phylogenetic analysis and reclassification of the genus *Priochirus* Sharp (Coleoptera: Staphylinidae: Osoriinae). *Invertebrate Systematics*, **21**, 73-107.

Yang, Z. & H.-Z. Zhou. (2010) Three new species of *Naddia* Fauvel (Coleoptera: Staphylinidae) from China. *Zootaxa*, **2531**, 1-14.

Yin, Z.-W. & L.-Z. Li (2015) Contribution to the knowledge of the genus *Syndicus* Motschulsky (Coleoptera: Staphylinidae: Scydmaeninae) in China. *Zootaxa*, **3918**, 415-423.

Yin, Z.-W. & L.-Z. Li (2016) Range extension for *Eupiestus spinifer* Fauvel in China (Coleoptera: Staphylinidae: Piestinae). *Zootaxa*, **4114**, 64-70.

Yin, Z.-W., R.-X. Jiang & H. Steiner (2016) Revision of the genus *Araneibatrus* (Coleoptera: Staphylinidae: Pselaphinae). *Zootaxa*, **4097**, 475-494.

Yin, Z.-W., X.-B. Song & L.-Z. Li (2014) A new species of *Syndicus* (*s. str*.) Motschulsky (Coleoptera, Staphylinidae, Scydmaeninae) from East China. *Zootaxa*, **3814**, 292-296.

Zanetti, A. (1979) Note sulle specie italiane del genere *Acrolocha* Thoms. con descrizione di una nuova specie (Col. Staphylinidae). *Bollettino del Museo Civico di Storia Naturale di Verona*, **5**, 547-553.

Zanetti, A. (1986) Contributo alla conoscenza delle Omaliinae europee con descrizione di nuove specie, note sinonimiche e designazione di lectotipi. *Studi Trentini di Scienze Naturali (Acta Biologica)*, **62**, 87-98.

Zanetti, A. (1987) Coleoptera. Staphylinidae. Omaliinae. In Fauna d'Italia 25: i-xii + 1-472. Bologna: Calderini.

Zanetti, A. (1987) Coleoptera. Staphylinidae. Omaliinae. In Fauna d'Italia 25: i-xii + 1-472pp. Bologna: Calderini.

Zanetti, A. (1987) Coleoptera. Staphylinidae. Omaliinae. In: Fauna d'Italia 25: i-xii + 1-472. Bologna: Calderini.

Zanetti, A. (1991) Contributo alla conoscenza degli *Eusphalerum* dell'Asia Centrale sovietica, con descrizione di due nuove specie (Coleoptera, Staphylinidae). *Nouvelle Revue d'Entomologie*, **7**, 289-301.

Zanetti, A. (1993) Contribution to the knowledge on Eastern Palaearctic *Eusphalerum* Kraatz with descriptions of new species (Coleoptera, Staphylinidae: Omaliinae). *Annales Historico-Naturales Musei Nationalis Hungarici*, **85**, 47-63.

Zanetti, A. (2002) Studies on *Omalium* Gravenhorst, 1802 from Turkey, Cyprus, and the Caucasus region, with notes on some European and Asian species (Coleoptera, Staphylinidae: Omaliinae). *Bollettino del Museo Civico di Storia Naturale di Verona*, **26**, 45-63.

Zanetti, A. (2004) Contributions to the knowledge of Eastern Palaearctic *Eusphalerum* Kraatz, 1857 (Coleoptera, Staphylinidae: Omaliinae). On some groups with setose parameres. *Bollettino del Museo Civico di Storia Naturale di Verona*, **28**, 51-95.

Zanetti, A. (2007) Contributions to the knowledge of Eastern Palaearctic *Eusphalerum* Kraatz, 1857 (Coleoptera, Staphylinidae: Omaliinae). New species and new records of the *fulvipenne* group. *Bollettino del Museo Civico di Storia Naturale di Verona*, **31**, 83-102.

Zanetti, A. (2008) Description of *Lesteva martinae* nov.sp. from Central Italy (Coleoptera, Staphylinidae: Omaliinae). *Linzer biologische Beiträge*, **40**, 993-997.

Zanetti, A. (2008) Synonymies in the European Omaliinae, with notes on distribution (Coleoptera: Staphylinidae). *Linzer biologische Beiträge*, **40**, 979-992.

Zanetti, A. (2014) Taxonomic revision of North American *Eusphalerum* Kraatz, 1857 (Coleoptera, Staphylinidae, Omaliinae). *Insecta Mundi*, **0379**, 1-80.

Zariquiey, R. (1917) Sobre el género *Troglocharinus* (Ins. Col.). *Treballs de la Institvció Catalana d'Història Natvral*, **3**, 283-294.

Zerche, L. (1988) Zur Taxonomie der Gattung *Pseudopsis* Newman, 1834 (Insecta, Coleoptera, Staphylinidae, Pseudopsinae). *Reichenbachia*, **25**, 151-155.

Zerche, L. (1992) Zur Taxonomie und Verbreitung der Gattung *Pseudopsis* Newman, 1834 (Coleoptera: Staphylinidae: Pseudopsinae). *Beiträge zur Entomologie*, **42**, 279-292.

Zerche, L. (1994) Die Revision der *Oxypoda*-Typen aus der Sammlung Claudius Rey im Musée Guimet d'Histoire naturelle de Lyon und einiger anderer Typen der Gattung sowie die Beschreibung von vier neuen *Oxypoda*-Arten (Coleoptera, Staphylinidae, Aleocharinae). - Coleoptera. *Schwanfelder Coleopterologische Mitteilungen*, **6**, 1-36.

Zerche, L. (1998) *Amblopusa magna* sp. n. - eine neue Art der amphipazifischen Gattung *Amblopusa* Casey, l893 aus dem Fernen Osten Rußlands und ihre Stellung im phylogenetischen System (Coleoptera: Staphylinidae, Aleocharinae, Liparocephalini). *Beiträge zur Entomologie*, **48**, 103-113.

Zerche, L. (1998) Phylogenetisch-systematische Revision der westpaläarktischen Gattung *Metopsia* Wollaston, 1854 (Coleoptera: Staphylinidae, Proteininae). *Beiträge zur Entomologie*, **48**, 3-101.

Zerche, L. (1998) Sieben neue *Pseudopsis*-Arten aus China mit einer Bestimmungstabelle der paläarktischen Arten (Coleoptera: Staphylinidae: Pseudopsinae). *Beiträge zur Entomologie*, **48**, 353-365.

Zerche, L. (2000) Eine neue *Pseudopsis*-Art aus Nepal und neue Funde von *Pseudopsis*-Arten (Coleoptera: Staphylinidae: Pseudopsinae). *Beiträge zur Entomologie*, **50**, 75-78.

Zerche, L. (2001) *Anthobium*- und *Deliphrosoma*-Arten und -Unterarten der Forschungs- und Sammelreise 2000 nach Bulgarien und Anmerkungen zu zwei anderen Arten der Balkanhalbinsel (Coleoptera: Staphylinidae: Omaliinae). *Beiträge zur Entomologie*, **51**, 341-354.

Zerche, L. (2002) *Leptusa*-Arten aus Bulgarien und Griechenland (Coleoptera, Staphylinidae, Aleocharinae). *Beiträge zur Entomologie*, **52**, 309-328.

Zerche, L. (2003) *Pseudopsis*-Studien 6: Neue Arten und neue Funde aus der Paläarktis, der Nearktis und der Neotropis (Insecta: Coleoptera: Staphylinidae: Pseudopsinae). *Entomologische Abhandlungen*, **60**, 161-169.

Zhao, C.-Y. & H.-Z. Zhou (2004) A new species of the genus *Micropeplus* (Coleoptera: Staphylinidae: Micropeplinae) in China. *Entomologia Sinica*, **11**, 235-238.

Zhao, C.-Y. & H.-Z. Zhou (2007) Two new species of the genus *Stenus* Latreille from China (Coleoptera: Staphylinidae: Steninae). *Entomologica Fennica*, **18**, 11-16.

Zhao, C.-Y., & H.-Z. Zhou (2006) Three new species of the genus *Stenus* Latreille (subgenus *Stenus s. str.*) from China (Coleoptera, Staphylinidae, Steninae). *Deutsche Entomologische Zeitschrift*, **53**, 282-289.

Zhao, C.-Y., W.-Z. Cai & H.-Z. Zhou. (2008) Two new *Stenus* (Hypostenus) species from China (Coleoptera, Staphylinidae, Steninae). *Zootaxa*, **1725**, 48-52.

Zhao, J.-Q., & L.-Z. Li (2013) New data on the genus *Derops* Sharp (Coleoptera, Staphylinidae, Tachyporinae) from China with description of two new species. *Zookeys*, **317**, 53-67.

Zhao, M.-J. (1996) A new species of the genus *Astenus* (Coleoptera, Staphylinidae) from Ryukyu Islands, Japan. *The Japanese Journal of Systematic Entomology*, **2**, 259-261.

Zhao, M.-J., L.-Z. Li & M. Sakai. (1999) Description of a new species of the genus *Astenus* (Coleoptera, Staphylinidae) from Honshu, Japan. *The Japanese Journal of Systematic Entomology*, **5**, 167-169.

Zhao, M.-J., L.-Z. Li & S.-H. Tang (2001) *Sepedophilus armatus*, a new Record for China (Coleoptera, Staphylinidae). *Journal of Shanghai Teachers University (Natural Sciences)*, **30**, 43-45.

Zhao, M.-J., L.-Z. Li & Y. Zhang. (2002) Description of a new species of the genus *Tachinus* (Coleoptera: Staphylinidae) from Sichuan, Southwest China. *Entomological Review of Japan*, **58**, 183-186.

Zhao, Z.-Y. & H.-Z. Zhou. (2010) Taxonomy of the genus *Indoquedius* Blackwelder (Coleoptera: Staphylinidae: Staphylininae) of China with description of four new species. *Zootaxa*, **2619**, 27-38.

Zheng, D.-L. & M.-J. Zhao (2014) Description of *Pella sichuanensis* sp. n. (Coleoptera: Staphylinidae: Aleocharinae) from Micang Mountain, Sichuan, China. *Zootaxa*, **3881**, 597-599.

Zheng, F.-K. & R.-R. Wang. (2007) Study on the genus *Indoquedius* Blackweldewr (Coleoptera, Staphylinidae, Staphylininae) from China. *Acta Zootaxonomica Sinica*, **32**, 76-79.

Zheng, F.-K. & S.-H. Pu. (2000) A new species of the genus *Omalium* Grevenhorst from China (Coleoptera: Staphylinldae: Omaliinae). *Journal of Sichuan Teachers College (Natural Science)*, **21**, 235-237.

Zheng, F.-K. & Y.-J. Li. (2010) New species and records of the subgenus *Oxyporus* of the genus *Oxyporus* from Sichuan and Ningxia, China (Coleoptera, Staphylinidae, Oxyporinae). *Acta Zootaxonomica Sinica*, **35**, 300-309.

Zheng, F.-K. (1987) A new species and a new record of genus *Trichophya* Mannerheim from China (Coleoptera: Staphylinidae, Trichophyinae). *Acta Entomologica Sinica*, **30**, 97-99.

Zheng, F.-K. (1992) Three new species of genus *Oxyporus* Fabricius from China (Coleoptera: Staphylinidae, Oxyporinae). *Acta Entomologica Sinica*, **35**, 326-330.

Zheng, F.-K. (1994) A new species and a new record of the genus *Neobisnius* Ganglbauer from China (Coleoptera: Staphylinidae, Staphylininae). *Acta Entomologica Sinica*, **37**, 213-214.

Zheng, F.-K. (1995) A new species of the genus *Gyrohypnus* Samouelle from Sichuan, China (Coleoptera: Staphylinidae, Xantholininae). *Acta Entomologica Sinica*, **38**, 220-221.

Zheng, F.-K. (1996) A new record of the genus *Bledius* Leach from Nei Mongol., China (Coleoptera: Staphylinidae, Oxytelinae). *Journal of Sichuan Teachers College (Natural Science)*, **17**, 8-10.

Zheng, F.-K. (1998) A preliminary study on the genus *Bledius* Leach (Coleoptera: Syaphylinidae, Oxytelinae) from China I. *gigantulus* group. *Acta Entomologica Sinica*, **41**, 171-173.

Zheng, F.-K. (1998) A taxonomic study on Staphylinidae in China, Oxytelinae: *Platystethus*. *Journal of Sichuan Teachers College (Natural Science)*, **19**, 249-256.

Zheng, F.-K. (2000) Notes on Chinese species of the genus *Platystethus* Mannerheim (Coleoptera: Staphylinidae: Oxytelinae). In Y.-L. Zhang (editor), Systematic and faunistic research on Chinese insects. Proceedings of the 5th National Congress of Insect Taxonomy. 110-116. Beijing: China Agriculture Press.

Zheng, F.-K. (2004) A new species of the *Bledius kosempoensis* group (Coleoptera: Staphylinidae, Oxytelinae) from China. *Coleopterists Bulletin*, **58**, 452-455.

Zheng, F.-K. (2012) A new species of the genus *Platysyethus* [*sic*]from China (Coleoptera, Staphylinidae, Oxytelinae). *Acta Zootaxonomica Sinica*, **37**, 341-344.

Zheng, F.-K., & C.-C. Wang (2012) A new species of the genus *Coprophilus* Latreille from China (Coleoptera, Staphylinidae, Oxytelinae). *Acta Zootaxonomica Sinica*, **37**, 763-766.

Zheng, F.-K., Y.-J. Li. & K. Liu. (2010) Six new species of the genus *Oxyporus* Fabricius from China (Coleoptera, Staphylinidae, Oxyporinae). *Acta Zootaxonomica Sinica*, **35**, 290-299.

Zhong, M., M.-J. Zhao, & L.-Z. Li (2009) Discovery of the genus *Acrolocha* Thomson (Coleoptera, Staphylinidae, Omalinae) of China with description of a new species. *Deutsche Entomologische Zeitschrift*, **56**, 295-297.

Zhou, Y.-L. & H.-Z. Zhou. (2011) Taxonomy of the genus *Metolinus* Cameron (Coleoptera, Staphylinidae, Staphylininae, Xantholinini) from China with description of three new species. *Zookeys*, **112**, 53-87.

Zhou, Y.-L. & H.-Z. Zhou (2013) Two new species of *Xanthophius* Motschulsky (Coleoptera: Staphylinidae, Staphylininae, Xantholinini) from China with notes on X. filum (Kraatz). *Zootaxa*, **3626**, 363-380.

Zhou, Y.-L. & H.-Z. Zhou. (2016) Taxonomy of the genus *Diochus* Erichson, 1839 (Coleoptera: Staphylinidae, Staphylininae, Diochini) in China with descriptions of four new species. *Zootaxa*, **4127**, 001-030.

Zhu, J.-W., L.-Z. Li & M.-J. Zhao (2005) A new species of the genus *Ischnosoma* from China (Coleoptera, Staphylinidae, Tachyporinae). *Acta Zootaxonomica Sinica*, **30**, 809-911.

Zoia, S. (1986) Catopidi raccolti nei dintorni di bologna con descrizione di una nuova specie del genere *Choleva* Latr. (Coleoptera). *Bollettino della Societa Entomologica Italiana, Genova*, **118**, 35-40.

Zoia, S. (1990) Nota sui *Namadeus* italiani con descrizione di *Namadeus italicus* n. sp. (Coleoptera, Cholevidae). *Bolle di Museo Regionale di Scienze Naturali, Torino*, **8**, 233-242.

Zou, S.-S. & Zhou, H.-Z. (2015) Taxonomy of the genus *Osorius* Guérin-Méneville (Coleoptera: Staphylinidae, Osoriinae) from China. *Zootaxa*, **4052**, 1-38.
