# Supplementary material for: Linking evolutionary mode to palaeoclimate change reveals rapid radiations of staphylinoid beetles in low-energy conditions
Source: Curr Zool. 2019 Oct 22;66(4):435–44. doi: 10.1093/cz/zoz053 (PMC7319441; doi:10.1093/cz/zoz053)
Supplement: zoz053_Supplementary_Data [file zoz053_supplementary_data.zip › zoz053-Suppl_Data/Appendix_S1_Tables.pdf]

Appendix S1: Tables

Table S1. Species richness (A. F. Newton unpublished database, 18-Sep-2016) and point estimates of divergence time of the most recent common ancestor (MRCA) of the family-level taxa. The subfamily in a box, absent from the phylogeny, is assigned to the sibling taxa. Abbreviations of taxon names are consistent with those shown in the figures.

**MRCA of (Super/Sub-)Family**	**Abbreviation**	**No. species**	**Age estimate (Ma)**
Root			208.90
Scarabaeoidea	SCARAB	32070*	184.65
Histeroidea	HIS	4657	181.15
Hydrophiloidea (*s. .str.*)	HYDP	3526	179.00
Staphylinoidea		69610	194.57
Ptiliidae	PTI	776	164.56
Hydraenidae	HYD	1961	181.50
Agyrtidae	AGY	72	174.00
Coloninae of Leiodidae	LEI (Coloninae)	155	186.90 †
Leiodidae (minus Coloninae)	LEI (minus Coloninae)	4006	170.24
Silphidae	SIL	187	170.97
Staphylinidae (including Silphidae)		62453	187.86 **
Apateticinae	APA	26	174.81 †
Habrocerinae	HAB	25	153.21
Trichophyinae	TRIC	18	164.98 †
Trigonurinae	TRIG	11	164.98 †
Pseudopsinae	PDP	55	142.40
Scaphidiinae	SCA	1712	165.72
Osoriinae (minus Osoriini)	OSO (minus Osoriini)	1336	107.42
Piestinae	PIE	103	162.99
Oxytelinae	OXYT	2101	168.38
Micropeplinae	MICP	87	151.52
Proteininae	PROT	231	153.74
Omaliinae-complex [Omaliinae, Empelinae, Glypholomatinae, Microsilphinae]	OMA+EMP+GLY+MICS	1543	170.53
Olisthaerinae	OLI	2	166.58 †
Mycetoporini of Tachyporinae	TAC (Mycetoporini)	424	139.35
Paederinae	PAE	7364	147.58
Staphylininae	STA	8201	160.41
Phloeocharinae (*Phloeocharis* related)	PHL (pt.1)	31	151.52
Tachyporinae (minus Mycetoporini)	TAC (minus Mycetoporini)	1142	165.00
Solieriinae	SOL	1	162.94 †
Scydmaeninae	SCY	5253	158.10
Megalopsidiinae	MEG	425	137.31 †
Leptotyphlinae	LEP	574	134.10 †
Oxyporinae	OXYP	128	125.00
Euaesthetinae	EUA	1155	135.54
Steninae	STE	2980	122.22

MRCA of (Super/Sub-)Family	Abbreviation	No. species	Age estimate (Ma)
Phloeocharinae (*Charhyphus* related)	PHL (pt.2)	30	136.43 †
Osoriinae (Osoriini)	OSO (Osoriini)	1031	117.61
Neophoninae	NEO	1	144.33 †
Dasycerinae	DAS	17	121.53
Pselaphinae + Protopselaphinae	PSE+PROP	9972	131.36
Aleocharinae	ALE	16474	139.40

* Richness of Scarabaeoidea is reported by Ślipiński *et al.* (2011), but not used in subsequent analyses.

** A Triassic fossil was described as the oldest staphylinid species, *Leehermania prorova* Chatzimanolis et al. (2012), but it is recently moved to Myxophaga by Fikáček et al. (in press). Currently there are no staphylinid fossils having been found in the Triassic strata.

† It is the age when the MRCA lineage split from the parental node.

Table S2. Fossils and age priors used in time calibration.

Node	MRCA or Split node	Fossil species	Min age (Ma)	Max age (Ma) †	Ref
1	Scarabaeoidea	*Antemnacrassa albosulcata*	157	252	(Nikolajev 2004; Grimaldi and Engel 2005; Lawrence and Ślipiński 2013)
2	Scarabaeoidea: *Glaresis*	*Glaresis orthochilus*	125	252	(Bai et al. 2010)
3	Histeroidea: *Histeridae*	*Pantostictus burmanicus*	98	252	(Poinar and Brown 2009)
4	Helophoridae: *Helophorus*	*Helophorus* (*Mesosperchus*) *tarsalis*	146	252	(Fikáček et al. 2012)
5	Hydrophilidae: *Helochares*	*Helochares undecimstriatum*	59	100.5	(Heer 1870; Birket-Smith 1977)
6	Ptiliidae	Undescribed [Lebanese amber]	135	252	(Poinar and Poinar 2008)
7	Hydraenidae	*Ochtebiites altus*	157	252	(Ponomarenko 1977; Ponomarenko 2003)
8	Hydraenidae: *Ochthebius*	*Ochthebius nitidulus*	59	100.5	(Heer 1870; Birket-Smith 1977)
9	Hydraenidae: *Hydraena*	*Hydraena reidiana*	1.8	100.5	(Lesne 1920)
10	Agyrtidae: Necrophilinae	Undescribed [Daohugou formation]	165	252	*
11	Leiodidae (minus Coloninae)	*Nemadus microtomographicus*	98	252	(Perreau and Tafforeau 2011)
12	Leiodidae: *Catops*	*Catops perkovskyi*	34	100.5	(Perreau 2012)
13	Leiodidae: *Ptomaphagus*	*Ptomaphagus germari*	34	100.5	(von Schlechtendal 1888)
14	Trigonurinae [split]	*Abolescus glabratus*	157	252	(Tikhomirova 1968)
15	Pseudopsinae	Undescribed [Yixian formation]	125	252	*
16	Scaphidiinae	*Scaphidiopsis aequivoca*	146	252	(Weyenbergh 1869)
17	Scaphidiinae: *Scaphidium*	*Scaphidium deletum*	12	100.5	(Heer 1847)
18	Scaphidiinae: Cypariini [split]	Undescribed [Burmese amber]	98	252	*
19	Scaphidiinae: *Scaphisoma*	*Scaphisoma gracile*	12	100.5	(Heer 1862)
20	Piestinae	*Paleosiagonium adaequatum*	125	252	(Yue et al. 2016)
21	Oxytelinae	*Mesoxytelus mandibularis*	157	252	(Tikhomirova 1968)
22	Oxytelinae: Euphaniini	*Protodeleaster glaber*	125	252	(Cai et al. 2013)
23	Oxytelinae: *Oxytelus*	*Oxytelus ominosus*	28	100.5	(Förster 1891)
24	Micropeplinae	*Protopeplus cretaceus*	98	252	(Cai and Huang 2014)
25	Proteininae	*Vetuproteinus cretaceus* [Burmese amber]	98	252	(Cai et al. 2016)
26	Omaliinae-complex	*Sinanthobium daohugouense*	165	252	(Cai and Huang 2013a)
27	Tachyporinae: Mycetoporini	*Glabrimycetoporus amoenus*	125	252	(Yue et al. 2009)
28	Staphylininae: Arrowiini	*Paleowinus rex*	125	252	(Solodovnikov et al. 2013)
29	Staphylininae (*s. str.*)	*Quedius cretaceus*	125	252	(Cai and Huang 2013b)
30	Staphylininae: *Algon*	*Algon spatiosus*	12	100.5	(Zhang 1989)
31	Staphylininae: *Platydracus*	*Platydracus breviantennatus*	34	100.5	(Cai et al. 2014a)
32	Phloeocharinae: *Phloeocharis*	*Phloeocharis agerata*	89	252	(Chatzimanolis et al. 2013)

Node	MRCA or Split node	Fossil species	Min age (Ma)	Max age (Ma) †	Ref
33	Tachyporinae (minus Mycetoporini)	Undescribed [Daohugou formation]	165	252	(Cai and Huang 2010)
34	Silphidae	Undescribed [Daohugou formation]	165	252	(Cai et al. 2014b)
35	Silphidae: Nicrophorinae	Undescribed [Yixian formation]	125	252	(Cai et al. 2014b)
36	Silphidae: *Necrodes*	*Necrodes primaevus*	34	100.5	(Beutenmüller and Cockerell 1908)
37	Silphidae: *Silpha*	*Silpha beutenmuelleri*	34	100.5	(Wickham 1914)
38	Scydmaeninae	Undescribed [Lebanese amber]	135	252	(Kirejtshuk and Azar 2013)
39	Scydmaeninae: *Stenichnus*	*Stenichnus carinulatus*	34	100.5	(Schaufuss 1890)
40	Scydmaeninae: *Euconnus*	*Euconnus fossilis*	34	100.5	(Franz 1976)
41	Oxyporinae	*Oxyporus yixianus*	125	252	(Yue et al. 2011)
42	Euaesthetinae	*Libanoeuaesthetus pentatarsus*	135	252	(Lefebvre et al. 2005)
43	Steninae	*Stenus inexpectatus*	101	252	(Schlüter 1978)
44	Osoriinae: Clavilispinina [split]	*Mesallotrochus longiantennatus*	98	252	(Cai and Huang 2015a)
45	Dasycerinae	*Protodasycerus aenigmaticus*	98	252	(Yamamoto 2016)
46	Pselaphinae: Faronitae	*Cretasonoma corinformibus*	109	252	(Peris et al. 2014)
47	Pselaphinae (minus Faronitae)	*Protrichonyx rafifrons*	98	252	(Parker 2016)
48	Aleocharinae	*Mesodeinopsis sp*	98	252	(Cai and Huang 2015b)
49	Aleocharinae: *Aleochara*	*Aleochara baltica*	34	100.5	(Paśnik and Kubisz 2002)
50	Aleocharinae: *Gyrophaena*	*Gyrophaena saxicola*	46	100.5	(Scudder 1876)

† We set the minimum bound of constrained MRCA by the recent limit age of the oldest fossils as we know, but the maximum bounds are not available, which are needed in the estimation by r8s program. So we assigned the maximum bounds using the boundary age between Upper and Lower Cretaceous (100.5 Ma) for the Cenozoic fossils and using that between Triassic and Permian (252 Ma) for the Mesozoic fossils respectively.

* The unpublished fossil species are fully identified by the third author (CYC) and deposited at Nanjing Institute of Geology and Palaeontology (NIGP), Chinese Academy of Sciences, Nanjing, China.

Table S3. Body size data of the sampled genera (for sources of body size data please see Appendix S4). The "body size" here means the ln (10 × body length), the "mean" of body size is the arithmetical mean of the body size of each genus. "SD" is short for standard deviation, and "SE" for standard error. Some genera have only one species or a single representative sampled, their standard deviation was set as 0, and the standard errors was estimated as the mean of the standard deviations of all the other genera (excluding the single species genera) in our database.

**Genus**	**Spp. sampled**	**Body size mean***	**Body size SD***	**Body size SE***
AGY: *Agyrtes*	8	3.826	0.177	0.062
AGY: *Apteroloma*	14	4.139	0.188	0.050
AGY: *Ipelates*	9	3.884	0.223	0.074
AGY: *Necrophilus*	3	4.781	0.199	0.115
ALE: *Acrotona*	29	3.191	0.173	0.032
ALE: *Aleochara*	62	3.878	0.244	0.031
ALE: *Aloconota*	14	3.563	0.223	0.060
ALE: *Amarochara*	8	3.510	0.243	0.086
ALE: *Amblopusa*	4	3.161	0.443	0.222
ALE: *Amischa*	9	2.974	0.105	0.035
ALE: *Atheta*	54	3.206	0.237	0.032
ALE: *Autalia*	29	3.005	0.250	0.046
ALE: *Baeostethus*	1	3.797	0	0.189
ALE: *Bolitochara*	4	3.543	0.137	0.069
ALE: *Brachyusa*	4	3.290	0.196	0.098
ALE: *Crataraea*	1	3.555	0	0.189
ALE: *Cypha*	23	2.584	0.221	0.046
ALE: *Deinopsis*	12	3.447	0.180	0.052
ALE: *Dexiogyia*	2	3.129	0.298	0.211
ALE: *Diaulota*	8	3.202	0.103	0.036
ALE: *Dilacra*	5	3.026	0.205	0.092
ALE: *Dinaraea*	6	3.502	0.158	0.065
ALE: *Drusilla*	26	3.833	0.098	0.019
ALE: *Encephalus*	6	3.340	0.257	0.105
ALE: *Gnypeta*	35	3.349	0.132	0.022
ALE: *Gymnusa*	9	3.968	0.103	0.034
ALE: *Gyrophaena*	101	2.878	0.319	0.032
ALE: *Halobrecta*	2	3.479	0.087	0.062
ALE: *Halorhadinus*	3	3.662	0.235	0.136
ALE: *Homalota*	5	3.019	0.253	0.113
ALE: *Ianmoorea*	1	3.512	0	0.189
ALE: *Leptusa*	84	3.123	0.218	0.024
ALE: *Liogluta*	18	3.572	0.189	0.045
ALE: *Liparocephalus*	4	3.799	0.068	0.034
ALE: *Myllaena*	20	3.162	0.256	0.057

Genus	Spp. sampled	Body size mean*	Body size SD*	Body size SE*
ALE: *Nopromaea*	9	3.496	0.156	0.052
ALE: *Oligota*	11	2.588	0.176	0.053
ALE: *Orphnebius*	21	3.795	0.173	0.038
ALE: *Oxypoda*	30	3.398	0.264	0.048
ALE: *Pella*	43	3.918	0.158	0.024
ALE: *Phloeopora*	6	3.423	0.196	0.080
ALE: *Pronomaea*	3	3.671	0.201	0.116
ALE: *Silusa*	2	3.504	0.032	0.023
ALE: *Stylogymnusa*	1	3.738	0	0.189
ALE: *Zyras*	13	4.094	0.175	0.049
APA: *Apatetica*	8	4.296	0.127	0.045
DAS: *Dasycerus*	16	2.929	0.151	0.038
EMP: *Empelus*	1	2.765	0	0.189
EUA: *Alzadaesthetus*	1	3.219	0	0.189
EUA: *Edaphus*	64	2.482	0.212	0.027
EUA: *Euaesthetus*	17	2.749	0.139	0.034
GLY: *Glypholoma*	5	3.329	0.085	0.038
HAB: *Habrocerus*	13	3.424	0.190	0.053
HAB: *Nomimocerus*	5	3.630	0.012	0.005
HYD: *Aulacochthebius*	2	2.527	0.183	0.129
HYD: *Hydraena*	125	3.000	0.224	0.020
HYD: *Limnebius*	7	2.882	0.380	0.144
HYD: *Meropathus*	8	3.150	0.135	0.048
HYD: *Ochthebius*	17	2.941	0.155	0.038
LEI: *Agathidium*	1	2.398	0	0.189
LEI: *Aglyptinus*	24	2.711	0.151	0.031
LEI: *Agyrtodes*	20	3.139	0.142	0.032
LEI: *Bathysciola*	3	2.988	0.151	0.087
LEI: *Camiarites*	2	3.463	0.211	0.149
LEI: *Catops*	22	3.717	0.196	0.042
LEI: *Choleva*	35	3.920	0.107	0.018
LEI: *Colon*	24	3.326	0.221	0.045
LEI: *Dissochaetus*	6	3.152	0.057	0.023
LEI: *Leiodes*	94	3.418	0.242	0.025
LEI: *Leptinus*	6	3.046	0.076	0.031
LEI: *Nemadus*	14	3.123	0.192	0.051
LEI: *Neopelatops*	1	3.689	0	0.189
LEI: *Platypsyllus*	1	3.020	0	0.189
LEI: *Ptomaphagus*	67	3.378	0.216	0.026
LEI: *Speonomus*	17	3.251	0.198	0.048
LEI: *Troglocharinus*	4	3.440	0.157	0.078
LEP: Neotyphlini *	30	2.617	0.174	0.032
MEG: *Megalopinus*	47	3.497	0.209	0.031
MICP: *Micropeplus*	13	3.031	0.216	0.060
MICS: *Microsilpha*	4	2.938	0.270	0.135
NEO: *Neophonus*	1	3.555	0	0.189

Genus	Spp. sampled	Body size mean*	Body size SD*	Body size SE*
OLI: *Olisthaerus*	2	4.028	0.094	0.067
OMA: *Acrolocha*	12	3.080	0.169	0.049
OMA: *Anthobium*	10	3.426	0.176	0.056
OMA: *Eusphalerum*	108	3.166	0.161	0.016
OMA: *Lesteva*	40	3.623	0.196	0.031
OMA: *Metacorneolabium*	22	2.400	0.139	0.030
OMA: *Olophrum*	15	3.770	0.141	0.036
OMA: *Omalium*	26	3.316	0.129	0.025
OMA: *Phyllodrepa*	16	3.389	0.253	0.063
OSO: *Indosorius*	7	3.931	0.331	0.125
OSO: *Lispinus*	3	3.872	0.285	0.164
OSO: *Nacaeus*	18	3.294	0.219	0.052
OSO: *Osorius*	11	4.348	0.289	0.087
OSO: *Plastus*	14	4.759	0.164	0.044
OSO: *Thoracophorus*	1	2.773	0	0.189
OXYP: *Oxyporus*	28	4.468	0.176	0.033
OXYT: *Anotylus*	24	3.591	0.286	0.058
OXYT: *Blediotrogus*	2	3.478	0.109	0.077
OXYT: *Bledius*	114	3.726	0.352	0.033
OXYT: *Carpelimus*	84	2.975	0.263	0.029
OXYT: *Coprophilus*	5	3.913	0.223	0.100
OXYT: *Coprostygnus*	3	4.074	0.142	0.082
OXYT: *Deleaster*	8	4.285	0.075	0.026
OXYT: *Ochthephilus*	62	3.595	0.148	0.019
OXYT: *Oxypius*	1	3.961	0	0.189
OXYT: *Oxytelus*	39	3.797	0.254	0.041
OXYT: *Platystethus*	30	3.472	0.269	0.049
OXYT: *Teropalpus*	2	3.644	0.230	0.163
OXYT: *Thinobius*	15	2.831	0.274	0.071
OXYT: *Thinodromus*	16	3.341	0.201	0.050
PAE: *Astenus*	7	3.673	0.155	0.059
PAE: *Domene*	8	4.433	0.124	0.044
PAE: *Lathrobium*	45	4.272	0.266	0.040
PAE: *Lithocharis*	16	3.720	0.115	0.029
PAE: *Lobrathium*	1	4.425	0	0.189
PAE: *Medon*	62	3.765	0.148	0.019
PAE: *Paederus*	51	4.454	0.164	0.023
PAE: *Pinophilus*	6	4.730	0.228	0.093
PAE: *Rugilus*	6	3.951	0.208	0.085
PAE: *Sunius*	36	3.432	0.177	0.030
PDP: *Pseudopsis*	50	3.553	0.173	0.024
PHL: *Charhyphus*	4	3.553	0.079	0.039
PHL: *Phloeocharis*	21	2.831	0.154	0.034
PIE: *Eupiestus*	6	3.552	0.391	0.160
PIE: *Piestus*	43	4.080	0.345	0.053
PIE: *Siagonium*	10	3.557	0.172	0.054

Genus	Spp. sampled	Body size mean*	Body size SD*	Body size SE*
PROT: *Megarthrus*	74	3.279	0.159	0.019
PROT: *Metopsia*	12	3.380	0.097	0.028
PROT: *Proteinus*	26	2.919	0.216	0.042
PSE: *Batrisodes*	2	3.013	0.142	0.101
PSE: *Brachygluta*	7	2.869	0.095	0.036
PSE: *Bryaxis*	166	2.769	0.142	0.011
PSE: *Euplectus*	12	2.705	0.120	0.034
PTI: *Acrotrichis*	47	2.153	0.185	0.027
PTI: *Cephaloplectus*	6	3.280	0.324	0.132
PTI: *Ptenidium*	12	2.236	0.081	0.023
PTI: *Pteryx*	4	1.921	0.207	0.104
PTI: *Ptilium*	13	1.884	0.075	0.021
PTI: *Ptinella*	10	2.113	0.242	0.076
SCA: *Baeocera*	43	2.699	0.217	0.033
SCA: *Brachynopus*	4	2.942	0.077	0.038
SCA: *Scaphidium*	44	3.893	0.246	0.037
SCY: *Cephennium*	13	2.303	0.247	0.069
SCY: *Euconnus*	34	2.734	0.183	0.031
SCY: *Neuraphes*	16	2.807	0.127	0.032
SCY: *Palaeostigus*	5	3.972	0.178	0.079
SCY: *Scydmaenus*	23	2.860	0.250	0.052
SCY: *Stenichnus*	68	2.697	0.153	0.019
SIL: *Dendroxena*	2	4.955	0.124	0.088
SIL: *Necrophila*	6	5.163	0.132	0.054
SIL: *Nicrophorus*	50	5.264	0.224	0.032
SIL: *Oiceoptoma*	9	4.944	0.043	0.014
SIL: *Oxelytrum*	8	4.951	0.225	0.080
SIL: *Ptomascopus*	1	5.190	0	0.189
SIL: *Silpha*	20	4.963	0.185	0.041
SIL: *Thanatophilus*	14	4.694	0.118	0.032
SOL: *Solierius*	1	3.219	0	0.189
STA: *Acylophorus*	46	4.181	0.201	0.030
STA: *Algon*	55	5.032	0.177	0.024
STA: *Anchocerus*	18	4.556	0.229	0.054
STA: *Antimerus*	9	5.052	0.070	0.023
STA: *Arrowinus*	4	5.255	0.390	0.195
STA: *Atanygnathus*	11	3.696	0.090	0.027
STA: *Atrecus*	12	4.040	0.108	0.031
STA: *Belonuchus*	20	4.439	0.208	0.047
STA: *Bolitogyrus*	10	4.453	0.079	0.025
STA: *Cafius*	13	4.187	0.224	0.062
STA: *Creophilus*	13	5.264	0.085	0.024
STA: *Dinothenarus*	11	4.986	0.251	0.076
STA: *Diochus*	13	3.742	0.243	0.067
STA: *Erichsonius*	44	3.934	0.267	0.040
STA: *Gabrius*	124	3.969	0.182	0.016

Genus	Spp. sampled	Body size mean*	Body size SD*	Body size SE*
STA: *Gastrisus*	2	5.030	0.150	0.106
STA: *Gyrohypnus*	15	4.253	0.125	0.032
STA: *Hadropinus*	1	5.165	0	0.189
STA: *Hadrotes*	2	4.976	0.049	0.034
STA: *Hesperopalpus*	3	4.401	0.078	0.045
STA: *Hesperus*	19	4.722	0.184	0.042
STA: *Heterothops*	2	3.761	0.049	0.035
STA: *Indoquedius*	16	4.471	0.190	0.048
STA: *Leptacinus*	15	3.777	0.228	0.059
STA: *Megalinus*	7	4.554	0.257	0.097
STA: *Metolinus*	102	3.918	0.252	0.025
STA: *Naddia*	9	5.186	0.209	0.070
STA: *Neobisnius*	48	3.797	0.157	0.023
STA: *Nordus*	34	4.578	0.110	0.019
STA: *Nudobius*	24	4.356	0.168	0.034
STA: *Ocypus*	86	5.064	0.178	0.019
STA: *Ontholestes*	21	4.787	0.115	0.025
STA: *Othius*	111	4.445	0.281	0.027
STA: *Phacophallus*	9	4.055	0.362	0.121
STA: *Philonthus*	219	4.351	0.317	0.021
STA: *Phucobius*	7	4.474	0.216	0.082
STA: *Platydracus*	11	4.953	0.169	0.051
STA: *Platyprosopus*	24	4.919	0.289	0.059
STA: *Quedius*	301	4.283	0.268	0.015
STA: *Remus*	4	3.848	0.184	0.092
STA: *Smilax*	4	5.043	0.044	0.022
STA: *Staphylinus*	7	5.221	0.171	0.065
STA: *Tasgius*	10	4.966	0.144	0.046
STA: *Thinocafius*	1	4.924	0	0.189
STA: *Thyreocephalus*	78	4.940	0.227	0.026
STA: *Tolmerinus*	13	4.660	0.204	0.056
STA: *Tympanophorus*	2	4.551	0.112	0.079
STA: *Xantholinus*	42	4.400	0.170	0.026
STA: *Xanthophius*	8	3.683	0.140	0.050
STA: *Xanthopygus*	3	4.970	0.262	0.151
STA: *Xenopygus*	6	4.702	0.215	0.088
STE: *Dianous*	104	4.009	0.255	0.025
STE: *Stenus*	41	3.631	0.239	0.037
TAC: *Coproporus*	7	3.176	0.224	0.085
TAC: *Derops*	14	3.833	0.078	0.021
TAC: *Lordithon*	41	3.909	0.270	0.042
TAC: *Sepedophilus*	19	3.582	0.317	0.073
TAC: *Tachinus*	68	3.814	0.336	0.041
TAC: *Tachyporus*	39	3.477	0.186	0.030
TRIC: *Trichophya*	13	3.224	0.333	0.092

* The body size of the tribe LEP: Neotyphlini was estimated from 13 out of 20 genera of this tribe: *Apheliotyphlus*, *Cainotyphlus*, *Eutyphlops*, *Heterotyphlus*, *Homeotyphlus*, *Macrotyphlus*, *Megatyphlus*, *Neotyphlus*, *Oreinotyphlus*, *Paramacrotyphlus*, *Prototyphlus*, *Telotyphlus*, *Xenotyphlus*.

Table S4. The taxa and sequences (GenBank accession numbers) used in phylogenetic analysis.

**(Super/sub)Family**	**Species**	***CAD***	***Wg***	***Cyt b***	***28S***	***18S***	***16S***
Scarabaeoidea	*Aphodius fimetarius*	KJ845318	---	---	KP419361	KP419009	EF487991
	Diphyllostoma fimbriata	KJ845325	KP250328	---	---	KP419082	KP250234
	Dynastes granti	---	EU677655	---	---	KP419087	JX994030
	Glaresis beckeri	---	KP250340	---	KP250415	KP250490	KP250245
	Glaresis ecostata	KJ845335	---	---	KP419464	KP419110	KP250246
	Melolontha melolontha	KJ845331	---	---	KJ845134	EF487702	EF487850
	Rhopaea magnicornis	KJ845183	---	NC_013252	---	---	NC_013252
	Trox gemmulatus	KJ845337	---	---	---	KP419326	EF570363
Histeroidea	*Abraeus globosus*	KJ845320	---	---	KJ844924	AY028338	---
	Bacanius globulinus	KJ845239	---	---	KJ844897	KP419018	---
	Dendrophilus punctatus	---	---	---	KJ844905	AJ810727	AM287076
	Euspilotus scissus	KJ845263	---	NC_018353	KP419459	KP419105	NC_018353
	Haeterius morsus	KJ845323	---	---	KJ844920	DQ124347	---
	Hister sp.1	---	---	---	KP419476	KP419121	---
	Hister unicolor	KJ845206	---	---	KJ844957	AY028360	---
	Hololepta plana	---	---	---	AJ810760	AJ810725	AM287075
	Hypocaccus lucidulus	KJ845326	---	---	KJ845032	---	---
	Margarinotus brunneus	---	---	---	AJ810761	AJ810726	AM287074
	Merohister jekeli	---	---	JF794630	---	---	JF794576
	Notosaprinus sp.1	---	---	---	KF802128	---	KF801800
	Omalodes grossus	KJ845193	---	---	KJ844916	AY028357	---
	Parepierus sp.1	KJ845316	---	---	KJ844896	---	---
	Peploglyptus belfragei	KJ845328	---	---	KJ844930	---	---
	Platysoma sp.1	KJ845264	---	---	KJ844892	---	---
	Quasimodopsis riedeli	KJ845329	---	---	KJ844898	---	---
	Saprinus planiusculus	---	---	JF794637	---	---	JF794585
	Saprinus semistriatus	---	---	JF794639	---	---	JF794587
	Syntelia histeroides	KJ845319	---	---	KP419658	KP419306	---
	Xylonaeus sp.1	---	---	---	KJ844879	---	---
Hydrophiloidea	*Andotypus ashworthi*	---	---	---	KP419352	KP419000	---
	Borborophorus tuberculus	---	---	---	KJ845053	KC935009	---
	Cercyon variegatus	---	---	---	KC992539	KC935011	KC992654
	Coelostoma orbiculare	---	---	---	KC992549	AJ810723	AM287072
	Cryptopleurum minutum	---	---	KT780640	---	---	KT780640
	Cylomissus glabratus	---	---	---	KC992556	KC935028	KC992663
	Dactylosternum marginale	KJ845266	---	---	KJ845048	---	KF801837
	Epimetopus sp.1	---	---	---	AJ810759	AJ810724	AM287060
	Georissus canalifer	---	---	---	KC992569	KC935041	KC992674
	Georissus sp.1	---	---	---	AJ810751	AJ810716	AM287061
	Georissus sp.2	KJ845180	---	---	KJ844998	---	---
	Helobata sp.1	---	---	---	KC992573	KC935045	KC992678
	Helochares lividus	---	---	KF755219	JX173154	AF201418	---
	Helochares obscurus	---	---	---	JX173147	AM287128	AM287069
	Helophorus aquaticus	---	---	---	AJ810749	AJ810714	AM287056
	Helophorus guttulus	---	---	---	AM287131	AM287123	AM287058
	Helophorus sp.1	KJ845298	---	---	KJ845004	KP419118	---
	Spercheus emarginatus	---	---	---	KJ845079	AJ810718	KC992709

(Super/sub)Family	Species	*CAD*	*Wg*	*Cyt b*	*28S*	*18S*	*16S*
	Spercheus sp.1	KJ845324	---	---	KP419644	KP419292	---
	Sphaeridium bipustulatum	---	---	NC_028612	KJ845093	AJ810722	KC992710
	Tropisternus ellipticus	---	EU797334	---	EU797397	EU797419	---
Staphylinoidea							
Ptiliidae	*Acrotrichis* sp.1	---	---	---	AJ810778	AJ810741	---
	Acrotrichis sp.2	---	---	---	KJ845098	KP418988	---
	Cephaloplectus sp.1	---	---	---	---	AY745600	---
	Limulodes parki	KJ845216	---	---	KJ844893	---	---
	Motschulskium sinuatocolle	---	---	---	KJ845034	---	---
	Nossidium sp.1	KJ845203	---	---	KP419544	KP419188	---
	Ptenidium laevigatum	---	---	---	EF213809	EF213788	---
	Ptenidium sp.1	---	---	---	HE970997	HE970959	HE971079
	Ptenidium sp.2	---	---	---	DQ202645	---	DQ202584
	Pteryx suturalis	---	---	---	---	EF362983	---
	Ptiliolum sp.1	---	---	---	HE971004	HE970967	HE971087
	Ptilium horioni	---	---	---	---	JN619331	---
	Ptinella errabunda	---	---	---	---	AY745602	---
Hydraenidae	*Adelphydraena orchymonti*	---	---	---	HM588527	HM588578	---
	Aulacochthebius sp.1	---	---	---	---	---	HF931432
	Aulacochthebius sp.2	---	---	---	---	---	HF931437
	Gymnochthebius laevipennis	---	---	---	KJ845090	---	---
	Gymnochthebius sp.1	---	---	---	KF802104	---	KF801777
	Hydraena californica	KJ845200	---	---	KP419481	KP419126	---
	Hydraena castanescens	---	---	---	HE970986	HE970940	HE971038
	Hydraena pulchella	---	---	---	HE970980	HE970934	HE971024
	Limnebius atomus	---	---	---	KT199120	KT199108	HF931394
	Limnebius mesatlanticus	---	---	---	KT199122	KT199110	HF931471
	Limnebius sp.1	KJ845240	---	---	KJ844953	---	---
	Meropathus sp.1	KJ845188	---	---	KP419520	KP419164	---
	Ochthebius minimus	---	---	---	AJ810766	AJ810731	DQ202575
	Ochthebius quadrifoveolatus	---	---	---	KT199127	KT199115	HF931446
	Ochthebius tivelunus	---	---	---	KT199128	KT199116	HF931420
	Orchymontia sp.1	KJ845205	---	---	KJ845113	---	---
Agyrtidae	*Agyrtes bicolor*	---	---	---	---	AY745595	---
	Apteroloma tahoecum	---	---	---	EF213806	AY745596	---
	Apteroloma tenuicorne	KJ845187	---	---	KJ844914	KP419010	---
	Ipelates latus	KJ845238	---	---	KP419489	KP419134	---
	Necrophilus hydrophiloides	KJ845185	---	AF021068	KP419538	---	---
	Zeanecrophilus thayerae	KJ845204	---	---	KJ844989	---	---
Leiodidae	*Afrocatops* sp.1	KJ845211	---	---	KJ845049	---	---
	Agathidium brevisternum	KJ845246	---	---	KJ845110	---	---
	Aglyptinus sp.1	KJ845248	---	---	KJ845081	---	---
	Agyrtodes ovatus	KJ845176	---	---	KP419346	KP418994	---
	Anillochlamys moroderi	---	---	HG915564	HG915484	HG915412	HG915637
	Anisotoma blanchardi	KJ845269	---	---	KJ845115	---	---
	Aranzadiella leizaolai	---	---	GU356804	---	JN619160	GU356747
	Astagobius angustatus	---	---	HG915565	HG915485	HG915413	HG915639
	Baronniesia delioti	---	---	HG915566	HG915486	HG915414	HG915640

(Super/sub)Family	Species	*CAD*	*Wg*	*Cyt b*	*28S*	*18S*	*16S*
	Bathyscimorphus byssinus	---	---	---	HG915487	HG915415	HG915641
	Bathysciola convena	---	---	HG915570	HG915490	HG915418	HG915646
	Bathysciola finismillennii	---	---	HG915571	HG915491	HG915419	HG915647
	Bathysciola liqueana	---	---	HG915575	HG915496	HG915425	HG915653
	Bathysciotes khevenhuelleri	---	---	HG915583	HG915503	HG915432	HG915660
	Bellesia espanyoli	---	---	GU356809	---	JN619168	GU356754
	Breuilia triangulum	---	---	---	HG915504	HG915433	HG915661
	Camiarites convexus	---	---	---	---	DQ337132	---
	Cantabrogeus nadali	---	---	HE663510	HE663525	HE663517	HE663531
	Catops paramericanus	KJ845268	---	---	KJ845015	---	---
	Catops picipes	---	---	---	AJ810769	AJ810734	FM209287
	Catops tristis	---	---	HE572882	---	---	GU356757
	Ceretophyes cenarroi	---	---	HG915584	HG915505	HG915434	HG915662
	Choleva angustata	---	---	---	HE572850	EF362994	HE576697
	Choleva cisteloides	---	---	HE572878	HE572851	HE572829	HE576698
	Choleva kocheri	---	---	---	HE572853	HE572831	HE576700
	Colon hirtale	KJ845192	---	---	KP419410	KP419056	---
	Cytodromus dapsoides	---	---	HG915585	HG915506	HG915435	HG915663
	Dasypelates gracilis	KJ845241	---	---	KJ844991	---	---
	Diaprysius caudatus	---	---	HG915586	HG915507	HG915436	HG915664
	Dictydiella turneri	KJ845209	---	---	KJ845000	---	---
	Dissochaetus oblitus	KJ845242	---	---	KJ844946	---	---
	Espanoliella urdialensis	---	---	HG915591	HG915512	HG915441	HG915669
	Euryspeonomus breuili	---	---	HG915592	HG915513	---	HG915670
	Fresnedaella lucius	---	---	HG915593	HG915514	HE663518	HE663534
	Inocatops sp.1	KJ845210	---	---	KJ845024	---	---
	Isereus colasi	---	---	HG915594	HG915515	HG915442	HG915671
	Josettekia mendizabali	---	---	GU356818	---	JN619164	GU356764
	Lagariella colominasi	---	---	GU356819	---	JN619250	GU356765
	Leiodes paludicola	KJ845294	---	---	KJ844959	---	---
	Leptinus testaceus	---	---	HE572877	HE572849	HE572827	HE576695
	Leptodirus hochenwarti	---	---	HG915596	HG915517	HG915444	---
	Nafarroa sorogainensis	---	---	HG915597	HG915518	HG915445	HG915673
	Nargus velox	---	---	---	AJ810770	AJ810735	GU356766
	Nemadus sp.1	---	---	---	KJ845018	---	---
	Neopelatops edwardsi	---	---	---	KJ844977	---	---
	Notidocharis uhagoni	---	---	HG915598	HG915520	HG915447	---
	Oresigenus jaspei	---	---	HG915599	HG915521	HG915448	HG915675
	Parabathyscia spagnoloi	---	---	HG915600	HG915525	HG915452	HG915679
	Paracatops sp.1	KJ845172	---	---	KJ844979	---	---
	Paranillochlamys catalonicus	---	---	HG915602	HG915527	HG915454	HG915681
	Parapropus sericeus	---	---	HG915603	HG915528	HG915455	HG915682
	Paraspeonomus vandeli	---	---	HE572881	HE572867	---	GU356771
	Parvospeonomus delarouzeei	---	---	HG915605	HG915530	JN619165	GU356772
	Phacomorphus sioberi	---	---	HE572874	HE572843	HE572821	HE576690
	Pinodytes delnorte	KJ845308	---	---	KJ844938	---	---
	Platycholeus opacellus	KJ845244	---	---	KJ844939	---	HE576701
	Platypsyllus castoris	KJ845312	---	---	KJ844966	---	---

(Super/sub)Family	Species	*CAD*	*Wg*	*Cyt b*	*28S*	*18S*	*16S*
	Prionochaeta opaca	KJ845186	---	---	KJ844885	KP419242	---
	Ptomaphagus sp.1	KJ845245	---	---	KJ844995	---	---
	Ptomaphagus tenuicornis	---	---	GU356829	---	---	GU356779
	Ptomaphagus troglodytes	---	---	GU356830	EF213808	---	GU356780
	Quaestus cisnerosii	---	---	HG915609	HG915535	HG915461	HG915689
	Quaestus noltei	---	---	HE572887	HE572869	JN619162	GU356781
	Sciaphyes sibiricus	---	---	HE572876	HE572847	HE572825	HF912605
	Sciodrepoides watsoni	---	---	HE572880	HE572857	JN619061	HE576705
	Silphopsyllus desmanae	---	---	---	HE572873	---	HE576719
	Speonemadus angusticollis	---	---	HE572888	HE572871	JN619271	GU356787
	Speonomites crypticola	---	---	HG915616	HG915544	HG915468	HG915696
	Speonomus alberti	---	---	HG915617	HG915545	HG915469	HG915697
	Speonomus bastideus	---	---	HG915618	HG915546	HG915470	HG915698
	Speonomus speluncarum	---	---	HG915628	HG915555	HG915476	HG915707
	Stygiophyes aldomai	---	---	HG915630	HG915557	HG915477	HG915708
	Trapezodirus orobios	---	---	HG915631	HG915558	HG915478	HG915709
	Troglocharinus ferreri	---	---	HF912509	HF912594	HF912582	HF912610
	Troglocharinus impellitierii	---	---	HF912514	HF912599	JN619167	HF912617
	Troglocharinus orcinus	---	---	HF912540	HF912600	HF912590	HF912642
	Troglodromus bucheti	---	---	HG915632	HG915559	HG915479	HG915710
	Zearagytodes sp.1	KJ845267	---	---	KJ844975	---	---
Staphylinidae							
Aleocharinae	*Acrotona assecla*	---	---	---	---	GQ981091	GQ980991
	Acrotona austiniana	---	---	---	---	GQ981093	GQ980993
	Aleochara (*Aleochara*) *collaris*	---	JX878792	---	JX878739	---	---
	Aleochara (*Aleochara*) *curtula*	---	---	---	KC132516	KC132432	JX536396
	Aleochara (*Xenochara*) *moerens*	---	---	---	KC132517	GQ981070	GQ980971
	Aleochara (*Tinotus*) *morion*	---	---	---	KC132519	JN619322	JX536398
	Aleochara (*Coprochara*) *sulcicollis*	KJ845249	---	---	KJ845103	---	---
	Aloconota cambrica	---	---	---	---	JN581777	JN581694
	Aloconota currax	---	---	---	---	JN581779	JN581696
	Aloconota gregaria	---	---	---	---	JN581780	JN581697
	Amarochara brevios	---	---	---	KC132554	KC132451	JX536419
	Amarochara sp.1	---	---	---	KC132555	KC132452	JX536420
	Amblopusa alaskana	---	---	---	---	FJ749907	---
	Amblopusa magna	---	---	---	KC132546	FJ749908	JX536414
	Amischa analis	---	---	---	---	JN581786	JN581702
	Amischa nigrofusca	---	---	---	---	JN581787	JN581703
	Apimela sp.1	---	---	---	KC132557	---	JX536422
	Apimela sp.2	---	---	---	KC132558	KC132454	JX536423
	Apimela sp.3	---	---	---	KC132556	KC132453	JX536421
	Atheta graminicola	---	---	---	KC132520	GQ981133	GQ981032
	Atheta laticeps	---	---	---	---	JN581840	JN581756
	Atheta vilis	---	---	---	EF213813	JN581789	JN581706
	Autalia longicornis	---	---	---	KC132528	EF213792	JX536402
	Baeostethus chiltoni	---	---	---	---	FJ749909	---
	Blepharhymenus corsicus	---	---	---	KC132560	KC132456	JX536425
	Blepharhymenus sp.1	---	---	---	KC132562	KC132458	JX536427

(Super/sub)Family	Species	*CAD*	*Wg*	*Cyt b*	*28S*	*18S*	*16S*
	Blepharhymenus sp.2	---	---	---	KC132561	KC132457	JX536426
	Bolitochara pulchra	---	---	---	KC132536	GQ981072	GQ980974
	Bolitochara sp.1	---	---	---	---	JN619243	---
	Brachyusa concolor	---	---	---	KC132563	KC132459	JX536428
	Cordalia obscura	---	---	---	KC132530	GQ981071	GQ980973
	Crataraea suturalis	---	---	---	KC132564	KC132461	JX536430
	Cypha imitator	---	---	---	---	JN619004	---
	Cypha longicornis	---	---	---	KC132543	KC132444	JX536411
	Dacrila fallax	---	---	---	KC132565	KC132462	JX536431
	Deinopsis erosa	---	---	---	EF213815	---	EU477777
	Devia prospera	---	---	---	KC132566	KC132463	JX536432
	Dexiogyia corticina	---	---	---	---	EF213793	---
	Diaulota densissima	---	---	---	KC132547	FJ749912	JX536415
	Diaulota uenoi	---	---	---	---	FJ749915	---
	Diaulota vandykei	---	---	---	---	FJ749916	---
	Dilacra luteipes	---	---	---	---	JN619321	---
	Dinaraea aequata	---	---	---	---	JN619007	GQ981040
	Dinarda hagensii	---	---	---	KC132567	KC132464	JX536433
	Dinarda maerkelii	---	---	---	KC132569	KC132466	JX536435
	Drusilla canaliculata	---	---	---	---	JN619013	GQ980982
	Drusilla sp.1	---	---	---	---	JN581795	JN581716
	Drusilla sp.2	---	---	---	---	JN581797	JN581717
	Encephalus lutilis	---	---	---	---	DQ337139	---
	Geostiba circellaris	---	---	---	KC132531	GQ981160	GQ981056
	Geostiba sp.1	---	---	---	KC132532	KC132438	JX536405
	Gnypeta caerulea	---	---	---	KC132572	KC132469	JX536438
	Gnypeta sp.1	---	---	---	KC132574	KC132470	---
	Gnypeta sp.2	---	---	---	KC132573	---	JX536439
	Gymnusa variegata	---	---	---	KC132533	GQ981068	GQ980969
	Gyrophaena congrua	---	---	---	KC132537	GQ981074	GQ980975
	Gyrophaena fasciata	---	---	---	---	GQ981075	GQ980977
	Gyrophaena sp.1	KJ845271	---	---	KJ845025	---	---
	Halobrecta algophila	---	---	---	KC132575	JN619083	JX536440
	Halobrecta halensis	---	---	---	---	GQ981172	GQ981065
	Halorhadinus aequalis	---	---	---	---	FJ749917	---
	Halorhadinus inaequalis	---	---	---	---	FJ749918	---
	Haploglossa villosula	---	---	---	KC132577	KC132472	JX536441
	Himalusa thailandensis	---	---	---	KC132534	KC132439	JX536406
	Homalota plana	---	---	---	KC132538	KC132441	JX536408
	Hoplandria lateralis	---	---	---	KC132541	GQ981079	GQ980980
	Hydrosmecta eximia	---	---	---	---	JN581812	JN581730
	Hydrosmecta gracilicornis	---	---	---	---	JN581813	JN581731
	Hydrosmecta valdieriana	---	---	---	---	JN581815	JN581733
	Ianmoorea zealandica	---	---	---	---	FJ749924	---
	Ilyobates bennetti	---	---	---	KC132578	KC132473	JX536442
	Ilyobates nigricollis	---	---	---	KC132579	KC132474	JX536443
	Ischnopoda objecta	---	---	---	KC132604	KC132497	JX536470
	Ischnopoderona gracilicornis	---	---	---	KC132580	KC132475	JX536444

(Super/sub)Family	Species	*CAD*	*Wg*	*Cyt b*	*28S*	*18S*	*16S*
	Leptusa kitazawai	---	---	---	---	FJ749926	---
	Leptusa opaca	---	---	---	---	---	EU477786
	Liogluta microptera	---	---	---	---	JN619011	GQ981041
	Liogluta nigropolita	---	---	---	---	GQ981144	---
	Liparocephalus cordicollis	KJ845250	---	---	KJ845042	FJ749919	JX536416
	Liparocephalus litoralis	---	---	---	---	FJ749920	---
	Lomechusa emarginata	---	---	---	KC132549	JN581817	JN581735
	Lomechusa pubicollis	---	---	---	---	JN581819	JN581737
	Meotica filiformis	---	---	---	KC132582	KC132477	JX536446
	Meronera venustula	---	---	---	KC132523	GQ981082	GQ980983
	Mniusa incrassata	---	---	---	KC132584	KC132478	JX536448
	Myllaena audax	---	---	---	KC132551	JN581833	JN581749
	Myrmecocephalus cingulatus	KJ845272	---	---	KJ845050	---	EU477795
	Myrmobiota sp.1	---	---	---	KC132586	KC132479	JX536450
	Neoisoglossa agnita	---	---	---	KC132581	KC132476	JX536445
	Neothetalia canadiana	---	---	---	KC132587	KC132480	JX536451
	Nopromaea sp.1	---	---	---	KC132614	KC132504	JX536478
	Nopromaea (*Stenectinobregma*) sp.2	---	---	---	KC132620	KC132509	JX536484
	Ocalea corsicana	---	---	---	KC132590	KC132483	JX536454
	Ocyusa picina	---	---	---	KC132592	KC132485	JX536456
	Ocyustiba sp.1	---	---	---	KC132593	KC132486	JX536457
	Oligota pumilio	---	---	---	KC132544	KC132445	JX536412
	Oreuryalea watanabei	---	---	---	KC132594	KC132487	JX536458
	Orphnebius draco	---	JX878794	---	JX878741	---	---
	Oxypoda alternans	---	---	---	KC132595	KC132488	JX536459
	Oxypoda longipes	---	---	---	KC132597	KC132490	JX536461
	Oxypoda opaca	---	---	---	KC132598	KC132491	JX536462
	Paraconosoma naviculare	---	---	---	KJ844888	---	---
	Paradilacra densissima	---	---	---	KC132601	KC132493	JX536465
	Parocyusa longitarsis	---	---	---	KC132607	KC132500	JX536473
	Pella caliginosa	---	---	---	KC132550	GQ981086	GQ980987
	Phloeopora sp.1	---	---	---	KC132602	KC132495	JX536468
	Placusa pinearum	---	---	---	---	JX845308	JX661722
	Placusa tachyporoides	---	---	---	---	GQ981090	GQ980990
	Pronomaea korgei	---	---	---	KC132616	KC132506	JX536480
	Silusa opaca	---	---	---	KC132539	KC132442	JX536409
	Silusida marginella	---	---	---	KC132540	GQ981078	GQ980978
	Stethusa dichroa	---	---	---	KC132524	GQ981154	GQ981051
	Strigota ambigua	---	---	---	KC132525	GQ981101	GQ981000
	Stylogymnusa sp.1	KJ845262	---	---	KJ844925	---	---
	Tachyusa gemma	---	---	---	KC132603	KC132496	JX536469
	Taxicera perfoliata	---	---	---	KC132526	KC132436	JX536400
	Taxicera truncata	---	---	---	KC132527	---	JX536401
	Tetralaucopora rubicunda	---	---	---	KC132608	KC132501	JX536474
	Thiasophila angulata	---	---	---	KC132609	KC132502	JX536475
	Typhloponemys sp.1	---	---	---	KC132618	KC132507	JX536482
	Zyras perdecoratus	---	---	---	---	JN581850	JN581765
	Zyras sp.1	---	---	---	---	JN581855	JN581772

(Super/sub)Family	Species	*CAD*	*Wg*	*Cyt b*	*28S*	*18S*	*16S*
Apateticinae	*Apatetica* spp. [*princeps*+sp.1]	KJ845302	---	---	KJ844960	FJ211609	---
Dasycerinae	*Dasycerus angulicollis*	---	---	AF021077	KJ844943	---	---
	Dasycerus carolinensis	---	---	---	KU238808	JN619307	---
Empelinae	*Empelus brunnipennis*	KJ845252	---	---	KJ845088	---	---
Euaesthetinae	*Alzadaesthetus furcillatus*	---	---	---	KJ845104	---	---
	Alzadaesthetus sp.1	---	---	---	---	FJ211611	---
	Edaphus sp.1	---	---	---	---	FJ211610	---
	Euaesthetus iripennis	KJ845212	---	---	KJ845116	---	---
	Euaesthetus ruficapillus	---	---	---	EF213816	AY745612	KJ144832
	Octavius sp.1	KJ845273	---	---	KJ845108	---	---
Glypholomatinae	*Glypholoma pecki*	KJ845189	---	---	KJ845077	---	---
	Proglypholoma aenigma	---	---	---	KJ845096	---	---
Habrocerinae	*Habrocerus capillaricornis*	KJ845226	---	AF021082	KJ844961	AY745613	JX536488
	Nomimocerus sp.1	---	---	---	KJ845092	---	---
Leptotyphlinae	Neotyphlini sp.1	KJ845217	---	---	KJ844993	FJ211607	---
Megalopsidiinae	*Megalopinus sanguinitriguttatus*	KJ845213	---	---	KJ844880	---	---
Micropeplinae	*Micropeplus fulvus*	---	---	---	EF213817	EF213794	---
	Micropeplus punctatus	---	---	---	KJ845026	---	---
	Micropeplus sp.1	---	---	---	HE572859	HE572837	HE576707
	Micropeplus sp.2	---	---	---	---	AY745611	---
Microsilphinae	*Microsilpha* sp.1	KJ845274	---	---	KJ845078	---	---
Neophoninae	*Neophonus bruchi*	KJ845275	---	---	KJ844883	JN619200	---
Olisthaerinae	*Olisthaerus megacephalus*	---	---	---	---	KC132513	JX536489
	Olisthaerus substriatus	KJ845276	---	---	KJ844932	---	---
Omaliinae	*Acrolocha sulcula*	---	---	---	---	AY745607	---
	Amphichroum sp.1	---	---	JX501014	---	---	---
	Anthobiomimus unicolor	---	---	---	KJ845069	---	---
	Anthobium sp.1	KJ845227	---	---	KJ845073	---	---
	Arpedium angulare	KJ845228	---	---	KJ845097	---	---
	Brathinus nitidus	KJ845229	---	---	KJ844981	---	---
	Eusphalerum sp.1	---	---	JX500997	KJ845017	---	---
	Lesteva sp.1	---	---	---	HE572858	HE572836	HE576706
	Metacorneolabium convexum	KJ845309	---	---	KJ845074	---	---
	Olophrum piceum	---	---	NC_028605	---	JN619029	NC_028605
	Omaliopsis ectopia	---	---	---	KJ844982	---	---
	Omalium caesum	---	---	---	EF213819	JN619067	---
	Pelecomalium sp.1	KJ845303	---	---	KJ845043	---	---
	Phyllodrepa devillei	---	---	---	---	JN619025	---
	Subhaida ingrata	---	---	---	KJ845100	---	---
Osoriinae	*Eleusis mutica*	KJ845230	---	---	KJ845091	---	---
	Indosorius sp.1	KJ845175	---	---	KJ844917	---	---
	Lispinus lineipennis	---	---	---	KJ844907	---	---
	Lispinus quadricollis	---	JX878783	---	JX878730	---	---
	Nacaeus longulus	---	JX878784	---	JX878731	---	---
	Neolispinus sp.1	KJ845177	---	---	KJ844994	---	---
	Osorius freyi	---	JX878785	---	JX878732	---	---
	Osorius tonkinensis	---	JX878786	---	JX878733	---	---
	Plastus magnificus	---	JX878787	---	JX878734	---	---

(Super/sub)Family	Species	*CAD*	*Wg*	*Cyt b*	*28S*	*18S*	*16S*
	Plastus miles	KJ845231	---	---	KJ844887	---	---
	Plastus unicolor	---	JX878788	---	JX878735	---	---
	Renardia nigrella	KJ845202	---	---	KP419613	KP419260	---
	Thoracochirus sp.1	KJ845317	---	---	KJ844913	---	---
	Thoracophorus costalis	---	---	---	KJ845059	---	---
Oxyporinae	*Oxyporus femoralis*	KT000243	KT022070	---	KT149213	---	---
	Oxyporus sp.1	---	JX878764	---	JX878711	---	---
	Oxyporus sp.2	---	JX878765	---	JX878712	---	---
	Oxyporus sp.3	KJ845293	---	---	KJ844985	---	---
Oxytelinae	*Anotylus* sp.1	---	---	---	KJ845070	---	---
	Blediotrogus sp.1	---	---	---	KJ845028	---	---
	Bledius sp.1	---	JX878774	---	JX878721	---	---
	Bledius tricornis	---	JX878775	---	JX878722	---	---
	Carpelimus bilineatus	---	---	---	EF213821	---	---
	Coprophilus pennifer	---	JX878776	---	JX878723	---	---
	Coprophilus striatulus	---	---	---	HE572863	HE572841	HE576713
	Coprostygnus sp.1	KJ845218	---	---	KJ845035	---	---
	Deleaster bactrianus	---	JX878777	---	JX878724	---	---
	Homalotrichus substriatus	---	---	---	KJ845055	---	---
	Ochthephilus sericinus	---	JX878778	---	JX878725	---	---
	Oxypius peckorum	---	---	---	KJ844984	---	---
	Oxytelus bengalensis	---	JX878779	---	JX878726	---	---
	Oxytelus incisus	---	JX878780	---	JX878727	---	---
	Oxytelus piceus	---	JX878781	---	JX878728	---	---
	Oxytelus varipennis	---	JX878782	---	JX878729	---	---
	Platystethus arenarius	---	---	---	KU238823	JN619068	---
	Sartallus signatus	KJ845219	---	---	KJ845027	---	---
	Teropalpus coloratus	---	---	---	KJ845008	---	---
	Thinobius sp.1	---	---	---	KJ845007	---	---
	Thinodromus sp.1	---	---	---	HE572861	HE572839	HE576709
Paederinae	*Achenomorphus corticinus*	KJ845277	---	AF021088	KJ844926	---	---
	Astenus lyonessius	---	---	---	EF213823	EF213796	---
	Cephalochetus sp.1	KJ845311	---	---	KJ844909	---	---
	Domene sp.1	---	---	---	LN624389	LN624388	---
	Gnathymenus apterus	KJ845278	---	---	KJ844955	---	---
	Homaeotarsus cinctus	KJ845279	---	---	KJ844910	---	---
	Hyperomma sp.1	KJ845300	---	---	KJ844900	---	---
	Lathrobium brunnipes	---	---	---	EF213824	AY745634	---
	Lathrobium sp.1	---	JX878761	---	JX878708	---	---
	Lithocharis nigriceps	---	---	---	---	JN619287	---
	Lobrathium sp.1	---	---	---	KJ844933	---	---
	Medon ripicola	---	---	---	---	JN619030	---
	Paederus littoralis	KJ845307	---	---	KJ844899	---	---
	Paederus littorarius	---	---	---	KJ844903	---	---
	Paederus sp.1	---	---	---	HE572860	HE572838	HE576708
	Palaminus sp.1	KJ845233	---	---	KJ844891	---	---
	Pinophilus latipes	KJ845234	---	---	KJ844889	---	---
	Rugilus geniculatus	---	---	---	---	JN619031	---

(Super/sub)Family	Species	*CAD*	*Wg*	*Cyt b*	*28S*	*18S*	*16S*
	Scopaeus sp.1	KJ845232	---	---	KJ844940	---	---
	Sunius confluentus	KJ845280	---	---	KJ844927	---	---
	Sunius (*Hypomedon*) *debilicornis*	---	---	---	---	JN619021	---
Pseudopsinae	*Pseudopsis* sp.1	---	---	---	EF213827	---	---
	Pseudopsis sulcata	---	---	---	DQ202651	AY745630	DQ202587
Phloeocharinae	*Charhyphus picipennis*	KJ845281	---	---	KJ844950	---	---
	Phloeocharis californica	KJ845282	---	---	KJ845082	---	---
	Phloeocharis diecki	---	---	---	HE572864	HE572842	HE576714
	Phloeocharis subtilissima	---	---	---	KC132624	AY745628	JX536490
Piestinae	*Eupiestus* sp.1	---	---	---	KJ845029	---	---
	Eupiestus sp.2	---	---	---	KJ845030	---	---
	Piestus extimus	---	---	---	KJ844987	---	---
	Siagonium americanum	---	---	---	KJ844988	---	---
Proteininae	*Alloproteinus* sp.1	KJ845283	---	---	KJ845283	---	---
	Anepius koebelei	---	---	---	KJ845051	---	---
	Austrorhysus sp.1	KJ845306	---	---	KJ845061	---	---
	Eupsorus sp.1	KJ845284	---	---	KJ845060	---	---
	Megarthroides sp.1	KJ845285	---	---	KJ845038	---	---
	Megarthrus antennalis	---	JX878770	---	JX878717	---	---
	Megarthrus sp.1	---	JX878771	---	JX878718	---	---
	Metopsia clypeata	---	---	---	EF213826	EF213797	---
	Nesoneus acuticeps	KJ845286	---	---	KJ845039	---	---
	Proteinus sp.1	KJ845287	---	---	KJ845062	---	---
	Silphotelus nitidus	KJ845288	---	---	KJ845063	---	---
Pselaphinae	*Adranes coecus*	---	KM350291	---	KM350313	KM350362	KM350408
	Adranes taylori	---	KM350292	---	KM350314	KM350363	KM350409
	Anaclasiger sp.1	---	KM350293	---	KM350315	KM350364	KM350410
	Andasibe sahondrae	---	KM350294	---	KM350316	KM350365	KM350411
	Apoderiger sp.1	---	KM350295	---	KM350317	KM350366	KM350412
	Articerodes thailandicus	---	---	---	KM350318	---	---
	Batrisodes lineaticollis	---	KM350296	---	KM350319	KM350367	KM350413
	Brachygluta helferi	---	---	---	---	---	DQ202573
	Brachygluta simplex	---	---	---	---	---	DQ202574
	Bryaxis curtisii	---	KM350297	---	KM350320	KM350368	KM350414
	Caccoplectus orbis	---	KM350298	---	KM350321	KM350369	KM350415
	Cerylambus reticulatus	---	KM350273	---	KM350322	KM350370	KM350416
	Claviger longicornis	---	KM350274	---	KM350323	KM350371	KM350417
	Claviger testaceus	---	KM350275	---	KM350324	KM350372	KM350418
	Conoplectus canaliculatus	---	---	AF021078	KM350328	KM350376	KM350421
	Ctenisodes sp.1	---	KM350278	---	KM350329	---	KM350422
	Curculionellus sp.1	---	KM350299	---	KM350330	KM350378	KM350423
	Diartiger fossulatus	---	KM350285	---	KM350331	KM350379	KM350424
	Diartiger spinipes	---	KM350300	---	KM350332	KM350380	---
	Dimerometopus sp.1	---	KM350301	---	KM350333	---	KM350425
	Euplectus karsteni	---	---	---	---	JN619154	---
	Faronus parallelus	---	---	---	KM350334	KM350381	KM350426
	Fustiger fuchsii	---	KM350279	---	KM350335	KM350382	KM350427
	Fustiger knausii	---	KM350303	---	KM350336	KM350383	KM350428

(Super/sub)Family	Species	*CAD*	*Wg*	*Cyt b*	*28S*	*18S*	*16S*
	Hadrophorus sp.1	---	KM350281	---	KM350339	KM350386	KM350432
	Lasinus mikado	---	KM350305	---	KM350340	KM350387	KM350433
	Leptoplectus pertenuis	---	---	---	KM350341	KM350388	KM350434
	Longacerus giraffa	---	KM350306	---	KM350342	KM350389	---
	Mastiger sp.1	---	KM350307	---	KM350343	KM350390	KM350435
	Mastiger sp.2	---	KM350282	---	KM350344	KM350391	---
	Megarafonus sp.1	---	---	---	KJ845071	---	---
	Melba thoracica	---	---	---	KM350345	KM350392	KM350437
	Micrapoderiger minutissimus	---	KM350283	---	KM350346	KM350393	---
	Miroclaviger sp.1	---	KM350284	---	KM350347	KM350394	KM350439
	Nearticerodes sp.1	---	KM350308	---	KM350348	---	KM350440
	Oropodes chumash	---	---	---	KM350349	KM350395	KM350441
	Pselaphogenius sp.1	---	KM350309	---	KM350350	KM350396	KM350442
	Pseudacerus sp.1	---	KM350286	---	KM350351	KM350397	KM350443
	Pyxidicerina sp.1	---	---	---	KM350352	KM350398	KM350444
	Radama sp.1	---	KM350310	---	KM350353	KM350399	---
	Rhytus sp.1	---	KM350287	---	KM350354	KM350400	KM350445
	Semiclaviger sikorae	---	KM350288	---	KM350355	KM350401	KM350446
	Sonoma sp.1	KJ845289	---	---	KJ845102	---	---
	Sonoma sp.2	---	---	---	KM350356	KM350402	KM350447
	Theocerus sp.1	---	KM350311	---	KM350357	KM350403	KM350448
	Tiracerus sp.1	---	---	---	---	---	KM350449
	Triartiger reductus	---	KM350289	---	KM350358	KM350404	KM350450
	Trichomatosus sp.1	---	KM350312	---	KM350359	KM350405	KM350451
	Tychobythinus sp.1	---	KM350290	---	KM350360	KM350406	KM350452
	Zethopsus sp.1	---	---	---	KM350361	KM350407	KM350453
Scaphidiinae	*Baeocera* sp.1	---	---	---	LC050261	---	---
	Baeoceridium celebense	---	---	---	LC050267	---	---
	Bironium amicale	---	---	---	LC050295	---	---
	Bironium sp.1	---	---	---	LC050266	---	---
	Brachynopus latus	---	---	JX412745	---	DQ337121	---
	Cyparium sp.1	---	---	---	LC050225	---	---
	Pseudobironium sp.1	---	---	---	LC050265	---	---
	Sapitia versicolor	---	---	---	LC050293	---	---
	Scaphicoma bidentia	---	---	---	LC050257	---	---
	Scaphicoma hiranoi	---	---	---	LC050247	---	---
	Scaphicoma quadrifasciata	---	---	---	LC050260	---	---
	Scaphidium celebense	---	---	---	LC050238	---	---
	Scaphidium picconii	---	---	---	LC050241	---	---
	Scaphidium quadrimaculatum	---	---	NC_028609	LC050228	AY745631	NC_028609
	Scaphidium sp.1	KJ845220	---	---	KP419633	KP419280	---
	Scaphisoma latitarse	---	---	---	LC050289	---	---
	Scaphisoma napu	---	---	---	LC050273	---	---
	Scaphisoma palu	---	---	---	LC050271	---	---
	Scaphium castanipes	KJ845291	---	---	KJ845099	---	---
	Scaphobaeocera sp.1	---	---	---	LC050243	---	---
	Scaphoxium sp.1	---	---	---	LC050262	---	---
	Termitoscaphium sp.1	---	---	---	LC050270	---	---

(Super/sub)Family	Species	*CAD*	*Wg*	*Cyt b*	*28S*	*18S*	*16S*
	Viturattella termitophilum	---	---	---	LC050268	---	---
	Xotidium sp.1	---	---	---	LC050263	---	---
	Xotidium sp.2	---	---	---	LC050264	---	---
Scydmaeninae	*Adrastia* sp.1	KJ845296	---	---	KJ845021	KP418991	---
	Cephennium gallicum	---	---	---	---	JN619060	---
	Cephennodes clavatus	KJ845270	---	---	KJ844878	---	---
	Clidicus sp.1	KJ845304	---	---	KJ844931	---	---
	Euconnus sp.1	KJ845199	---	---	KP419454	KP419100	---
	Euconnus sp.2	---	---	---	---	AY745604	---
	Neuraphes angulatus	---	---	---	---	EF362984	---
	Palaeostigus bifoveolatus	KJ845198	---	---	KP419567	KP419210	---
	Palaeomastigus sp.1	---	---	---	AJ810768	AJ810733	---
	Scydmaenus sp.1	---	---	---	---	AY745603	DQ202571
	Stenichnus godarti	---	---	---	---	JN619319	---
	Stenichnus scutellaris	---	---	---	---	EF362963	---
Silphidae	*Dendroxena sexcarinata*	---	AB285630	---	AB285567	---	AB285535
	Diamesus osculans	---	AB285649	---	AB285586	---	AB285554
	Necrodes littoralis	---	AB285631	---	AB285568	---	AB285536
	Necrodes nigricornis	---	AB285639	---	AB285576	---	AB285544
	Necrodes surinamensis	KJ845299	---	---	KJ845002	---	---
	Necrophila americana	KJ845197	AB285638	---	KP419537	KP419182	AB285543
	Necrophila (*Calosilpha*) *brunnicollis*	---	AB285645	---	AB285582	---	AB285550
	Necrophila (*Chrysosilpha*) *chloroptera*	---	AB285648	---	AB285585	---	AB285553
	Necrophila (*Eusilpha*) *jakowlewi*	---	AB285642	---	AB285579	KC413740	AB285547
	Necrophila (*Eusilpha*) *japonica*	---	AB285634	---	AB285571	---	AB285539
	Necrophila (*Calosilpha*) *kurosawai*	---	AB285646	---	AB285583	---	AB285551
	Nicrophorus concolor	EU147370	AB285650	---	AB285587	---	GQ118349
	Nicrophorus orbicollis	EU147397	AB285657	---	AB285594	---	AB285562
	Nicrophorus tomentosus	KJ845201	AB285656	---	KP419542	KP419186	AB285561
	Oiceoptoma nigropunctatum	---	AB285635	---	AB285572	---	AB285540
	Oiceoptoma noveboracense	KC821499	---	AF021083	KC821503	---	---
	Oiceoptoma subrufum	---	AB285632	---	AB285569	---	AB285537
	Oxelytrum discicolle	---	AB285647	---	AB285584	---	AB285552
	Phosphuga atrata	---	AB285636	---	AB285573	---	AB285541
	Ptomascopus morio	---	AB285654	---	AB285591	---	AB285559
	Silpha longicornis	---	AB285633	---	AB285570	---	AB285538
	Silpha perforata	---	AB285629	---	AB285566	KC413808	AB285534
	Silpha tristis	---	AB285637	---	AB285574	---	AB285542
	Thanatophilus rugosus	---	AB285641	---	AB285578	EF213790	AB285546
	Thanatophilus sinuatus	---	AB285643	JF794616	AB285580	---	AB285548
Solieriinae	*Solierius obscurus*	KJ845237	---	---	KJ844973	---	---
Staphylininae	*Acylophorus capensis*	KT000226	KT022067	---	KR559831	---	---
	Acylophorus sp.1	KT000228	---	---	---	---	---
	Acylophorus sp.2	---	GU377456	---	---	---	---
	Afroquedius sexpunctatus	KT000237	KT022060	---	KR559843	---	---
	Algon sp.1	KF178818	GU377457	---	GU377314	---	---
	Algon sp.2	KF178819	KF178732	---	KF178761	---	---
	Amblyopinus emarginatus	KT000257	GU377458	---	GU377315	---	---

(Super/sub)Family	Species	*CAD*	*Wg*	*Cyt b*	*28S*	*18S*	*16S*
	Anaquedius vernix	KT000225	GU377459	---	GU377316	---	---
	Anchocerus sp.1	KT000254	---	---	---	---	---
	Anisolinus sp.1	KT000230	KT022042	---	KR559828	---	---
	Antimerus punctipennis	KT000236	KT022045	---	KR559853	---	---
	Arrowinus minutus	KT000244	KT022057	---	---	---	---
	Arrowinus peckorum	---	---	---	KJ844962	---	---
	Arrowinus relictus	---	GU377462	---	KR559837	---	---
	Astrapaeus ulmi	KT000211	KT022049	---	KR559848	---	---
	Atanygnathus acuminatus	KF178817	GU377464	---	GU377319	---	---
	Atanygnathus sp.1	KJ845314	---	---	KJ844911	---	---
	Atanygnathus sp.2	---	GU377465	---	GU377320	---	---
	Atrecus macrocephalus	---	---	---	KJ844941	---	---
	Atrecus punctiventris	KT000259	GU377466	---	GU377321	---	---
	Belonuchus mimeticus	---	GU377467	---	GU377322	---	---
	Bolitogyrus bullatus	KT000205	KT022052	---	KR559833	---	---
	Bolitogyrus flavus	KT000207	KT022054	---	KR559835	---	---
	Bolitogyrus salvini	KT000206	KT022053	---	KR559834	---	---
	Cafius canescens	KJ845253	---	---	KJ844923	JF796498	JF796463
	Cafius seminitens	KF178812	GU377469	---	---	JF796514	JF796478
	Chroaptomus centralis	KT000252	KT149215	---	---	---	---
	Creophilus maxillosus	---	KF178730	---	KP419414	KP419060	GQ118385
	Dinothenarus saphyrinus	KT000238	GU377470	---	GU377324	---	---
	Diochus schaumi	KJ845221	---	---	KJ845045	---	---
	Diochus sp.1	KT000245	KT022068	---	---	---	---
	Diochus sp.1	---	GU377471	---	GU377325	---	---
	Elecatopselaphus sp.1	KF178822	GU377472	---	GU377326	---	---
	Elmas hanleyi	KF178823	KF178735	---	KF178764	---	---
	Emus hirtus	KF178816	KF178731	---	---	---	---
	Erichsonius nanus	KT000204	KT022050	---	KR559830	---	---
	Erichsonius sp.1	---	KT022051	---	KR559857	---	---
	Euryporus picipes	KT000219	KT022029	---	KR559847	---	---
	Gabrius keysianus	---	---	---	---	JN619018	---
	Gabrius osseticus	---	---	---	---	JN619329	---
	Gabrius unzenensis	---	---	---	---	JF796521	JF796485
	Gastrisus sp.1	---	GU377473	---	GU377327	---	---
	Gastrisus sp.2	KF178824	---	---	---	---	---
	Glenus flohri	KF178825	KF178736	---	KF178765	---	---
	Gyrohypnus spp. [*fracticornis*+sp.1]	---	---	KT780650	HM583930	KT780650	KT780650
	Hadropinus fossor	---	GU377474	---	GU377328	---	---
	Hadrotes crassus	---	GU377475	---	GU377329	JF796522	JF796486
	Hemiquedius ferox	KT000233	KT022039	---	KR559838	---	---
	Hesperopalpus sp.1	---	GU377490	---	GU377341	---	---
	Hesperosoma pederseni	KT000251	KT022041	---	---	---	---
	Hesperus inaequalis	KT000232	---	---	---	---	---
	Heterothops sp.1	KT000202	KT022062	---	KR559825	---	---
	Holisus sp.1	---	GU377477	---	GU377331	---	---
	Indoquedius aculeus	KT000249	---	---	---	---	---
	Indoquedius bicornutus	KT000248	KT022046	---	---	---	---

(Super/sub)Family	Species	*CAD*	*Wg*	*Cyt b*	*28S*	*18S*	*16S*
	Indoquedius sp.1	---	GU377478	---	---	---	---
	Korgella caucasica	---	KT022026	---	---	---	---
	Leptacinus harbinensis	---	JX878750	---	JX878697	---	---
	Linohesperus sp.1	KJ845301	---	---	KJ845031	---	---
	Lithocharodes sp.1	KT000197	KT022059	---	KR559824	---	---
	Maorothius brouni	KT000199	KT022061	---	KR559854	---	---
	Maorothius sp.1	---	---	---	KJ845014	---	---
	Megalinus sp.1	---	JX878751	---	JX878698	---	---
	Megalinus suffusus	---	JX878752	---	JX878699	---	---
	Metolinus shanicus	---	JX878753	---	JX878700	---	---
	Naddia sp.1	---	GU377479	---	GU377332	---	---
	Neobisnius occidentoides	KT000253	GU377481	---	---	---	---
	Neohypnus sp.1	KT000241	KT022066	---	KR559823	---	---
	Nordus fungicola	KF178828	GU377482	---	GU377334	---	---
	Nudobius cephalus	KJ845255	---	---	KJ845089	---	---
	Nudobius pugetanus	KT000198	GU377483	---	GU377335	---	---
	Ocypus olens	---	GU377484	---	GU377336	---	---
	Oligotergus sp.1	---	GU377485	---	GU377337	---	---
	Oligotergus sp.3	KF178830	KF178738	---	KF178767	---	---
	Ontholestes cingulatus	KJ845222	---	AF021089	KJ844922	---	---
	Ontholestes murinus	---	GU377486	---	GU377338	---	---
	Othius punctulatus	KT000200	GU377487	---	GU377339	---	---
	Othius sp.1	KT000201	KT022058	---	KR559858	---	---
	Pammegus ruficollis	KT000231	KT022055	---	KR559829	---	---
	Paratolmerus siamensis	KT000227	KT022056	---	KR559839	---	---
	Phacophallus japonicus	---	JX878754	---	JX878701	---	---
	Phacophallus sp.1	---	JX878755	---	JX878702	---	---
	Phanolinus sp.1	KF178831	KF178739	---	KF178768	---	---
	Philonthus caeruleipennis	KJ845292	---	---	KJ844963	---	---
	Philonthus spinipes	---	JX878748	---	JX878695	---	---
	Philothalpus bilobus	KF178820	KF178733	---	KF178762	---	---
	Philothalpus falini	KF178821	KF178734	---	KF178763	---	---
	Phucobius simulator	---	---	---	---	JF796527	JF796491
	Phucobius sp.1	---	---	---	---	JF796525	JF796489
	Phucobius sp.2	---	---	---	---	JF796526	JF796490
	Platydracus cinnamopterus	KT000239	GU377491	---	GU377342	---	---
	Platydracus maculosus	KJ845223	---	---	KJ844929	---	---
	Platyprosopus sp.1	KJ845224	---	---	KJ844956	---	---
	Plociopterus sp.1	KF178832	KF178740	---	KF178769	---	---
	Quediocafus insolitus	---	GU377494	---	GU377345	---	---
	Quediocafus taieriensis	KT000255	KT022064	---	---	---	---
	Quedius alticola	KT000221	KT022030	---	KR559846	---	---
	Quedius capucinus	KT000222	KT022031	---	KR559852	---	---
	Quedius (*Velleius*) *dilatatus*	KT000235	GU377504	---	GU377352	---	---
	Quedius molochinus	KF178814	GU377498	---	GU377348	---	---
	Remus corallicola	---	---	---	---	JF796528	JF796492
	Remus sericeus	---	---	---	---	JF796529	JF796493
	Scaponopselaphus mutator	---	KF178741	---	KF178770	---	---

(Super/sub)Family	Species	*CAD*	*Wg*	*Cyt b*	*28S*	*18S*	*16S*
	Smilax pilosa	KF178833	KF178742	---	KF178771	---	---
	Staphylinus spp. [*dimidiaticornis*+sp.1]	---	---	---	EF213828	AY745632	---
	Styngetus flavicaudus	KF178834	KF178743	---	KF178772	---	---
	Tasgius pedator	KT000240	GU377501	---	GU377349	JN619016	---
	Thinocafius insularis	---	---	---	---	JF796530	---
	Thinopinus pictus	KT000234	GU377502	---	GU377350	---	---
	Thyreocephalus annulatus	KT000196	KT022069	---	KR559822	---	---
	Thyreocephalus sp.1	---	JX878756	---	JX878703	---	---
	Tolmerinus sp.1	---	GU377461	---	GU377317	---	---
	Tympanophorus sp.1	---	GU377503	---	GU377351	---	---
	Xantholinus spp. [*linearis*+sp.1]	---	---	---	HM583940	AY745633	---
	Xanthophius angustus	---	JX878757	---	JX878704	---	---
	Xanthophius filum	---	JX878758	---	JX878705	---	---
	Xanthopygus cacti	---	EU797335	---	EU797398	AF002810	---
	Xanthopygus xanthopygus	---	---	---	KJ844952	---	---
	Xenopygus analis	KF178836	GU377506	---	GU377354	---	---
	Zackfalinus lamperos	---	KF178744	---	KF178773	---	---
Steninae	*Dianous nitidulus*	KJ845256	---	AF021084	KJ844934	FJ211608	---
	Dianous sp.1	---	JX878766	---	JX878713	---	---
	GenusNov sp.1	---	---	---	KJ844969	---	---
	Stenus fornicatus	---	---	---	EF213829	---	---
	Stenus sp.1	KJ845257	---	---	KJ844958	---	---
	Stenus sp.2	---	JX878767	---	JX878714	---	---
Tachyporinae	*Austrotachinus* sp.1	KJ845315	---	---	KJ845003	---	---
	Bryophacis punctatissimus	KJ845258	---	---	KJ844964	---	---
	Coproporus ventriculus	KJ845260	---	---	KJ845075	---	---
	Derops divalis	KJ845174	---	---	KJ845022	---	---
	Derops uenoi	---	---	---	KC132621	KC132510	JX536485
	Leucotachinus luteonitens	KJ845190	---	---	KP419504	KP419147	---
	Lordithon lunulatus	---	---	---	EF213830	---	---
	Lordithon scutellaris	KJ845259	---	---	KJ844935	---	---
	Lordithon thoracicus	---	---	---	KC132622	---	JX536486
	Sepedophilus bipunctatus	---	---	---	---	JN619026	---
	Sepedophilus castaneus	KJ845261	---	---	KJ845076	---	---
	Tachinus fumipennis	KJ845225	---	---	KJ845041	---	---
	Tachinus humeralis	---	---	---	---	JN619023	---
	Tachinus rufipes	---	---	---	---	KT204333	---
	Tachyporus chrysomelinus	---	---	---	---	KT204334	---
	Tachyporus hypnorum	---	---	---	HE572862	---	HE576710
	Tachyporus obtusus	---	---	---	---	KT204335	---
	Vatesus spp. [*clypeaus*+sp.1]	KT783382	KT783296	---	---	KC132511	JX536487
Trichophyinae	*Trichophya tarsalis*	KJ845297	---	---	KJ845094	---	---
Trigonurinae	*Trigonurus crotchii*	---	---	---	KJ845111	---	---

References

Bai M, Krell FT, Ren D, Yang X-K, 2010. A new, weil-preserved species of Glaresidae (Coleoptera: Scarabaeoidea) from the Jehol Biota of China. *Acta Geol Sin Engl Ed* **84**:676–679.

Beutenmüller W, Cockerell TDA, 1908. Taxonomic descriptions Pp. 14–19. *In:* Cockerell, T.D.A., Fossil insects from Florissant, Colorado. *Bull Am Mus Nat Hist* **24**:59–69.

Birket-Smith SJR, 1977. Fossil insects from Spitsbergen. *Acta Arctica* **19**:1–42.

Cai C, Newton AF, Thayer MK, Leschen RAB, Huang D, 2016. Specialized proteinine rove beetles shed light on insect–fungal associations in the Cretaceous. *Proc R Soc B-Biol Sci* **283**:20161439.

Cai C-Y, Huang D-Y, 2010. Current knowledge on Jurassic staphylinids of China (Insecta, Coleoptera). *Earth Sci Front* **17**:151–153.

Cai C-Y, Huang D-Y, 2013a. *Sinanthobium daohugouense*, a tiny new omaliine rove beetle (Coleoptera: Staphylinidae) from the Middle Jurassic of China. *Can Entomol* **145**:496–500.

Cai C-Y, Huang D-Y, 2013b. A new species of small-eyed *Quedius* (Coleoptera: Staphylinidae: Staphylininae) from the Early Cretaceous of China. *Cretac Res* **44**:54–57.

Cai C-Y, Huang D-Y, 2014. The oldest micropepline beetle from Cretaceous Burmese amber and its phylogenetic implications (Coleoptera: Staphylinidae). *Naturwissenschaften* **101**:813–817.

Cai C-Y, Huang D-Y, 2015a. The oldest osoriine rove beetle from Cretaceous Burmese amber (Coleoptera: Staphylinidae). *Cretac Res* **52**:495–500.

Cai C-Y, Huang D-Y, 2015b. The oldest aleocharine rove beetle (Coleoptera, Staphylinidae) in Cretaceous Burmese amber and its implications for the early evolution of the basal group of hyper-diverse Aleocharinae. *Gondwana Res* **28**:1579–1584.

Cai C-Y, Newton AF, Huang D-Y, Tang L, 2014a. A new species of *Platydracus* Thomson, 1858 (Coleoptera, Staphylinidae, Staphylininae) from the upper Eocene Florissant beds, Colorado, USA. *Palaeoworld* **23**:321–326.

Cai C-Y, Thayer MK, Engel MS, Newton AF, Ortega-Blanco J, Wang B, Wang X-D, Huang D-Y, 2014b. Early origin of parental care in Mesozoic carrion beetles. *Proc Natl Acad Sci U S A* **111**:14170–14174.

Cai C-Y, Thayer MK, Huang D-Y, Wang X-D, Newton AF, 2013. A basal oxyteline rove beetle (Coleoptera: Staphylinidae) from the Early Cretaceous of China: Oldest record for the tribe Euphaniini. *Comptes Rendus Palevol* **12**:159–163.

Chatzimanolis S, Grimaldi DA, Engel MS, Fraser NC, 2012. *Leehermania prorova*, the Earliest Staphyliniform Beetle, from the Late Triassic of Virginia (Coleoptera: Staphylinidae). *Am Mus Novit* **3761**:1–28.

Chatzimanolis S, Newton AF, Soriano C, Engel MS, 2013. Remarkable stasis in a Phloeocharine rove beetle from the Late Cretaceous of New Jersey (Coleoptera, Staphylinidae). *J Paleontol* **87**:177–182.

Fikáček M, Beutel RG, Cai CY, Lawrence JF, Newton AF, Solodovnikov A, Ślipiński A, Thayer MK, Yamamoto S. Reliable placement of beetle fossils via phylogenetic analyses - Triassic Leehermania as a case study (Staphylinidae or Myxophaga?). *Syst Entomol* (in press).

Fikáček M, Prokin A, Angus RB, Ponomarenko A, Yue Y-L, Ren D, Prokop J, 2012. Revision of Mesozoic fossils of the helophorid lineage of the superfamily Hydrophiloidea (Coleoptera: Polyphaga). *Acta Entomol Musei Natl Pragae* **52**:89–127.

Förster B, 1891. Die Insekten des "Plattigen Steinmergels" von Brunstatt. *Abh Zur Geol Spec Von Elsass-Lothr* **3**:333–594.

Franz H, 1976. Scydameniden (Coleoptera) aus dem baltischen Bernstein. *Verhandlungen Zool-Bot Ges Wien* **115**:80–85.

Grimaldi DA, Engel MS, 2005. Evolution of the Insects. Cambridge, New York, Melbourne, Madrid, Cape Town, Singapore, Sao Paulo: Cambridge University Press

Heer O, 1847. Die Insektenfauna der Tertiärgebilde von Oeningen und von Radoboj in Croatien. Erster Theil: Käfer. Leipzig: W. Engelmann

Heer O, 1862. Beiträge zur Insektenfauna Oeningens. Harlem: Erben Loosjes

Heer O, 1870. Die Miocene flora und fauna Spitzbergens. *Kongliga Sven Vetensk-Akad Handl* **8**:1–98.

Kirejtshuk AG, Azar D, 2013. Current knowledge of Coleoptera (Insecta) from the Lower Cretaceous Lebanese amber and taxonomical notes for some Mesozoic groups. *Terr Arthropod Rev* **6**:103–134.

Lawrence JF, Ślipiński SA, 2013. Australian Beetles. Collingwood, Victoria: CSIRO Publishing

Lefebvre F, Vincent B, Azar D, Nel A, 2005. The oldest beetle of the Euaesthetinae (Staphylinidae) from Early Cretaceous Lebanese amber. *Cretac Res* **26**:207–211.

Lesne P, 1920. Quelques insectes du Pliocène supérieur du Comté de Durham II. *Bull Muséum Natl Hist Nat* **26**:484–488.

Nikolajev GV, 2004. Mezozoyskiy etap v evolyutsii podsemeystva Aclopinae (Coleoptera, Scarabaeidae). *Tethys Entomol Res* **10**:33–46.

Parker J, 2016. Emergence of a superradiation: Pselaphine rove beetles in mid-Cretaceous amber from Myanmar and their evolutionary implications. *Syst Entomol* **41**:541–566.

Paśnik G, Kubisz D, 2002. A new genus and new species of the Staphylinidae (Coleoptera) from Baltic amber. *Eur J Entomol* **99**:353–361.

Peris D, Chatzimanolis S, Delclòs X, 2014. Diversity of rove beetles (Coleoptera: Staphylinidae) in Early Cretaceous Spanish amber. *Cretac Res* **48**:85–95.

Perreau M, 2012. Description of a new genus and two new species of Leiodidae (Coleoptera) from Baltic amber using phase contrast synchrotron X-ray microtomography. *Zootaxa* **3455**:81–88.

Perreau M, Tafforeau P, 2011. Virtual dissection using phase-contrast X-ray synchrotron microtomography: Reducing the gap between fossils and extant species. *Syst Entomol* **36**:573–580.

Poinar G, Brown AE, 2009. *Pantostictus burmanicus*, a new genus and species of Cretaceous beetles (Coleoptera: Hydrophiloidea: Histeridae) in Burmese amber. *Proc Entomol Soc Wash* **111**:38–46.

Poinar GOJr, Poinar R, 2008. What Bugged the Dinosaurs? Insects, Disease, and Death in the Cretaceous. Princeton: Princeton University Press

Ponomarenko AG, 1977. Suborder Adephaga, Polyphaga incertae sedis, infraorder Staphyliniformia, in Mezozoiskie zhestkokryiye [Mesozoic Coleoptera]. *Tr Paleontol Instituta Akad Nauk SSSR* **161**:17–119.

Ponomarenko AG, 2003. Ecological evolution of beetles (Insecta: Coleoptera). *Acta Zool Cracoviensia* **46**:319–328.

Schaufuss LW, 1890. Die Scydmaeniden des baltischen Bernsteines. *Nunquam Otiosus* **3**:561–586.

von Schlechtendal D, 1888. Mittheilungen über die in der Sammlung aufbewahrten Originale zu Germar's: "Insekten in Bernstein eingeschlossen" mit Rücksicht auf Giebels "Fauna der Vorwelt." *Z Für Naturwissenschaften* **61**:473–491.

Schlüter T, 1978. Zur Systematik und Palökologie harzkonservierter Arthropoda einer Taphozönose aus dem Cenomanium von NW-Frankreich. *Berl Geowiss Abh Reihe A* **9**:1–150.

Scudder SH, 1876. Fossil Coleoptera from the Rocky Mountain Tertiaries. *Bull U S Geol Geogr Surv Territ* **II**:77–87.

Ślipiński SA, Leschen RAB, Lawrence JF, 2011. Order Coleoptera Linnaeus, 1758. *In*: Zhang, Z.-Q. (Ed.) Animal biodiversity: An outline of higher-level classification and survey of taxonomic richness. *Zootaxa* **3148**:203–208.

Solodovnikov AYu, Yue Y, Tarasov S, Ren D, 2013. Extinct and extant rove beetles meet in the matrix: Early Cretaceous fossils shed light on the evolution of a hyperdiverse insect lineage (Coleoptera: Staphylinidae: Staphylininae). *Cladistics* **29**:360–403.

Tikhomirova AL, 1968. Zhuki-stafilinidy Yuri Karatau [Rove beetles from the Jurassic of Karatau]. In: Rodendorf BB, editor. *Yurskie nasekomye Karatau [Jurassic insects of Karatau]*. Moscow: Akademia Nauk SSSR, Otdelenie obshej biologii/ Izdatelstvo Nauka. pp. 139–154.

Weyenbergh H, 1869. Sur les Insectes fossiles du Calcaire Lithographique de la Bavière, qui se trouvent au Musée Teyler. *Arch Mus Teyler* **2**:1–48.

Wickham HF, 1914. New Miocene Coleoptera from Florissant. *Bull Mus Comp Zool Harv Univ* **58**:423–494.

Yamamoto S, 2016. The first fossil of dasycerine rove beetle (Coleoptera: Staphylinidae) from Upper Cretaceous Burmese amber: Phylogenetic implications for the omaliine group subfamilies. *Cretac Res* **58**:63–68.

Yue Y, Gu J-J, Yang Q, Wang J, Ren D, 2016. The first fossil species of subfamily Piestinae (Coleoptera: Staphylinidae) from the Lower Cretaceous of China. *Cretac Res* **63**:63–67.

Yue Y-L, Ren D, Solodovnikov A, 2011. The oldest fossil species of the rove beetle subfamily Oxyporinae (Coleoptera: Staphylinidae) from the Early Cretaceous (Yixian Formation, China) and its phylogenetic significance. *J Syst Palaeontol* **9**:467–471.

Yue Y-L, Zhao Y-Y, Ren D, 2009. *Glabrimycetoporus amoenus*, a new tachyporine genus and species of Mesozoic Staphylinidae (Coleoptera) from Liaoning, China. *Zootaxa* **2225**:63–68.

Zhang J-F, 1989. Fossil insects from Shanwang. Jinan, China: Shandong Science and Technology Press
