# Supplementary material for: Linking evolutionary mode to palaeoclimate change reveals rapid radiations of staphylinoid beetles in low-energy conditions
Source: Curr Zool. 2019 Oct 22;66(4):435–44. doi: 10.1093/cz/zoz053 (PMC7319441; doi:10.1093/cz/zoz053)
Supplement: zoz053_Supplementary_Data [file zoz053_supplementary_data.zip › zoz053-Suppl_Data/Appendix_S2_Figures.pdf]

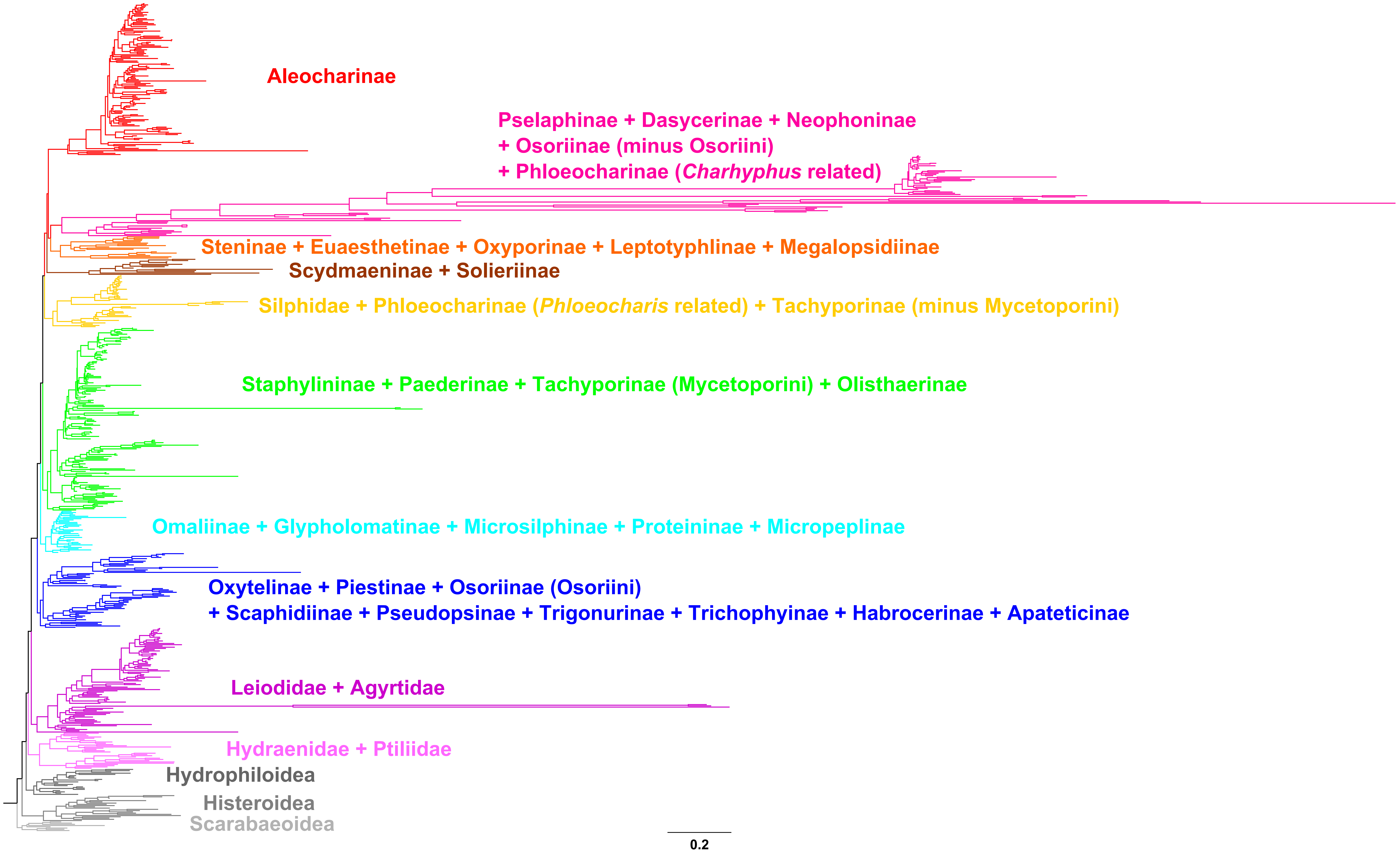

Figure S1. Phylogram of the phylogeny with branch length proportional to the number of nucleotide substitutions per site. Different colors mark the "clades" cited in the Phylogenetic analysis in Appendix S3.

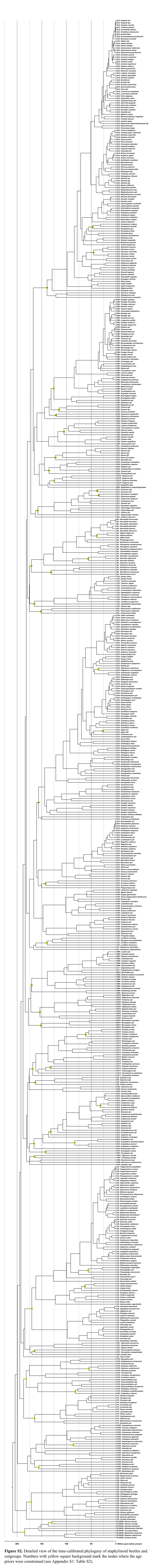

Figure S2. Detailed view of the time-calibrated phylogeny of staphylinoid beetles and outgroups. Numbers with yellow square background mark the nodes where the age priors were constrained (see Appendix S1: Table S2).

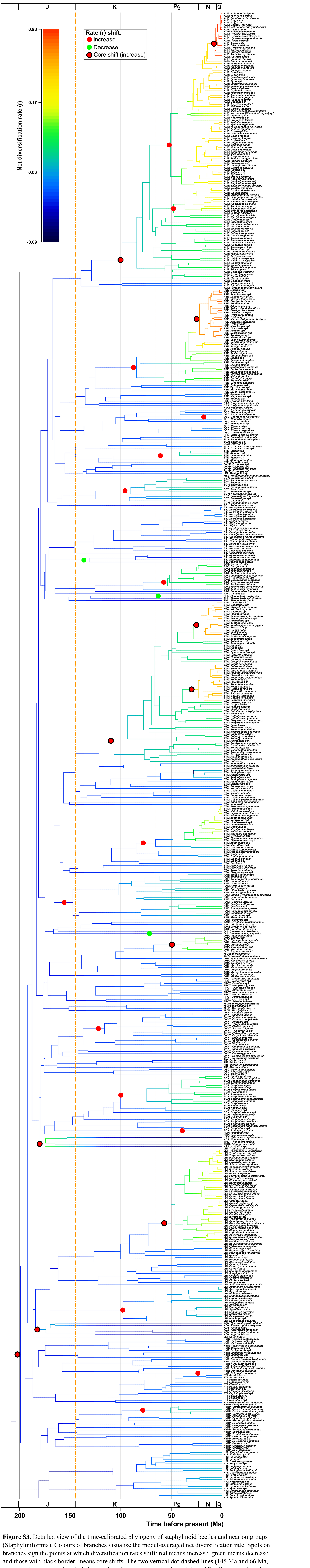

Figure S3. Detailed view of the time-calibrated phylogeny of staphylinoid beetles and near outgroups (Staphyliniformia). Colours of branches visualise the model-averaged net diversification rate. Spots on branches sign the points at which diversification rates shift: red means increase, green means decrease, and those with black border means core shifts. The two vertical dot-dashed lines (145 Ma and 66 Ma, respectively) separate the whole history into three stages: early (Jurassic), middle (Cretaceous), and late (Cenozoic).Era abbreviations: J, Jurassic; K, Cretaceous; Pg, Palaeogene; N, Neogene; Q, Quaternary.

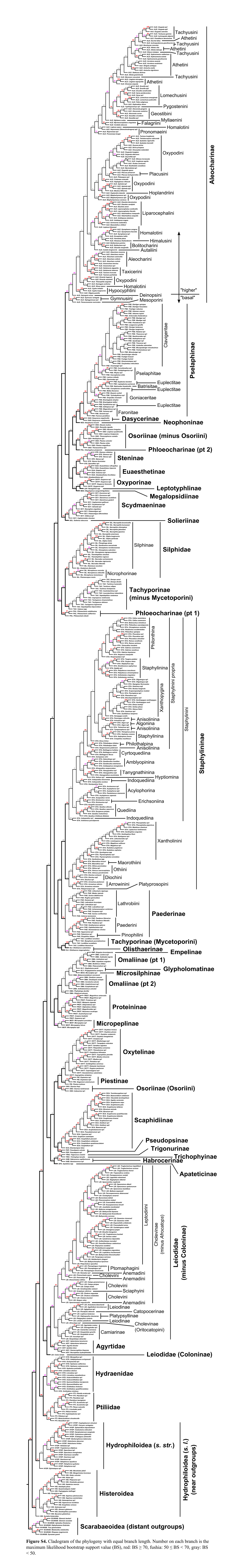

Figure S4. Cladogram of the phylogeny with equal branch length. Number on each branch is the maximum likelihood bootstrap support value (BS), red: BS ≥ 70, fushia: 50 ≤ BS < 70, gray: BS < 50.
