# Supplementary material for: Linking evolutionary mode to palaeoclimate change reveals rapid radiations of staphylinoid beetles in low-energy conditions
Source: Curr Zool. 2019 Oct 22;66(4):435–44. doi: 10.1093/cz/zoz053 (PMC7319441; doi:10.1093/cz/zoz053)
Supplement: zoz053_Supplementary_Data [file zoz053_supplementary_data.zip › zoz053-Suppl_Data/Appendix_S3_Phylogenetic_analyses.pdf]

Appendix S3: Phylogenetic analyses

We sampled a total of 664 species of Staphylinoidea (Table S3), including 614 representatives of 31 out of 32 staphylinid subfamilies and 5 other staphylinoid families as ingroups, 21 species of Hydrophiloidea *s. str.* and 21 species of Histeroidea as near outgroups (Staphyliniformia), and 8 species of Scarabaeoidea as distant outgroups. We used six genes: two nuclear protein-coding genes, carbamoyl-phosphate synthase domain (*CAD*) and *wingless* (*Wg*); two nuclear non-coding genes, *28S* and *18S* rDNA; two mitochondrial genes, one coding Cytochrome b (*Cyt b*) and one non-coding *16S* rDNA. All these sequences were mined from the GenBank database. For the three coding genes, we used the amino acid sequences provided by the database.

Sequences of each gene were initially aligned using E-INS-i algorithm in the online version of MAFFT v7.3 (Katoh et al. 2002; Katoh and Standley 2013) with adjustment to the direction of the first sequence, retaining gappy regions, and other alignment parameters left as default. The program MEGA v7.0.14 (Kumar et al. 2016) was used to refine the primary alignment manually and to produce single-gene matrices. Multiple genes were concatenated into a supermatrix with 7130 positions in Mesquite v3.04 (Maddison and Maddison 2016). Then we used PartitionFinder v2.0.0 (Lanfear et al.) to select both a data-partitioning scheme and a best-fitting substitution model for the three coding genes using the greedy algorithm (Lanfear et al. 2012) by Bayesian information criterion (Posada and Crandall 2001). The LG4X model was selected as best for *CAD*, JTT + Gamma for *Wg*, and MTART + Gamma for *Cyt b*. The GTR + Gamma model was suggested by the RAxML manual for the nucleotide partitions of a mixed matrix.

We performed maximum likelihood (ML) inference of the topology and number of substitutions per site along branches in RAxML v8.2.10 (Stamatakis 2014). We searched the best-scoring tree by new rapid hill-climbing algorithm and fixed long branch lengths using the branch length stealing algorithm (Stamatakis et al. 2007). The eight scarabaeoid species (distant outgroups) were set as outgroups. We executed a rapid bootstrap analysis (Stamatakis et al. 2008) (2000 replicates), not distinguishing ingroups and outgroups, and then computed the bipartition values as ML bootstrap support values (BS) for the best-scoring tree.

The phylogram with branch lengths proportional to the number of nucleotide substitutions is displayed in Figure S1, where the clades that we discuss in the following text are differentiated by colours. This tree is the one used in estimating the divergence time. The cladogram with equal branch lengths is displayed in Figure S4. Maximum likelihood bootstrap support value is shown above branches. We consider BS $\geq$ 70 to constitute strong nodal support, between 50 and 70 to constitute moderate support.

Overall, monophyly of most families and subfamilies was supported. Our phylogeny did not support the Hydrophiloidea *sensu lato* that includes Histeroidea, rather Histeroidea came out as the sister group of

Staphylinoidea plus Hydrophiloidea *sensu stricto*, though the bootstrap support for the latter clade was only 61. Each of the three superfamilies was supported by bootstrapping analysis. Although Staphylinoidea was well supported (BS = 73), the four-subfamily-group system proposed by Lawrence & Newton (1982; 1995) was not. Staphylinoidea in our phylogeny can be tentatively divided into ten clades, the last eight of which form a larger clade constituting Staphylinidae plus Silphidae.

(1) **Hydraenidae + Ptiliidae** was strongly supported (BS = 98), and the monophyly of each family was also strongly supported. This result is consistent with some previous studies (Hansen 1997; Beutel and Leschen 2005; Caterino et al. 2005; McKenna et al. 2015), except in the molecular phylogeny by Grebennikov & Newton (2009), where Ptiliidae was nested within Staphylinidae. But both in Hansen (1997) and McKenna et al. (2015), this clade was sister group with the clade Leiodidae + Agyrtidae or derived from the latter, unlike our result, in which the Hydraenidae + Ptiliidae clade is the sister group of the clade (Leiodidae + Agyrtidae clade) + (Staphylinidae + Silphidae) (BS = 72).

(2) **Agyrtidae + Leiodidae** (minus Coloninae) is a moderately supported clade that was also supported by some previous studies (Hansen 1997; Beutel and Leschen 2005; Caterino et al. 2005; McKenna et al. 2015), but was paraphyletic in Grebennikov & Newton (2009) (in both molecular and morphological phylogenies). *Colon* in McKenna et al. (2015) was sister to Hydraenidae + Ptiliidae (with only moderate support), whereas our result places *Colon* (or maybe Coloninae) as sister to the remaining Leiodidae + Agyrtidae clade, which is consistent with Lawrence et al. (2011), though in all cases the support for the position of *Colon* is only weak to moderate. Within Leiodidae, Camiarinae was clustered as a clade but is not well-supported (BS = 49); Platypsyllinae includes three inquiline genera, only two of them clustered strongly together here, and it, as well as Catopocerinae, is intermixed with Leiodinae. These subfamilies were not clearly resolved in our phylogeny. Cholevinae is a strongly supported monophyletic subfamily, except for the exclusion of *Afrocatops* (Oritocatopini), although no strong conflicting support unites that genus with any other group. These results also turned up in McKenna et al. (2015). Our phylogeny shows that the almost entirely troglobitic tribe Leptodirini, which is strongly supported as monophyletic (BS = 93; BS = 100 for the troglobitic clade) seems nested deep within other leiodids, which is consistent with Fresneda et al. (2011). But in our tree, Cholevini is intermixed with at least Anemadini and Sciaphyini, whereas in Fresneda et al. (2011) Anemadini and Sciaphyini were each strongly supported as monophyletic, though the relationship among the three tribes remains unclear.

(3) **"Oxytelinae +"** includes Oxytelinae, Piestinae, Osoriinae (Osoriini), Scaphidiinae, Pseudopsinae, Trigonurinae, Trichophyinae, Habrocerinae, and Apateticinae, with very low support. This group includes the main components of the former oxyteline group. The monophylies of Oxytelinae (BS = 39) and Piestinae (BS = 23) were poorly supported. Monophyly of Scaphidiinae was supported despite the lack of 28S gene for

Brachynopus latus, to the detriment of nodal support (BS = 49, but 93 without *Brachynopus latus*). The placement of Scaphidiinae has been unsettled (see Lawrence and Newton 1982; Lawrence and Newton 1995; Hansen 1997; Caterino et al. 2005; Grebennikov and Newton 2012); our results support Grebennikov & Newton (2012) and the Bayesian tree of McKenna et al. (2015). The splitting of Osoriinae was also found by McKenna et al. (2015); Newton (1982) noted a difference in larval tentorial structure between Osoriini and the others, but a thorough phylogenetic analysis of Osoriinae has never been done. Grebennikov & Newton (2012) considered Osoriinae as monophyletic, but did not include any Osoriini and thus did not address this question. The other three tribes, Eleusinini, Leptochirini, and Thoracophorini, in our phylogeny remain clustered together but are nested (with low support) within the "Pselaphinae +" clade. The subclade containing Apateticinae, Habrocerinae, Trigonurinae, and Trichophyinae is without high cladal support. Apateticinae and Trigonurinae were removed from the oxyteline group and placed at the base of Staphylinidae in Grebennikov & Newton (2012), but both McKenna et al. (2015) (Apateticinae under Bayesian analysis) and our result (with very low BS) supported their close relatedness with Scaphidiinae. The near relatedness of Trigonurinae and Trichophyinae have not been found in morphological phylogenies but oddly are strongly supported by molecular studies (see McKenna et al. (2015) and BS = 89 in our study). Habrocerinae + Trichophyinae was thought to be near Aleocharinae and placed in the former tachyporine group (Lawrence and Newton 1982; Ashe and Newton 1993; Lawrence and Newton 1995; Ashe 2005), but both of them (and Trigonurinae) were near the Oxytelinae-related clade in McKenna et al. (2015) (moderate to strong support in Bayesian analysis, none in ML). The placement of Pseudopsinae has been much controversial. Grebennikov (2005) and Grebennikov & Newton (2009) placed it as sister clade of Staphylininae + Paederinae, which is consistent with the hypothesis in Lawrence & Newton (1982; 1995), but McKenna et al. (2015) supported its relatedness with Mycetoporini (Tachyporinae). Our result puts it near the former oxyteline group, but with very little support. In earlier classifications, its genera were placed in or near Oxytelinae or Piestinae (see detailed reviews by Herman (1975; 1977) and Newton (1982)). The core members of this clade should be Oxytelinae, Piestinae, and Osoriinae (Osoriini), and others are wandering but tentatively placed here.

(4) **"Omaliinae +"** combines the subfamilies recently considered near Omaliinae, *i. e.* Glypholomatinae, Microsilphinae, Empelinae, Proteininae, Micropeplinae (*e.g.*, Lawrence and Newton 1982; Lawrence and Newton 1995; McKenna et al. 2015), though with low support at best, although Micropeplinae was placed close to Pseudopsinae + Mycetoporini in McKenna et al. (2015) (Bayesian analysis only). Micropeplinae (BS = 63) and Proteininae (BS = 90) were supported as monophylies. Interestingly, Micropeplinae was placed near Pselaphinae and Neophoninae was close to Proteininae in Hansen (1997), but in our study, they exchanged their positions (with moderate support for Micropeplinae). Omaliinae in our phylogeny is not monophyletic, with Glypholomatinae, Microsilphinae, and Empelinae mixed into it. These three small subfamilies have distinguishable characters, though they share some characters with Omaliinae, and used to be a tribe of

Omaliinae (Glypholomatinae) or part of the former omaliine group (Microsilphinae and Empelinae) (see Newton and Thayer 1992; Newton and Thayer 1995; Thayer 2016). In our analysis, only Proteininae and some of its subclades, and a few small subclades of Omaliinae, are well-supported.

(5) **"Staphylininae +"**, embracing Staphylininae, Paederinae, Tachyporinae (Mycetoporini), and Olisthaerinae, is very species rich. The monophyly of Tachyporinae has been disputed in previous studies (Ashe and Newton 1993; McKenna et al. 2015). Its placement has usually been near Aleocharinae, but the subfamily divided into two distant groups in McKenna et al. (2015) and our study, with the tribe Mycetoporini separated from other members of Tachyporinae (although neither study included the tribe Megarthropsini). Our results show Mycetoporini (strongly supported) and Olisthaerinae are possibly related, but with very weak support (BS = 23). This point is somewhat consistent with Hansen (1997), which considered Mycetoporini and Olisthaerinae to be the most primitive members of the former tachyporine group but much unlike the other members. But Ashe & Newton (1993) and Ashe (2005) found Olisthaerinae as sister group to Phloeocharinae. In McKenna et al. (2015), Olisthaerinae was related to Staphylininae + Paederinae (moderately supported by Bayesian MCMC sampling but not supported by ML bootstrapping). The sister relationship of Staphylininae and Paederinae has been tested and agreed by previous studies no matter whether using molecular or morphological data (Grebennikov and Newton 2009; Solodovnikov et al. 2013; McKenna et al. 2015); it is surprising that its support was so low in our analysis. Paederinae is confirmed as monophyletic in our phylogeny, but the traditional Paederini are divided into two clades, in agreement with the recent results of Schomann & Solodovnikov (2017), though in our phylogeny Pinophilini is sister to Paederini rather than Lathrobiini. Our phylogeny shows a weakly supported Staphylininae (BS = 29) but a strongly supported Staphylinini (BS = 79), which is sister of the negligibly supported clade ((Xantholinini + Maorothiini + Othiini + Diochini) + (Arrowiini + Platyprosopini)) (BS=12; support for both subclades also low, but the individual tribes except Xantholinini are very strongly supported). Our tribal relations are consistent with the parsimony inference using larval morphological characters in Solodovnikov & Newton (2005). Within Staphylinini, the Quediina *sensu* Herman (2001) turned out to be paraphyletic according to many previous studies, and has been divided into several subtribes (Solodovnikov and Newton 2005; Chatzimanolis et al. 2010; Solodovnikov et al. 2013; Brunke et al. 2016). In our phylogeny, Indoquediina (non-monophyletic), Quediina (monophyletic except for *Korgella*), Erichsoniina, Acylophorina, Hyptiomina, Tanygnathinina, Amblyopinina, Cyrtoquediina, and two genera (*Afroquedius*, *Antimerus*) formed a basal grade below Staphylinini propria (*sensu* Brunke et al. 2016), a clade strongly supported as monophyletic (BS = 84). This clade was also supported in Solodovnikov & Newton (2005) (adult-larval combined analysis), Chatzimanolis et al. (2010), and Solodovnikov et al. (2013), as well as Brunke et al. (2016). Within Staphylinini propria, Anisolinina (one clade plus three other genera) and Staphylinina (two clades plus *Emus*) were polyphyletic, whereas Philothalpina (BS = 99), Algonina (BS = 100), Xanthopygina (BS = 99), and Philonthina (BS = 91) were each supported as monophyletic. No other

relationships among subtribes and other lineages of Staphylinini were well-supported in our study. Although the relationships are not well resolved, it seems clear that (i) Xantholinini, Maorothiini, Othiini, Diochini, Arrowiini, and Platyprosopini are separate from Staphylinini, and within Staphylinini (ii) Quediina *sensu* Herman (2001) is non-monophyletic and basal to Staphylinina, Philonthina, Xanthopygina, and Anisolinina and (iii) Staphylinini propria is well supported.

(6) **"Silphidae +"** clade was moderately supported (BS = 59 for the clade, 100 for Phloeocharinae (in part: *Phloeocharis* spp.), 99 for Tachyporinae [minus Mycetoporini], and 87 for Silphidae). This pattern first appeared in the molecular phylogeny by Grebennikov & Newton (2009) but was not supported by their morphological analysis. The close relation of *Phloeocharis* to Silphidae was also found in Caterino et al. (2005). In McKenna et al. (2015), Silphidae and Tachyporinae (minus Mycetoporini) constituted a monophyletic clade but without *Phloeocharis* as its sister group. The inclusion of Silphidae within Staphylinidae has been proposed by many studies (*e.g.*, Hansen 1997; some analyses of Beutel and Leschen 2005; molecular data of Grebennikov and Newton 2009; Lawrence et al. 2011 [<50% support]; Bayesian analysis of McKenna et al. 2015), but generally with low or inconsistent support.

(7) **"Scydmaeninae +"** clade, including Scydmaeninae and Solieriinae, was strongly supported (BS = 70) as a monophyletic group. In McKenna et al. (2015), they were also clustered in the Bayesian analysis. In Grebennikov & Newton (2009), they were nested in the former staphylinine group, but not as sister taxa. Hansen (1997), on the contrary, indicated they were greatly separated in some analyses. The close relationship of these two subfamilies was supported by characters of fossil Solieriinae (Thayer et al. 2012).

(8) **"Steninae +"** includes Steninae, Euaesthetinae, Oxyporinae, Leptotyphlinae, and Megalopsidiinae. These subfamilies all used to be placed at the base of the former staphylinine group in the Lawrence & Newton system (Grebennikov and Newton 2009). The sister relationship of Steninae and Euaesthetinae also turned up in McKenna et al. (2015) and Grebennikov & Newton (2009), but is not strongly supported in our results (BS = 38), nor are any other relationships among the subfamilies of this group. Oxyporinae and Megalopsidiinae were clustered in our phylogeny but not supported by ML bootstrapping. Our undescribed fossil of Burmese amber, however, seems to agree this hypothesis, which has a distinctive megalopsidiine appearance but possesses apical labial palpomeres enlarged as oxyporine species. But in McKenna et al. (2015), Oxyporinae had close relation to Leptotyphlinae with strong Bayesian support but low ML support, while Megalopsidiinae was related to Piestinae (Bayesian inference) or Habrocerinae (low support under ML inference). Grebennikov & Newton (2009) suggested Leptotyphlinae as sister group to (Pseudopsinae + (Staphylininae + Paederinae)).

(9) **"Pselaphinae +"** clade includes Pselaphinae, Dasycerinae, Neophoninae, Osoriinae (minus Osoriini), Phloeocharinae (*Charhyphus* related). The close relatedness of Osoriinae (minus Osoriini) and Phloeocharinae

(*Charhyphus*) was very unexpected, but had low support (BS = 43). Monophyly of the clade containing Eleusinini, Leptochirini, and Thoracophorini of Osoriinae was supported strongly (BS = 97), which is consistent with some previous studies (Grebennikov and Newton 2012; McKenna et al. 2015). The monophyly of Phloeocharinae has long been questioned (*e.g.*, Ashe and Newton 1993; McKenna et al. 2015; Thayer 2016). Although fossils of *Charhyphus* (Herman 1972 [doubtful placement]) and *Phloeocharis* (Chatzimanolis et al. 2013) have been found, genera other than these two remain little-known and lack molecular data, so the within-subfamily relationships need further research. The relatedness of Pselaphinae, Dasycerinae, and Neophoninae was strongly supported in our study (nodal BS = 99, 98, 100, 85) and indicated by some earlier studies (Lawrence and Newton 1982; Lawrence and Newton 1995; Newton and Thayer 1995; Hansen 1997 [in part]; Thayer 2016), though generally placed with Omaliinae-related subfamilies. Some recent analyses question whether this clade does belong with other members of the former omaliine group, but support for other placements is inconsistent. And as observed by us and by McKenna et al. (2015), the sequences of these three subfamilies are highly modified (resulting in extremely long branches, Figure S1) compared to other members of Staphylinoidea. Within Pselaphinae, Faronitae and Clavigeritae were strongly (BS = 99) or moderately (BS = 64) supported as monophyletic groups, but Pselaphitae, Euplectitae, and Goniaceritae seem not to be monophyletic. Parker (2016) used morphological and molecular data to build phylogenies of Pselaphinae, in which only Faronitae, Clavigeritae, and Batrisitae (only one species in our sample) were monophyletic and Faronitae was treated as basal group and the remaining were "higher Pselaphinae". In McKenna et al. (2015), the ML tree (Pselaphinae not monophyletic) disagreed with the Bayesian tree (Pselaphinae monophyletic), but both trees agreed that Faronitae was sister to Clavigeritae (a single exemplar, with extremely long branch length) and not a stem group as inferred by both morphological (including fossils) and molecular (*28S*) data in Parker (2016). Our result overall agrees with the morphological tree in Parker (2016), with respect to monophyly of Faronitae and Clavigeritae, paraphyly of the other supertribes, Faronitae being the sister of the rest of the subfamily, and Clavigeritae arising from within Pselaphitae.

(10) **Aleocharinae**, a moderately supported monophyletic subfamily (BS = 66) with more than 16,000 described species, outnumbers the other family-level members in Staphylinoidea and even Staphyliniformia. This subfamily has usually been considered as sister or very closely related to Tachyporinae (especially non-Mycetoporini) (Ashe and Newton 1993; Ashe 2000; Ashe 2005; Elven et al. 2010; Elven et al. 2012; Cai and Huang 2015; Thayer 2016), but McKenna et al. (2015) did not find that. In our results, no significant support links Aleocharinae to any other groups of Staphylinidae. Ashe (2000) divided this subfamily into two stems: "basal" Aleocharinae (comprising Deinopsini, Gymnusini, and Mesoporini) and "higher" Aleocharinae, which has been followed in later studies (Ashe 2005 [added Trichopseniini, but found the basal groups formed a grade]; Cai and Huang 2015). Our dataset included four genera from the "basal" groups: *Paraconosoma* (Mesoporini), *Stylogymnusa* and *Gymnusa* (Gymnusini, BS = 70), and *Deinopsis* (Deinopsini). Mesoporini +

Gymnusini were a strongly supported clade (BS = 98), but Deinopsini + higher Aleocharinae and the latter clade itself were rather weakly supported (BS = 52, 53, respectively). The relationships within the “higher” Aleocharinae are mostly unclear in our tree. Some rough patterns, however, could be found: Athetini and Oxypodini might not be monophyletic, as seen previously (*e.g.*, Elven et al. 2010; Elven et al. 2012; Osswald et al. 2013); Homalotini is not resolved as monophyletic, although there is not strong evidence against it; Tachyusini (“Tachyusina” in some classifications) and Athetini (treating Geostibini as a separate tribe, after Elven et al. (2012)) were clustered with strong support (BS = 72), and these two tribes (including *Meronera*, treated as Tachyusini by Paśnik (2010)) constitute the most derivative clade in Aleocharinae, but neither of them is monophyletic; Geostibini is clustered with Lomechusini (the “true Lomechusini” of Elven et al. (2012)) plus Pygostenini (BS = 79); that clade plus Tachyusini and Athetini are moderately supported (BS = 60); Taxicerini and Aleocharini are sister groups (BS = 72); and considering only our sampling, some tribes with ≥3 representatives, *e.g.*, Geostibini (BS = 100), Lomechusini (BS = 77), Liparocephalini (BS = 98), Taxicerini (BS = 95), and Hypocyphtini (BS = 100), were strongly supported as monophyletic. Most of these results are consistent with previous studies (Newton et al. 2000; Elven et al. 2010; Elven et al. 2012; Osswald et al. 2013).

In summary, our phylogeny supports an among-family relationship of Staphyliniformia like: ((((Staphylinidae + Silphidae, (Leiodidae, Agyrtidae)), (Hydraenidae, Ptiliidae)), Hydrophiloidea), Histeroidea). Staphylinoid families appear to be monophyletic, except that Coloninae is not inside Leiodidae and Staphylinidae (including Silphidae) has lower BS than other families. Within Staphylinidae plus Silphidae, most of the subfamilies are supported as monophyletic, except Omaliinae, Osoriinae, Tachyporinae, and Phloeocharinae. The superficial paraphyly of Glypholomatinae and Olisthaerinae is likely caused by low overlap of gene coverage between representatives of those subfamilies. Oxytelinae, Piestinae, and Staphylininae have only weak support for their monophyly, which need further investigations. It is interesting that most of the very deep and shallow nodes present high support values but the nodes ranked between family and subfamily do not, which was also found in McKenna et al. (2015) and earlier authors. Although relationships among staphylinid subfamilies remain uncertain, because of the low support values on the intermediate nodes and lack of consensus from different studies, the ten main clades and some subclades can be roughly recognized. In each of the clades, there exist species-poor basal members and species-rich derived members. This pattern suggests that staphylinoid beetles underwent multiple radiations in both sequential and parallel modes.

References

Ashe JS, 2000. Mouthpart structure of *Stylogymnusa subantarctica* Hammond, 1975 (Coleoptera: Staphylinidae: Aleocharinae) with a reanalysis of the phylogenetic position of the genus. *Zool J Linn Soc* **130**:471–498.

Ashe JS, 2005. Phylogeny of the tachyporine group subfamilies and "basal" lineages of the Aleocharinae (Coleoptera: Staphylinidae) based on larval and adult characteristics. *Syst Entomol* **30**:3–37.

Ashe JS, Newton AF, 1993. Larvae of *Trichophya* and phylogeny of the tachyporine group of subfamilies (Coleoptera: Staphylinidae) with a review, new species and characterization of the Trichophyinae. *Syst Entomol* **18**:267–286.

Beutel RG, Leschen RAB, 2005. Phylogenetic analysis of Staphyliniformia (Coleoptera) based on characters of larvae and adults. *Syst Entomol* **30**:510–548.

Brunke AJ, Chatzimanolis S, Schillhammer H, Solodovnikov A, 2016. Early evolution of the hyperdiverse rove beetle tribe Staphylinini (Coleoptera: Staphylinidae: Staphylininae) and a revision of its higher classification. *Cladistics* **32**:427–451.

Cai C-Y, Huang D-Y, 2015. The oldest aleocharine rove beetle (Coleoptera, Staphylinidae) in Cretaceous Burmese amber and its implications for the early evolution of the basal group of hyper-diverse Aleocharinae. *Gondwana Res* **28**:1579–1584.

Caterino MS, Hunt T, Vogler AP, 2005. On the constitution and phylogeny of Staphyliniformia (Insecta: Coleoptera). *Mol Phylogenet Evol* **34**:655–672.

Chatzimanolis S, Cohen IM, Schomann A, Solodovnikov AYu, 2010. Molecular phylogeny of the mega-diverse rove beetle tribe Staphylinini (Insecta, Coleoptera, Staphylinidae). *Zool Scr* **39**:436–449.

Chatzimanolis S, Newton AF, Soriano C, Engel MS, 2013. Remarkable stasis in a Phloeocharine rove beetle from the Late Cretaceous of New Jersey (Coleoptera, Staphylinidae). *J Paleontol* **87**:177–182.

Elven H, Bachmann L, Gusarov VI, 2010. Phylogeny of the tribe Athetini (Coleoptera: Staphylinidae) inferred from mitochondrial and nuclear sequence data. *Mol Phylogenet Evol* **57**:84–100.

Elven H, Bachmann L, Gusarov VI, 2012. Molecular phylogeny of the Athetini–Lomechusini–Ecitocharini clade of aleocharine rove beetles (Insecta). *Zool Scr* **41**:617–636.

Fresneda J, Grebennikov VV, Ribera I, 2011. The phylogenetic and geographic limits of Leptodirini (Insecta: Coleoptera: Leiodidae: Cholevinae), with a description of *Sciaphyes shestakovi* sp. n. from the Russian Far East. *Arthropod Syst Phylogeny* **69**:99–123.

Grebennikov VV, 2005. Older-instar larvae of Pseudopsinae (Coleoptera: Staphylinidae): morphological description of three genera and phylogenetic placement of the subfamily. *Eur J Entomol* **102**:713–724.

Grebennikov VV, Newton AF, 2009. Good-bye Scydmaenidae, or why the ant-like stone beetles should become megadiverse Staphylinidae sensu latissimo (Coleoptera). *Eur J Entomol* **106**:275–301.

Grebennikov VV, Newton AF, 2012. Detecting the basal dichotomies in the monophylum of carrion and rove beetles (Insecta: Coleoptera: Silphidae and Staphylinidae) with emphasis on the Oxyteline group of subfamilies. *Arthropod Syst Phylogeny* **70**:133–165.

Hansen M, 1997. Phylogeny and classification of the staphyliniform beetle families (Coleoptera). *K Dan Vidensk Selsk Biol Skr* **48**:1–339.

Herman LH, 1972. A revision of the rove-beetle genus *Charhyphus* (Coleoptera, Staphylinidae, Phloeocharinae). *Am Mus Novit* **2496**:1–16.

Herman LH, 1975. Revision and phylogeny of the monogeneric subfamily Pseudopsinae for the world (Staphylinidae, Coleoptera). *Bull Am Mus Nat Hist* **155**:243–317.

Herman LH, 1977. Revision and phylogeny of *Zalobius*, *Asemobius*, and *Nanobius,* new genus (Coleoptera, Staphylinidae, Piestinae). *Bull Am Mus Nat Hist* **159**:45–86.

Herman LH, 2001. Catalog of the Staphylinidae (Insecta, Coleoptera): 1758 to the end of the second millennium. *Bull Am Mus Nat Hist* **265**:1–4218.

Katoh K, Misawa K, Kuma K, Miyata T, 2002. MAFFT: A novel method for rapid multiple sequence alignment based on fast Fourier transform. *Nucleic Acids Res* **30**:3059–3066.

Katoh K, Standley DM, 2013. MAFFT multiple sequence alignment software version 7: Improvements in performance and usability. *Mol Biol Evol* **30**:772–780.

Kumar S, Stecher G, Tamura K, 2016. MEGA7: Molecular Evolutionary Genetics Analysis Version 7.0 for Bigger Datasets. *Mol Biol Evol* **33**:1870–1874.

Lanfear R, Calcott B, Frandsen B, Forthcoming P,. PartitionFinder 2: New methods for selecting partitioning schemes and models of molecular evolution for large datasets. *Prep*.

Lanfear R, Calcott B, Ho SYW, Guindon S, 2012. PartitionFinder: Combined selection of partitioning schemes and substitution models for phylogenetic analyses. *Mol Biol Evol* **29**:1695–1701.

Lawrence JF, Newton AF, 1982. Evolution and classification of beetles. *Annu Rev Ecol Syst* **13**:261–290.

Lawrence JF, Newton AF, 1995. Families and subfamilies of Coleoptera (with selected genera, notes, references and data on family-group names). In: Pakaluk J, Ślipiński SA, editors. *Biology, Phylogeny, and Classification of Coleoptera: Papers Celebrating the 80th Birthday of Roy A. Crowson*. Warszawa: Museum i Instytut Zoologii PAN. pp. 779–1006.

Lawrence JF, Ślipiński A, Seago AE, Thayer MK, Newton AF, Marvaldi AE, 2011. Phylogeny of the Coleoptera based on morphological characters of adults and larvae. *Ann Zool Warszawa* **61**:1–217.

Maddison WP, Maddison DR, 2016. Mesquite: A modular system for evolutionary analysis.

McKenna DD, Farrell BD, Caterino MS, Farnum CW, Hawks DC, Maddison DR, Seago AE, Short AEZ, Newton AF, Thayer MK, 2015. Phylogeny and evolution of Staphyliniformia and Scarabaeiformia: Forest litter as a stepping stone for diversification of nonphytophagous beetles. *Syst Entomol* **40**:35–60.

Newton AF, 1982. Redefinition, revised phylogeny, and relationships of Pseudopsinae (Coleoptera, Staphylinidae). *Am Mus Novit* **2743**:1–13.

Newton AF, Thayer MK, 1995. Protopselaphinae new subfamily for Protopselaphus new genus from Malaysia, with a phylogenetic analysis and review of the Omaliinae Group of Staphylinidae including Pselaphidae(Coleoptera). In: Pakaluk J, Ślipiński SA, editors. *Biology, Phylogeny, and Classification of Coleoptera: Papers Celebrating the 80th Birthday of Roy A. Crowson*. Warszawa: Museum i Instytut Zoologii PAN. pp. 219–320.

Newton AF, Thayer MK, Ashe JS, Chandler DS, 2000. 22. Staphylinidae. In: Arnett RH, Thomas MC, editors. *American Beetles. Archostemata, Myxophaga, Adephaga, Polyphaga: Staphyliniformia*. Vol. 1. Boca Raton, London, New York, and Washington D.C.: CRC Press. pp. 272–418.

Newton AFJ, Thayer MK, 1992. Current classification and family-group names in Staphyliniformia Coleoptera. *Fieldiana Zool New Ser* **67**:1–92.

Osswald J, Bachmann L, Gusarov VI, 2013. Molecular phylogeny of the beetle tribe Oxypodini (Coleoptera: Staphylinidae: Aleocharinae). *Syst Entomol* **38**:507–522.

Parker J, 2016. Emergence of a superradiation: Pselaphine rove beetles in mid-Cretaceous amber from Myanmar and their evolutionary implications. *Syst Entomol* **41**:541–566.

Paśnik G, 2010. Phylogeny and generic classification of Tachyusini (Coleoptera, Staphylinidae: Aleocharinae).

Posada D, Crandall KA, 2001. Selecting the best-fit model of nucleotide substitution. *Syst Biol* **50**:580–601.

Schomann AM, Solodovnikov A, 2017. Phylogenetic placement of the austral rove beetle genus *Hyperomma* triggers changes in classification of Paederinae (Coleoptera: Staphylinidae). *Zool Scr* **46**:336–347.

Solodovnikov AYu, Newton AF, 2005. Phylogenetic placement of Arrowinini trib.n. within the subfamily Staphylininae (Coleoptera: Staphylinidae), with revision of the relict South African genus *Arrowinus* and description of its larva. *Syst Entomol* **30**:398–441.

Solodovnikov AYu, Yue Y, Tarasov S, Ren D, 2013. Extinct and extant rove beetles meet in the matrix: Early Cretaceous fossils shed light on the evolution of a hyperdiverse insect lineage (Coleoptera: Staphylinidae: Staphylininae). *Cladistics* **29**:360–403.

Stamatakis A, 2014. RAxML version 8: A tool for phylogenetic analysis and post-analysis of large phylogenies. *Bioinformatics* **30**:1312–1313.

Stamatakis A, Blagojevic F, Nikolopoulos DS, Antonopoulos CD, 2007. Exploring new search algorithms and hardware for phylogenetics: RAxML meets the IBM cell. *J VLSI Signal Process Syst Signal Image Video Technol* **48**:271–286.

Stamatakis A, Hoover P, Rougemont J, 2008. A rapid bootstrap algorithm for the RAxML web servers. *Syst Biol* **57**:758–771.

Thayer MK, 2016. Staphylinidae Latreille, 1802. In: Beutel RG, Leschen RAB, editors. *Coleoptera, Beetles - Volume I. Morphology and Systematics (Archostemata, Adephaga, Myxophaga, Polyphaga partim) (2nd edition). Handbook of Zoology - Arthropoda: Insecta*. Berlin/Boston: Walter de Gruyter. pp. 394–344.

Thayer MK, Newton AF, Chatzimanolis S, 2012. *Prosolierius*, a new mid-Cretaceous genus of Solieriinae (Coleoptera: Staphylinidae) with three new species from Burmese amber. *Cretac Res* **34**:124–134.
